# Immunoglobulin recognition of fecal bacteria in stunted and non-stunted children: findings from the Afribiota study

**DOI:** 10.1186/s40168-020-00890-1

**Published:** 2020-07-27

**Authors:** Kelsey E. Huus, André Rodriguez-Pozo, Nathalie Kapel, Alison Nestoret, Azimdine Habib, Michel Dede, Amee Manges, Jean-Marc Collard, Philippe J. Sansonetti, Pascale Vonaesch, B. Brett Finlay, for the Afribiota Investigators, Emilson Jean Andriatahirintsoa, Alexandra Bastaraud, Jean-Marc Collard, Maria Doria, Serge Ghislain Djorie, Aurélie Etienne, Brett Finlay, Tamara Giles-Vernick, Jean-Chrysostome Gody, Bolmbaye Privat Godje, Ionela Gouandjika-Vassilache, Francis Allan Hunald, Nathalie Kapel, Jean-Pierre Lombart, Alexandre Manirakiza, Synthia Nazita Nigatoloum, Lisette Raharimalala, Maheninasy Rakotondrainipiana, Rindra Randremanana, Harifetra Mamy Richard Randriamizao, Frédérique Randrianirina, Annick Robinson, Pierre-Alain Rubbo, Philippe Sansonetti, Laura Schaeffer, Inès Vigan-Womas, Sonia Sandrine Vondo, Pascale Vonaesch, Laura Wegener-Parfrey

**Affiliations:** 1 Michael Smith Laboratories, University of British Columbia, Vancouver, BC Canada; 2 Department of Microbiology and Immunology, University of British Columbia, Vancouver, BC Canada; 3 Unité de Pathogénie Microbienne Moléculaire, Institut Pasteur, Paris, France; 4 Laboratoire de coprologie fonctionnelle, APHP.SU, Hôpital de la Pitié-Salpêtrière, Paris, France; 5 Unité des Helminthiases, Institut Pasteur de Madagascar, Antananarivo, Madagascar; 6 Laboratoire d’Analyse médicale, Institut Pasteur de Bangui, Bangui, Central African Republic; 7 School of Population and Public Health, University of British Columbia, Vancouver, BC Canada; 8 Unité de Bactériologie Expérimentale, Institut Pasteur de Madagascar, Antananarivo, Madagascar; 9 Current address: Center for Microbes, Development and Health, Institut Pasteur de Shanghai, Shanghai, China; 10 Current address: Human and Animal Health Unit, Swiss Tropical and Public Health Institute & University of Basel, Basel, Switzerland; 11 Department of Biochemistry and Molecular Biology, University of British Columbia, Vancouver, BC Canada

**Keywords:** Microbiome, Immunoglobulin A, Immunoglobulin G, Undernutrition, Stunting, Environmental Enteric dysfunction, Sub-Saharan Africa

## Abstract

**Background:**

Child undernutrition is a global health issue that is associated with poor sanitation and an altered intestinal microbiota. Immunoglobulin (Ig) A mediates host-microbial homeostasis in the intestine, and acutely undernourished children have been shown to have altered IgA recognition of the fecal microbiota. We sought to determine whether chronic undernutrition (stunting) or intestinal inflammation were associated with antibody recognition of the microbiota using two geographically distinct populations from the Afribiota project. Fecal bacteria from 200 children between 2 and 5 years old in Antananarivo, Madagascar, and Bangui, Central African Republic (CAR), were sorted into IgA-positive (IgA+) and IgA-negative (IgA−) populations by flow cytometry and subsequently characterized by 16S rRNA gene sequencing to determine IgA-bacterial targeting. We additionally measured IgG+ fecal bacteria by flow cytometry in a subset of 75 children.

**Results:**

Stunted children (height-for-age z-score ≤ −2) had a greater proportion of IgA+ bacteria in the fecal microbiota compared to non-stunted controls. This trend was consistent in both countries, despite the higher overall IgA-targeting of the microbiota in Madagascar, but lost significance in each country individually. Two of the most highly IgA-recognized bacteria regardless of nutritional status were *Campylobacter* (in CAR) and *Haemophilus* (in both countries), both of which were previously shown to be more abundant in stunted children; however, there was no association between IgA-targeting of these bacteria and either stunting or inflammatory markers. IgG-bound intestinal bacteria were rare in both stunted and non-stunted children, similar to levels observed in healthy populations.

**Conclusions:**

Undernourished children carry a high load of intestinal pathogens and pathobionts. Our data suggest that stunted children have a greater proportion of IgA-recognized fecal bacteria. We moreover identify two putative pathobionts, *Haemophilus* and *Campylobacter*, that are broadly targeted by intestinal IgA. This study furthers our understanding of host-microbiota interactions in undernutrition and identifies immune-recognized microbes for future study.

## Background

Undernutrition is responsible for nearly half of all deaths in children less than five years old [1]. Surprisingly, even when undernourished children are fed nutrient-rich therapeutic foods, they often fail to regain normal height and weight [2]; it has been estimated that the universal implementation of all existing nutritional interventions would only reduce stunting by one-third [3]. This suggests that factors other than diet contribute to malnutrition. Currently, the contribution of non-dietary factors to stunting represents an area of intensive research and interest for the prevention and treatment of undernutrition, and the intestinal microbiota has been increasingly implicated [4–6].

Undernutrition is associated with dysbiosis and immaturity of the gut microbiota [7–10] and increased susceptibility to diarrheal disease [11]. Recently, we showed that stunted children have a dysbiotic fecal microbiota characterized by an overabundance of pathobionts and of bacteria typically associated with the oral cavity, including *Haemophilus*, *Campylobacter*, and *Escherichia-Shigella* [12]. Using duodenal aspirates to sample the microbiota of the upper gastrointestinal tract, we further showed that small intestinal bacterial overgrowth (SIBO) was common and that oral taxa were abundant in the upper gastrointestinal tract of stunted children [12].

In low-income regions with poor sanitation, undernutrition is frequently associated with chronic intestinal inflammation and permeability, a condition referred to as environmental enteropathy or environmental enteric dysfunction (EED) [9, 10, 13]. EED is hypothesized to interfere with nutrient absorption and thus exacerbate undernutrition, but it is a heterogeneous condition that is difficult to define or diagnose, and its etiology is unclear [5]. Nevertheless, multiple studies have found an association between intestinal inflammation and linear growth in children [5], indicating that intestinal homeostasis is important for child nutrition.

Immunoglobulin (Ig) A regulates host-bacterial homeostasis in the mammalian gut [14, 15]. Studies of IgA-targeted bacteria indicate that acute undernutrition and inflammatory bowel disease are both associated with altered interactions between IgA and the intestinal microbiota [16–19]. Acutely malnourished children had higher IgA-targeting of *Bacteroidales* and *Escherichia coli*, while patients with inflammatory bowel disease (IBD) had higher targeting of a variety of bacteria including *Haemophilus* and *E. coli* [16–18], and these IgA+ bacteria induced intestinal inflammation and disease when transferred into germ-free mice*.* Moreover, chronic undernutrition in mice directly alters IgA recognition of the microbiota, in part by driving bacterial dietary adaptation [19]. Here, we investigated whether chronic undernutrition and EED are also associated with altered Ig-recognition of the microbiota in children, using two geographically distinct populations from the Afribiota study.

## Results

### IgA+ fecal bacteria are higher in stunted children

**Fig. 1:**
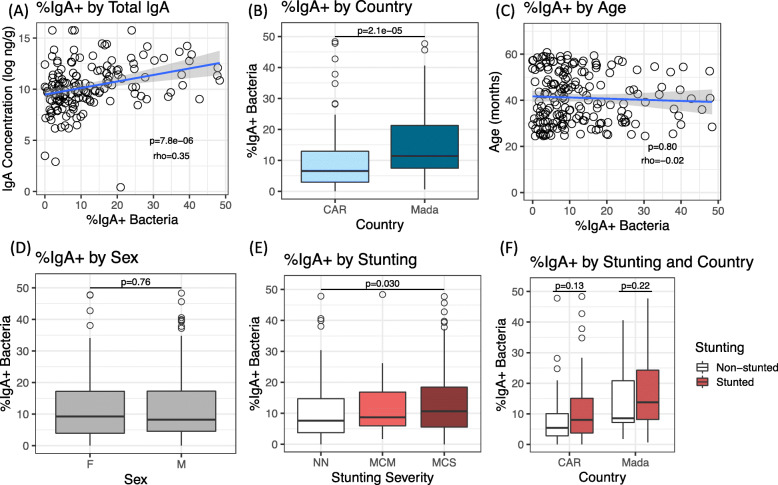
IgA-coating of fecal bacteria. The percentage of IgA-positive (%IgA+) fecal bacteria was measured by flow cytometry relative to an isotype control. **a–d** The relationship between %IgA+ bacteria and **a** the total concentration of fecal IgA in each sample (ng/g of feces, wet weight); **b** the country of origin; **c** child age in months and **d** sex. **e** %IgA+ bacteria by stunting severity. NN, non-stunted; MCM, moderately stunted; MCS, severely stunted. **f** Percent IgA-targeting by stunting status in each country individually. Mada, Madagascar; CAR, Central African Republic. *N* = 188 total children; *N* = 93 in Madagascar and *N* = 95 in CAR. Significance was determined by Spearman’s correlation (**a**, **c**), Wilcoxon rank sum test (**b**, **d**, **f**), and Jonkheere’s trend test (**e**). A linear fit is shown in **a** and **c**.

Two hundred children aged 2 to 5 years old who were enrolled in the Afribiota study in Madagascar and the Central African Republic (CAR) [20] were selected for analysis of the IgA-targeted microbiota (Fig. S1). IgA-coating of fecal bacteria was measured using flow cytometry (Fig. S2), and cytometric data was ultimately available for 188 children of whom 98 were stunted and 90 were non-stunted (Fig. S1). The proportion of fecal bacteria coated by IgA (IgA+) varied widely, from zero to nearly 50%, which is consistent with findings from other human studies [15, 17]. The percentage of IgA+ bacteria in a sample by FACS correlated strongly with total fecal IgA levels (Fig. 1a, *p* = 7.8e−06, rho = 0.35 by Spearman’s correlation), validating our technique and indicating a real biological variation in the availability of secretory IgA within the gut.

The population of IgA+ fecal bacteria was significantly higher in children from Madagascar than in children from CAR (Fig. 1b, *p* = 2.1e−05 by Wilcoxon rank sum test). It did not vary by age or by sex (Fig. 1c, d). Interestingly, there was a modest association between IgA-coating and height-for-age z-score (HAZ; Fig. S3A, *p* = 0.039, rho = −0.019 by Spearman’s correlation), with stunted children having a greater proportion of IgA-coated bacteria in the fecal microbiota compared to non-stunted controls (Fig. S3B, *p* = 0.029 by Wilcoxon rank sum test). The proportion of IgA-coated bacteria also increased stepwise with the severity of stunting (Fig. 1e, *p* = 0.030 by Jonkheere’s non-parametric trend test). Further, IgA-coating was increased in stunted children compared to non-stunted children in both countries individually, although it did not reach statistical significance in the country subsets (Fig. 1f; *p* = 0.13 and *p* = 0.22 by Wilcoxon rank sum test in Madagascar and in CAR, respectively). Removing 12 outliers also led to borderline significance (*p* = 0.054 by Wilcoxon rank sum test of stunted vs non-stunted in the pooled dataset). This may be partly due to reduced statistical power in smaller sample sizes but could also reflect bias in the data. Due to the large number of samples, IgA-sorting was performed in two major batches over several days each: although there was some variation in the data by batch, trend directions by stunting and by country were consistent in both main sorting batches (Fig. S3C–F). Further, the association between %IgA+ and HAZ was robust to non-parametric bootstrapping when including country (*p* = 0.045) or batch (*p* = 0.042) as grouping factors to constrain permutations.

Total fecal IgA was higher in Madagascar than in CAR, mirroring the flow cytometry data, but to a much smaller degree (Fig. S3G, *p* = 0.072 by Wilcoxon rank sum test). In contrast, there was no difference in total fecal IgA by stunting (Fig. S3H, *p* = 0.571 by Wilcoxon rank sum test).

We additionally did not find any association between %IgA-targeting and the inflammatory “EED” biomarkers fecal calprotectin, fecal alpha-1-antitrypsin (AAT), or serum C-reactive protein (CRP), although all trends were positive (Fig S4A–C; *p* = 0.862 for CRP by Wilcoxon rank sum test; *p* = 0.139 and *p* = 0.103 for AAT and calprotectin, respectively, by Spearman’s correlation).

Together, our data indicate a large difference in IgA recognition of fecal microbiota between geographically distinct populations. IgA recognition of fecal bacteria may moreover be higher in chronically undernourished children; however, larger sample sizes are needed to validate this result.

### Highly IgA-targeted taxa include Firmicutes, Prevotella, and Haemophilus

To characterize the IgA-targeted microbiota, 16S rRNA gene sequencing of the V4 region was applied to the sorted IgA+ and IgA− fractions in a method known as “IgA-SEQ” [16]. After filtering and rarefaction, a total of 138 children had valid sequencing data from both the IgA+ and IgA− fractions (Fig. S1), allowing us to calculate a log-normalized IgA Index as reported previously [16]. Demographics of this IgA-SEQ subpopulation are reported in Table 1 and Table S1.

**Table 1:** Demographics of stunted and non-stunted children with valid IgA-seq data (*N* = 138)

Description	Non-stunted (*N* = 67)	Stunted (*N* = 71)	*P*
Country			
Madagascar	35/67 (52.2%)	43/71 (60.6%)	0.3909
Central African Republic (CAR)	32/67 (47.8%)	28/71 (39.4%)	
Sex			
Male	29/67 (43.3%)	35/71 (49.3%)	0.4993
Female	38/67 (56.7%)	36/71 (50.7%)	
Age			
Median (months)	42.7	38.4	0.3553
2–3 years	24/67 (35.8%)	30/71 (42.2%)	
3–4 years	18/67 (26.9%)	17/71 (23.9%)	
4–5+ years	25/67 (37.3%)	24/71 (33.8%)	
Nutritional status			
Median HAZ score	− 1.05	− 3.48	*2.2e−16*
Median WHZ score	− 0.21	− 0.54	0.0600
Median hemoglobin (g/100 mL serum)	11.6	10.9	*0.0040*
Presence of anemia	18/64 (28.1%)	34/66 (51.5%)	*0.0076*
Breastfeeding			
Median duration (months)	20	20	0.8842
Inflammation			0.4979
Median AAT (mg/g dry fecal weight)	42.0	43.5	
Median calprotectin (μg/g dry fecal weight)	367.5	405.0	0.7026
Elevated CRP (> 10 mg/l serum)	4/61 (6.5%)	13/66 (19.7%)	*0.0374*
Parasites			
Presence of helminths	34/59 (57.6%)	36/66 (54.5%)	0.8569
Presence of *Giardia*	11/59 (18.6%)	17/66 (25.7%)	0.3942
Sequencing batch			
Batch 1	26/67 (38.8%)	27/71 (38.0%)	1.000
Batch 2	41/67 (61.2%)	44/71 (62.0%)	

Values are presented as the group median (continuous variable) or as counts (categorical variable) with missing values excluded. Significance was determined by Wilcoxon rank sum test (continuous variable) or Fisher’s exact test (categorical variable). Hemoglobin was adjusted by altitude (− 0.2 g/100 mL in Madagascar to account for the height above sea level). Anemia was defined as an adjusted hemoglobin level below 11 g/100 mL. Elevated CRP was defined as > 10 mg/l serum. Breastfeeding duration represents the total number of months a child was previously breastfed for; all but four children had been weaned by the time of sampling

**Fig. 2:**
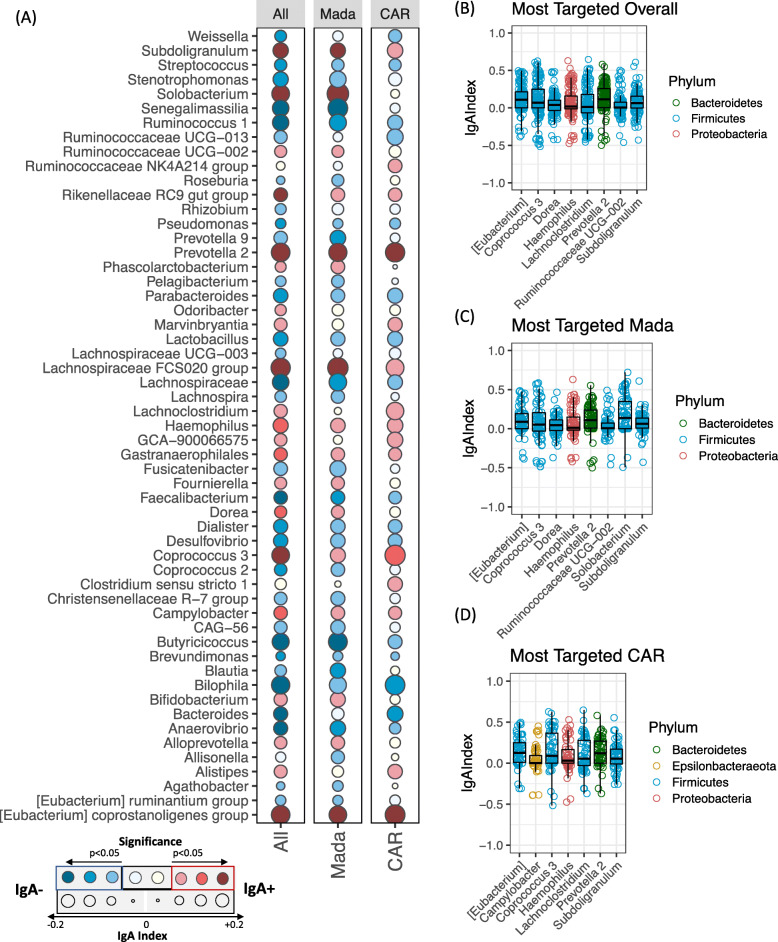
Highly IgA-targeted taxa at genus level. **a** IgA-targeting profiles at genus level in the full dataset (All), Madagascar (Mada), and Central African Republic (CAR). Genera are included if the IgA Index was significantly different from zero by a one-sided Wilcoxon (FDR-adjusted *p* < 0.05) in at least one subset. Color of circles indicates the direction of IgA-targeting (red = positive IgA Index, blue = negative IgA Index), and saturation of the color represents FDR-corrected statistical significance. The size of the circle indicates overall effect size as measured by average IgA Index. **b–d** Most highly IgA-targeted taxa in **b** the full dataset, **c** Madagascar, and **d** CAR, as defined by a median IgA Index greater than zero with FDR-adjusted *p* < 0.05. *N* = 138 total children; *N* = 78 in Madagascar and *N* = 60 in CAR

IgA-targeting of the microbiota was taxonomically diverse, differing between closely related amplicon sequence variants (ASVs) as has been reported in previous studies (Fig. S5A) [17, 21, 22]. At the genus level, twenty-one taxa were considered to be significantly IgA-targeted in the total dataset (Fig. 2a; FDR-adjusted *p* < 0.05 by one-sided Wilcoxon rank sum test, and mean IgA Index > 0). Of these, 12 (57.1%) belonged to the *Firmicutes* phylum, 5 (23.8%) to the *Bacteroidetes*, and the remainder to the *Proteobacteria*, *Actinobacteria*, *Cyanobacteria*, and *Epsilonbacteraeota*. The distribution was similar at the ASV level (Fig. S5A), with 24 out of 35 highly targeted ASVs (68.6%) belonging to the *Firmicutes*. These data are consistent with previous findings that *Firmicutes* are the most frequently IgA-recognized taxa in human feces [15].

To identify the most highly IgA-targeted taxa in a given subpopulation, we selected taxa with (a) an IgA Index significantly different from zero (FDR-adjusted *p* < 0.05) and (b) a positive median IgA Index. Notably, because IgA-targeting is highly variable between individuals, the use of median IgA index excluded many taxa identified above where targeting may have been driven by a small number of individuals. By these criteria, therefore, eight genera were highly and consistently targeted in the total study population: six members of the phylum *Firmicutes* (*Eubacterium*, *Coprococcus*, *Dorea*, *Lachnoclostridium*, unclassified *Ruminococcaceae* UCG-002, and *Subdoligranulum*), one *Bacteroidetes* (*Prevotella* 2), and one *Gammaproteobacterium* (*Haemophilus*) (Fig. 2b)*.* Most of these taxa were targeted in both countries: *Eubacterium*, *Coprococcus*, *Subdoligranulum*, *Prevotella* 2, and *Haemophilus* were significantly targeted in Madagascar and in CAR (Fig. 2c, d). In addition, *Solobacterium* was highly recognized in Madagascar (Fig. 2c), and *Campylobacter* was highly recognized in CAR (Fig. 2d)*.* These patterns were similar at the ASV level and often seemed to be driven by one or two dominant ASVs (Fig. S5B–D). A larger number of taxa met the criteria for “consistently untargeted” (FDR < 0.05 and median negative IgA index) than for significantly targeted, in the pooled dataset and in each country individually, including *Streptococcus*, *Faecalibacterium*, *Pseudomonas*, and several *Alphaproteobacteria* of likely environmental origin (Table S2). As expected, for most taxa, there was no relationship between IgA-targeting and their relative abundance in the unsorted intestinal microbiota of the same children; the exception was a negative correlation in *Faecalibacterium* (Fig. S6A; FDR-corrected *p* = 0.003, rho = − 0.35 by Spearman’s correlation).

Together, these data suggest a relatively conserved pattern of IgA-targeting in Madagascar and CAR, with high IgA recognition of *Haemophilus*, *Prevotella*, and multiple *Firmicutes*.

### IgA-targeting of putative pathobionts does not correlate with stunting or inflammation

Two of the most targeted taxa, *Haemophilus* (in both countries) and *Campylobacter* (in CAR), are putative pathobionts that were previously found to be more abundant in the fecal microbiota of stunted children [12]. Both *Haemophilus* and *Campylobacter* were positively IgA-targeted in stunted and non-stunted children, regardless of country (Fig. S6B–C). Notably, *Campylobacter* was significantly IgA-positive in Madagascar (as well as in CAR) when mean IgA Index, rather than median IgA index, was used as a criterion, suggesting a positive but less well-distributed IgA recognition of this taxon (Fig. 2a). In addition, both taxa had a mean positive IgA Index in each sequencing batch, although there was some batch variability in the magnitude of the index (Fig. S6D–E). We did not find any association between the IgA Index of either *Haemophilus* or *Campylobacter* and the inflammatory biomarkers fecal calprotectin, fecal AAT, or serum CRP (Fig. S7A–F; *p* > 0.1 for all tests by Spearman’s correlation (calprotectin, AAT) or Wilcoxon rank sum test (binary CRP levels)).

Together, these data indicate that both *Campylobacter* and *Haemophilus* are highly recognized by IgA, with IgA recognition of *Haemophilus* being particularly widespread among children in Madagascar and CAR. However, there is no significant association between IgA recognition of these taxa and chronic undernutrition or inflammatory markers.

### Variation in IgA-targeting is influenced by geography, age, and breastfeeding

**Fig. 3:**
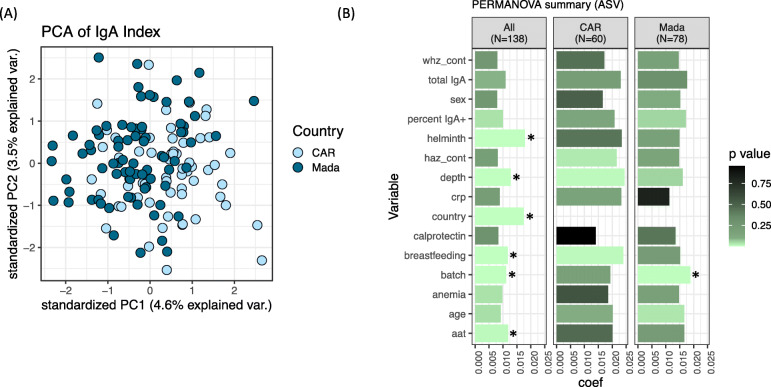
Distribution of IgA-targeting by study metadata. **a** Principal coordinate analysis based on Euclidian distance of the IgA Index, colored by country of origin. **b** Summary of PERMANOVA analysis of the IgA Index in the full dataset (All) and in each country individually. Analysis is based on taxa maintained at the ASV level. Starred variables are significant with an FDR-corrected *p* < 0.05. Each variable was tested individually in the PERMANOVA without other covariates. CAR, Central African Republic; Mada, Madacascar; Coef, the coefficient of variance by PERMANOVA. Briefly, the tested variables were as follows: whz_cont, weight-for-height z-score; total IgA, total fecal IgA (ng/g of feces, wet weight); percent IgA+, the percentage of IgA+ bacteria by flow cytometry; helminth, presence or absence of helminths; haz, height-for-age z-score; depth, the relative sequencing depth of the IgA+ fraction compared to the IgA− fraction of a given sample; crp, serum c-reactive protein in mg/l; calprotectin, fecal calprotectin in μg/g dry weight; breastfeeding, total duration of breastfeeding in months; batch, sequencing batch; anemia, presence or absence of anemia; age, child’s age in months; aat, fecal alpha-1 antitrypsin in mg/g dry weight. *N* = 138 total; *N* = 78 in Madagascar and *N* = 60 in CAR

Principle component analysis of the IgA Index shows high inter-individual variability of IgA-bacterial targeting (Fig. 3a). To identify factors which explain variation in IgA-targeting between individuals, we performed PERMANOVA analysis of the IgA Index against a set of rationally chosen metadata variables (Fig. 3b). Country of origin had a large and statistically significant effect on the IgA Index (Fig. 3b); indeed, several variables which might influence IgA or the microbiota (ex., breastfeeding duration and helminth carriage) differed significantly by country (Table S1). In addition to a strong country effect, sources of technical variation (sequencing batch and the relative sequencing depth of the IgA+ and IgA− fractions before rarefaction) influenced the IgA Index (Fig. 3b). Notably, sorting and sequencing batches largely corresponded (i.e., the same samples were included in each), and therefore, we have only included one batch variable here, although both processes might be contributing to the batch effect. To account for country and batch variation in PERMANOVA, we repeated this analysis with (a) these variables as grouping factors to constrain permutations (using the “strata” parameter) and (b) analysis performed separately in each country and in each batch (fully stratified data). Significance was determined by an FDR-corrected *p* < 0.05.

None of the tested measures of nutrition (HAZ, weight-for-height z-score (WHZ), anemia) explained variation in the IgA Index in either full or stratified datasets (Fig. 3b). Note that WHZ was within the normal range for this study and that no children were considered acutely undernourished; however, WHZ tended to be lower in stunted children (Table 1) and was therefore included for analysis. The inflammatory markers calprotectin and CRP were also non-significant. Fecal AAT was significant in the pooled dataset and when permutations were constrained by country (Fig. S8A); however, AAT was not a significant factor in either country individually (Fig. 3b). There was also some variability in the significance of these nutritional and inflammatory markers between batches (Fig. S8A), indicating noisiness in the data. Thus, nutritional or inflammatory markers measured in this study do not have a consistent measurable influence on the IgA-targeting of fecal bacteria.

Helminth carriage was also significant in pooled analysis but was almost fully confounded by country (88.5% prevalence in Madagascar versus 2.1% in CAR; Table S1) and was no longer significant when data were stratified or constrained by country (Fig. 3b, S8A).

The prior duration of breastfeeding (i.e., total months the child was breastfed for; all but four children had been weaned by the time of sampling) was also significant in pooled analysis but differed by country (Table S1). Duration of breastfeeding was no longer significant in country-constrained PERMANOVA after FDR correction; however, it was a marginally significant factor in CAR alone (FDR-adjusted *p* = 0.06).

In general, PERMANOVA results for these variables were similar if data were binned taxonomically at the genus level rather than the ASV level (Fig. S8B).However, in genus-level data, age was a statistically significant factor after FDR correction in the full dataset (Fig. S8B) and was marginally significant in Madagascar alone (FDR-adjusted *p* = 0.084). Age was also a marginally significant factor at the ASV level but did not pass FDR correction (FDR-adjusted *p* = 0.07, *p* = 0.23, and *p* = 0.35 in the pooled dataset, Madagascar, and CAR, respectively).

Taken together, variation in IgA-targeting among children in this population is strongly influenced by geography. Age and prior duration of breastfeeding might also contribute, but the influence of these variables is less significant and differs by country. In contrast, markers of undernutrition and inflammation do not show an important influence.

### IgA-targeting of specific taxa differs by geography but not by stunting

We further explored whether the IgA-targeting of any individual taxa was correlated with chronic undernutrition or with additional factors identified by PERMANOVA (country of origin, age, breastfeeding duration). Reflecting the contribution of technical factors, a handful of taxa correlated significantly with batch effect by FDR-corrected Wilcoxon Rank Sum test (Table S3); of these, however, most had already been identified as “significantly untargeted” by IgA (Table S2), underlining the utility of IgA-SEQ in distinguishing host-interacting bacteria.

The IgA-targeting of several taxa differed significantly by country in FDR-corrected linear models adjusted for sequencing depth, batch effect, age, and sex (Fig. S8C–F). Although linear models were used as an exploratory approach to allow for the inclusion of covariates, we verified that significant hits were also robust to non-parametric methods, including non-parametric bootstrapping of the model and basic uncorrected Wilcoxon rank sum test (all taxa in Fig. S8C–F remained significant by these tests at *p* < 0.05; see R Markdown for further detail). Interestingly, differences in IgA Index by country did not seem to reflect differences in unsorted relative abundance of the same taxa (Fig. S8G–J), although differences in the processing of these two datasets could have biased detection. Notably, in the case of *Solobacterium* ASV 461 (Fig. S8C, G), this 16S sequence was barely detected in the unsorted relative abundance data (Fig. S8G).

No taxa were correlated with age or with breastfeeding in the full dataset at FDR-corrected *p* < 0.05. However, at a more relaxed cutoff of *p* < 0.1, the IgA Index of *Intestinimonas* was negatively correlated with age in a linear model adjusted for sequencing depth, batch effect, country of origin, and sex of the child (FDR-adjusted *p* = 0.09). This association was also significant by uncorrected Spearman’s correlation (Fig. S9A; rho = − 0.29, *p* = 0.0004). An ASV of *Christensenellaceae* was also modestly associated with months of breastfeeding in a linear model adjusted for depth, batch, country, age, and sex (FDR-adjusted *p* = 0.09). However, *Christensenellaceae* was not significant by uncorrected Spearman’s (Fig. S9B; rho = 0.14, *p* = 0.10 in the full dataset). Both *Intestinimonas* and *Christensenellaceae* were sparsely detected, and associations with these taxa were also significant after removal of zero values (supplemental R markdown; rho = − 0.50 and *p* = 0.0003 for *Intestinimonas* and age; rho = 0.24 and *p* = 0.025 for *Christensenellaceae* and breastfeeding, by Spearman’s correlation). No taxa were significant by age or by breastfeeding in either country individually.

We next looked for taxa whose IgA Index correlated with chronic undernutrition (as either a continuous variable (HAZ) or binary factor (stunting)) in linear models corrected for relative sequencing depth, batch effect, country, and age. No taxa were associated with HAZ or with stunting in the full dataset or in CAR using an FDR-adjusted *p* < 0.1. In Madagascar, a single ASV of *Lachnospiraceae* NK4A136 was negatively associated with HAZ (FDR-corrected *p* = 0.042) and with stunting status (FDR-corrected *p* = 0.012) and this association remained significant after non-parametric bootstrapping or by uncorrected Spearman’s correlation (Fig. S9C; *p* = 6.7e−05, rho = − 0.43).

Together, these data indicate that the IgA-targeting of specific taxa varies strongly by geography. In contrast, while a small number of taxa correlate with age, breastfeeding status, and HAZ, these correlations are inconsistent by geography and should be interpreted with caution.

### IgG targeting of fecal bacteria is rare in stunted and non-stunted children

**Fig. 4:**
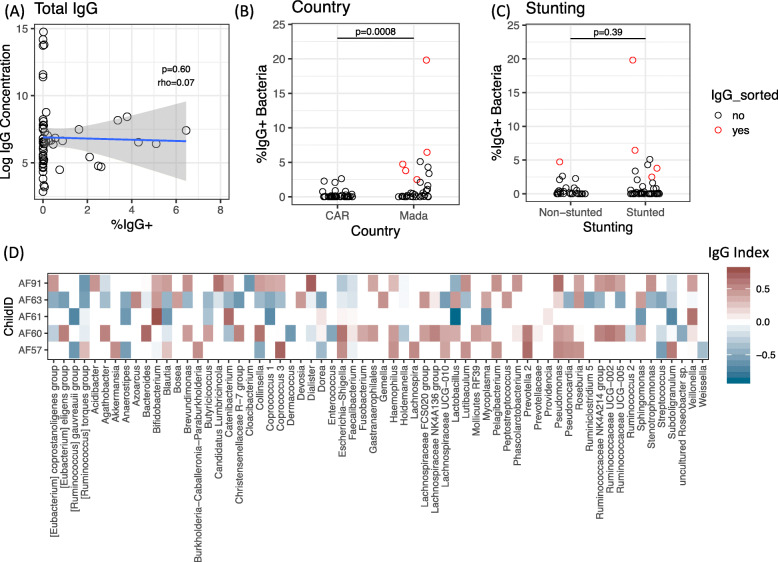
IgG targeting of the fecal microbiota. The percentage of IgG-positive (%IgG+) fecal bacteria by flow cytometry relative to an isotype control and **a** total IgG concentration (ng/g wet weight); **b** country of origin and **c** stunting status. *N* = 75. Significance was determined by Spearman’s correlation (**a**) and Wilcox rank sum test (**b**, c). Red datapoints in **b** and **c** indicate the five children selected for IgG-SEQ analysis. **d** Top IgG-targeted taxa in five children with high IgG-coating of the fecal microbiota. Color saturation reflects the numerical IgG Index (red = positively targeted, blue = negatively targeted). The top 10 most and least-targeted taxa in each child were selected to display in the heatmap.

Given that bacterially-targeted IgG is elevated in the intestines of IBD patients [23] and that many biomarkers of intestinal inflammation appear to be shared between EED and IBD [24], we investigated whether IgG targeting of intestinal bacteria was similarly elevated in this population. Fecal samples from 75 children were screened, and most samples demonstrated little to no measurable IgG+ bacteria when compared to an isotype control. However, a small number of samples did show IgG-coated bacterial populations, with 12 out of 75 children having an IgG+ population ≥ 2% above background and one child displaying a population of nearly 20% IgG+ bacteria (Fig. 4). IgG-coating did not correlate with levels of total IgG in the feces (Fig. 4a; *p* = 0.6 and rho = 0.07 by Spearman’s correlation). Fecal IgG+ bacteria were significantly higher in children from Madagascar than CAR (Fig. 4b, *p* = 0.0008 by Wilcoxon rank sum test), similar to the observed increase in IgA+ bacteria in these children (Fig. 1b). There was no significant relationship between IgG+ bacteria and stunting (Fig. 4c; *p* = 0.39 by Wilcoxon rank sum test) or markers of inflammation (*p* = 0.425 and *p* = 0.347 for calprotectin and AAT, respectively, by Spearman’s correlation and *p* = 0.152 for CRP by Wilcoxon rank sum test). However, all of these observations should be interpreted with caution due to the outlier-skewed distribution of the data.

We selected five highly IgG-targeted samples for a pilot analysis of IgG-targeted microbiota in this population (data points highlighted in red, Fig. 4b, c). All of these children were from Madagascar; four out of five were severely stunted; two reported a history of diarrheal illness in early life; and three had higher than average AAT in the fecal sample (Table S4). IgG+ and IgG− populations of these five children were sorted, sequenced, and used to calculate an IgG Index analogous to the IgA Index above. IgG+ bacteria in these children included many members of the normal and IgA+ microbiota, including multiple *Clostridiales* and *Prevotellaceae*, as well as *Bifidobacterium* and *Escherichia-Shigella* (Fig. 4d). Although the sample size is too small for statistical analysis, these data support previous reports that IgG broadly recognizes many members of the intestinal microbiota [22]. Together, our findings indicate that unlike IBD, chronic undernutrition and EED are not generally associated with elevated IgG-coating of the microbiota; however, occasional IgG+ populations exist, and IgG may broadly recognize fecal bacteria.

## Discussion

Studies on the microbiota of undernourished children have established a signature of dysbiosis that includes increased abundance of a variety of pathogens and pathobionts and that may drive the inflammatory intestinal condition called EED [10, 12, 25–27]. IgA is a critical mediator of intestinal homeostasis, and recent studies suggest that IgA recognition of the intestinal microbiota is altered during undernutrition and intestinal inflammation [16–19]. We examined IgA-bacterial targeting in almost 200 stunted and non-stunted children across two study sites, constituting one of the largest datasets examined to date and providing valuable insights into IgA-bacterial interactions during chronic undernutrition.

We found here that the overall proportion of fecal microbes bound by IgA was slightly but significantly higher in stunted children. Child stunting has been previously correlated with increased serum antibodies against flagellin and LPS [28–30]. Interestingly, high IgA recognition of fecal bacteria is also consistently observed in IBD patients, who exhibit an intestinal dysbiosis and inflammation that shares many biomarkers with EED [17, 18, 24, 31]. High IgA recognition of intestinal bacteria in stunted children might reflect past or current disruption of intestinal homeostasis and is consistent with the increased pathogen loads of these children [25, 27]. However, although the difference observed here was significant in pooled datasets and was consistent in both countries, it was non-significant in stratified data and may have been biased by outliers. Reproduction of this finding in other populations of stunted children would therefore be valuable.

We did not find that IgA recognition of specific bacteria correlated with stunting or inflammatory markers. However, several of the highly IgA-recognized bacteria in both stunted and non-stunted children are noteworthy for their potential as pathogens or pathobionts. Oral bacteria have been proposed as pathobionts in both undernutrition and IBD [12, 23, 32], and several of the highly targeted genera in this study, including *Haemophilus*, *Prevotella*, *Solobacterium*, and *Campylobacter*, are traditional members of oral microbiomes [33]. Moreover, multiple species of both *Haemophilus* and *Campylobacter* cause human disease. Both *Haemophilus* and *Campylobacter* are more abundant in stunted children of the larger Afribiota population [12], and *Campylobacter* has been strongly and consistently associated with linear growth faltering in children [12, 34, 35], suggesting an important relationship with undernutrition. Both *Haemophilus* and *Campylobacter* are additionally associated with host inflammation, including the species *H. parainfluenzae* to which the ASV observed here was most closely related [34, 36, 37]. In fact, in a population of adults from high-income countries, highly IgA-coated *H. parainfluenzae* was unique to IBD patients [17]. Overall, these data thus highlight immunogenic members of the microbiota, to which children in Madagascar and CAR are widely exposed.

It is noteworthy that *Haemophilus*, in particular, seems to have an important functional relationship with IgA. Abundance of intestinal *Haemophilus* expands in patients with secretory IgA deficiency [15], and commensal *Pasteurellaceae*, the family which *Haemophilus* belongs, are persistently expanded in mice born to IgA-deficient dams [38]. Indeed, pathogenic strains of *H. influenzae*, which are a major cause of bacterial pneumonia in children [39], encode IgA proteases as an important virulence factor [40]. The literature therefore supports the importance of IgA in controlling *Haemophilus* colonization, suggesting a functional relevance for the IgA-*Haemophilus* interaction observed so pervasively in this population.

Another major finding of this study was the large difference by geography. Total IgA recognition of fecal bacteria was significantly higher in Madagascar than in CAR; in addition, several taxa were differentially targeted. The unsorted fecal microbiota of children in Madagascar and CAR differs significantly [12], and many environmental and immunological differences exist between these populations (Table S1). This makes it difficult to conclude whether differences in IgA-bacterial targeting are driven by host or microbial factors. However, as has been observed previously, IgA-targeting tended not to correlate with taxonomic abundance [19, 21]. One notable difference between the two study populations is the burden of intestinal helminths, which are almost absent in CAR due to successful deworming campaigns but are widespread in Madagascar [41]. Helminths are known to elicit robust IgA responses, and some of this induced IgA has off-target specificity to the bacterial microbiota [42], offering a possible explanation for the increased IgA-targeting in Madagascar. Although it is not possible to infer causality, these data show clearly for the first time that IgA-targeting patterns are different between geographically distinct populations. In addition, we saw trends in IgA-targeting by both age and history of breastfeeding, two factors well known to influence the development of the microbiota and of the mucosal immune system [21, 43, 44].

Although IgA is the major mucosal antibody, other isotypes may also bind to the intestinal microbiota [45]. In addition to IgA, bacterially-targeted IgG is elevated in the intestines of IBD patients [31, 46], and specific taxa may be differentially recognized by IgG during IBD [23, 47]. As noted, many biomarkers of intestinal inflammation are shared between EED and IBD, such as elevated fecal calprotectin and blunting of small intestinal villi [24]: we therefore hypothesized that children with EED markers might also have increased IgG-recognized fecal bacteria. However, fecal IgG-coating of the microbiota was low in almost all samples measured, similar to values reported in healthy human populations [22]. Interestingly, a small number of samples in Madagascar did have measurable IgG-coating of the microbiota; two out of five of these samples had reported a previous diarrheal infection and another two had elevated levels of fecal inflammatory markers (AAT or calprotectin). We speculate that transient IgG-coating reflects recent or ongoing intestinal infections. IgG targeting in this small set of samples also supports findings that IgG, like IgA, recognizes diverse members of the bacterial microbiota [22, 48].

Our study has several limitations. Ig Index data is extremely heterogeneous and sparse, and popular analysis tools like Deseq2 cannot be applied to normalized data like the log-adjusted IgA Index, making it challenging to find appropriate statistical methods. Published studies that have detected phenotypes by IgA-SEQ have used relatively lenient cut-offs for differences in the IgA Index, including defining “IgA-targeted” as any taxon recognized in at least one patient [17] and using the microbiota of a single twin pair replicated multiple times into recipient gnotobiotic mice for analysis [16]. Our FDR-corrected models were comparatively stringent and were further limited by the use of non-parametric methods; thus, although our sample size was large for a study of this kind, we were likely underpowered to detect small differences after FDR correction. We may therefore have underestimated differences in IgA-targeting by stunting or inflammation. We additionally had significant technical variation between sorting and sequencing batches, and although we attempted to correct and account for this as rigorously as possible, technical variation further limited our statistical power. Of note, we also found that the IgA Index was influenced by the difference in sequencing depth between the IgA+ and IgA− fractions, even after rarefaction; to our knowledge, this depth bias has not been previously accounted for in IgA-SEQ datasets. Nevertheless, our study benefited from the acknowledgement of these technical biases, as well as from a relatively large sample size and from geographically distinct populations which allowed us to find reproducible trends. Given the high inter-individual variability in bacterial IgA-targeting, we feel the detection of consistent signatures between countries is particularly valuable.

## Conclusions

A correlation between undernutrition and poor sanitation has now been recognized for many years, but it is not certain how these factors are linked or how they relate to the heterogenous disease EED [5]: a better understanding of host-microbial interactions during human undernutrition is required. Our data suggest that stunted children have a greater proportion of IgA-recognized bacteria in the fecal microbiota; validation of this finding in other populations would be valuable. We moreover identify two putative pathobionts, *Haemophilus* and *Campylobacter*, that are highly targeted by intestinal IgA. These data improve our understanding of immune-microbiota interactions during undernutrition and may inform causative studies on the role of intestinal microbes in child growth.

## Methods

### Study participants and sample collection

Study participants were recruited as part of the Afribiota project [12, 20]. The study protocol for Afribiota was approved by the Institutional Review Board of the Institut Pasteur (2016-06/IRB), the National Ethical Review Boards of Madagascar (55/MSANP/CE) and the Central African Republic (173/UB/FACSS/CSCVPER/16), and the Human Ethics Board of the University of British Columbia (H18-01108). All participants received oral and written information about the study, and the legal representatives of the children provided written consent to participate in the study [20]. Subject characteristics such as age, sex, and breastfeeding history were assessed by standardized questionnaire. The detailed inclusion and exclusion criteria and recruitment procedures of the Afribiota study are described in the published protocol [20].

All children were aged 2–5 years living in Antananarivo, Madagascar, and in Bangui, Central African Republic. Children were categorized as either stunted cases (height-for-age z-score ≤ − 2) or non-stunted controls (height-for-age z-score > − 2), using the World Health Organization (WHO) Anthro Software and growth standards [49]. For the analysis presented in this study, samples from 100 children in Madagascar and 100 children in CAR were selected for IgA-sorting and sequencing. Participants were selected from the full study based on sample availability; within these constraints, samples were divided equally between stunted and non-stunted children and matched for country, sex, and age (± 3 months). Stunted children were initially included if they met the criteria for severe stunting (height-for-age z-score ≤ − 3) in order to better differentiate cases and controls; however, 12 out of 96 stunted children were re-categorized after inclusion as moderately stunted (height-for-age z-score > − 3 and ≤ − 2) and were maintained in the analysis.

Biobanking of fecal samples was performed by the Clinical Investigation and Access to BioResources Platform (ICAReB) at the Pasteur Institute, Paris, and by the Pasteur Institutes of Madagascar and of Bangui. An aliquot of fecal material for each sample was shipped on dry ice to Vancouver, Canada, and frozen immediately upon arrival at − 70 °C.

### Ig-sorting

IgA-sequencing was performed as described by Kau et al [16]. Approximately 50 mg of each fecal sample (± 10 mg) were homogenized in 1 mL of phosphate buffered saline (PBS; HyClone DPBS−/−, SH30028.02) and spun gently to settle debris; intestinal bacteria were then filtered through a 0.7-μm filter. A volume of suspension equal to 5 mg of sample was washed in FACS buffer (PBS containing 1% bovine serum albumin) and blocked for 20 min in FACS buffer containing 10% fetal bovine serum. Samples were then stained with anti-human IgA-PE (Miltenyl 130-093-128) or an isotype control (eBioscience, 12-4714-42) at 1:25 dilution for 30 min in the dark. For IgG-sorting, an anti-human IgG Fc APC (BioLegend 409306) and isotype control (BioLegend Mouse IgG2a kappa isotype, 400222) were used at the same 1:25 dilution. Samples were washed twice more and fixed overnight in 2% paraformaldehyde (PFA) at 4 °C in the dark without shaking. The next day, the PFA was washed off and samples were stained with SYTO-BC (1:4000 dilution) for bacterial DNA, washed again, and sorted by flow cytometry into Ig-positive and Ig-negative populations. A minimum of 50 000 events are collected in the IgA+ and IgA− fraction and frozen at − 20 °C for further analysis. Each sample was stained with both an anti-human IgA antibody and an isotype control to distinguish between specific and non-specific binding, and the final percentage of IgA-positive bacteria was reported after subtraction of the isotype-positive population. Samples in which the isotype and antibody-specific populations could not be distinguished were excluded from further sequencing analysis.

### 16S Library preparation

Sorted bacterial suspensions were boiled for 15 min at 100 °C, and 2 μL of lysate was used as template for 16S PCR, using Illumina-tagged and barcoded primers specific for the 16S V4 region [50]. PCR was performed with Phusion polymerase under the following cycling conditions: 5 min initial denaturation at 98 °C, 30 cycles of 20 s at 98 °C, 15 s at 55 °C, 30 s at 72 °C, and 10 min final extension at 72 °C. Reactions were run on a gel to ensure successful amplification and were purified and normalized using the 96well Sequel-Prep kit (ThermoFisher A1051001). All reactions were subsequently pooled and gel extracted (GeneJet K0692) to remover primer-dimers. Sequencing was performed on an Illumina MiSeq using a v2 kit for 2x250 bp reads with 30% PhiX at the Biomedical Research Centre (BRC) Sequencing Core of the University of British Columbia.

### Bioinformatics analysis of 16S rRNA data

Demultiplexed forward reads were analyzed in QIIME2 (https://qiime2.org/) [51], using the Dada2 option [52] for sequence quality control and trimming to 250 bp. Taxonomic assignment was performed using the SILVA database [53]. Further filtering was then performed in R using phyloseq [54]. Filtering included removal of unintended targets (archaea, mitochondria, and chloroplast), removal of singleton taxa, and rarefaction to 5000 reads. A log-adjusted IgA index was calculated as described previously [16, 21]: $Ig{A}_{Index}=-\left(\frac{\log \left({IgA}^{+} taxon\right)-\log \left({IgA}^{-} taxon\right)}{\log \left({IgA}^{+} taxon\right)+\log \left({IgA}^{-} taxon\right)}\right)$, after adding a pseudocount of 0.0000001 relative abundance to allow for zero values. Taxa were maintained at either the ASV level or the genus level for calculation of the IgA Index. IgA Index data was further filtered for prevalence within each sequencing batch, by excluding taxa in which ≥ 75% of samples had zero values in either batch individually. Multiple additional statistical methods were explored to further reduce batch effect (ex., ComBat package in R, percentile normalization in qiime2), but were discarded as they did not substantially reduce batch effect in our data. Instead, sequencing batch was taken as a covariate in all models (see statistical analysis) and analyses were repeated to ensure similar trends in each batch individually. Raw sequencing data has been deposited to the Sequence Read Archive (SRA) under BioProject PRJNA603512 and is available at the following reviewer-access link: https://dataview.ncbi.nlm.nih.gov/object/PRJNA603512?reviewer=vk3tkm0bkrm8nbqkegvmtqs5vs.

### Quantification of total fecal IgA and IgG

For total Ig quantification, 200 mg of fecal samples were homogenized in 1 mL of PBS containing Protease Inhibitor Cocktail (Roche Diagnostics GmbH, Mannheim, Germany), incubated for 30 min on ice, and centrifuged for 10 min at 10 000 g at 4 °C. The supernatant was taken for analysis and stored at − 80 °C. The concentration of antibodies (IgA, IgG1, IgG2, IgG3, IgG4) was measured on supernatants at a dilution of 1/100–1/10000 with the Bio-Plex Pro Human Isotyping Panel (Bio-Rad Laboratories, Hercules, CA, USA) using the DropArray method (Curiox Biosystems Pte Ltd, Singapore) on a Bio-Plex 200 machine (LUMINEX) according to manufacturer instructions. Values were measured in duplicates, and all samples with more than 20% difference in between the two measurements or values below or above the standard curve were repeated. Final values were normalized to the initial fecal weight used for extraction. Analysis was performed with Bio-Plex Manager Software version 6.1.1.

### Assessment of inflammatory markers

Fecal calprotectin and alpha-1 antitrypsin (AAT) were measured as described previously [55]. Briefly, stool samples were diluted 1:5 in 0.15 M NaCl and vortexed vigorously until complete homogenization; the homogenate was then centrifuged and the supernatant collected for analysis. Calprotectin concentrations were assayed in duplicate by sandwich ELISA using a polyclonal antibody system (Calprest; Eurospital, Italy) according to the manufacturer’s instructions. Fecal alpha-1 antitrypsin (AAT) was measured using an immuno-nephelemetric method adapted on the BN ProSpec system (Siemens, Germany) [55].

To measure serum C-reactive protein (CRP), venous blood was collected through the AFRIBIOTA project using Ethylene Diamine Tetra Acetic Acid (EDTA) microtainer tubes (BD Vacutainer). C-reactive protein (CRP) was measured at the Clinical Biology Center of the Institut Pasteur the Madagascar and the Laboratoire d’Analyse Médicale at the Institut Pasteur de Bangui within 4 h after blood collection according to accredited methods.

### Helminth detection

Fecal samples were examined microscopically for the presence of helminths using the Merthiolate-Iodine-Formaldehyde (MIFs) and Kato-Katz (KK) techniques [56]. Briefly, MIF solution (200 mL merthiolate tincture, 25 mL formalin 10%, 5 mL glycerin, 250 mL distilled water) was prepared, and fecal samples were triturated in the MIF solution in a haemolysis tube at approximately 250 mg stool per 2.5 mL. Stool was allowed to stand in the MIF solution at room temperature for 30 min. Using a Pasteur pipette, the stool was then removed and deposited in a microscope slide, and the sample was visually examined for the presence of helminth eggs or larva, and on the surface of the sediment for protozoa trophozoites, cysts, or oocysts. For KK technique, a solution of 3% malachite green-glycerol solution was prepared in advance and cellophane strips the size of a slide were immersed in the solution for 24 h prior to use. After thorough homogenization of the samples, approximately 1 gram of feces was placed on a tissue paper and covered with a wire mesh. With the aid of a spatula, pressure was applied and the feces that passed through the mesh were deposited onto a standard template holding 41.7 mg of feces located on a glass slide. The template was then removed, and the sample was spread onto a strip of cellophane paper embedded in the 3% malachite green-glycerol solution. Finally, the preparation was left at rest for at least 1 h before observation under optical microscope (× 10 objective) for the visual identification of helminth eggs.

### Statistical analysis

All statistical analysis was performed in R studio using the phyloseq and ggplot2 packages as indicated in the attached R markdown file (Supplemental File 1). Unless otherwise indicated, all analyses were performed both in the pooled dataset and in each country individually, as well as in each batch individually, and multiple correction of statistical tests was applied using the false discovery rate (FDR). Data structure of the IgA Index was explored using a principle component analysis based on Euclidean distance. A PERMANOVA analysis was iteratively applied to each variable of interest to determine its contribution to the distribution of the IgA Index. Non-parametric Wilcoxon rank sum tests were used to compare data with two groups (ex. stunted versus non-stunted or Madagascar versus CAR). A one-sided Wilcoxon rank sum test was used to identify taxa with an IgA Index significantly different from zero. Spearman’s correlation was applied for continuous variables. To look for associations between the IgA Index and metadata of interest, we iteratively fitted a linear model correcting for confounders (batch, relative sequencing depth (the difference in sequencing depth between IgA+ and IgA− fractions for a given taxon), country, age, and sex) for each individual taxon and corrected the p using FDR. Where indicated, bootstrapping analysis was further applied to models that contained covariates, in order to obtain a non-parametric estimate of the statistical significance.

## Supplementary information

**Additional file 1:.** Fig. S1. Subject inclusion/exclusion tree. Mada, Madagascar; CAR, Central African Republic; NS, non-stunted; S, stunted

**Additional file 2:.** Fig. S2 Representative flow cytometry gating. Fecal samples were processed unstained (A) or were stained with SYTO-Green FITC to detect bacterial DNA (B–C) and with an isotype control PE (B) or an anti-human IgA-PE (anti-hIgA) (C). Top panels: bacterially-sized events were selected by forward scatter (FSC) and side scatter (SSC). Bottom panels: events were further gated by FITC and PE to detect IgA+ and IgA− bacterial populations

**Additional file 3: **Fig. S3 IgA-bacterial targeting and stunting, by country and batch. (A–B) The percentage of IgA-positive fecal bacteria by flow cytometry (%IgA+) versus height-for-age z-score (HAZ) (A), and stunting status (B) in the full dataset. (C–D) Trend in stunting status by (C) major sorting batch and (D) daily sorting batch. (E–F) Country effect by (E) major sorting batch and (F) daily sorting batch. (G–H) Total fecal IgA by country (G) and stunting status (H). Mada, Madagascar; CAR, Central African Republic. *N* = 188 total samples. *N* = 93 in Madagascar and *N* = 95 in CAR; *N* = 91 in Batch 2017 and *N* = 88 in Batch 2018. An additional *N* = 9 samples were sorted in a single day in 2019 and are thus included in daily batch effect. Statistical significance was determined by Spearman’s correlation (A) and Wilcoxon Rank Sum test (B, C, E, G, H)

**Additional file 4: **Fig. S4 IgA-bacterial targeting by inflammatory markers. Associations between the percentage of IgA-positive fecal bacteria by flow cytometry (%IgA+) and serum C-reactive protein (CRP) (A), fecal alpha-1 antitrypsin (AAT) (B), and fecal calprotectin (C). *N* = 188. “High” CRP was defined as > 10 mg/l. Statistical significance was determined by Wilcoxon rank sum test (A) and Spearman’s correlation (B–C)

**Additional file 5: **Fig. S5 Highly IgA-targeted taxa at amplicon sequence variant (ASV) level. (A) IgA-targeting profiles at ASV level in the full dataset (All), Madagascar (Mada), and Central African Republic (CAR). ASVs are included if the IgA Index was significantly different from zero by a one-sided Wilcoxon (FDR-adjusted *p* < 0.05) in at least one subset. Color of circles indicates the direction of IgA-targeting (red = positively targeted, blue = negatively targeted), and saturation of the color represents FDR-corrected statistical significance. The size of the circle indicates overall effect size as measured by average IgA Index. (B–D) Most highly IgA-targeted taxa in (B) the full dataset, (C) Madagascar, and (D) CAR, as defined by a median IgA Index greater than zero with FDR-adjusted *p* < 0.05. *N* = 138 total; *N* = 78 in Madagascar and *N* = 60 in CAR

**Additional file 6: **Fig. S6 Abundance and IgA Index correlations in main targeted taxa. (A) Correlation between IgA-targeting and unsorted relative abundance at genus level. The most- and least-targeted taxa, as defined by median IgA Index in Fig. 2 and Table S1, are shown. Color represents Spearman’s rho. A star (*) represents significant correlation at FDR-corrected *p* < 0.05. (B–C) IgA Index of *Haemophilus* and *Campylobacter* by stunting status in Madagascar (Mada) and Central African Republic (CAR). (D–E) IgA Index of *Haemophilus* and *Campylobacter* by sequencing batch. Statistical significance determined by Wilcoxon Rank Sum test (B–E). *N* = 138 total; *N* = 78 in Madagascar and *N* = 60 in CAR; *N* = 53 in Batch 1 and *N* = 85 in Batch 2

**Additional file 7: **Fig. S7 IgA-targeting of *Haemophilus* and *Campylobacter* does not correlate with inflammatory markers. Serum C-reactive protein (CRP) levels (A, D), fecal calprotectin (B, E) and fecal alpha-1-antitrypsin (AAT) (C, F) by the IgA Index of *Haemophilus* (A–C) and *Campylobacter* (D–F). Statistical significance was determined by Wilcoxon Rank Sum Test (A, D) or Spearman’s correlation (B,C,E,F). “High” CRP was defined as > 10 mg/l. *N* = 138

**Additional file 8: **Fig. S8 Distribution of IgA-targeting by study metadata according to batch and country. (A) Summary of PERMANOVA analysis of the IgA Index in the full dataset when permutations were constrained by either sequencing batch (All ~Batch) or country (All ~Country) using the ‘strata’ parameter, or when analysis was performed in each batch individually (Batch1 and Batch2). Analysis is based on taxa maintained at the ASV level. (B) Summary of PERMANOVA analysis of the IgA Index in the full dataset and in each batch and country individually, based on taxa binned at the genus level. Starred variables are significant with an FDR-corrected p < 0.05. Each variable was tested individually in the PERMANOVA without other covariates. (C–F) IgA Index of taxa that different significantly by country in IgA-targeting. Statistical significance was determined by FDR-corrected linear models that incorporated sequencing depth, batch effect, age and sex. (G–J) Unsorted relative abundance of these same taxa. Numbers and letters following a genus indicate the first digits of the qiime2 feature code for a unique ASV. CAR, Central African Republic; Mada, Madacascar; Coef, the coefficient of variance by PERMANOVA; whz_cont, weight-for-height z-score; haz, height-for-age z-score; crp, serum c-reactive protein; aat, fecal alpha-1 antitrypsin. *N* = 138 total; *N* = 78 in Madagascar and *N* = 60 in CAR; *N* = 53 in Batch 1 and *N* = 85 in Batch 2.

**Additional file 9: **Fig. S9 IgA-targeting of taxa that correlated with age, breastfeeding or HAZ. Taxa in (A–C) were selected by linear models which incorporated country (A–B only), age, sex, batch and sequencing depth as covariates, at an FDR-corrected *p* < 0.1. Statistical significance and rho as presented in the plots derive from uncorrected Spearman’s correlation. All, full dataset; Mada, Madgascar; Lachno., *Lachnospiraceae. N* = 138 in full dataset (A–B); *N* = 78 in Madagascar (C)

**Additional file 10: **Table S1. Demographics of children with valid IgA-seq data (*n* = 138) by country. Values are presented as the group median (continuous variable) or as counts (categorical variable) with missing values excluded. Significance was determined by Wilcoxon Rank Sum test (continuous variable) or Fisher’s exact test (categorical variable). Hemoglobin was adjusted by altitude (− 0.2 g/100 mL in Madagascar to account for the height above sea level). Anemia was defined as an adjusted hemoglobin level below 11 g/100 mL. Elevated CRP was defined as > 10 mg/l serum. Breastfeeding duration represents the total number of months a child was previously breastfed for; all but four children had been weaned by the time of sampling.

**Additional file 11: **Table S2. Least IgA-targeted taxa at genus level, as defined by a median IgA Index < 0 and an FDR-adjusted *p* < 0.05 by one-sided Wilcoxon test. Mada, Madagascar; CAR, Central African Republic

**Additional file 12: **Table S3. Batch-associated taxa. Taxa which correlate significantly with flow cytometry sorting batch (sort) or 16S rDNA sequencing batch (seq). Batch (es) indicates which type of batch the taxon was significant in, according to Wilcoxon Rank Sum test with an FDR-corrected *p* < 0.05. Taxa in bold were also significantly untargeted by IgA

**Additional file 13:.** Table S4. Characteristics of children selected for IgG-SEQ analysis. HAZ, height-for-age z-score; WHZ, weight-for-age z-score; AAT, fecal alpha-1-antitrypsin (mg/g dry fecal weight); Calpro, fecal calprotectin (μg/g dry fecal weight); CRP, serum C-reactive protein (mg/l serum)

**Additional file 14:.** Supplementary Files. File S1. R markdown code and statistical analysis used in this study

## Data Availability

Raw sequencing data has been deposited to the Sequence Read Archive (SRA) under BioProject PRJNA603512 and is available at the following reviewer-access link: https://dataview.ncbi.nlm.nih.gov/object/PRJNA603512?reviewer=vk3tkm0bkrm8nbqkegvmtqs5vs.

## Abbreviations

IgA: Immunoglobulin A IgG Immunoglobulin G CAR Central African Republic EED Environmental enteric dysfunction IBD Inflammatory bowel disease ASV Amplicon sequence variant FDR False discovery rate HAZ Height-for-age z-score WHZ Weight-for-height z-score AAT Alpha-1-antitrypsin CRP C-Reactive protein
