# Supplementary material for: Immunoglobulin recognition of fecal bacteria in stunted and non-stunted children: findings from the Afribiota study
Source: Microbiome. 2020 Jul 27;8:113. doi: 10.1186/s40168-020-00890-1 (PMC7385872; doi:10.1186/s40168-020-00890-1)

FileS1_RCode_AfribiotaIgASEQ_June2020

Kelsey Elizabeth Huus

6/30/2020

# Set Up

## Load packages

library(phyloseq)
library(readstata13)
library(plyr)
library(dplyr)
library(tidyr)
library(vegan)
library(ggplot2)
library(ggbiplot)
library(gvlma)
library(boot)
library(clinfun)

# Percentage IgA-targeting (%IgA+) Analysis

## Import and distribution of %IgA+ data (Fig S1)

#Import metadata
metaIgA <- read.table("FinalAnalysis/metadata_2020-04-16.txt",
 sep='\t', header=TRUE)
metaIgA <- dplyr::filter(metaIgA, metaIgA$Sort=="IgApos") #to get rid of sorting duplicates per sample
metaIgA <- dplyr::filter(metaIgA, metaIgA$ChildID!="1429HMET018") #no consent
metaIgA <- dplyr::filter(metaIgA, !duplicated(metaIgA$ChildID)) #to get rid of any other duplicates


#children with valid %IgA data
metaIgA_filt <- filter(metaIgA, Percent_IgA_clean!="NA")
dim(metaIgA_filt)

## [1] 188 395

#12 children are missing %IgA data because either the isotype control or the main antibody file (.fsc) were improperly saved during sorting

#distribution of children with valid %IgA data
dplyr::count(metaIgA_filt, stunted)

## # A tibble: 2 x 2
## stunted n
## <int> <int>
## 1 0 90
## 2 1 98

dplyr::count(metaIgA_filt, Country)

## # A tibble: 2 x 2
## Country n
## <chr> <int>
## 1 Mada 93
## 2 RCA 95

dplyr::count(metaIgA_filt, Country, stunted)

## # A tibble: 4 x 3
## Country stunted n
## <chr> <int> <int>
## 1 Mada 0 41
## 2 Mada 1 52
## 3 RCA 0 49
## 4 RCA 1 46

dplyr::count(metaIgA_filt, Country, stunt_categ)

## # A tibble: 6 x 3
## Country stunt_categ n
## <chr> <chr> <int>
## 1 Mada MCM 9
## 2 Mada MCS 43
## 3 Mada NN 41
## 4 RCA MCM 3
## 5 RCA MCS 43
## 6 RCA NN 49

#concordance of batch effect #most samples were sorted and sequenced in two main batches
#sort 2017 => seq batch 1 and sort 2018 => seq batch 2
dplyr::count(metaIgA, Batch, Sorting_batch_major)

## # A tibble: 5 x 3
## Batch Sorting_batch_major n
## <chr> <int> <int>
## 1 Batch1 2017 92
## 2 Batch2 2017 1
## 3 Batch2 2018 96
## 4 Batch2 2019 9
## 5 Batch2 NA 1

#is stunting significantly different by country in this dataset
fisher.test(as.factor(metaIgA_filt$stunted), metaIgA_filt$Country)

##
## Fisher's Exact Test for Count Data
##
## data: as.factor(metaIgA_filt$stunted) and metaIgA_filt$Country
## p-value = 0.3115
## alternative hypothesis: true odds ratio is not equal to 1
## 95 percent confidence interval:
## 0.4004255 1.3672588
## sample estimates:
## odds ratio
## 0.74139

## %IgA+ Data: Fig 1 and Fig S3

#%IgA-targeting and total IgA
cor.test(metaIgA_filt$Percent_IgA_clean, metaIgA_filt$IgA_Obs_Conc, method='spearman')

##
## Spearman's rank correlation rho
##
## data: metaIgA_filt$Percent_IgA_clean and metaIgA_filt$IgA_Obs_Conc
## S = 420315, p-value = 7.816e-06
## alternative hypothesis: true rho is not equal to 0
## sample estimates:
## rho
## 0.3483044

#plot correlation: %IgA vs total IgA - Fig 1A
p <- ggplot(metaIgA_filt, aes(x=Percent_IgA_clean, y=log(IgA_Obs_Conc))) + geom_point(shape=1, size=4) + geom_smooth(method=lm)
p <- p + ggtitle("%IgA+ by Total IgA")
p <- p + theme_bw(base_size=16)
p <- p + xlab("%IgA+ Bacteria") + ylab("IgA Concentration (log ng/g)")
p <- p + annotate("text", x = 35, y=5, label = "p=7.8e-06", size=4)
p <- p + annotate("text", x = 35, y=3.5, label = "rho=0.35", size=4)
p

## `geom_smooth()` using formula 'y ~ x'


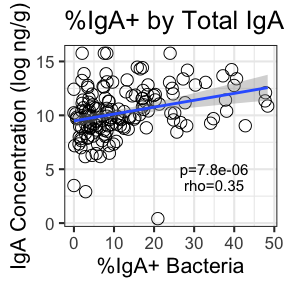


#%IgA-targeting and Country
wilcox.test(metaIgA_filt$Percent_IgA_clean~metaIgA_filt$Country)

##
## Wilcoxon rank sum test with continuity correction
##
## data: metaIgA_filt$Percent_IgA_clean by metaIgA_filt$Country
## W = 6004, p-value = 2.122e-05
## alternative hypothesis: true location shift is not equal to 0

#plot boxplot: %IgA vs Country - Fig 1B
p <- ggplot(metaIgA_filt) + geom_boxplot(aes(x=Country2, y=Percent_IgA_clean, fill=Country2),
 outlier.shape=1, outlier.size=3)
p <- p + ggtitle("%IgA+ by Country")
p <- p + theme_bw(base_size=16)
p <- p + ylab("%IgA+ Bacteria") + xlab("Country")
p <- p + scale_fill_manual(values=c("lightskyblue1", "deepskyblue4"))
p <- p + guides(colour=FALSE, size=FALSE, shape=FALSE, fill=FALSE)
p <- p + annotate("text", x = 1.5, y=52, label = "p=2.1e-05", size=4)
p <- p + annotate("segment", x = 1, xend=2, y=50, yend=50)
p


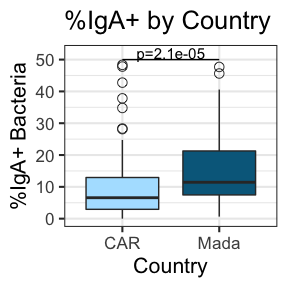


#%IgA-targeting and age
cor.test(metaIgA_filt$Percent_IgA_clean, metaIgA_filt$age, method='spearman')

##
## Spearman's rank correlation rho
##
## data: metaIgA_filt$Percent_IgA_clean and metaIgA_filt$age
## S = 1128362, p-value = 0.7967
## alternative hypothesis: true rho is not equal to 0
## sample estimates:
## rho
## -0.01891639

#plot correlation: %IgA vs age - Fig 1C
p <- ggplot(metaIgA_filt, aes(x=Percent_IgA_clean, y=age)) + geom_point(shape=1, size=4) + geom_smooth(method=lm)
p <- p + ggtitle("%IgA+ by Age")
p <- p + theme_bw(base_size=16)
p <- p + xlab("%IgA+ Bacteria") + ylab("Age (months)")
p <- p + ylim(0,70)
p <- p + annotate("text", x = 35, y=20, label = "p=0.80", size=4)
p <- p + annotate("text", x = 35, y=14, label = "rho=-0.02", size=4)
p

## `geom_smooth()` using formula 'y ~ x'


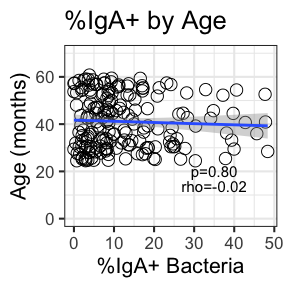


#%IgA and sex
wilcox.test(metaIgA_filt$Percent_IgA_clean~metaIgA_filt$sexe)

##
## Wilcoxon rank sum test with continuity correction
##
## data: metaIgA_filt$Percent_IgA_clean by metaIgA_filt$sexe
## W = 4529.5, p-value = 0.7567
## alternative hypothesis: true location shift is not equal to 0

p <- ggplot(metaIgA_filt) + geom_boxplot(aes(x=sexe2, y=Percent_IgA_clean),
 outlier.shape=1, outlier.size=3, fill="gray70")
p <- p + ggtitle("%IgA+ by Sex")
p <- p + theme_bw(base_size=16)
p <- p + ylab("%IgA+ Bacteria") + xlab("Sex")
p <- p + annotate("text", x = 1.5, y=52, label = "p=0.76", size=4)
p <- p + annotate("segment", x = 1, xend=2, y=50, yend=50)
p <- p + guides(colour=FALSE, size=FALSE, shape=FALSE, fill=FALSE)
p


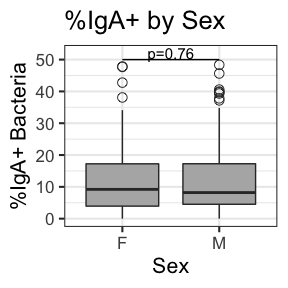


## %IgA+ Bacteria, HAZ and Stunting - including batch consistency, co-variates, and non-parametric permutation (Fig 1E-F, Fig S3A-D)

##%IgA and chronic undernutrition##
#HAZ
cor.test(metaIgA_filt$Percent_IgA_clean, metaIgA_filt$haz_cont, method='spearman')

##
## Spearman's rank correlation rho
##
## data: metaIgA_filt$Percent_IgA_clean and metaIgA_filt$haz_cont
## S = 1274601, p-value = 0.03863
## alternative hypothesis: true rho is not equal to 0
## sample estimates:
## rho
## -0.1509706

cor.test(metaIgA_filt$IgA_Obs_Conc, metaIgA_filt$haz_cont, method='spearman')

##
## Spearman's rank correlation rho
##
## data: metaIgA_filt$IgA_Obs_Conc and metaIgA_filt$haz_cont
## S = 616978, p-value = 0.5896
## alternative hypothesis: true rho is not equal to 0
## sample estimates:
## rho
## 0.04337936

#plot correlation: %IgA vs HAZ - Fig S3A
p <- ggplot(metaIgA_filt, aes(x=Percent_IgA_clean, y=haz_cont)) + geom_point(shape=1, size=4) + geom_smooth(method=lm)
p <- p + ggtitle("%IgA+ by HAZ")
p <- p + theme_bw(base_size=16)
p <- p + xlab("%IgA+ Bacteria") + ylab("Height-for-age z-score (HAZ)")
p <- p + annotate("text", x = 35, y=2, label = "p=0.039", size=4)
p <- p + annotate("text", x = 35, y=1.5, label = "rho=-0.15", size=4)
p

## `geom_smooth()` using formula 'y ~ x'


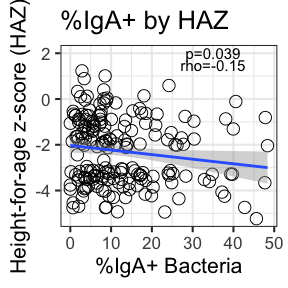


#total IgA by HAZ - Fig S4F
p <- ggplot(metaIgA_filt, aes(x=log(IgA_Obs_Conc), y=haz_cont)) + geom_point(shape=1, size=4) + geom_smooth(method=lm)
p <- p + ggtitle("Total IgA by HAZ")
p <- p + theme_bw(base_size=16)
p <- p + xlab("Log Total IgA") + ylab("Height-for-age z-score (HAZ)")
#p <- p + facet_grid(.~Country2)
p <- p + annotate("text", x = 2.5, y=0.5, label = "p=0.590", size=4)
p <- p + annotate("text", x = 2.5, y=0, label = "rho=0.04", size=4)
p

## `geom_smooth()` using formula 'y ~ x'


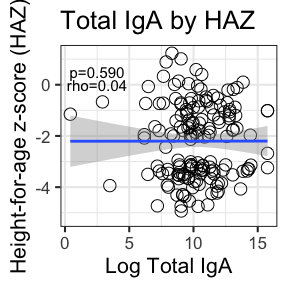


#%IgA by stunting
wilcox.test(metaIgA_filt$Percent_IgA_clean~as.factor(metaIgA_filt$stunted))

##
## Wilcoxon rank sum test with continuity correction
##
## data: metaIgA_filt$Percent_IgA_clean by as.factor(metaIgA_filt$stunted)
## W = 3596, p-value = 0.02906
## alternative hypothesis: true location shift is not equal to 0

#total IgA by stunting
wilcox.test(metaIgA_filt$IgA_Obs_Conc~as.factor(metaIgA_filt$stunted))

##
## Wilcoxon rank sum test with continuity correction
##
## data: metaIgA_filt$IgA_Obs_Conc by as.factor(metaIgA_filt$stunted)
## W = 3243, p-value = 0.5707
## alternative hypothesis: true location shift is not equal to 0

#plot boxplot: %IgA vs Stunting - Fig S3B
metaIgA_filt$stunt_categ2 <- ifelse(metaIgA_filt$stunted==0, yes="Non-stunted", no="Stunted") #recode for nicer label
p <- ggplot(metaIgA_filt) + geom_boxplot(aes(x=stunt_categ2, y=Percent_IgA_clean, fill=stunt_categ2),
 outlier.shape=1, outlier.size=3)
p <- p + ggtitle("%IgA+ by Stunting")
p <- p + theme_bw(base_size=16)
p <- p + ylab("%IgA+ Bacteria") + xlab("Stunting")
p <- p + scale_fill_manual(values=c("white", "indianred3"))
p <- p + guides(colour=FALSE, size=FALSE, shape=FALSE, fill=FALSE)
p <- p + annotate("text", x = 1.5, y=52, label = "p=0.029", size=4)
p <- p + annotate("segment", x = 1, xend=2, y=50, yend=50)
p


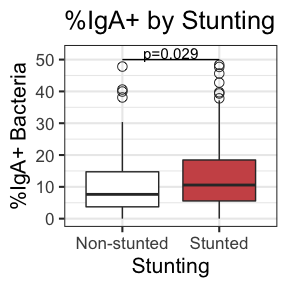


#plot boxplot: Total IgA vs Stunting - Fig S4E
metaIgA_filt$stunt_categ2 <- ifelse(metaIgA_filt$stunted==0, yes="Non-stunted", no="Stunted") #recode for nicer label
p <- ggplot(metaIgA_filt) + geom_boxplot(aes(x=stunt_categ2, y=log(IgA_Obs_Conc), fill=stunt_categ2),
 outlier.shape=1, outlier.size=3)
p <- p + ggtitle("Total IgA by Stunting")
p <- p + theme_bw(base_size=16)
p <- p + ylab("log Fecal IgA (ng/g)") + xlab("Stunting")
p <- p + scale_fill_manual(values=c("white", "indianred3"))
p <- p + guides(colour=FALSE, size=FALSE, shape=FALSE, fill=FALSE)
p <- p + annotate("text", x = 1.5, y=20, label = "p=0.571", size=4)
p <- p + annotate("segment", x = 1, xend=2, y=19, yend=19)
p


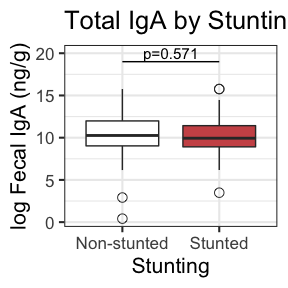


#%IgA-targeted by stunting severity (normally nourished, moderately stunted, or severely stunted)
#non-parametric ANOVA
a1 <- kruskal.test(metaIgA_filt$Percent_IgA_clean ~ as.factor(metaIgA_filt$stunt_categ),
 data=metaIgA_filt)
a1

##
## Kruskal-Wallis rank sum test
##
## data: metaIgA_filt$Percent_IgA_clean by as.factor(metaIgA_filt$stunt_categ)
## Kruskal-Wallis chi-squared = 4.7927, df = 2, p-value = 0.09105

posthoc <- pairwise.wilcox.test(metaIgA_filt$Percent_IgA_clean, as.factor(metaIgA_filt$stunt_categ),
 p.adjust.method='BH')
posthoc

##
## Pairwise comparisons using Wilcoxon rank sum test with continuity correction
##
## data: metaIgA_filt$Percent_IgA_clean and as.factor(metaIgA_filt$stunt_categ)
##
## MCM MCS
## MCS 0.867 -
## NN 0.537 0.096
##
## P value adjustment method: BH

#non-parametric trend test that takes into account ordinal data (ie MCS>MCM>NN)
#MCS = severely stunted; MCM = moderately stunted; NN = non-stunted
metaIgA_filt$stunt_categ <- factor(metaIgA_filt$stunt_categ, levels=c("NN", "MCM", "MCS"))
metaIgA_filt$stunt_categ_num <- gsub("NN", 0, metaIgA_filt$stunt_categ)
metaIgA_filt$stunt_categ_num <- gsub("MCM", 1, metaIgA_filt$stunt_categ_num)
metaIgA_filt$stunt_categ_num <- gsub("MCS", 2, metaIgA_filt$stunt_categ_num)
metaIgA_filt$stunt_categ_num <- as.numeric(metaIgA_filt$stunt_categ_num)

set.seed(123)
j <- jonckheere.test(metaIgA_filt$Percent_IgA_clean, metaIgA_filt$stunt_categ_num, alternative = "two.sided", nperm=10000)
j

##
## Jonckheere-Terpstra test
##
## data:
## JT = 5756, p-value = 0.0298
## alternative hypothesis: two.sided

#plot boxplot: %IgA vs Stunting MCM vs MCS - Fig 1E
metaIgA_filt$stunt_categ <- factor(metaIgA_filt$stunt_categ, levels=c("NN", "MCM", "MCS"))
p <- ggplot(metaIgA_filt) + geom_boxplot(aes(x=stunt_categ, y=Percent_IgA_clean, fill=stunt_categ),
 outlier.shape=1, outlier.size=3)
p <- p + ggtitle("%IgA+ by Stunting")
p <- p + theme_bw(base_size=16)
p <- p + ylab("%IgA+ Bacteria") + xlab("Stunting Severity")
p <- p + guides(colour=FALSE, size=FALSE, shape=FALSE, fill=FALSE)
p <- p + annotate("text", x = 2, y=52, label = "p=0.030", size=4)
p <- p + annotate("segment", x=1, xend=3, y=50, yend=50, colour="black")
p <- p + scale_fill_manual(values=c("white", "indianred2", "indianred4"))
p


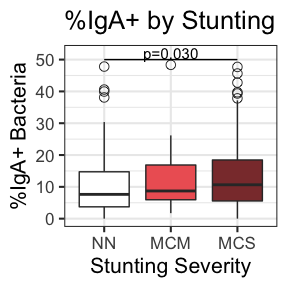


#Testing stunting/HAZ in each country individually
#Mada
metaIgA_filt_Mada <- dplyr::filter(metaIgA_filt, Country=="Mada")
cor.test(metaIgA_filt_Mada$Percent_IgA_clean, metaIgA_filt_Mada$haz_cont, method='spearman')

##
## Spearman's rank correlation rho
##
## data: metaIgA_filt_Mada$Percent_IgA_clean and metaIgA_filt_Mada$haz_cont
## S = 156891, p-value = 0.1024
## alternative hypothesis: true rho is not equal to 0
## sample estimates:
## rho
## -0.1704461

wilcox.test(metaIgA_filt_Mada$Percent_IgA_clean~as.factor(metaIgA_filt_Mada$stunted))

##
## Wilcoxon rank sum test with continuity correction
##
## data: metaIgA_filt_Mada$Percent_IgA_clean by as.factor(metaIgA_filt_Mada$stunted)
## W = 907.5, p-value = 0.2215
## alternative hypothesis: true location shift is not equal to 0

#CAR
metaIgA_filt_CAR <- dplyr::filter(metaIgA_filt, Country=="RCA") #CAR=RCA (name depends on metadata file)
cor.test(metaIgA_filt_CAR$Percent_IgA_clean, metaIgA_filt_CAR$haz_cont, method='spearman')

##
## Spearman's rank correlation rho
##
## data: metaIgA_filt_CAR$Percent_IgA_clean and metaIgA_filt_CAR$haz_cont
## S = 160867, p-value = 0.2241
## alternative hypothesis: true rho is not equal to 0
## sample estimates:
## rho
## -0.1258893

wilcox.test(metaIgA_filt_CAR$Percent_IgA_clean~as.factor(metaIgA_filt_CAR$stunted))

##
## Wilcoxon rank sum test with continuity correction
##
## data: metaIgA_filt_CAR$Percent_IgA_clean by as.factor(metaIgA_filt_CAR$stunted)
## W = 924, p-value = 0.1315
## alternative hypothesis: true location shift is not equal to 0

#Mada and CAR by stunting in the same plot - Fig 1F
p <- ggplot(metaIgA_filt) + geom_boxplot(aes(x=Country2, y=Percent_IgA_clean, fill=stunt_categ2),
 outlier.shape=1, outlier.size=3)
p <- p + ggtitle("%IgA+ by Stunting and Country")
p <- p + theme_bw(base_size=16) + ylab("%IgA+ Bacteria") + xlab("Country") + labs(fill="Stunting")
p <- p + scale_fill_manual(values=c("white", "indianred3"))
#p <- p + guides(colour=FALSE, size=FALSE, shape=FALSE, fill=FALSE)
p <- p + annotate("text", x = 1, y=52, label = "p=0.13", size=4)
p <- p + annotate("segment", x=0.75, xend=1.25, y=50, yend=50 )
p <- p + annotate("text", x = 2, y=52, label = "p=0.22", size=4)
p <- p + annotate("segment", x=1.75, xend=2.25, y=50, yend=50 )
p


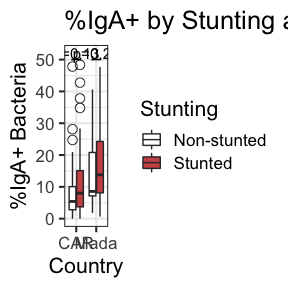


#Controlling for country & batch by non-parametric BOOTSTRAP analysis

# function to obtain regression weights
bs <- function(formula, data, indices) {
 d <- data[indices,] # allows boot to select sample
 fit <- lm(formula, data=d)
 return(coef(fit))
}

#bootstrap on regression coefficient
N1=1000 #number of permutations
N2=10000 #larger number of permutations for more precise p value

#bootstrap estimate (%IgA+ vs HAZ) without any co-variates
bootiga <- boot(data=metaIgA_filt, statistic=bs,
 R=N2, formula=Percent_IgA_clean ~ haz_cont)
bootiga

##
## ORDINARY NONPARAMETRIC BOOTSTRAP
##
##
## Call:
## boot(data = metaIgA_filt, statistic = bs, R = N2, formula = Percent_IgA_clean ~
## haz_cont)
##
##
## Bootstrap Statistics :
## original bias std. error
## t1* 9.866764 0.01822888 1.4219942
## t2* -1.162375 0.01071189 0.5775166

# get 95% confidence intervals
boot.ci(bootiga, type="all", index=2)

## BOOTSTRAP CONFIDENCE INTERVAL CALCULATIONS
## Based on 10000 bootstrap replicates
##
## CALL :
## boot.ci(boot.out = bootiga, type = "all", index = 2)
##
## Intervals :
## Level Normal Basic
## 95% (-2.305, -0.041 ) (-2.313, -0.036 )
##
## Level Percentile BCa
## 95% (-2.289, -0.012 ) (-2.336, -0.065 )
## Calculations and Intervals on Original Scale

#p-value (probability) based on estimate distribution
hist(bootiga$t[,2])


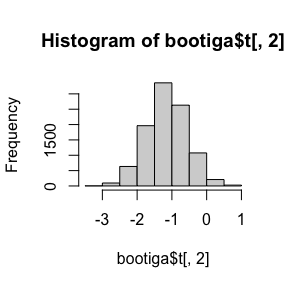


estimate <- bootiga$t[,2]
p1 = (1 - sum((mean(estimate) + estimate) < mean(estimate)) / N2) *2
p1

## [1] 0.0472

#bootstrap estimate where permutations are constrained by country
bootiga2 <- boot(data=metaIgA_filt, statistic=bs,
 R=N2, formula=Percent_IgA_clean ~ haz_cont,
 strata=as.factor(metaIgA_filt$Country))
bootiga2

##
## STRATIFIED BOOTSTRAP
##
##
## Call:
## boot(data = metaIgA_filt, statistic = bs, R = N2, strata = as.factor(metaIgA_filt$Country),
## formula = Percent_IgA_clean ~ haz_cont)
##
##
## Bootstrap Statistics :
## original bias std. error
## t1* 9.866764 -0.01307572 1.411146
## t2* -1.162375 -0.00884404 0.580866

# get 95% confidence intervals
boot.ci(bootiga2, type="all", index=2)

## BOOTSTRAP CONFIDENCE INTERVAL CALCULATIONS
## Based on 10000 bootstrap replicates
##
## CALL :
## boot.ci(boot.out = bootiga2, type = "all", index = 2)
##
## Intervals :
## Level Normal Basic
## 95% (-2.292, -0.015 ) (-2.295, 0.008 )
##
## Level Percentile BCa
## 95% (-2.332, -0.029 ) (-2.324, -0.023 )
## Calculations and Intervals on Original Scale

#p-value (probability) based on estimate distribution
hist(bootiga2$t[,2])


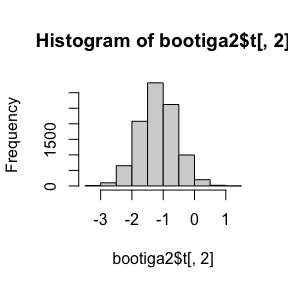


estimate <- bootiga2$t[,2]
p2 = (1 - sum((mean(estimate) + estimate) < mean(estimate)) / N2) *2
p2

## [1] 0.0446

#bootstrap estimate where permutations are constrained by sorting batch
bootiga3 <- boot(data=metaIgA_filt, statistic=bs,
 R=N2, formula=Percent_IgA_clean ~ haz_cont,
 strata=as.factor(metaIgA_filt$Sorting_batch_major))
bootiga3

##
## STRATIFIED BOOTSTRAP
##
##
## Call:
## boot(data = metaIgA_filt, statistic = bs, R = N2, strata = as.factor(metaIgA_filt$Sorting_batch_major),
## formula = Percent_IgA_clean ~ haz_cont)
##
##
## Bootstrap Statistics :
## original bias std. error
## t1* 9.866764 0.024561771 1.4347369
## t2* -1.162375 0.009023458 0.5760118

# get 95% confidence intervals
boot.ci(bootiga3, type="all", index=2)

## BOOTSTRAP CONFIDENCE INTERVAL CALCULATIONS
## Based on 10000 bootstrap replicates
##
## CALL :
## boot.ci(boot.out = bootiga3, type = "all", index = 2)
##
## Intervals :
## Level Normal Basic
## 95% (-2.300, -0.042 ) (-2.285, -0.028 )
##
## Level Percentile BCa
## 95% (-2.296, -0.040 ) (-2.344, -0.079 )
## Calculations and Intervals on Original Scale

#p-value (probability) based on estimate distribution
hist(bootiga3$t[,2])


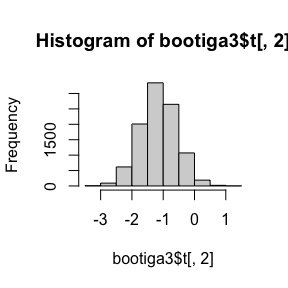


estimate <- bootiga3$t[,2]
p3 = (1 - sum((mean(estimate) + estimate) < mean(estimate)) / N2) *2
p3

## [1] 0.0422

#testing stunting in each sorting batch individually
dplyr::count(metaIgA_filt, Sorting_batch_major, stunt_categ2)

## # A tibble: 6 x 3
## Sorting_batch_major stunt_categ2 n
## <int> <chr> <int>
## 1 2017 Non-stunted 41
## 2 2017 Stunted 50
## 3 2018 Non-stunted 43
## 4 2018 Stunted 45
## 5 2019 Non-stunted 6
## 6 2019 Stunted 3

#n=9 children in 2019 #this batch is too small to compare with the two main ones - instead include 2019 sort in daily batch variation below
#note that sorting in 2017 and 2018 were grouped into ~1 week time period each, over several days
#sorting 2017
metaIgA_filt_2017 <- dplyr::filter(metaIgA_filt, Sorting_batch_major==2017)
wilcox.test(metaIgA_filt_2017$Percent_IgA_clean~as.factor(metaIgA_filt_2017$stunted))

##
## Wilcoxon rank sum test with continuity correction
##
## data: metaIgA_filt_2017$Percent_IgA_clean by as.factor(metaIgA_filt_2017$stunted)
## W = 752, p-value = 0.02973
## alternative hypothesis: true location shift is not equal to 0

dplyr::count(metaIgA_filt_2017, stunt_categ2)

## # A tibble: 2 x 2
## stunt_categ2 n
## <chr> <int>
## 1 Non-stunted 41
## 2 Stunted 50

#sorting 2018
metaIgA_filt_2018 <- dplyr::filter(metaIgA_filt, Sorting_batch_major==2018)
wilcox.test(metaIgA_filt_2018$Percent_IgA_clean~as.factor(metaIgA_filt_2018$stunted))

##
## Wilcoxon rank sum test with continuity correction
##
## data: metaIgA_filt_2018$Percent_IgA_clean by as.factor(metaIgA_filt_2018$stunted)
## W = 852.5, p-value = 0.3392
## alternative hypothesis: true location shift is not equal to 0

dplyr::count(metaIgA_filt_2018, stunt_categ2)

## # A tibble: 2 x 2
## stunt_categ2 n
## <chr> <int>
## 1 Non-stunted 43
## 2 Stunted 45

#stunting and batch in the same plot
metaIgA_filt_2017_18 <- dplyr::filter(metaIgA_filt, Sorting_batch_major%in%c(2017, 2018))
p <- ggplot(metaIgA_filt_2017_18) + geom_boxplot(aes(x=as.factor(Sorting_batch_major), y=Percent_IgA_clean, fill=stunt_categ2),
 outlier.shape=1, outlier.size=3)
p <- p + ggtitle("Major Sorting Batch")
p <- p + theme_bw(base_size=16) + ylab("%IgA+ Bacteria") + xlab(NULL)
p <- p + guides(colour=FALSE, size=FALSE, shape=FALSE, fill=FALSE)
p <- p + scale_fill_manual(values=c("white", "indianred3"))
p <- p + annotate("text", x = 1, y=52, label = "p=0.030", size=4)
p <- p + annotate("segment", x=0.75, xend=1.25, y=50, yend=50 )
p <- p + annotate("text", x = 2, y=52, label = "p=0.339", size=4)
p <- p + annotate("segment", x=1.75, xend=2.25, y=50, yend=50 )
p


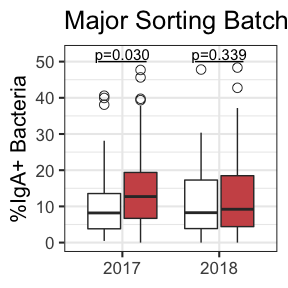


#stunting by minor day
p2 <- ggplot(metaIgA_filt) + geom_boxplot(aes(x=as.factor(Sorting_batch_minor), y=Percent_IgA_clean, fill=stunt_categ2),
 outlier.shape=NA, outlier.size=NA,)
p2 <- p2 + geom_point(aes(x=as.factor(Sorting_batch_minor), y=Percent_IgA_clean, shape=stunt_categ2),
 size=2, position=position_dodge(width=0.75))
p2 <- p2 + ggtitle("Daily Batch Effect (Stunting)")
p2 <- p2 + theme_bw(base_size=16)
p2 <- p2 + ylab("%IgA+ Bacteria") + xlab("Sorting Batch")
p2 <- p2 + scale_fill_manual(values=c("white", "indianred3"))
p2 <- p2 + theme(axis.text.x = element_text(angle=90, vjust=0.5, size=12))
p2


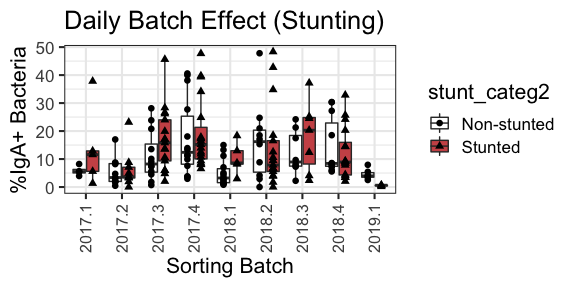


#Effect of outliers on %IgA+ vs Stunting
#based on the boxplots shown in Fig S3B, there are several outliers in both groups in the highest quartile
UpperQuantile <- quantile(metaIgA_filt$Percent_IgA_clean, probs=0.75)
LowerQuantile <- quantile(metaIgA_filt$Percent_IgA_clean, probs=0.25)
IQR <- IQR(metaIgA_filt$Percent_IgA_clean)
no_outliers <- subset(metaIgA_filt,
 metaIgA_filt$Percent_IgA_clean<(UpperQuantile+1.5*IQR)
 &metaIgA_filt$Percent_IgA_clean>(LowerQuantile-1.5*IQR)) #standard statistical definition of outliers
dim(metaIgA_filt)

## [1] 188 396

dim(no_outliers) #removed 12 values

## [1] 176 396

wilcox.test(no_outliers$Percent_IgA_clean~as.factor(no_outliers$stunted))

##
## Wilcoxon rank sum test with continuity correction
##
## data: no_outliers$Percent_IgA_clean by as.factor(no_outliers$stunted)
## W = 3219, p-value = 0.0542
## alternative hypothesis: true location shift is not equal to 0

## Further investigation of the trend by country - batch consistency & total IgA (Fig S3E-F, S4D)

#sorting 2017
wilcox.test(metaIgA_filt_2017$Percent_IgA_clean~as.factor(metaIgA_filt_2017$Country))

##
## Wilcoxon rank sum test with continuity correction
##
## data: metaIgA_filt_2017$Percent_IgA_clean by as.factor(metaIgA_filt_2017$Country)
## W = 1519.5, p-value = 0.0001082
## alternative hypothesis: true location shift is not equal to 0

dplyr::count(metaIgA_filt_2017, Country)

## # A tibble: 2 x 2
## Country n
## <chr> <int>
## 1 Mada 48
## 2 RCA 43

#sorting 2018
wilcox.test(metaIgA_filt_2018$Percent_IgA_clean~as.factor(metaIgA_filt_2018$Country))

##
## Wilcoxon rank sum test with continuity correction
##
## data: metaIgA_filt_2018$Percent_IgA_clean by as.factor(metaIgA_filt_2018$Country)
## W = 1211, p-value = 0.03882
## alternative hypothesis: true location shift is not equal to 0

dplyr::count(metaIgA_filt_2018, Country)

## # A tibble: 2 x 2
## Country n
## <chr> <int>
## 1 Mada 41
## 2 RCA 47

#both major batches by country in one plot - Fig S3E
p <- ggplot(metaIgA_filt_2017_18) + geom_boxplot(aes(x=as.factor(Sorting_batch_major), y=Percent_IgA_clean, fill=Country2),
 outlier.shape=1, outlier.size=3)
p <- p + ggtitle("Major Sorting Batch")
p <- p + theme_bw(base_size=16) + ylab("%IgA+ Bacteria") + xlab(NULL)
p <- p + scale_fill_manual(values=c("lightskyblue1", "deepskyblue4"))
p <- p + guides(colour=FALSE, size=FALSE, shape=FALSE, fill=FALSE)
p <- p + annotate("text", x = 1, y=52, label = "p=0.0001", size=4)
p <- p + annotate("segment", x=0.75, xend=1.25, y=50, yend=50 )
p <- p + annotate("text", x = 2, y=52, label = "p=0.039", size=4)
p <- p + annotate("segment", x=1.75, xend=2.25, y=50, yend=50 )
p


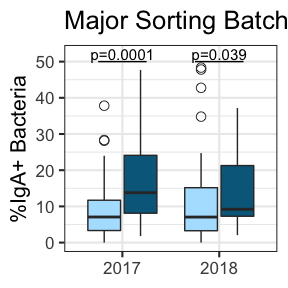


#country effect by day - Fig S3F
p2 <- ggplot(metaIgA_filt) + geom_boxplot(aes(x=as.factor(Sorting_batch_minor), y=Percent_IgA_clean, fill=Country2),
 outlier.shape=NA, outlier.size=NA,)
p2 <- p2 + geom_point(aes(x=as.factor(Sorting_batch_minor), y=Percent_IgA_clean, shape=Country2),
 size=2, position=position_dodge(width=0.75))
p2 <- p2 + ggtitle("Daily Batch Effect (Country)")
p2 <- p2 + theme_bw(base_size=16)
p2 <- p2 + ylab("%IgA+ Bacteria") + xlab("Sorting Batch")
p2 <- p2 + scale_fill_manual(values=c("lightskyblue1", "deepskyblue4"))
p2 <- p2 + theme(axis.text.x = element_text(angle=90, vjust=0.5, size=12))
p2


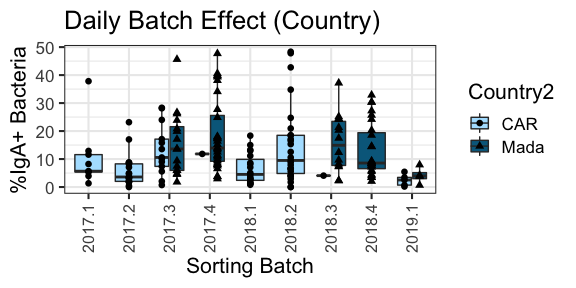


#Total IgA levels by country
wilcox.test(metaIgA_filt$IgA_Obs_Conc~metaIgA_filt$Country)

##
## Wilcoxon rank sum test with continuity correction
##
## data: metaIgA_filt$IgA_Obs_Conc by metaIgA_filt$Country
## W = 3587.5, p-value = 0.07197
## alternative hypothesis: true location shift is not equal to 0

#plot boxplot: total IgA vs Country - Fig S4D
p <- ggplot(metaIgA_filt) + geom_boxplot(aes(x=Country2, y=log(IgA_Obs_Conc), fill=Country2),
 outlier.shape=1, outlier.size=3)
p <- p + ggtitle("Total IgA by Country")
p <- p + theme_bw(base_size=16)
p <- p + ylab("log Fecal IgA (ng/g)") + xlab("Country")
p <- p + scale_fill_manual(values=c("lightskyblue1", "deepskyblue4"))
p <- p + guides(colour=FALSE, size=FALSE, shape=FALSE, fill=FALSE)
p <- p + annotate("text", x = 1.5, y=20, label = "p=0.072", size=4)
p <- p + annotate("segment", x = 1, xend=2, y=19, yend=19)
p


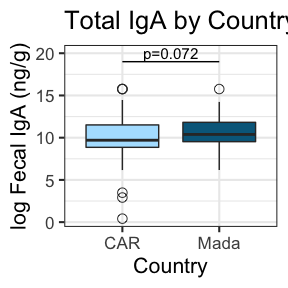


## %IgA correlations with inflammatory markers (Fig S4)

#inflammatory markers by %IgA+

#serum CRP
#maintained as binary factor because of the irregular distribution (many samples below detection limit etc.)
wilcox.test(metaIgA_filt$Percent_IgA_clean~metaIgA_filt$crp_seuil)

##
## Wilcoxon rank sum test with continuity correction
##
## data: metaIgA_filt$Percent_IgA_clean by metaIgA_filt$crp_seuil
## W = 1536.5, p-value = 0.8618
## alternative hypothesis: true location shift is not equal to 0

wilcox.test(metaIgA_filt_CAR$Percent_IgA_clean~metaIgA_filt_CAR$crp_seuil)

##
## Wilcoxon rank sum test with continuity correction
##
## data: metaIgA_filt_CAR$Percent_IgA_clean by metaIgA_filt_CAR$crp_seuil
## W = 469.5, p-value = 0.7283
## alternative hypothesis: true location shift is not equal to 0

wilcox.test(metaIgA_filt_Mada$Percent_IgA_clean~metaIgA_filt_Mada$crp_seuil)

##
## Wilcoxon rank sum test with continuity correction
##
## data: metaIgA_filt_Mada$Percent_IgA_clean by metaIgA_filt_Mada$crp_seuil
## W = 333, p-value = 0.488
## alternative hypothesis: true location shift is not equal to 0

#plot CRP by %IgA+ - Fig S4A
pdata <- metaIgA_filt %>% select(crp_seuil, Percent_IgA_clean)
pdata$CRP <- ifelse(pdata$crp_seuil=="CRP normale (<=10 mg/l)", yes="Normal", no="High")
pdata$CRP <- factor(pdata$CRP, levels=c("Normal", "High"))
pdata <- pdata[!is.na(pdata$CRP),]

p <- ggplot(pdata) + geom_boxplot(aes(x=CRP, y=Percent_IgA_clean),
 outlier.shape=1, outlier.size=3, fill="grey70"
)
p <- p + ggtitle("%IgA+ by CRP")
p <- p + theme_bw(base_size=16)
p <- p + ylab("%IgA+ Bacteria") + xlab("CRP")
#p <- p + scale_fill_manual(values=c("white", "black"))
p <- p + guides(colour=FALSE, size=FALSE, shape=FALSE, fill=FALSE)
p <- p + annotate("text", x = 1.5, y=52, label = "p=0.862", size=4)
p <- p + annotate("segment", x=1, xend=2, y=50, yend=50, colour="black")
p


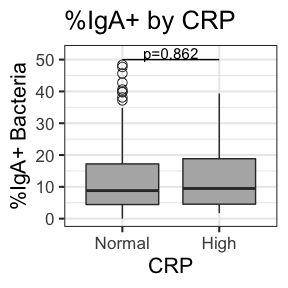


#aat
cor.test(metaIgA_filt$Percent_IgA_clean, metaIgA_filt$AATmggdePS, method='spearman')

##
## Spearman's rank correlation rho
##
## data: metaIgA_filt$Percent_IgA_clean and metaIgA_filt$AATmggdePS
## S = 625768, p-value = 0.1387
## alternative hypothesis: true rho is not equal to 0
## sample estimates:
## rho
## 0.1168473

cor.test(metaIgA_filt_CAR$Percent_IgA_clean, metaIgA_filt_CAR$AATmggdePS, method='spearman')

##
## Spearman's rank correlation rho
##
## data: metaIgA_filt_CAR$Percent_IgA_clean and metaIgA_filt_CAR$AATmggdePS
## S = 80110, p-value = 0.5905
## alternative hypothesis: true rho is not equal to 0
## sample estimates:
## rho
## 0.06106848

cor.test(metaIgA_filt_Mada$Percent_IgA_clean, metaIgA_filt_Mada$AATmggdePS, method='spearman')

##
## Spearman's rank correlation rho
##
## data: metaIgA_filt_Mada$Percent_IgA_clean and metaIgA_filt_Mada$AATmggdePS
## S = 84162, p-value = 0.453
## alternative hypothesis: true rho is not equal to 0
## sample estimates:
## rho
## 0.08401546

#plot AAT by %IgA+ - Fig S4B
p <- ggplot(metaIgA_filt, aes(x=Percent_IgA_clean, y=AATmggdePS)) + geom_point(shape=1, size=4) + geom_smooth(method=lm)
p <- p + ggtitle("%IgA+ by AAT")
p <- p + theme_bw(base_size=16)
p <- p + xlab("%IgA+ Bacteria") + ylab("AAT (mg/g)")
p <- p + annotate("text", x = 35, y=120, label = "p=0.139", size=4)
p <- p + annotate("text", x = 35, y=110, label = "rho=0.12", size=4)
p

## `geom_smooth()` using formula 'y ~ x'


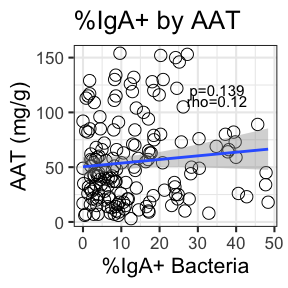


#calpro
cor.test(metaIgA_filt$Percent_IgA_clean, metaIgA_filt$CALPROTECTINEggdePS, method='spearman')

##
## Spearman's rank correlation rho
##
## data: metaIgA_filt$Percent_IgA_clean and metaIgA_filt$CALPROTECTINEggdePS
## S = 810602, p-value = 0.1032
## alternative hypothesis: true rho is not equal to 0
## sample estimates:
## rho
## 0.122892

cor.test(metaIgA_filt_CAR$Percent_IgA_clean, metaIgA_filt_CAR$CALPROTECTINEggdePS, method='spearman')

##
## Spearman's rank correlation rho
##
## data: metaIgA_filt_CAR$Percent_IgA_clean and metaIgA_filt_CAR$CALPROTECTINEggdePS
## S = 100465, p-value = 0.6333
## alternative hypothesis: true rho is not equal to 0
## sample estimates:
## rho
## 0.05217523

cor.test(metaIgA_filt_Mada$Percent_IgA_clean, metaIgA_filt_Mada$CALPROTECTINEggdePS, method='spearman')

##
## Spearman's rank correlation rho
##
## data: metaIgA_filt_Mada$Percent_IgA_clean and metaIgA_filt_Mada$CALPROTECTINEggdePS
## S = 123282, p-value = 0.8633
## alternative hypothesis: true rho is not equal to 0
## sample estimates:
## rho
## 0.01829577

#plot Calpro by %IgA+ - Fig S4C
p <- ggplot(metaIgA_filt, aes(x=Percent_IgA_clean, y=CALPROTECTINEggdePS)) + geom_point(shape=1, size=4) + geom_smooth(method=lm)
p <- p + ggtitle("%IgA+ by Calprotectin")
p <- p + theme_bw(base_size=16)
p <- p + xlab("%IgA+ Bacteria") + ylab("Calprotectin (mg/g)")
#p <- p + facet_grid(.~Country2)
p <- p + annotate("text", x = 35, y=3500, label = "p=0.103", size=4)
p <- p + annotate("text", x = 35, y=3200, label = "rho=0.12", size=4)
p

## `geom_smooth()` using formula 'y ~ x'


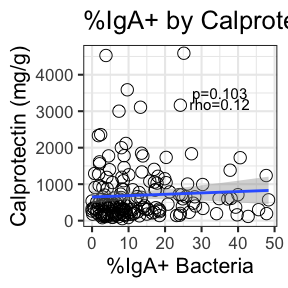


# Data processing

## Import and filter IgA-sorted 16S data using phyloseq

#Import data into phyloseq
biom<-("FilesForR/feat-with-taxonomy.biom")
featurebiom<-import_biom(biom, parseFunction = parse_taxonomy_default)
tree <- read_tree_greengenes ("FilesForR/tree.nwk")
#to export this metadata file as a .txt also
meta_phyloseq <- import_qiime_sample_data("FinalAnalysis/metadata_2020-04-16.txt")

#merge into phyloseq object
Table<-merge_phyloseq(featurebiom,meta_phyloseq,tree)
Table

## phyloseq-class experiment-level object
## otu_table() OTU Table: [ 5045 taxa and 450 samples ]
## sample_data() Sample Data: [ 450 samples by 395 sample variables ]
## tax_table() Taxonomy Table: [ 5045 taxa by 7 taxonomic ranks ]
## phy_tree() Phylogenetic Tree: [ 5045 tips and 5006 internal nodes ]

#Prune out unwanted taxa / known contaminants
taxotu <- data.frame(OTU = row.names(otu_table(Table)), tax_table(Table))
halotus <- subset(taxotu, Rank1 == "D_0__Archaea" | Rank1 == "D_0__Eukaryota" |Rank6 == "D_5__Halomonas" | Rank6 == "D_5__Shewanella" | Rank5 == "D_4__Mitochondria" | Rank5 == "D_3__Chloroplast")
halotus <- as.character(halotus[["OTU"]])
Table_pruned1 <- prune_taxa(setdiff(row.names(otu_table(Table)), halotus), Table)
Table_pruned1 <- prune_taxa(taxa_sums(Table_pruned1) > 0, Table_pruned1)

#Prune out samples with very low read counts
Table_pruned2 <- prune_samples(sample_sums(Table_pruned1)>=1000, Table_pruned1)
#also prune out corresponding sorted pair
samples_pruned <- prune_samples(sample_sums(Table_pruned1)<1000, Table_pruned1)
pairs_to_exclude <- sample_data(samples_pruned)$ChildID
Table_pruned3 = subset_samples(Table_pruned2, !(ChildID %in% pairs_to_exclude))
#remove sample missing metadata due to withdrawn consent
Table_pruned3b = subset_samples(Table_pruned3, (ChildID != "1429HMET018"))
#missing data from two sorted samples after sequencing - to exclude corresponding neg/pos
Table_pruned3b = subset_samples(Table_pruned3b, !(SampleID %in% c("HMET079neg","HMET141posBatch1")))

#some samples were resequenced across both batches; pick whichever one has the highest read count to keep
Table_pruned3c = subset_samples(Table_pruned3b, !(SampleID %in% c("CPB435pos", "CPB435neg", "CPB448pos", "CPB448neg",
 "HMET146pos", "HMET146neg", "HMET184pos", "HMET184neg",
 "HMET032neg", "HMET032pos")))

#remove singletons
Table_pruned4 = prune_taxa(taxa_sums(Table_pruned3c) > 1, Table_pruned3c)
#Table_pruned4b = prune_taxa(taxa_sums(Table_pruned3b) > 1, Table_pruned3b)

#save sampling depth information before rarefying
sample_data(Table_pruned4)$sample_sums <- sample_sums(Table_pruned4)

#rarefy to 5000 reads
set.seed(3)
Table_pruned5 <- rarefy_even_depth(Table_pruned4, sample.size = 5000)
#and the corresponding pairs
samples_pruned <- prune_samples(sample_sums(Table_pruned4)<5000, Table_pruned4)
pairs_to_exclude <- as.data.frame(sample_data(samples_pruned))$ChildID
Table_pruned6 = subset_samples(Table_pruned5, !(ChildID %in% pairs_to_exclude))
Table_pruned6

## phyloseq-class experiment-level object
## otu_table() OTU Table: [ 3970 taxa and 281 samples ]
## sample_data() Sample Data: [ 281 samples by 396 sample variables ]
## tax_table() Taxonomy Table: [ 3970 taxa by 7 taxonomic ranks ]
## phy_tree() Phylogenetic Tree: [ 3970 tips and 3936 internal nodes ]

# Create the IgA Index and filter by taxa prevalence within each batch (ASV level)

##Batch 1##
Batch1 <- subset_samples(Table_pruned6, Batch=="Batch1")
#rel abund
Ig <- transform_sample_counts(Batch1, function(OTU) OTU/sum(OTU))
#add a pseudocount to transformed samples
otu_table(Ig) <- otu_table(Ig) + 0.0000001
#subset
IgNeg_forindexB1 = subset_samples(Ig, Sort %in% c("IgAneg"))
IgPos_forindexB1 = subset_samples(Ig, Sort %in% c("IgApos"))
#make into dataframes
IgNegOTU <- data.frame(otu_table(IgNeg_forindexB1))
IgPosOTU <- data.frame(otu_table(IgPos_forindexB1))
#now the dataframes should be the same dimensions.
dim(IgNegOTU)

## [1] 3970 53

dim(IgPosOTU)

## [1] 3970 53

#but they don't seem to be in the same order. Transpose, then "order".
IgNegOTU <- t(IgNegOTU)
IgNeg_Order <- IgNegOTU[ order(row.names(IgNegOTU)), ]
IgPosOTU <- t(IgPosOTU)
IgPos_Order <- IgPosOTU[ order(row.names(IgPosOTU)), ]
#Log transform and calculate IgA Index.
IgNegLog <- log(IgNeg_Order)
IgPosLog <- log(IgPos_Order)
IgAIndexB1 <- -(IgPosLog - IgNegLog)/(IgPosLog + IgNegLog)

#trim step to remove never-targeted taxa (length-1) or taxa that are zero-targeted in at least "x"% of samples (length*x)
IgA_t <- as.data.frame(t(IgAIndexB1))
IgAB1_trim75 <- IgA_t[rowSums(IgA_t==0) <=(length(IgA_t)*0.75), ]
dim(IgA_t)

## [1] 3970 53

dim(IgAB1_trim75)

## [1] 188 53

#add full taxonomy ID to the IgA Index
IgTAX <- as.data.frame(IgPos_forindexB1@tax_table@.Data)
IgTAX$ASV <- row.names(IgTAX)
IgTAX$combined <- paste(IgTAX$Rank1, IgTAX$Rank2, IgTAX$Rank3, IgTAX$Rank4, IgTAX$Rank5, IgTAX$Rank6, IgTAX$Rank7, IgTAX$ASV, sep="|")

TaxaToKeep75 <- row.names(IgAB1_trim75)
IgTAX75_B1 <- filter(IgTAX, ASV %in% TaxaToKeep75)
row.names(IgAB1_trim75) <- IgTAX75_B1$combined

#will generally want to have it transposed so that you can filter by ID and add metadata
IgAB1_trim75_t <- as.data.frame(t(IgAB1_trim75))

rownames(IgAB1_trim75_t) == sample_data(IgPos_forindexB1)$SampleID

## [1] TRUE TRUE TRUE TRUE TRUE TRUE TRUE TRUE TRUE TRUE TRUE TRUE TRUE TRUE TRUE
## [16] TRUE TRUE TRUE TRUE TRUE TRUE TRUE TRUE TRUE TRUE TRUE TRUE TRUE TRUE TRUE
## [31] TRUE TRUE TRUE TRUE TRUE TRUE TRUE TRUE TRUE TRUE TRUE TRUE TRUE TRUE TRUE
## [46] TRUE TRUE TRUE TRUE TRUE TRUE TRUE TRUE

rownames(IgAB1_trim75_t) <- sample_data(IgPos_forindexB1)$ChildID

## Batch 2 ##
Batch2 <- subset_samples(Table_pruned6, Batch=="Batch2")
#rel abund
Ig <- transform_sample_counts(Batch2, function(OTU) OTU/sum(OTU))
#add a pseudocount to transformed samples
otu_table(Ig) <- otu_table(Ig) + 0.0000001
#subset
IgNeg_forindexB2 = subset_samples(Ig, Sort %in% c("IgAneg"))
IgPos_forindexB2 = subset_samples(Ig, Sort %in% c("IgApos"))
#make into dataframes
IgNegOTU <- data.frame(otu_table(IgNeg_forindexB2))
IgPosOTU <- data.frame(otu_table(IgPos_forindexB2))
#now the dataframes should be the same dimensions.
dim(IgNegOTU)

## [1] 3970 85

dim(IgPosOTU)

## [1] 3970 85

#but not the same order. Transpose, then "order".
IgNegOTU <- t(IgNegOTU)
IgNeg_Order <- IgNegOTU[ order(row.names(IgNegOTU)), ]
IgPosOTU <- t(IgPosOTU)
IgPos_Order <- IgPosOTU[ order(row.names(IgPosOTU)), ]
#Log transform and calculate IgA Index.
IgNegLog <- log(IgNeg_Order)
IgPosLog <- log(IgPos_Order)
IgAIndexB2 <- -(IgPosLog - IgNegLog)/(IgPosLog + IgNegLog)

#trim step to remove never-targeted taxa (length-1) or taxa that are zero-targeted in at least "x"% of samples (length*x)
IgA_t <- as.data.frame(t(IgAIndexB2))
IgAB2_trim75 <- IgA_t[rowSums(IgA_t==0) <=(length(IgA_t)*0.75), ] #try more filtered for multiple comparisons
dim(IgAB2_trim75)

## [1] 188 85

#add full taxonomy ID to the IgA Index
IgTAX <- data.frame(tax_table(IgPos_forindexB2))
IgTAX$ASV <- row.names(IgTAX)
IgTAX$combined <- paste(IgTAX$Rank1, IgTAX$Rank2, IgTAX$Rank3, IgTAX$Rank4, IgTAX$Rank5, IgTAX$Rank6, IgTAX$Rank7, IgTAX$ASV, sep="|")

TaxaToKeep75 <- row.names(IgAB2_trim75)
IgTAX75_B2 <- filter(IgTAX, ASV %in% TaxaToKeep75)
row.names(IgAB2_trim75) <- IgTAX75_B2$combined

#will generally want to have it transposed so that you can filter by ID and add metadata
IgAB2_trim75_t <- as.data.frame(t(IgAB2_trim75))

rownames(IgAB2_trim75_t) == sample_data(IgPos_forindexB2)$SampleID

## [1] TRUE TRUE TRUE TRUE TRUE TRUE TRUE TRUE TRUE TRUE TRUE TRUE TRUE TRUE TRUE
## [16] TRUE TRUE TRUE TRUE TRUE TRUE TRUE TRUE TRUE TRUE TRUE TRUE TRUE TRUE TRUE
## [31] TRUE TRUE TRUE TRUE TRUE TRUE TRUE TRUE TRUE TRUE TRUE TRUE TRUE TRUE TRUE
## [46] TRUE TRUE TRUE TRUE TRUE TRUE TRUE TRUE TRUE TRUE TRUE TRUE TRUE TRUE TRUE
## [61] TRUE TRUE TRUE TRUE TRUE TRUE TRUE TRUE TRUE TRUE TRUE TRUE TRUE TRUE TRUE
## [76] TRUE TRUE TRUE TRUE TRUE TRUE TRUE TRUE TRUE TRUE

rownames(IgAB2_trim75_t) <- sample_data(IgPos_forindexB2)$ChildID

##Create IgA Index on the whole data set; filter for taxa that are prevalent in BOTH batches##
#rel abund
Ig <- transform_sample_counts(Table_pruned6, function(OTU) OTU/sum(OTU))
#add a pseudocount to transformed samples
otu_table(Ig) <- otu_table(Ig) + 0.0000001
#subset
IgNeg_forindex = subset_samples(Ig, Sort %in% c("IgAneg"))
IgPos_forindex = subset_samples(Ig, Sort %in% c("IgApos"))
#make into dataframes
IgNegOTU <- data.frame(otu_table(IgNeg_forindex))
IgPosOTU <- data.frame(otu_table(IgPos_forindex))
#now the dataframes should be the same dimensions.
dim(IgNegOTU)

## [1] 3970 138

dim(IgPosOTU)

## [1] 3970 138

#but not the same order. Transpose, then "order".
IgNegOTU <- t(IgNegOTU)
IgNeg_Order <- IgNegOTU[ order(row.names(IgNegOTU)), ]
IgPosOTU <- t(IgPosOTU)
IgPos_Order <- IgPosOTU[ order(row.names(IgPosOTU)), ]

#Log transform and calculate IgA Index.
IgNegLog <- log(IgNeg_Order)
IgPosLog <- log(IgPos_Order)
IgAIndex <- -(IgPosLog - IgNegLog)/(IgPosLog + IgNegLog)

#trim step to remove never-targeted taxa (length-1) or taxa that are zero-targeted in at least "x"% of samples (length*x)
IgA_t <- as.data.frame(t(IgAIndex))
IgA_trim75 <- IgA_t[rowSums(IgA_t==0) <=(length(IgA_t)*0.75), ]
dim(IgA_trim75)

## [1] 197 138

#add full taxonomy ID to the IgA Index
IgTAX <- data.frame(tax_table(IgPos_forindex))
IgTAX$ASV <- row.names(IgTAX)
IgTAX$combined <- paste(IgTAX$Rank1, IgTAX$Rank2, IgTAX$Rank3, IgTAX$Rank4, IgTAX$Rank5, IgTAX$Rank6, IgTAX$Rank7, IgTAX$ASV, sep="|")

TaxaToKeep75 <- row.names(IgA_trim75)
IgTAX75 <- filter(IgTAX, ASV %in% TaxaToKeep75)
row.names(IgA_trim75) <- IgTAX75$combined

#will generally want to have it transposed so that you can filter by ID and add metadata
IgA_trim75_t <- as.data.frame(t(IgA_trim75))

rownames(IgA_trim75_t) == sample_data(IgPos_forindex)$SampleID

## [1] TRUE TRUE TRUE TRUE TRUE TRUE TRUE TRUE TRUE TRUE TRUE TRUE TRUE TRUE TRUE
## [16] TRUE TRUE TRUE TRUE TRUE TRUE TRUE TRUE TRUE TRUE TRUE TRUE TRUE TRUE TRUE
## [31] TRUE TRUE TRUE TRUE TRUE TRUE TRUE TRUE TRUE TRUE TRUE TRUE TRUE TRUE TRUE
## [46] TRUE TRUE TRUE TRUE TRUE TRUE TRUE TRUE TRUE TRUE TRUE TRUE TRUE TRUE TRUE
## [61] TRUE TRUE TRUE TRUE TRUE TRUE TRUE TRUE TRUE TRUE TRUE TRUE TRUE TRUE TRUE
## [76] TRUE TRUE TRUE TRUE TRUE TRUE TRUE TRUE TRUE TRUE TRUE TRUE TRUE TRUE TRUE
## [91] TRUE TRUE TRUE TRUE TRUE TRUE TRUE TRUE TRUE TRUE TRUE TRUE TRUE TRUE TRUE
## [106] TRUE TRUE TRUE TRUE TRUE TRUE TRUE TRUE TRUE TRUE TRUE TRUE TRUE TRUE TRUE
## [121] TRUE TRUE TRUE TRUE TRUE TRUE TRUE TRUE TRUE TRUE TRUE TRUE TRUE TRUE TRUE
## [136] TRUE TRUE TRUE

rownames(IgA_trim75_t) <- sample_data(IgPos_forindex)$ChildID

##Filter for taxa prevalent in both batches, to reduce contaminants/batch effect##
batch1taxa75 <- row.names(IgAB1_trim75)
batch2taxa75 <- row.names(IgAB2_trim75)

IgA_t <- as.data.frame(t(IgAIndex))
IgTAX <- data.frame(tax_table(IgPos_forindex))
IgTAX$ASV <- row.names(IgTAX)
IgTAX$combined <- paste(IgTAX$Rank1, IgTAX$Rank2, IgTAX$Rank3, IgTAX$Rank4, IgTAX$Rank5, IgTAX$Rank6, IgTAX$Rank7, IgTAX$ASV, sep="|")
IgA_t$Taxa <- IgTAX$combined

IgA_batchtrim75 <- filter(IgA_t, Taxa %in% batch1taxa75 & Taxa %in% batch2taxa75)
row.names(IgA_batchtrim75) <- IgA_batchtrim75$Taxa
IgA_batchtrim75$Taxa <- NULL

IgA_batchtrim75_t <- as.data.frame(t(IgA_batchtrim75))
rownames(IgA_batchtrim75_t) == sample_data(IgPos_forindex)$SampleID

## [1] TRUE TRUE TRUE TRUE TRUE TRUE TRUE TRUE TRUE TRUE TRUE TRUE TRUE TRUE TRUE
## [16] TRUE TRUE TRUE TRUE TRUE TRUE TRUE TRUE TRUE TRUE TRUE TRUE TRUE TRUE TRUE
## [31] TRUE TRUE TRUE TRUE TRUE TRUE TRUE TRUE TRUE TRUE TRUE TRUE TRUE TRUE TRUE
## [46] TRUE TRUE TRUE TRUE TRUE TRUE TRUE TRUE TRUE TRUE TRUE TRUE TRUE TRUE TRUE
## [61] TRUE TRUE TRUE TRUE TRUE TRUE TRUE TRUE TRUE TRUE TRUE TRUE TRUE TRUE TRUE
## [76] TRUE TRUE TRUE TRUE TRUE TRUE TRUE TRUE TRUE TRUE TRUE TRUE TRUE TRUE TRUE
## [91] TRUE TRUE TRUE TRUE TRUE TRUE TRUE TRUE TRUE TRUE TRUE TRUE TRUE TRUE TRUE
## [106] TRUE TRUE TRUE TRUE TRUE TRUE TRUE TRUE TRUE TRUE TRUE TRUE TRUE TRUE TRUE
## [121] TRUE TRUE TRUE TRUE TRUE TRUE TRUE TRUE TRUE TRUE TRUE TRUE TRUE TRUE TRUE
## [136] TRUE TRUE TRUE

rownames(IgA_batchtrim75_t) <- sample_data(IgPos_forindex)$ChildID

dim(IgA_batchtrim75_t)

## [1] 138 140

# Create the IgA Index and filter by taxa prevalence within each batch (Genus level)

Table6_genus <- tax_glom(Table_pruned6, "Rank6")
#Batch 1 genus
Batch1_genus <- subset_samples(Table6_genus, Batch=="Batch1")
#rel abund
Ig <- transform_sample_counts(Batch1_genus, function(OTU) OTU/sum(OTU))
#add a pseudocount to transformed samples
otu_table(Ig) <- otu_table(Ig) + 0.0000001
#subset
IgNeg_forindexB1_genus = subset_samples(Ig, Sort %in% c("IgAneg"))
IgPos_forindexB1_genus = subset_samples(Ig, Sort %in% c("IgApos"))
#make into dataframes
IgNegOTU_genus <- data.frame(otu_table(IgNeg_forindexB1_genus))
IgPosOTU_genus <- data.frame(otu_table(IgPos_forindexB1_genus))
#now the dataframes should be the same dimensions.
dim(IgNegOTU_genus)

## [1] 468 53

dim(IgPosOTU_genus)

## [1] 468 53

#but not the same order. Transpose, then "order".
IgNegOTU_genus <- t(IgNegOTU_genus)
IgNeg_Order_genus <- IgNegOTU_genus[ order(row.names(IgNegOTU_genus)), ]
IgPosOTU_genus <- t(IgPosOTU_genus)
IgPos_Order_genus <- IgPosOTU_genus[ order(row.names(IgPosOTU_genus)), ]
#Log transform and calculate IgA Index.
IgNegLog_genus <- log(IgNeg_Order_genus)
IgPosLog_genus <- log(IgPos_Order_genus)
IgAIndexB1_genus <- -(IgPosLog_genus - IgNegLog_genus)/(IgPosLog_genus + IgNegLog_genus)

#trim step to remove never-targeted taxa (length-1) or taxa that are zero-targeted in at least "x"% of samples (length*x)
IgA_t_genus <- as.data.frame(t(IgAIndexB1_genus))
IgAB1_trim75_genus <- IgA_t_genus[rowSums(IgA_t_genus==0) <=(length(IgA_t_genus)*0.75), ]
dim(IgA_t_genus)

## [1] 468 53

dim(IgAB1_trim75_genus)

## [1] 109 53

#add full taxonomy ID to the IgA Index
IgTAX_genus <- as.data.frame(IgPos_forindexB1_genus@tax_table@.Data)
IgTAX_genus$ASV <- row.names(IgTAX_genus)
IgTAX_genus$combined <- paste(IgTAX_genus$Rank1, IgTAX_genus$Rank2, IgTAX_genus$Rank3,
 IgTAX_genus$Rank4, IgTAX_genus$Rank5, IgTAX_genus$Rank6,
 sep="|")

TaxaToKeep75_genus <- row.names(IgAB1_trim75_genus)
IgTAX75_B1_genus <- filter(IgTAX_genus, ASV %in% TaxaToKeep75_genus)
row.names(IgAB1_trim75_genus) <- IgTAX75_B1_genus$combined

#will generally want to have it transposed so that you can filter by ID and add metadata
IgAB1_trim75_genus_t <- as.data.frame(t(IgAB1_trim75_genus))
rownames(IgAB1_trim75_genus_t) <- sample_data(IgPos_forindexB1_genus)$ChildID

#batch2 genus
Batch2_genus <- subset_samples(Table6_genus, Batch=="Batch2")
#rel abund
Ig <- transform_sample_counts(Batch2_genus, function(OTU) OTU/sum(OTU))
#add a pseudocount to transformed samples
otu_table(Ig) <- otu_table(Ig) + 0.0000001
#subset
IgNeg_forindexB2_genus = subset_samples(Ig, Sort %in% c("IgAneg"))
IgPos_forindexB2_genus = subset_samples(Ig, Sort %in% c("IgApos"))
#make into dataframes
IgNegOTU_genus <- data.frame(otu_table(IgNeg_forindexB2_genus))
IgPosOTU_genus <- data.frame(otu_table(IgPos_forindexB2_genus))
#now the dataframes should be the same dimensions.
dim(IgNegOTU_genus)

## [1] 468 85

dim(IgPosOTU_genus)

## [1] 468 85

#but they don't seem to be in the same order. Transpose, then "order".
IgNegOTU_genus <- t(IgNegOTU_genus)
IgNeg_Order_genus <- IgNegOTU_genus[ order(row.names(IgNegOTU_genus)), ]
IgPosOTU_genus <- t(IgPosOTU_genus)
IgPos_Order_genus <- IgPosOTU_genus[ order(row.names(IgPosOTU_genus)), ]
#Log transform and calculate IgA Index.
IgNegLog_genus <- log(IgNeg_Order_genus)
IgPosLog_genus <- log(IgPos_Order_genus)
IgAIndexB2_genus <- -(IgPosLog_genus - IgNegLog_genus)/(IgPosLog_genus + IgNegLog_genus)

#trim step to remove never-targeted taxa (length-1) or taxa that are zero-targeted in at least "x"% of samples (length*x)
IgA_t_genus <- as.data.frame(t(IgAIndexB2_genus))
IgAB2_trim75_genus <- IgA_t_genus[rowSums(IgA_t_genus==0) <=(length(IgA_t_genus)*0.75), ]
dim(IgA_t_genus)

## [1] 468 85

dim(IgAB2_trim75_genus)

## [1] 124 85

#add full taxonomy ID to the IgA Index
IgTAX_genus <- as.data.frame(IgPos_forindexB2_genus@tax_table@.Data)
IgTAX_genus$ASV <- row.names(IgTAX_genus)
IgTAX_genus$combined <- paste(IgTAX_genus$Rank1, IgTAX_genus$Rank2, IgTAX_genus$Rank3,
 IgTAX_genus$Rank4, IgTAX_genus$Rank5, IgTAX_genus$Rank6,
 sep="|")

TaxaToKeep75_genus <- row.names(IgAB2_trim75_genus)
IgTAX75_B2_genus <- filter(IgTAX_genus, ASV %in% TaxaToKeep75_genus)
row.names(IgAB2_trim75_genus) <- IgTAX75_B2_genus$combined

#will generally want to have it transposed so that you can filter by ID and add metadata
IgAB2_trim75_genus_t <- as.data.frame(t(IgAB2_trim75_genus))
rownames(IgAB2_trim75_genus_t) <- sample_data(IgPos_forindexB2_genus)$ChildID

#full dataset genus
#rel abund
Ig <- transform_sample_counts(Table6_genus, function(OTU) OTU/sum(OTU))
#add a pseudocount to transformed samples
otu_table(Ig) <- otu_table(Ig) + 0.0000001
#subset
IgNeg_forindexGen = subset_samples(Ig, Sort %in% c("IgAneg"))
IgPos_forindexGen = subset_samples(Ig, Sort %in% c("IgApos"))
#make into dataframes
IgNegOTU <- data.frame(otu_table(IgNeg_forindexGen))
IgPosOTU <- data.frame(otu_table(IgPos_forindexGen))
#now the dataframes should be the same dimensions.
dim(IgNegOTU)

## [1] 468 138

dim(IgPosOTU)

## [1] 468 138

#but not the same order. Transpose, then "order".
IgNegOTU <- t(IgNegOTU)
IgNeg_Order <- IgNegOTU[ order(row.names(IgNegOTU)), ]
IgPosOTU <- t(IgPosOTU)
IgPos_Order <- IgPosOTU[ order(row.names(IgPosOTU)), ]
#Log transform and calculate IgA Index.
IgNegLog <- log(IgNeg_Order)
IgPosLog <- log(IgPos_Order)
IgAIndex_genus <- -(IgPosLog - IgNegLog)/(IgPosLog + IgNegLog)

#trim step to remove never-targeted taxa (length-1) or taxa that are zero-targeted in at least "x"% of samples (length*x)
IgA_t <- as.data.frame(t(IgAIndex_genus))
IgA_genus_trim75 <- IgA_t[rowSums(IgA_t==0) <=(length(IgA_t)*0.75), ]
dim(IgAIndex_genus)

## [1] 138 468

dim(IgA_genus_trim75)

## [1] 126 138

#add full taxonomy ID to the IgA Index
IgTAX <- as.data.frame(IgPos_forindexGen@tax_table@.Data)
IgTAX$ASV <- row.names(IgTAX)
IgTAX$combined <- paste(IgTAX$Rank1, IgTAX$Rank2, IgTAX$Rank3, IgTAX$Rank4, IgTAX$Rank5, IgTAX$Rank6, sep="|")

TaxaToKeep75 <- row.names(IgA_genus_trim75)
IgTAX75_genus <- filter(IgTAX, ASV %in% TaxaToKeep75)
row.names(IgA_genus_trim75) <- IgTAX75_genus$combined

#will generally want to have it transposed so that you can filter by ID and add metadata
IgA_genus_trim75_t <- as.data.frame(t(IgA_genus_trim75))

row.names(IgA_genus_trim75_t) <- sample_data(IgPos_forindexGen)$ChildID

##Filter for taxa prevalent in both batches, to reduce contaminants/batch effect##
batch1taxa75_genus <- row.names(IgAB1_trim75_genus)
batch2taxa75_genus <- row.names(IgAB2_trim75_genus)

IgA_t_genus <- as.data.frame(t(IgAIndex_genus))
IgTAX_genus <- as.data.frame(IgPos_forindexGen@tax_table@.Data)
IgTAX_genus$ASV <- row.names(IgTAX_genus)
IgTAX_genus$combined <- paste(IgTAX_genus$Rank1, IgTAX_genus$Rank2, IgTAX_genus$Rank3,
 IgTAX_genus$Rank4, IgTAX_genus$Rank5, IgTAX_genus$Rank6,
 sep="|")
IgA_t_genus$Taxa <- IgTAX_genus$combined #to use the full taxonomy
#IgA_t_genus$Taxa <- IgTAX_genus$Rank6 #to use genus only

IgA_batchtrim75_genus <- filter(IgA_t_genus, Taxa %in% batch1taxa75_genus & Taxa %in% batch2taxa75_genus) #also 169 taxa remaining
row.names(IgA_batchtrim75_genus) <- IgA_batchtrim75_genus$Taxa
IgA_batchtrim75_genus$Taxa <- NULL
dim(IgA_batchtrim75_genus)

## [1] 96 138

IgA_batchtrim75_genus_t <- as.data.frame(t(IgA_batchtrim75_genus))
rownames(IgA_batchtrim75_genus_t) <- sample_data(IgPos_forindexGen)$ChildID

dim(IgA_batchtrim75_genus_t)

## [1] 138 96

# Tables 1, S1, S2: Metadata counts on final filtered IgA-SEQ dataset

metaIgA <- as.data.frame(sample_data(IgPos_forindex))
dim(metaIgA)

## [1] 138 396

dplyr::count(metaIgA, Country)

## # A tibble: 2 x 2
## Country n
## <chr> <int>
## 1 Mada 78
## 2 RCA 60

dplyr::count(metaIgA, sexe)

## # A tibble: 2 x 2
## sexe n
## <chr> <int>
## 1 FÃ©minin 74
## 2 Masculin 64

dplyr::count(metaIgA, Batch)

## # A tibble: 2 x 2
## Batch n
## <chr> <int>
## 1 Batch1 53
## 2 Batch2 85

#general distribution
median(metaIgA$age)

## [1] 40.53654

dplyr::count(metaIgA, age_years_categ)

## # A tibble: 4 x 2
## age_years_categ n
## <chr> <int>
## 1 Four 48
## 2 Three 35
## 3 Two 54
## 4 <NA> 1

dplyr::count(metaIgA, stunted)

## # A tibble: 2 x 2
## stunted n
## <int> <int>
## 1 0 67
## 2 1 71

dplyr::count(metaIgA, haz)

## # A tibble: 3 x 2
## haz n
## <int> <int>
## 1 0 67
## 2 1 9
## 3 2 62

# stunting and country
dplyr::count(metaIgA, stunted, Country)

## # A tibble: 4 x 3
## stunted Country n
## <int> <chr> <int>
## 1 0 Mada 35
## 2 0 RCA 32
## 3 1 Mada 43
## 4 1 RCA 28

dplyr::count(metaIgA, stunted, Batch)

## # A tibble: 4 x 3
## stunted Batch n
## <int> <chr> <int>
## 1 0 Batch1 26
## 2 0 Batch2 41
## 3 1 Batch1 27
## 4 1 Batch2 44

dplyr::count(metaIgA, Batch, Country)

## # A tibble: 4 x 3
## Batch Country n
## <chr> <chr> <int>
## 1 Batch1 Mada 40
## 2 Batch1 RCA 13
## 3 Batch2 Mada 38
## 4 Batch2 RCA 47

fisher.test(as.factor(metaIgA$stunted), metaIgA$Country)

##
## Fisher's Exact Test for Count Data
##
## data: as.factor(metaIgA$stunted) and metaIgA$Country
## p-value = 0.3909
## alternative hypothesis: true odds ratio is not equal to 1
## 95 percent confidence interval:
## 0.3428883 1.4776043
## sample estimates:
## odds ratio
## 0.7139859

fisher.test(as.factor(metaIgA$stunted), metaIgA$Batch)

##
## Fisher's Exact Test for Count Data
##
## data: as.factor(metaIgA$stunted) and metaIgA$Batch
## p-value = 1
## alternative hypothesis: true odds ratio is not equal to 1
## 95 percent confidence interval:
## 0.491278 2.172132
## sample estimates:
## odds ratio
## 1.033192

fisher.test(as.factor(metaIgA$Batch), metaIgA$Country)

##
## Fisher's Exact Test for Count Data
##
## data: as.factor(metaIgA$Batch) and metaIgA$Country
## p-value = 0.00042
## alternative hypothesis: true odds ratio is not equal to 1
## 95 percent confidence interval:
## 1.686734 8.841089
## sample estimates:
## odds ratio
## 3.768074

#sexe by stunting, country
dplyr::count(metaIgA, stunted, sexe)

## # A tibble: 4 x 3
## stunted sexe n
## <int> <chr> <int>
## 1 0 FÃ©minin 38
## 2 0 Masculin 29
## 3 1 FÃ©minin 36
## 4 1 Masculin 35

dplyr::count(metaIgA, Country, sexe)

## # A tibble: 4 x 3
## Country sexe n
## <chr> <chr> <int>
## 1 Mada FÃ©minin 41
## 2 Mada Masculin 37
## 3 RCA FÃ©minin 33
## 4 RCA Masculin 27

fisher.test(as.factor(metaIgA$stunted), as.factor(metaIgA$sexe))

##
## Fisher's Exact Test for Count Data
##
## data: as.factor(metaIgA$stunted) and as.factor(metaIgA$sexe)
## p-value = 0.4993
## alternative hypothesis: true odds ratio is not equal to 1
## 95 percent confidence interval:
## 0.6174726 2.6319443
## sample estimates:
## odds ratio
## 1.271696

fisher.test(metaIgA$Country, as.factor(metaIgA$sexe))

##
## Fisher's Exact Test for Count Data
##
## data: metaIgA$Country and as.factor(metaIgA$sexe)
## p-value = 0.8636
## alternative hypothesis: true odds ratio is not equal to 1
## 95 percent confidence interval:
## 0.4363415 1.8800898
## sample estimates:
## odds ratio
## 0.9072788

#age by stunting, country
median(metaIgA[metaIgA$stunted==0]$age)

## [1] 42.70638

median(metaIgA[metaIgA$stunted==1]$age)

## [1] 38.43246

dplyr::count(metaIgA, stunted, age_years_categ)

## # A tibble: 7 x 3
## stunted age_years_categ n
## <int> <chr> <int>
## 1 0 Four 25
## 2 0 Three 18
## 3 0 Two 24
## 4 1 Four 23
## 5 1 Three 17
## 6 1 Two 30
## 7 1 <NA> 1

fisher.test(metaIgA$stunted, metaIgA$age_years_categ)

##
## Fisher's Exact Test for Count Data
##
## data: metaIgA$stunted and metaIgA$age_years_categ
## p-value = 0.7458
## alternative hypothesis: two.sided

wilcox.test(metaIgA$age ~ as.factor(metaIgA$stunted))

##
## Wilcoxon rank sum test with continuity correction
##
## data: metaIgA$age by as.factor(metaIgA$stunted)
## W = 2596, p-value = 0.3553
## alternative hypothesis: true location shift is not equal to 0

median(metaIgA[metaIgA$Country=="Mada"]$age)

## [1] 42.59131

median(metaIgA[metaIgA$Country=="RCA"]$age)

## [1] 37.97219

dplyr::count(metaIgA, Country, age_years_categ)

## # A tibble: 7 x 3
## Country age_years_categ n
## <chr> <chr> <int>
## 1 Mada Four 27
## 2 Mada Three 23
## 3 Mada Two 28
## 4 RCA Four 21
## 5 RCA Three 12
## 6 RCA Two 26
## 7 RCA <NA> 1

fisher.test(metaIgA$Country, metaIgA$age_years_categ)

##
## Fisher's Exact Test for Count Data
##
## data: metaIgA$Country and metaIgA$age_years_categ
## p-value = 0.4402
## alternative hypothesis: two.sided

wilcox.test(metaIgA$age ~ as.factor(metaIgA$Country))

##
## Wilcoxon rank sum test with continuity correction
##
## data: metaIgA$age by as.factor(metaIgA$Country)
## W = 2437.5, p-value = 0.677
## alternative hypothesis: true location shift is not equal to 0

#HAZ by stunting, country
median(metaIgA[metaIgA$stunted==0]$haz_cont)

## [1] -1.05

median(metaIgA[metaIgA$stunted==1]$haz_cont)

## [1] -3.48

wilcox.test(metaIgA$haz_cont ~ as.factor(metaIgA$stunted))

##
## Wilcoxon rank sum test with continuity correction
##
## data: metaIgA$haz_cont by as.factor(metaIgA$stunted)
## W = 4757, p-value < 2.2e-16
## alternative hypothesis: true location shift is not equal to 0

median(metaIgA[metaIgA$Country=="Mada"]$haz_cont)

## [1] -2.125

median(metaIgA[metaIgA$Country=="RCA"]$haz_cont)

## [1] -1.9

wilcox.test(metaIgA$haz_cont ~ as.factor(metaIgA$Country))

##
## Wilcoxon rank sum test with continuity correction
##
## data: metaIgA$haz_cont by as.factor(metaIgA$Country)
## W = 2261, p-value = 0.736
## alternative hypothesis: true location shift is not equal to 0

#WHZ by stunting, country, batch
median(metaIgA[metaIgA$stunted==0]$whz_cont)

## [1] -0.21

median(metaIgA[metaIgA$stunted==1]$whz_cont)

## [1] -0.54

wilcox.test(metaIgA$whz_cont ~ as.factor(metaIgA$stunted))

##
## Wilcoxon rank sum test with continuity correction
##
## data: metaIgA$whz_cont by as.factor(metaIgA$stunted)
## W = 2820.5, p-value = 0.05998
## alternative hypothesis: true location shift is not equal to 0

median(metaIgA[metaIgA$Country=="Mada"]$whz_cont)

## [1] -0.4

median(metaIgA[metaIgA$Country=="RCA"]$whz_cont)

## [1] -0.505

wilcox.test(metaIgA$whz_cont ~ as.factor(metaIgA$Country))

##
## Wilcoxon rank sum test with continuity correction
##
## data: metaIgA$whz_cont by as.factor(metaIgA$Country)
## W = 2374.5, p-value = 0.8839
## alternative hypothesis: true location shift is not equal to 0

#Breastfeeding duration by stunting, country
median(metaIgA[metaIgA$stunted==0]$age_allaite, na.rm = TRUE)

## [1] 20

median(metaIgA[metaIgA$stunted==1]$age_allaite, na.rm = TRUE)

## [1] 20

wilcox.test(metaIgA$age_allaite ~ as.factor(metaIgA$stunted))

##
## Wilcoxon rank sum test with continuity correction
##
## data: metaIgA$age_allaite by as.factor(metaIgA$stunted)
## W = 2211, p-value = 0.8842
## alternative hypothesis: true location shift is not equal to 0

median(metaIgA[metaIgA$Country=="Mada"]$age_allaite, na.rm = TRUE)

## [1] 24

median(metaIgA[metaIgA$Country=="RCA"]$age_allaite, na.rm = TRUE)

## [1] 18

wilcox.test(metaIgA$age_allaite ~ metaIgA$Country)

##
## Wilcoxon rank sum test with continuity correction
##
## data: metaIgA$age_allaite by metaIgA$Country
## W = 3295.5, p-value = 1.27e-06
## alternative hypothesis: true location shift is not equal to 0

median(metaIgA[metaIgA$Batch=="Batch1"]$age_allaite, na.rm = TRUE)

## [1] 24

median(metaIgA[metaIgA$Batch=="Batch2"]$age_allaite, na.rm = TRUE)

## [1] 18

wilcox.test(metaIgA$age_allaite ~ metaIgA$Batch)

##
## Wilcoxon rank sum test with continuity correction
##
## data: metaIgA$age_allaite by metaIgA$Batch
## W = 2578.5, p-value = 0.04027
## alternative hypothesis: true location shift is not equal to 0

#Inflammatory markers by stunting
median(metaIgA[metaIgA$stunted==0]$AATmggdePS, na.rm = TRUE)

## [1] 42

median(metaIgA[metaIgA$stunted==1]$AATmggdePS, na.rm = TRUE)

## [1] 43.5

wilcox.test(metaIgA$AATmggdePS ~ as.factor(metaIgA$stunted))

##
## Wilcoxon rank sum test with continuity correction
##
## data: metaIgA$AATmggdePS by as.factor(metaIgA$stunted)
## W = 1898, p-value = 0.4979
## alternative hypothesis: true location shift is not equal to 0

median(metaIgA[metaIgA$stunted==0]$CALPROTECTINEggdePS, na.rm = TRUE)

## [1] 367.5

median(metaIgA[metaIgA$stunted==1]$CALPROTECTINEggdePS, na.rm = TRUE)

## [1] 405

wilcox.test(metaIgA$CALPROTECTINEggdePS ~ as.factor(metaIgA$stunted))

##
## Wilcoxon rank sum test with continuity correction
##
## data: metaIgA$CALPROTECTINEggdePS by as.factor(metaIgA$stunted)
## W = 2029.5, p-value = 0.7026
## alternative hypothesis: true location shift is not equal to 0

dplyr::count(metaIgA, stunted, crp_seuil)

## # A tibble: 6 x 3
## stunted crp_seuil n
## <int> <chr> <int>
## 1 0 CRP haute (>10 mg/l) 4
## 2 0 CRP normale (<=10 mg/l) 57
## 3 0 <NA> 6
## 4 1 CRP haute (>10 mg/l) 13
## 5 1 CRP normale (<=10 mg/l) 53
## 6 1 <NA> 5

fisher.test(metaIgA$stunted, metaIgA$crp_seuil)

##
## Fisher's Exact Test for Count Data
##
## data: metaIgA$stunted and metaIgA$crp_seuil
## p-value = 0.03737
## alternative hypothesis: true odds ratio is not equal to 1
## 95 percent confidence interval:
## 0.06453882 1.01106678
## sample estimates:
## odds ratio
## 0.2887595

#Inflammatory markers by country
median(metaIgA[metaIgA$Country=="Mada"]$AATmggdePS, na.rm = TRUE)

## [1] 49.5

median(metaIgA[metaIgA$Country=="RCA"]$AATmggdePS, na.rm = TRUE)

## [1] 37

wilcox.test(metaIgA$AATmggdePS ~ metaIgA$Country)

##
## Wilcoxon rank sum test with continuity correction
##
## data: metaIgA$AATmggdePS by metaIgA$Country
## W = 2059.5, p-value = 0.08091
## alternative hypothesis: true location shift is not equal to 0

median(metaIgA[metaIgA$Country=="Mada"]$CALPROTECTINEggdePS, na.rm = TRUE)

## [1] 502

median(metaIgA[metaIgA$Country=="RCA"]$CALPROTECTINEggdePS, na.rm = TRUE)

## [1] 296

wilcox.test(metaIgA$CALPROTECTINEggdePS ~ metaIgA$Country)

##
## Wilcoxon rank sum test with continuity correction
##
## data: metaIgA$CALPROTECTINEggdePS by metaIgA$Country
## W = 2740, p-value = 0.0009272
## alternative hypothesis: true location shift is not equal to 0

dplyr::count(metaIgA, Country, crp_seuil)

## # A tibble: 6 x 3
## Country crp_seuil n
## <chr> <chr> <int>
## 1 Mada CRP haute (>10 mg/l) 7
## 2 Mada CRP normale (<=10 mg/l) 70
## 3 Mada <NA> 1
## 4 RCA CRP haute (>10 mg/l) 10
## 5 RCA CRP normale (<=10 mg/l) 40
## 6 RCA <NA> 10

fisher.test(metaIgA$Country, metaIgA$crp_seuil)

##
## Fisher's Exact Test for Count Data
##
## data: metaIgA$Country and metaIgA$crp_seuil
## p-value = 0.1088
## alternative hypothesis: true odds ratio is not equal to 1
## 95 percent confidence interval:
## 0.1200282 1.2782455
## sample estimates:
## odds ratio
## 0.4030471

#iron status by stunting
median(metaIgA[metaIgA$stunted==0]$hemoglobine2, na.rm = TRUE)

## [1] 11.6

median(metaIgA[metaIgA$stunted==1]$hemoglobine2, na.rm = TRUE)

## [1] 10.9

wilcox.test(metaIgA$hemoglobine2 ~ as.factor(metaIgA$stunted))

##
## Wilcoxon rank sum test with continuity correction
##
## data: metaIgA$hemoglobine2 by as.factor(metaIgA$stunted)
## W = 2729.5, p-value = 0.004043
## alternative hypothesis: true location shift is not equal to 0

dplyr::count(metaIgA, stunted, anemie2)

## # A tibble: 6 x 3
## stunted anemie2 n
## <int> <int> <int>
## 1 0 0 46
## 2 0 1 18
## 3 0 NA 3
## 4 1 0 32
## 5 1 1 34
## 6 1 NA 5

fisher.test(metaIgA$stunted, metaIgA$anemie2)

##
## Fisher's Exact Test for Count Data
##
## data: metaIgA$stunted and metaIgA$anemie2
## p-value = 0.007559
## alternative hypothesis: true odds ratio is not equal to 1
## 95 percent confidence interval:
## 1.236296 6.022368
## sample estimates:
## odds ratio
## 2.69378

#iron status by country
median(metaIgA[metaIgA$Country=="Mada"]$hemoglobine2, na.rm = TRUE)

## [1] 11.5

median(metaIgA[metaIgA$Country=="RCA"]$hemoglobine2, na.rm = TRUE)

## [1] 10.7

wilcox.test(metaIgA$hemoglobine2 ~ as.factor(metaIgA$Country))

##
## Wilcoxon rank sum test with continuity correction
##
## data: metaIgA$hemoglobine2 by as.factor(metaIgA$Country)
## W = 2715, p-value = 0.001399
## alternative hypothesis: true location shift is not equal to 0

dplyr::count(metaIgA, Country, anemie2)

## # A tibble: 6 x 3
## Country anemie2 n
## <chr> <int> <int>
## 1 Mada 0 55
## 2 Mada 1 22
## 3 Mada NA 1
## 4 RCA 0 23
## 5 RCA 1 30
## 6 RCA NA 7

fisher.test(metaIgA$Country, metaIgA$anemie2)

##
## Fisher's Exact Test for Count Data
##
## data: metaIgA$Country and metaIgA$anemie2
## p-value = 0.001887
## alternative hypothesis: true odds ratio is not equal to 1
## 95 percent confidence interval:
## 1.469578 7.260397
## sample estimates:
## odds ratio
## 3.229083

#helminths & parasites by stunting
dplyr::count(metaIgA, stunted, helminth)

## # A tibble: 6 x 3
## stunted helminth n
## <int> <int> <int>
## 1 0 0 25
## 2 0 1 34
## 3 0 NA 8
## 4 1 0 30
## 5 1 1 36
## 6 1 NA 5

fisher.test(metaIgA$stunted, metaIgA$helminth)

##
## Fisher's Exact Test for Count Data
##
## data: metaIgA$stunted and metaIgA$helminth
## p-value = 0.8569
## alternative hypothesis: true odds ratio is not equal to 1
## 95 percent confidence interval:
## 0.4088954 1.9003944
## sample estimates:
## odds ratio
## 0.8832386

dplyr::count(metaIgA, stunted, giardiase)

## # A tibble: 6 x 3
## stunted giardiase n
## <int> <chr> <int>
## 1 0 Non 48
## 2 0 Oui 11
## 3 0 <NA> 8
## 4 1 Non 49
## 5 1 Oui 17
## 6 1 <NA> 5

fisher.test(metaIgA$stunted, metaIgA$giardiase)

##
## Fisher's Exact Test for Count Data
##
## data: metaIgA$stunted and metaIgA$giardiase
## p-value = 0.3942
## alternative hypothesis: true odds ratio is not equal to 1
## 95 percent confidence interval:
## 0.5947434 3.9653767
## sample estimates:
## odds ratio
## 1.508914

#helminths & parasites by Country
dplyr::count(metaIgA, Country, helminth)

## # A tibble: 5 x 3
## Country helminth n
## <chr> <int> <int>
## 1 Mada 0 9
## 2 Mada 1 69
## 3 RCA 0 46
## 4 RCA 1 1
## 5 RCA NA 13

fisher.test(metaIgA$Country, metaIgA$Country)

##
## Fisher's Exact Test for Count Data
##
## data: metaIgA$Country and metaIgA$Country
## p-value < 2.2e-16
## alternative hypothesis: true odds ratio is not equal to 1
## 95 percent confidence interval:
## 627.6341 Inf
## sample estimates:
## odds ratio
## Inf

dplyr::count(metaIgA, Country, giardiase)

## # A tibble: 5 x 3
## Country giardiase n
## <chr> <chr> <int>
## 1 Mada Non 59
## 2 Mada Oui 19
## 3 RCA Non 38
## 4 RCA Oui 9
## 5 RCA <NA> 13

fisher.test(metaIgA$Country, metaIgA$giardiase)

##
## Fisher's Exact Test for Count Data
##
## data: metaIgA$Country and metaIgA$giardiase
## p-value = 0.6584
## alternative hypothesis: true odds ratio is not equal to 1
## 95 percent confidence interval:
## 0.2646958 1.9277124
## sample estimates:
## odds ratio
## 0.7372305

# Fig S5: Main IgA-targeted taxa (ASV level)

## Defining main IgA-targeted taxa, full dataset (ASV level)

#select data table and metadata, filter for category as necessary

IgA_simplewilcox <- IgA_batchtrim75_t
meta_wilcox <- as.data.frame(sample_data(IgPos_forindex))
row.names(IgA_simplewilcox) <- meta_wilcox$ChildID

##ALL - full dataset##
#apply wilcox test to test if Index is sig. diff. from 0
wilcox.data1 <- sapply(1:ncol(IgA_simplewilcox), function(i){wilcox.test(IgA_simplewilcox[,i], mu=0)$p.value})
wilcox.data1 <- as.data.frame(wilcox.data1)
rownames(wilcox.data1) <- colnames(IgA_simplewilcox)
#Perform multiple comparison correction using FDR
wilcox.data1$rel.fdr <- p.adjust(wilcox.data1$wilcox.data1, method="fdr")
#incorporate other metrics of data centre and dispersion
wilcox.data1$mean <- t(summarise_each(IgA_simplewilcox,funs(mean)))
wilcox.data1$median <- t(summarise_each(IgA_simplewilcox,funs(median)))
wilcox.data1$magnitude <- abs(wilcox.data1$mean)
wilcox.data1$variance <- t(summarise_each(IgA_simplewilcox,funs(var)))
wilcox.data1$IQR <- t(summarise_each(IgA_simplewilcox,funs(IQR))) #interquartile range
wilcox.data1$Taxa <- row.names(wilcox.data1)
wilcox.data1$SampleType <- c("All")

#Selecting the most targeted taxa; FDR<0.05, median index >0
most_targeted_all <- dplyr::filter(wilcox.data1, rel.fdr<0.05&median>0)
most_targeted_all$Taxa #5

## [1] "D_0__Bacteria|D_1__Proteobacteria|D_2__Gammaproteobacteria|D_3__Pasteurellales|D_4__Pasteurellaceae|D_5__Haemophilus|NA|e27680d4009f98f30248d823bc17fb8e"
## [2] "D_0__Bacteria|D_1__Firmicutes|D_2__Clostridia|D_3__Clostridiales|D_4__Ruminococcaceae|D_5__Subdoligranulum|NA|a1a300c872047a7d8b6b294b17cfb63b"
## [3] "D_0__Bacteria|D_1__Firmicutes|D_2__Clostridia|D_3__Clostridiales|D_4__Ruminococcaceae|D_5__Subdoligranulum|NA|a180485ac456840a8e7a0014a4bcd598"
## [4] "D_0__Bacteria|D_1__Firmicutes|D_2__Clostridia|D_3__Clostridiales|D_4__Lachnospiraceae|D_5__Dorea|NA|afd87e82de329a1ed75b98b5b606843c"
## [5] "D_0__Bacteria|D_1__Firmicutes|D_2__Clostridia|D_3__Clostridiales|D_4__Lachnospiraceae|D_5__Coprococcus 3|NA|a0b7d83fb64749f9a4b15b3728425b97"

#plot most targeted taxa - all
#collect IgA Index and taxa data into a data frame
targeted_index <- IgA_simplewilcox[,which(names(IgA_simplewilcox) %in% most_targeted_all$Taxa)]
targeted_taxa <- IgTAX75[which(IgTAX75$combined %in% most_targeted_all$Taxa),]
targeted_taxa$Simple <- paste(targeted_taxa$Rank6, substr(targeted_taxa$ASV, start=0, stop=3))
targeted_taxa$Simple <- gsub("D_5__", "", targeted_taxa$Simple)
targeted_taxa$Order <- gsub("D_3__", "", targeted_taxa$Rank4)
targeted_taxa$Phylum <- gsub("D_1__", "", targeted_taxa$Rank2)

pdata1 <- data.frame(IgAIndex=targeted_index[,1], Taxa=targeted_taxa$Simple[1], Phylum=targeted_taxa$Phylum[1])
pdata2 <- data.frame(IgAIndex=targeted_index[,2], Taxa=targeted_taxa$Simple[2], Phylum=targeted_taxa$Phylum[2])
pdata3 <- data.frame(IgAIndex=targeted_index[,3], Taxa=targeted_taxa$Simple[3], Phylum=targeted_taxa$Phylum[3])
pdata4 <- data.frame(IgAIndex=targeted_index[,4], Taxa=targeted_taxa$Simple[4], Phylum=targeted_taxa$Phylum[4])
pdata5 <- data.frame(IgAIndex=targeted_index[,5], Taxa=targeted_taxa$Simple[5], Phylum=targeted_taxa$Phylum[5])

pdata <- Reduce(full_join, list(pdata1, pdata2, pdata3, pdata4, pdata5))

## Joining, by = c("IgAIndex", "Taxa", "Phylum")
## Joining, by = c("IgAIndex", "Taxa", "Phylum")
## Joining, by = c("IgAIndex", "Taxa", "Phylum")
## Joining, by = c("IgAIndex", "Taxa", "Phylum")

#plot - most targeted all
p <- ggplot(pdata) + geom_jitter(aes(x=Taxa, y=IgAIndex, color=Phylum),
 width=0.2, height=0, shape=1, size=3)
p <- p + geom_boxplot(aes(x=Taxa, y=IgAIndex), color="black", fill=NA, outlier.shape=NA)
p <- p + ggtitle("Most Targeted ASV Overall")
p <- p + theme_bw(base_size=16)
p <- p + theme(axis.text.x = element_text(angle=45, hjust=1))
p <- p + ylim(c(-1, 1))
p <- p + scale_color_manual(values=c("deepskyblue3", "indianred3"))
p


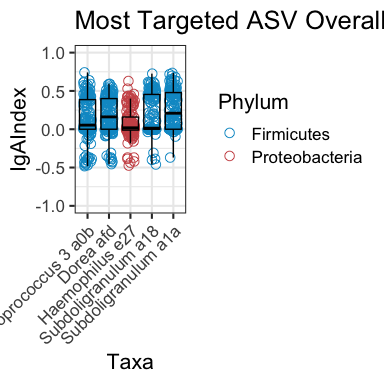


#Selecting the least targeted taxa; FDR<0.05, median index <0
least_targeted_all <- dplyr::filter(wilcox.data1, rel.fdr<0.05&median<0)
least_targeted_all$Taxa

## [1] "D_0__Bacteria|D_1__Firmicutes|D_2__Bacilli|D_3__Lactobacillales|D_4__Streptococcaceae|D_5__Streptococcus|NA|bd2ebc70501f7d867c204f94c4e483da"
## [2] "D_0__Bacteria|D_1__Firmicutes|D_2__Negativicutes|D_3__Selenomonadales|D_4__Veillonellaceae|D_5__Dialister|NA|d6960828e54e3b0059ec5cdf24b16d2f"
## [3] "D_0__Bacteria|D_1__Proteobacteria|D_2__Gammaproteobacteria|D_3__Pseudomonadales|D_4__Pseudomonadaceae|D_5__Pseudomonas|NA|402e5913597695a16d7cad415ffff02f"
## [4] "D_0__Bacteria|D_1__Proteobacteria|D_2__Gammaproteobacteria|D_3__Pseudomonadales|D_4__Pseudomonadaceae|D_5__Pseudomonas|NA|1f878f615fcfc8d7bd381a7841ac1e41"
## [5] "D_0__Bacteria|D_1__Proteobacteria|D_2__Alphaproteobacteria|D_3__Caulobacterales|D_4__Caulobacteraceae|D_5__Brevundimonas|NA|7b054f04ff8194f7a97cbbd55455ceef"
## [6] "D_0__Bacteria|D_1__Proteobacteria|D_2__Alphaproteobacteria|D_3__Rhizobiales|D_4__Devosiaceae|D_5__Pelagibacterium|NA|7082a7b15693f29fda85e73fe87292fb"
## [7] "D_0__Bacteria|D_1__Proteobacteria|D_2__Alphaproteobacteria|D_3__Rhizobiales|D_4__Rhizobiaceae|D_5__Allorhizobium-Neorhizobium-Pararhizobium-Rhizobium|NA|438dc2a2ea2405a782df5830ae62100e"
## [8] "D_0__Bacteria|D_1__Firmicutes|D_2__Clostridia|D_3__Clostridiales|D_4__Lachnospiraceae|D_5__Dorea|NA|90a05d597112b554e4480a8eaae4e0aa"
## [9] "D_0__Bacteria|D_1__Firmicutes|D_2__Clostridia|D_3__Clostridiales|D_4__Ruminococcaceae|D_5__Faecalibacterium|NA|23fed68c6c76ab10ba1be8a43e9176e7"
## [10] "D_0__Bacteria|D_1__Firmicutes|D_2__Clostridia|D_3__Clostridiales|D_4__Ruminococcaceae|D_5__Faecalibacterium|NA|c3bdda568b2c1580d5cce7407ef43909"
## [11] "D_0__Bacteria|D_1__Firmicutes|D_2__Clostridia|D_3__Clostridiales|D_4__Lachnospiraceae|D_5__[Ruminococcus] torques group|NA|9154fa6a1f8d4e5c562994121242f5b5"
## [12] "D_0__Bacteria|D_1__Firmicutes|D_2__Clostridia|D_3__Clostridiales|D_4__Lachnospiraceae|NA|NA|51dd453b71a4aba20d9f349601ea5203"
## [13] "D_0__Bacteria|D_1__Bacteroidetes|D_2__Bacteroidia|D_3__Bacteroidales|D_4__Prevotellaceae|D_5__Prevotella 9|NA|351c1c41eb96b3ed2975bfe7f01eb9d7"
## [14] "D_0__Bacteria|D_1__Bacteroidetes|D_2__Bacteroidia|D_3__Bacteroidales|D_4__Prevotellaceae|D_5__Prevotella 9|NA|4bf3198c78397be5af0b7325d20558de"

#Select and count the most and least targeted taxa by average; FDR<0.05, mean index >0 or <0
most_targeted_mean_all <- dplyr::filter(wilcox.data1, rel.fdr<0.05&mean>0)
targeted_taxa <- IgTAX75[which(IgTAX75$combined %in% most_targeted_mean_all$Taxa),]
target_count <- as.data.frame(dplyr::count(targeted_taxa, Rank2))
target_count$percent <- (target_count$n/sum(target_count$n))*100
target_count

## Rank2 n percent
## 1 D_1__Actinobacteria 1 2.857143
## 2 D_1__Bacteroidetes 8 22.857143
## 3 D_1__Cyanobacteria 1 2.857143
## 4 D_1__Firmicutes 24 68.571429
## 5 D_1__Proteobacteria 1 2.857143

sum(target_count$n)

## [1] 35

least_targeted_mean_all <- dplyr::filter(wilcox.data1, rel.fdr<0.05&mean<0)
targeted_taxa <- IgTAX75[which(IgTAX75$combined %in% least_targeted_mean_all$Taxa),]
target_count <- as.data.frame(dplyr::count(targeted_taxa, Rank2))
target_count$percent <- (target_count$n/sum(target_count$n))*100
target_count

## Rank2 n percent
## 1 D_1__Actinobacteria 1 2.631579
## 2 D_1__Bacteroidetes 7 18.421053
## 3 D_1__Firmicutes 23 60.526316
## 4 D_1__Proteobacteria 7 18.421053

sum(target_count$n)

## [1] 38

## Defining main IgA-targeted taxa, Madagascar (ASV level)

##filter by Country - Madagascar##
meta_wilcox_filt = filter(meta_wilcox, Country=="Mada")
samples_kept <- meta_wilcox_filt$ChildID
IgA_simplewilcox_filt <- filter(IgA_simplewilcox, rownames(IgA_simplewilcox) %in% samples_kept)

#make table from filtered df
wilcox.data2 <- sapply(1:ncol(IgA_simplewilcox_filt), function(i){wilcox.test(IgA_simplewilcox_filt[,i], mu=0)$p.value})
wilcox.data2 <- as.data.frame(wilcox.data2)
rownames(wilcox.data2) <- colnames(IgA_simplewilcox_filt)
#Perform multiple comparison correction using FDR
wilcox.data2$rel.fdr <- p.adjust(wilcox.data2$wilcox.data2, method="fdr")
#incorporate other metrics of data centre and dispersion
wilcox.data2$mean <- t(summarise_each(IgA_simplewilcox_filt,funs(mean)))
wilcox.data2$median <- t(summarise_each(IgA_simplewilcox_filt,funs(median)))
wilcox.data2$magnitude <- abs(wilcox.data2$mean)
wilcox.data2$variance <- t(summarise_each(IgA_simplewilcox_filt,funs(var)))
wilcox.data2$IQR <- t(summarise_each(IgA_simplewilcox_filt,funs(IQR))) #interquartile range
wilcox.data2$Taxa <- row.names(wilcox.data2)
wilcox.data2$SampleType <- c("Mada")

#Selecting the most targeted taxa; FDR<0.05, median index >0
most_targeted_Mada <- dplyr::filter(wilcox.data2, rel.fdr<0.05&median>0)
most_targeted_Mada$Taxa #7

## [1] "D_0__Bacteria|D_1__Firmicutes|D_2__Erysipelotrichia|D_3__Erysipelotrichales|D_4__Erysipelotrichaceae|D_5__Solobacterium|D_6__uncultured bacterium|4615dffd5616f456ec13b4b8b2e2c556"
## [2] "D_0__Bacteria|D_1__Proteobacteria|D_2__Gammaproteobacteria|D_3__Pasteurellales|D_4__Pasteurellaceae|D_5__Haemophilus|NA|e27680d4009f98f30248d823bc17fb8e"
## [3] "D_0__Bacteria|D_1__Firmicutes|D_2__Clostridia|D_3__Clostridiales|D_4__Ruminococcaceae|D_5__Subdoligranulum|NA|a1a300c872047a7d8b6b294b17cfb63b"
## [4] "D_0__Bacteria|D_1__Firmicutes|D_2__Clostridia|D_3__Clostridiales|D_4__Ruminococcaceae|D_5__Subdoligranulum|NA|a180485ac456840a8e7a0014a4bcd598"
## [5] "D_0__Bacteria|D_1__Firmicutes|D_2__Clostridia|D_3__Clostridiales|D_4__Lachnospiraceae|D_5__Lachnospiraceae UCG-004|NA|f30ac5063ec37bc24a902343326db800"
## [6] "D_0__Bacteria|D_1__Firmicutes|D_2__Clostridia|D_3__Clostridiales|D_4__Lachnospiraceae|D_5__Dorea|NA|afd87e82de329a1ed75b98b5b606843c"
## [7] "D_0__Bacteria|D_1__Bacteroidetes|D_2__Bacteroidia|D_3__Bacteroidales|D_4__Prevotellaceae|D_5__Prevotella 9|D_6__uncultured bacterium|dad9c1aff4e6d7faab64199863616bc7"

#Selecting the least targeted taxa; FDR<0.05, median index <0
least_targeted_Mada <- dplyr::filter(wilcox.data2, rel.fdr<0.05&median<0)
least_targeted_Mada$Taxa #14

## [1] "D_0__Bacteria|D_1__Firmicutes|D_2__Erysipelotrichia|D_3__Erysipelotrichales|D_4__Erysipelotrichaceae|D_5__Holdemanella|D_6__uncultured bacterium|10541b6401f0ea0b38e3fcd6265d3c86"
## [2] "D_0__Bacteria|D_1__Firmicutes|D_2__Bacilli|D_3__Lactobacillales|D_4__Streptococcaceae|D_5__Streptococcus|NA|bd2ebc70501f7d867c204f94c4e483da"
## [3] "D_0__Bacteria|D_1__Firmicutes|D_2__Negativicutes|D_3__Selenomonadales|D_4__Veillonellaceae|D_5__Dialister|NA|d6960828e54e3b0059ec5cdf24b16d2f"
## [4] "D_0__Bacteria|D_1__Proteobacteria|D_2__Gammaproteobacteria|D_3__Pseudomonadales|D_4__Pseudomonadaceae|D_5__Pseudomonas|NA|402e5913597695a16d7cad415ffff02f"
## [5] "D_0__Bacteria|D_1__Proteobacteria|D_2__Gammaproteobacteria|D_3__Pseudomonadales|D_4__Pseudomonadaceae|D_5__Pseudomonas|NA|1f878f615fcfc8d7bd381a7841ac1e41"
## [6] "D_0__Bacteria|D_1__Proteobacteria|D_2__Alphaproteobacteria|D_3__Caulobacterales|D_4__Caulobacteraceae|D_5__Brevundimonas|NA|7b054f04ff8194f7a97cbbd55455ceef"
## [7] "D_0__Bacteria|D_1__Proteobacteria|D_2__Alphaproteobacteria|D_3__Rhizobiales|D_4__Devosiaceae|D_5__Pelagibacterium|NA|7082a7b15693f29fda85e73fe87292fb"
## [8] "D_0__Bacteria|D_1__Firmicutes|D_2__Clostridia|D_3__Clostridiales|D_4__Lachnospiraceae|D_5__Dorea|NA|90a05d597112b554e4480a8eaae4e0aa"
## [9] "D_0__Bacteria|D_1__Firmicutes|D_2__Clostridia|D_3__Clostridiales|D_4__Lachnospiraceae|D_5__Agathobacter|NA|2d34c22edce4b1f2d8a5228ad78f8ea8"
## [10] "D_0__Bacteria|D_1__Firmicutes|D_2__Clostridia|D_3__Clostridiales|D_4__Ruminococcaceae|D_5__Faecalibacterium|NA|23fed68c6c76ab10ba1be8a43e9176e7"
## [11] "D_0__Bacteria|D_1__Firmicutes|D_2__Clostridia|D_3__Clostridiales|D_4__Ruminococcaceae|D_5__Faecalibacterium|NA|c3bdda568b2c1580d5cce7407ef43909"
## [12] "D_0__Bacteria|D_1__Firmicutes|D_2__Clostridia|D_3__Clostridiales|D_4__Lachnospiraceae|D_5__Blautia|NA|ee293984c0110b2eeceb8427fdf448fb"
## [13] "D_0__Bacteria|D_1__Firmicutes|D_2__Clostridia|D_3__Clostridiales|D_4__Lachnospiraceae|D_5__Blautia|NA|c1dc9ad5116d96b8ed863458fc0d0aec"
## [14] "D_0__Bacteria|D_1__Firmicutes|D_2__Clostridia|D_3__Clostridiales|D_4__Lachnospiraceae|NA|NA|51dd453b71a4aba20d9f349601ea5203"
## [15] "D_0__Bacteria|D_1__Bacteroidetes|D_2__Bacteroidia|D_3__Bacteroidales|D_4__Prevotellaceae|D_5__Prevotella 9|NA|351c1c41eb96b3ed2975bfe7f01eb9d7"
## [16] "D_0__Bacteria|D_1__Bacteroidetes|D_2__Bacteroidia|D_3__Bacteroidales|D_4__Prevotellaceae|D_5__Prevotella 9|NA|4bf3198c78397be5af0b7325d20558de"

#plot most targeted ASV - Madagascar
#collect IgA Index and taxa data into a data frame
targeted_index <- IgA_simplewilcox_filt[,which(names(IgA_simplewilcox_filt) %in% most_targeted_Mada$Taxa)]
targeted_taxa <- IgTAX75[which(IgTAX75$combined %in% most_targeted_Mada$Taxa),]
targeted_taxa$Simple <- paste(targeted_taxa$Rank6, substr(targeted_taxa$ASV, start=0, stop=3))
targeted_taxa$Simple <- gsub("D_5__", "", targeted_taxa$Simple)
targeted_taxa$Phylum <- gsub("D_1__", "", targeted_taxa$Rank2)

pdata1 <- data.frame(IgAIndex=targeted_index[,1], Taxa=targeted_taxa$Simple[1], Phylum=targeted_taxa$Phylum[1])
pdata2 <- data.frame(IgAIndex=targeted_index[,2], Taxa=targeted_taxa$Simple[2], Phylum=targeted_taxa$Phylum[2])
pdata3 <- data.frame(IgAIndex=targeted_index[,3], Taxa=targeted_taxa$Simple[3], Phylum=targeted_taxa$Phylum[3])
pdata4 <- data.frame(IgAIndex=targeted_index[,4], Taxa=targeted_taxa$Simple[4], Phylum=targeted_taxa$Phylum[4])
pdata5 <- data.frame(IgAIndex=targeted_index[,5], Taxa=targeted_taxa$Simple[5], Phylum=targeted_taxa$Phylum[5])
pdata6 <- data.frame(IgAIndex=targeted_index[,6], Taxa=targeted_taxa$Simple[6], Phylum=targeted_taxa$Phylum[6])
pdata7 <- data.frame(IgAIndex=targeted_index[,7], Taxa=targeted_taxa$Simple[7], Phylum=targeted_taxa$Phylum[7])

pdata <- Reduce(full_join, list(pdata1, pdata2, pdata3, pdata4, pdata5, pdata6, pdata7))

## Joining, by = c("IgAIndex", "Taxa", "Phylum")
## Joining, by = c("IgAIndex", "Taxa", "Phylum")
## Joining, by = c("IgAIndex", "Taxa", "Phylum")
## Joining, by = c("IgAIndex", "Taxa", "Phylum")
## Joining, by = c("IgAIndex", "Taxa", "Phylum")
## Joining, by = c("IgAIndex", "Taxa", "Phylum")

pdata$Taxa <- gsub("Lachnospiraceae UCG-004 f30", "Lachnospiraceae f30", pdata$Taxa)

#plot - most targeted ASV Mada
p <- ggplot(pdata) + geom_jitter(aes(x=Taxa, y=IgAIndex, color=Phylum),
 width=0.2, height=0, shape=1, size=3)
p <- p + geom_boxplot(aes(x=Taxa, y=IgAIndex), colour="black", fill=NA, outlier.shape=NA)
p <- p + ggtitle("Most Targeted ASV Mada")
p <- p + theme_bw(base_size=16)
p <- p + theme(axis.text.x = element_text(angle=45, hjust=1))
p <- p + ylim(c(-1, 1))
p <- p + scale_color_manual(values=c("darkgreen", "deepskyblue3", "indianred3"))
p


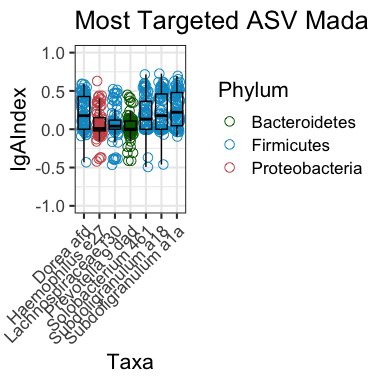


## Defining main IgA-targeted taxa, Central African Republic (ASV level)

##filter by Country - CAR##
meta_wilcox_filt = filter(meta_wilcox, Country=="RCA")
samples_kept <- meta_wilcox_filt$ChildID
IgA_simplewilcox_filt <- filter(IgA_simplewilcox, rownames(IgA_simplewilcox) %in% samples_kept)

#make table from filtered df
wilcox.data3 <- sapply(1:ncol(IgA_simplewilcox_filt), function(i){wilcox.test(IgA_simplewilcox_filt[,i], mu=0)$p.value})
wilcox.data3 <- as.data.frame(wilcox.data3)
rownames(wilcox.data3) <- colnames(IgA_simplewilcox_filt)
#Perform multiple comparison correction using FDR
wilcox.data3$rel.fdr <- p.adjust(wilcox.data3$wilcox.data3, method="fdr")
#incorporate other metrics of data centre and dispersion
wilcox.data3$mean <- t(summarise_each(IgA_simplewilcox_filt,funs(mean)))
wilcox.data3$median <- t(summarise_each(IgA_simplewilcox_filt,funs(median)))
wilcox.data3$magnitude <- abs(wilcox.data3$mean)
wilcox.data3$variance <- t(summarise_each(IgA_simplewilcox_filt,funs(var)))
wilcox.data3$IQR <- t(summarise_each(IgA_simplewilcox_filt,funs(IQR))) #interquartile range
wilcox.data3$Taxa <- row.names(wilcox.data3)
wilcox.data3$SampleType <- c("CAR")

#Selecting the most targeted taxa; FDR<0.05, median index >0
most_targeted_RCA <- dplyr::filter(wilcox.data3, rel.fdr<0.05&median>0)
most_targeted_RCA$Taxa #5

## [1] "D_0__Bacteria|D_1__Proteobacteria|D_2__Gammaproteobacteria|D_3__Pasteurellales|D_4__Pasteurellaceae|D_5__Haemophilus|NA|e27680d4009f98f30248d823bc17fb8e"
## [2] "D_0__Bacteria|D_1__Firmicutes|D_2__Clostridia|D_3__Clostridiales|D_4__Ruminococcaceae|D_5__Subdoligranulum|NA|a1a300c872047a7d8b6b294b17cfb63b"
## [3] "D_0__Bacteria|D_1__Firmicutes|D_2__Clostridia|D_3__Clostridiales|D_4__Lachnospiraceae|D_5__Blautia|NA|c1dc9ad5116d96b8ed863458fc0d0aec"
## [4] "D_0__Bacteria|D_1__Firmicutes|D_2__Clostridia|D_3__Clostridiales|D_4__Lachnospiraceae|D_5__Dorea|NA|afd87e82de329a1ed75b98b5b606843c"
## [5] "D_0__Bacteria|D_1__Firmicutes|D_2__Clostridia|D_3__Clostridiales|D_4__Lachnospiraceae|D_5__Coprococcus 3|NA|a0b7d83fb64749f9a4b15b3728425b97"

#Selecting the least targeted taxa; FDR<0.05, median index <0
least_targeted_RCA <- dplyr::filter(wilcox.data3, rel.fdr<0.05&median<0)
least_targeted_RCA$Taxa #8

## [1] "D_0__Bacteria|D_1__Bacteroidetes|D_2__Bacteroidia|D_3__Bacteroidales|D_4__Bacteroidaceae|D_5__Bacteroides|D_6__Bacteroides thetaiotaomicron|51e441cbdcc80da0656e82293ae160b5"
## [2] "D_0__Bacteria|D_1__Bacteroidetes|D_2__Bacteroidia|D_3__Bacteroidales|D_4__Bacteroidaceae|D_5__Bacteroides|NA|668fdb718997fc1589c7817655d4bb5f"
## [3] "D_0__Bacteria|D_1__Proteobacteria|D_2__Gammaproteobacteria|D_3__Pseudomonadales|D_4__Pseudomonadaceae|D_5__Pseudomonas|NA|402e5913597695a16d7cad415ffff02f"
## [4] "D_0__Bacteria|D_1__Proteobacteria|D_2__Gammaproteobacteria|D_3__Pseudomonadales|D_4__Pseudomonadaceae|D_5__Pseudomonas|NA|1f878f615fcfc8d7bd381a7841ac1e41"
## [5] "D_0__Bacteria|D_1__Firmicutes|D_2__Clostridia|D_3__Clostridiales|D_4__Ruminococcaceae|D_5__Faecalibacterium|NA|23fed68c6c76ab10ba1be8a43e9176e7"
## [6] "D_0__Bacteria|D_1__Firmicutes|D_2__Clostridia|D_3__Clostridiales|D_4__Lachnospiraceae|NA|NA|3481bdc6a2a89366f073a9d46a436cf3"
## [7] "D_0__Bacteria|D_1__Firmicutes|D_2__Clostridia|D_3__Clostridiales|D_4__Lachnospiraceae|D_5__[Ruminococcus] torques group|NA|9154fa6a1f8d4e5c562994121242f5b5"
## [8] "D_0__Bacteria|D_1__Bacteroidetes|D_2__Bacteroidia|D_3__Bacteroidales|D_4__Prevotellaceae|D_5__Prevotella 9|NA|4bf3198c78397be5af0b7325d20558de"

#plot most targeted ASV - RCA
#collect IgA Index and taxa data into a data frame
targeted_index <- IgA_simplewilcox_filt[,which(names(IgA_simplewilcox_filt) %in% most_targeted_RCA$Taxa)]
targeted_taxa <- IgTAX75[which(IgTAX75$combined %in% most_targeted_RCA$Taxa),]
targeted_taxa$Simple <- paste(targeted_taxa$Rank6, substr(targeted_taxa$ASV, start=0, stop=3))
targeted_taxa$Simple <- gsub("D_5__", "", targeted_taxa$Simple)
targeted_taxa$Phylum <- gsub("D_1__", "", targeted_taxa$Rank2)

pdata1 <- data.frame(IgAIndex=targeted_index[,1], Taxa=targeted_taxa$Simple[1], Phylum=targeted_taxa$Phylum[1])
pdata2 <- data.frame(IgAIndex=targeted_index[,2], Taxa=targeted_taxa$Simple[2], Phylum=targeted_taxa$Phylum[2])
pdata3 <- data.frame(IgAIndex=targeted_index[,3], Taxa=targeted_taxa$Simple[3], Phylum=targeted_taxa$Phylum[3])
pdata4 <- data.frame(IgAIndex=targeted_index[,4], Taxa=targeted_taxa$Simple[4], Phylum=targeted_taxa$Phylum[4])
pdata5 <- data.frame(IgAIndex=targeted_index[,5], Taxa=targeted_taxa$Simple[5], Phylum=targeted_taxa$Phylum[5])

pdata <- Reduce(full_join, list(pdata1, pdata2, pdata3, pdata4, pdata5))

## Joining, by = c("IgAIndex", "Taxa", "Phylum")
## Joining, by = c("IgAIndex", "Taxa", "Phylum")
## Joining, by = c("IgAIndex", "Taxa", "Phylum")
## Joining, by = c("IgAIndex", "Taxa", "Phylum")

#plot - most targeted CAR
p <- ggplot(pdata) + geom_jitter(aes(x=Taxa, y=IgAIndex, color=Phylum),
 width=0.2, height=0, shape=1, size=3)
p <- p + geom_boxplot(aes(x=Taxa, y=IgAIndex), colour="black", fill=NA, outlier.shape=NA)
p <- p + ggtitle("Most Targeted ASV CAR")
p <- p + theme_bw(base_size=16)
p <- p + theme(axis.text.x = element_text(angle=45, hjust=1))
p <- p + ylim(c(-1, 1))
p <- p + scale_color_manual(values=c("deepskyblue3", "indianred3"))
p


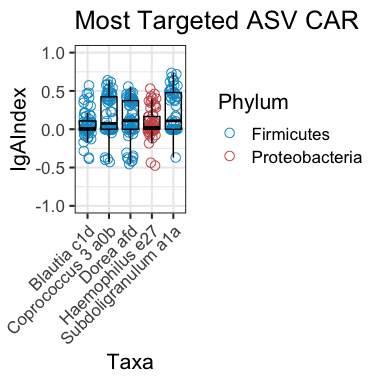


## Bubble plot of the main IgA-targeted taxa in full dataset, Mada, and CAR at ASV level - Fig S5A

###make a bubble plot of all targeted taxa with FDR<0.05
names(wilcox.data1)[names(wilcox.data1) == 'wilcox.data1'] <- 'wilcox.data'
names(wilcox.data2)[names(wilcox.data2) == 'wilcox.data2'] <- 'wilcox.data'
names(wilcox.data3)[names(wilcox.data3) == 'wilcox.data3'] <- 'wilcox.data'
bubble.all <- Reduce(full_join, list(wilcox.data1,wilcox.data2, wilcox.data3))

## Joining, by = c("wilcox.data", "rel.fdr", "mean", "median", "magnitude", "variance", "IQR", "Taxa", "SampleType")
## Joining, by = c("wilcox.data", "rel.fdr", "mean", "median", "magnitude", "variance", "IQR", "Taxa", "SampleType")

#add a "p value category"
bubble.all$p.category <- bubble.all$wilcox.data
bubble.all$p.category[bubble.all$rel.fdr<0.05] <- "p<0.05"
bubble.all$p.category[bubble.all$rel.fdr<0.01] <- "p<0.01"
bubble.all$p.category[bubble.all$rel.fdr<0.001] <- "p<0.001"
bubble.all$p.category[bubble.all$rel.fdr<0.0001] <- "p<0.0001"
bubble.all$p.category[bubble.all$rel.fdr>=0.05] <- "NS"

#add a pos/neg category ####need to retain an actual average column as well as magnitude
bubble.all$posneg <- bubble.all$mean
bubble.all$posneg[bubble.all$mean>0] <- "pos"
bubble.all$posneg[bubble.all$mean<0] <- "neg"

#combine into 1 p value category
bubble.all$p.value.category <- paste(bubble.all$p.category, bubble.all$posneg, sep="")

#simplify by keeping only Taxa with at least one significance value.
bubble.simple <- filter(bubble.all, rel.fdr<0.05)
TaxaToKeep <- bubble.simple$Taxa
bubble.filter <- filter(bubble.all, Taxa %in% TaxaToKeep)
TaxaNames <- filter(IgTAX75, IgTAX75$combined %in% bubble.filter$Taxa)
TaxaNames$Simple <- gsub("D_5__", "", TaxaNames$Rank6)
TaxaNames$Simple <- ifelse(TaxaNames$Simple=="uncultured"|TaxaNames$Simple=="uncultured bacterium",
 yes = as.character(TaxaNames$Rank5), no=TaxaNames$Simple)
TaxaNames$Simple <- gsub("D_4__", "", TaxaNames$Simple)
TaxaNames$Simple <- ifelse(TaxaNames$Simple=="uncultured"|TaxaNames$Simple=="uncultured bacterium",
 yes = as.character(TaxaNames$Rank4), no=TaxaNames$Simple)
TaxaNames$Simple <- gsub("D_3__", "", TaxaNames$Simple)
TaxaNames$Simple <- gsub("Allorhizobium-Neorhizobium-Pararhizobium-Rhizobium", "Rhizobium", TaxaNames$Simple,
 fixed=TRUE)
TaxaNames$SimpleASV <- paste(TaxaNames$Simple, substr(TaxaNames$ASV, start=0, stop=3), sep=" ")
TaxaNames$Taxa <- TaxaNames$combined
bubble.filter <- full_join(bubble.filter, TaxaNames, by="Taxa")

###bubble plot
bubble.filter$SampleType <- factor(bubble.filter$SampleType, levels = c("All", "Mada", "CAR"))
bubble.filter$p.value.category <- factor(bubble.filter$p.value.category, levels = c("p<0.0001neg", "p<0.001neg", "p<0.01neg", "p<0.05neg", "NSneg",
 "NS", "NSpos", "p<0.05pos", "p<0.01pos", "p<0.001pos", "p<0.0001pos"))

p <- ggplot(bubble.filter, aes(x=SampleType, y=SimpleASV, size=magnitude)) + geom_point(shape=21, aes(fill=p.value.category), colour="grey30")
p <- p + scale_fill_manual(values=c("p<0.0001pos"="indianred4",
 "p<0.001pos"="indianred2",
 "p<0.01pos"="lightpink2",
 "p<0.05pos"="lightpink2",
 "NSpos"="ivory",
 "p<0.0001neg"="deepskyblue4",
 "p<0.001neg"="deepskyblue3",
 "p<0.01neg"="skyblue2",
 "p<0.05neg"="skyblue2",
 "NSneg"="aliceblue",
 "NS"="grey80"))
p <- p + theme(axis.text.x = element_text(angle=90, vjust=0.5, size=12), legend.key.size=unit(0.75, "cm"))
p <- p + scale_y_discrete(limits = rev(levels(bubble.filter$Taxa)))
p <- p + theme(strip.background = element_rect(fill="gray85"),
 panel.background = element_rect(fill="white"),
 panel.border = element_rect(colour="black", linetype="solid", fill="transparent")
)
p <- p + facet_grid(.~SampleType, scales = "free", space = "free")
p <- p + guides(colour=FALSE, size=FALSE, shape=FALSE, fill=FALSE)
p


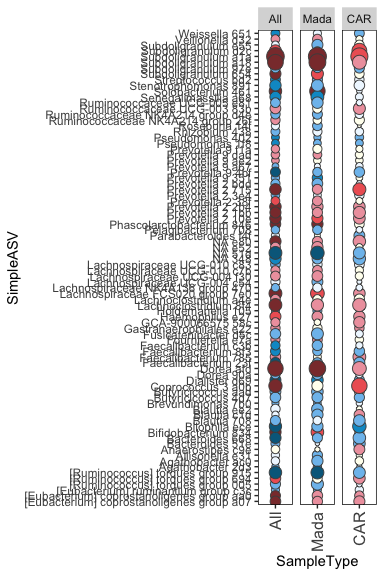


# Fig 2: Main IgA-targeted taxa (genus level)

## Main IgA-targeted taxa, full dataset (genus level) - Fig 2B

#select data table and metadata, filter for category as necessary
IgA_simplewilcox <- IgA_batchtrim75_genus_t
meta_wilcox <- as.data.frame(sample_data(IgPos_forindexGen))
row.names(IgA_simplewilcox) <- meta_wilcox$ChildID

##ALL - full dataset##
#apply wilcox test to test if Index is sig. diff. from 0
wilcox.dataGenus <- sapply(1:ncol(IgA_simplewilcox), function(i){wilcox.test(IgA_simplewilcox[,i], mu=0)$p.value})
wilcox.dataGenus <- as.data.frame(wilcox.dataGenus)
rownames(wilcox.dataGenus) <- colnames(IgA_simplewilcox)
#Perform multiple comparison correction using FDR
wilcox.dataGenus$rel.fdr <- p.adjust(wilcox.dataGenus$wilcox.dataGenus, method="fdr")
#incorporate other metrics of data centre and dispersion
wilcox.dataGenus$mean <- t(summarise_each(IgA_simplewilcox,funs(mean)))
wilcox.dataGenus$median <- t(summarise_each(IgA_simplewilcox,funs(median)))
wilcox.dataGenus$magnitude <- abs(wilcox.dataGenus$mean)
wilcox.dataGenus$variance <- t(summarise_each(IgA_simplewilcox,funs(var)))
wilcox.dataGenus$IQR <- t(summarise_each(IgA_simplewilcox,funs(IQR))) #interquartile range
wilcox.dataGenus$Taxa <- row.names(wilcox.dataGenus)
wilcox.dataGenus$SampleType <- c("All")

#Selecting the most targeted taxa; FDR<0.05, median index >0
most_targeted_all <- dplyr::filter(wilcox.dataGenus, rel.fdr<0.05&median>0)
most_targeted_all$Taxa

## [1] "D_0__Bacteria|D_1__Proteobacteria|D_2__Gammaproteobacteria|D_3__Pasteurellales|D_4__Pasteurellaceae|D_5__Haemophilus"
## [2] "D_0__Bacteria|D_1__Firmicutes|D_2__Clostridia|D_3__Clostridiales|D_4__Lachnospiraceae|D_5__Dorea"
## [3] "D_0__Bacteria|D_1__Firmicutes|D_2__Clostridia|D_3__Clostridiales|D_4__Ruminococcaceae|D_5__Ruminococcaceae UCG-002"
## [4] "D_0__Bacteria|D_1__Firmicutes|D_2__Clostridia|D_3__Clostridiales|D_4__Ruminococcaceae|D_5__[Eubacterium] coprostanoligenes group"
## [5] "D_0__Bacteria|D_1__Firmicutes|D_2__Clostridia|D_3__Clostridiales|D_4__Ruminococcaceae|D_5__Subdoligranulum"
## [6] "D_0__Bacteria|D_1__Firmicutes|D_2__Clostridia|D_3__Clostridiales|D_4__Lachnospiraceae|D_5__Lachnoclostridium"
## [7] "D_0__Bacteria|D_1__Firmicutes|D_2__Clostridia|D_3__Clostridiales|D_4__Lachnospiraceae|D_5__Coprococcus 3"
## [8] "D_0__Bacteria|D_1__Bacteroidetes|D_2__Bacteroidia|D_3__Bacteroidales|D_4__Prevotellaceae|D_5__Prevotella 2"

least_targeted_all <- dplyr::filter(wilcox.dataGenus, rel.fdr<0.05&median<0)
least_targeted_all$Taxa

## [1] "D_0__Bacteria|D_1__Firmicutes|D_2__Bacilli|D_3__Lactobacillales|D_4__Streptococcaceae|D_5__Streptococcus"
## [2] "D_0__Bacteria|D_1__Firmicutes|D_2__Negativicutes|D_3__Selenomonadales|D_4__Veillonellaceae|D_5__Dialister"
## [3] "D_0__Bacteria|D_1__Bacteroidetes|D_2__Bacteroidia|D_3__Bacteroidales|D_4__Tannerellaceae|D_5__Parabacteroides"
## [4] "D_0__Bacteria|D_1__Bacteroidetes|D_2__Bacteroidia|D_3__Bacteroidales|D_4__Bacteroidaceae|D_5__Bacteroides"
## [5] "D_0__Bacteria|D_1__Proteobacteria|D_2__Gammaproteobacteria|D_3__Pseudomonadales|D_4__Pseudomonadaceae|D_5__Pseudomonas"
## [6] "D_0__Bacteria|D_1__Proteobacteria|D_2__Gammaproteobacteria|D_3__Xanthomonadales|D_4__Xanthomonadaceae|D_5__Stenotrophomonas"
## [7] "D_0__Bacteria|D_1__Proteobacteria|D_2__Alphaproteobacteria|D_3__Caulobacterales|D_4__Caulobacteraceae|D_5__Brevundimonas"
## [8] "D_0__Bacteria|D_1__Proteobacteria|D_2__Alphaproteobacteria|D_3__Rhizobiales|D_4__Devosiaceae|D_5__Pelagibacterium"
## [9] "D_0__Bacteria|D_1__Proteobacteria|D_2__Alphaproteobacteria|D_3__Rhizobiales|D_4__Rhizobiaceae|D_5__Allorhizobium-Neorhizobium-Pararhizobium-Rhizobium"
## [10] "D_0__Bacteria|D_1__Firmicutes|D_2__Clostridia|D_3__Clostridiales|D_4__Lachnospiraceae|D_5__Roseburia"
## [11] "D_0__Bacteria|D_1__Firmicutes|D_2__Clostridia|D_3__Clostridiales|D_4__Lachnospiraceae|D_5__Agathobacter"
## [12] "D_0__Bacteria|D_1__Firmicutes|D_2__Clostridia|D_3__Clostridiales|D_4__Ruminococcaceae|D_5__Butyricicoccus"
## [13] "D_0__Bacteria|D_1__Firmicutes|D_2__Clostridia|D_3__Clostridiales|D_4__Ruminococcaceae|D_5__Faecalibacterium"
## [14] "D_0__Bacteria|D_1__Firmicutes|D_2__Clostridia|D_3__Clostridiales|D_4__Lachnospiraceae|D_5__Blautia"
## [15] "D_0__Bacteria|D_1__Firmicutes|D_2__Clostridia|D_3__Clostridiales|D_4__Lachnospiraceae|D_5__uncultured"
## [16] "D_0__Bacteria|D_1__Bacteroidetes|D_2__Bacteroidia|D_3__Bacteroidales|D_4__Prevotellaceae|D_5__Prevotella 9"

#by mean
most_targeted_all_mean <- dplyr::filter(wilcox.dataGenus, rel.fdr<0.05&mean>0)
most_targeted_all_mean$Taxa

## [1] "D_0__Bacteria|D_1__Firmicutes|D_2__Erysipelotrichia|D_3__Erysipelotrichales|D_4__Erysipelotrichaceae|D_5__Solobacterium"
## [2] "D_0__Bacteria|D_1__Cyanobacteria|D_2__Melainabacteria|D_3__Gastranaerophilales|D_4__uncultured bacterium|D_5__uncultured bacterium"
## [3] "D_0__Bacteria|D_1__Firmicutes|D_2__Negativicutes|D_3__Selenomonadales|D_4__Acidaminococcaceae|D_5__Phascolarctobacterium"
## [4] "D_0__Bacteria|D_1__Actinobacteria|D_2__Actinobacteria|D_3__Bifidobacteriales|D_4__Bifidobacteriaceae|D_5__Bifidobacterium"
## [5] "D_0__Bacteria|D_1__Epsilonbacteraeota|D_2__Campylobacteria|D_3__Campylobacterales|D_4__Campylobacteraceae|D_5__Campylobacter"
## [6] "D_0__Bacteria|D_1__Bacteroidetes|D_2__Bacteroidia|D_3__Bacteroidales|D_4__Rikenellaceae|D_5__Alistipes"
## [7] "D_0__Bacteria|D_1__Bacteroidetes|D_2__Bacteroidia|D_3__Bacteroidales|D_4__Prevotellaceae|D_5__Alloprevotella"
## [8] "D_0__Bacteria|D_1__Bacteroidetes|D_2__Bacteroidia|D_3__Bacteroidales|D_4__Marinifilaceae|D_5__Odoribacter"
## [9] "D_0__Bacteria|D_1__Bacteroidetes|D_2__Bacteroidia|D_3__Bacteroidales|D_4__Rikenellaceae|D_5__Rikenellaceae RC9 gut group"
## [10] "D_0__Bacteria|D_1__Proteobacteria|D_2__Gammaproteobacteria|D_3__Pasteurellales|D_4__Pasteurellaceae|D_5__Haemophilus"
## [11] "D_0__Bacteria|D_1__Firmicutes|D_2__Clostridia|D_3__Clostridiales|D_4__Lachnospiraceae|D_5__GCA-900066575"
## [12] "D_0__Bacteria|D_1__Firmicutes|D_2__Clostridia|D_3__Clostridiales|D_4__Lachnospiraceae|D_5__Dorea"
## [13] "D_0__Bacteria|D_1__Firmicutes|D_2__Clostridia|D_3__Clostridiales|D_4__Ruminococcaceae|D_5__Ruminococcaceae UCG-002"
## [14] "D_0__Bacteria|D_1__Firmicutes|D_2__Clostridia|D_3__Clostridiales|D_4__Ruminococcaceae|D_5__[Eubacterium] coprostanoligenes group"
## [15] "D_0__Bacteria|D_1__Firmicutes|D_2__Clostridia|D_3__Clostridiales|D_4__Ruminococcaceae|D_5__Fournierella"
## [16] "D_0__Bacteria|D_1__Firmicutes|D_2__Clostridia|D_3__Clostridiales|D_4__Ruminococcaceae|D_5__Subdoligranulum"
## [17] "D_0__Bacteria|D_1__Firmicutes|D_2__Clostridia|D_3__Clostridiales|D_4__Lachnospiraceae|D_5__Marvinbryantia"
## [18] "D_0__Bacteria|D_1__Firmicutes|D_2__Clostridia|D_3__Clostridiales|D_4__Lachnospiraceae|D_5__Lachnospiraceae FCS020 group"
## [19] "D_0__Bacteria|D_1__Firmicutes|D_2__Clostridia|D_3__Clostridiales|D_4__Lachnospiraceae|D_5__Lachnoclostridium"
## [20] "D_0__Bacteria|D_1__Firmicutes|D_2__Clostridia|D_3__Clostridiales|D_4__Lachnospiraceae|D_5__Coprococcus 3"
## [21] "D_0__Bacteria|D_1__Bacteroidetes|D_2__Bacteroidia|D_3__Bacteroidales|D_4__Prevotellaceae|D_5__Prevotella 2"

#plot most targeted taxa - all
#collect IgA Index and taxa data into a data frame
targeted_index <- IgA_simplewilcox[,which(names(IgA_simplewilcox) %in% most_targeted_all$Taxa)]
targeted_taxa <- IgTAX75_genus[which(IgTAX75_genus$combined %in% most_targeted_all$Taxa),]
targeted_taxa$Simple <- paste(targeted_taxa$Rank6)
targeted_taxa$Simple <- gsub("D_5__", "", targeted_taxa$Simple)
targeted_taxa$Simple <- gsub(" coprostanoligenes group", "", targeted_taxa$Simple)
targeted_taxa$Phylum <- gsub("D_1__", "", targeted_taxa$Rank2)

pdata1 <- data.frame(IgAIndex=targeted_index[,1], Taxa=targeted_taxa$Simple[1], Phylum=targeted_taxa$Phylum[1])
pdata2 <- data.frame(IgAIndex=targeted_index[,2], Taxa=targeted_taxa$Simple[2], Phylum=targeted_taxa$Phylum[2])
pdata3 <- data.frame(IgAIndex=targeted_index[,3], Taxa=targeted_taxa$Simple[3], Phylum=targeted_taxa$Phylum[3])
pdata4 <- data.frame(IgAIndex=targeted_index[,4], Taxa=targeted_taxa$Simple[4], Phylum=targeted_taxa$Phylum[4])
pdata5 <- data.frame(IgAIndex=targeted_index[,5], Taxa=targeted_taxa$Simple[5], Phylum=targeted_taxa$Phylum[5])
pdata6 <- data.frame(IgAIndex=targeted_index[,6], Taxa=targeted_taxa$Simple[6], Phylum=targeted_taxa$Phylum[6])
pdata7 <- data.frame(IgAIndex=targeted_index[,7], Taxa=targeted_taxa$Simple[7], Phylum=targeted_taxa$Phylum[7])
pdata8 <- data.frame(IgAIndex=targeted_index[,8], Taxa=targeted_taxa$Simple[8], Phylum=targeted_taxa$Phylum[8])

pdata <- Reduce(full_join, list(pdata1, pdata2, pdata3, pdata4, pdata5, pdata6, pdata7, pdata8))

## Joining, by = c("IgAIndex", "Taxa", "Phylum")
## Joining, by = c("IgAIndex", "Taxa", "Phylum")
## Joining, by = c("IgAIndex", "Taxa", "Phylum")
## Joining, by = c("IgAIndex", "Taxa", "Phylum")
## Joining, by = c("IgAIndex", "Taxa", "Phylum")
## Joining, by = c("IgAIndex", "Taxa", "Phylum")
## Joining, by = c("IgAIndex", "Taxa", "Phylum")

#plot - most targeted all
p <- ggplot(pdata) + geom_jitter(aes(x=Taxa, y=IgAIndex, color=Phylum),
 width=0.2, height=0, shape=1, size=3)
p <- p + geom_boxplot(aes(x=Taxa, y=IgAIndex), color="black", fill=NA, outlier.shape=NA)
p <- p + ggtitle("Most Targeted Overall")
p <- p + theme_bw(base_size=16)
p <- p + theme(axis.text.x = element_text(angle=45, hjust=1), axis.title.x = element_blank())
p <- p + ylim(c(-1, 1))
p <- p + scale_color_manual(values=c("darkgreen", "deepskyblue3", "indianred3"))
p


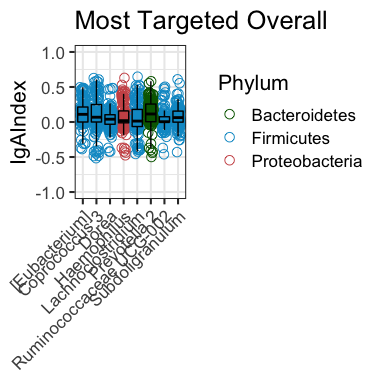


#Select and count the most and least targeted taxa by average; FDR<0.05, mean index >0 or <0
most_targeted_mean_allg <- dplyr::filter(wilcox.dataGenus, rel.fdr<0.05&mean>0)
targeted_taxa <- IgTAX75_genus[which(IgTAX75_genus$combined %in% most_targeted_mean_allg$Taxa),]
target_count <- as.data.frame(dplyr::count(targeted_taxa, Rank2))
target_count$percent <- (target_count$n/sum(target_count$n))*100
target_count

## Rank2 n percent
## 1 D_1__Actinobacteria 1 4.761905
## 2 D_1__Bacteroidetes 5 23.809524
## 3 D_1__Cyanobacteria 1 4.761905
## 4 D_1__Epsilonbacteraeota 1 4.761905
## 5 D_1__Firmicutes 12 57.142857
## 6 D_1__Proteobacteria 1 4.761905

least_targeted_mean_allg <- dplyr::filter(wilcox.dataGenus, rel.fdr<0.05&mean<0)
targeted_taxa <- IgTAX75_genus[which(IgTAX75_genus$combined %in% least_targeted_mean_allg$Taxa),]
target_count <- as.data.frame(dplyr::count(targeted_taxa, Rank2))
target_count$percent <- (target_count$n/sum(target_count$n))*100
target_count

## Rank2 n percent
## 1 D_1__Actinobacteria 1 3.225806
## 2 D_1__Bacteroidetes 3 9.677419
## 3 D_1__Firmicutes 20 64.516129
## 4 D_1__Proteobacteria 7 22.580645

## Main IgA-targeted taxa, Madagascar (genus level) - Fig 2C

##filter by Country - Madagascar - genus level##
meta_wilcox_filt = filter(meta_wilcox, Country=="Mada")
samples_kept <- meta_wilcox_filt$ChildID
IgA_simplewilcox_filt <- filter(IgA_simplewilcox, rownames(IgA_simplewilcox) %in% samples_kept)

#make table from filtered df
wilcox.dataGenus2 <- sapply(1:ncol(IgA_simplewilcox_filt), function(i){wilcox.test(IgA_simplewilcox_filt[,i], mu=0)$p.value})
wilcox.dataGenus2 <- as.data.frame(wilcox.dataGenus2)
rownames(wilcox.dataGenus2) <- colnames(IgA_simplewilcox_filt)
#Perform multiple comparison correction using FDR
wilcox.dataGenus2$rel.fdr <- p.adjust(wilcox.dataGenus2$wilcox.dataGenus2, method="fdr")
#incorporate other metrics of data centre and dispersion
wilcox.dataGenus2$mean <- t(summarise_each(IgA_simplewilcox_filt,funs(mean)))
wilcox.dataGenus2$median <- t(summarise_each(IgA_simplewilcox_filt,funs(median)))
wilcox.dataGenus2$magnitude <- abs(wilcox.dataGenus2$mean)
wilcox.dataGenus2$variance <- t(summarise_each(IgA_simplewilcox_filt,funs(var)))
wilcox.dataGenus2$IQR <- t(summarise_each(IgA_simplewilcox_filt,funs(IQR))) #interquartile range
wilcox.dataGenus2$Taxa <- row.names(wilcox.dataGenus2)
wilcox.dataGenus2$SampleType <- c("Mada")

#Selecting the most targeted taxa; FDR<0.05, median index >0
most_targeted_Mada <- dplyr::filter(wilcox.dataGenus2, rel.fdr<0.05&median>0)
most_targeted_Mada$Taxa

## [1] "D_0__Bacteria|D_1__Firmicutes|D_2__Erysipelotrichia|D_3__Erysipelotrichales|D_4__Erysipelotrichaceae|D_5__Solobacterium"
## [2] "D_0__Bacteria|D_1__Proteobacteria|D_2__Gammaproteobacteria|D_3__Pasteurellales|D_4__Pasteurellaceae|D_5__Haemophilus"
## [3] "D_0__Bacteria|D_1__Firmicutes|D_2__Clostridia|D_3__Clostridiales|D_4__Lachnospiraceae|D_5__Dorea"
## [4] "D_0__Bacteria|D_1__Firmicutes|D_2__Clostridia|D_3__Clostridiales|D_4__Ruminococcaceae|D_5__Ruminococcaceae UCG-002"
## [5] "D_0__Bacteria|D_1__Firmicutes|D_2__Clostridia|D_3__Clostridiales|D_4__Ruminococcaceae|D_5__[Eubacterium] coprostanoligenes group"
## [6] "D_0__Bacteria|D_1__Firmicutes|D_2__Clostridia|D_3__Clostridiales|D_4__Ruminococcaceae|D_5__Subdoligranulum"
## [7] "D_0__Bacteria|D_1__Firmicutes|D_2__Clostridia|D_3__Clostridiales|D_4__Lachnospiraceae|D_5__Coprococcus 3"
## [8] "D_0__Bacteria|D_1__Bacteroidetes|D_2__Bacteroidia|D_3__Bacteroidales|D_4__Prevotellaceae|D_5__Prevotella 2"

#mean
#Selecting the most targeted taxa; FDR<0.05, median index >0
most_targeted_Mada_mean <- dplyr::filter(wilcox.dataGenus2, rel.fdr<0.05&mean>0)
most_targeted_Mada_mean$Taxa

## [1] "D_0__Bacteria|D_1__Firmicutes|D_2__Erysipelotrichia|D_3__Erysipelotrichales|D_4__Erysipelotrichaceae|D_5__Solobacterium"
## [2] "D_0__Bacteria|D_1__Cyanobacteria|D_2__Melainabacteria|D_3__Gastranaerophilales|D_4__uncultured bacterium|D_5__uncultured bacterium"
## [3] "D_0__Bacteria|D_1__Firmicutes|D_2__Negativicutes|D_3__Selenomonadales|D_4__Acidaminococcaceae|D_5__Phascolarctobacterium"
## [4] "D_0__Bacteria|D_1__Actinobacteria|D_2__Actinobacteria|D_3__Bifidobacteriales|D_4__Bifidobacteriaceae|D_5__Bifidobacterium"
## [5] "D_0__Bacteria|D_1__Epsilonbacteraeota|D_2__Campylobacteria|D_3__Campylobacterales|D_4__Campylobacteraceae|D_5__Campylobacter"
## [6] "D_0__Bacteria|D_1__Bacteroidetes|D_2__Bacteroidia|D_3__Bacteroidales|D_4__Prevotellaceae|D_5__Alloprevotella"
## [7] "D_0__Bacteria|D_1__Bacteroidetes|D_2__Bacteroidia|D_3__Bacteroidales|D_4__Rikenellaceae|D_5__Rikenellaceae RC9 gut group"
## [8] "D_0__Bacteria|D_1__Proteobacteria|D_2__Gammaproteobacteria|D_3__Pasteurellales|D_4__Pasteurellaceae|D_5__Haemophilus"
## [9] "D_0__Bacteria|D_1__Firmicutes|D_2__Clostridia|D_3__Clostridiales|D_4__Lachnospiraceae|D_5__Dorea"
## [10] "D_0__Bacteria|D_1__Firmicutes|D_2__Clostridia|D_3__Clostridiales|D_4__Ruminococcaceae|D_5__Ruminococcaceae UCG-002"
## [11] "D_0__Bacteria|D_1__Firmicutes|D_2__Clostridia|D_3__Clostridiales|D_4__Ruminococcaceae|D_5__[Eubacterium] coprostanoligenes group"
## [12] "D_0__Bacteria|D_1__Firmicutes|D_2__Clostridia|D_3__Clostridiales|D_4__Ruminococcaceae|D_5__Fournierella"
## [13] "D_0__Bacteria|D_1__Firmicutes|D_2__Clostridia|D_3__Clostridiales|D_4__Ruminococcaceae|D_5__Subdoligranulum"
## [14] "D_0__Bacteria|D_1__Firmicutes|D_2__Clostridia|D_3__Clostridiales|D_4__Lachnospiraceae|D_5__Lachnospiraceae FCS020 group"
## [15] "D_0__Bacteria|D_1__Firmicutes|D_2__Clostridia|D_3__Clostridiales|D_4__Lachnospiraceae|D_5__Coprococcus 3"
## [16] "D_0__Bacteria|D_1__Bacteroidetes|D_2__Bacteroidia|D_3__Bacteroidales|D_4__Prevotellaceae|D_5__Prevotella 2"

#Selecting the least targeted taxa; FDR<0.05, median index <0
least_targeted_Mada <- dplyr::filter(wilcox.dataGenus2, rel.fdr<0.05&median<0)
least_targeted_Mada$Taxa

## [1] "D_0__Bacteria|D_1__Firmicutes|D_2__Bacilli|D_3__Lactobacillales|D_4__Streptococcaceae|D_5__Streptococcus"
## [2] "D_0__Bacteria|D_1__Firmicutes|D_2__Negativicutes|D_3__Selenomonadales|D_4__Veillonellaceae|D_5__Dialister"
## [3] "D_0__Bacteria|D_1__Proteobacteria|D_2__Gammaproteobacteria|D_3__Pseudomonadales|D_4__Pseudomonadaceae|D_5__Pseudomonas"
## [4] "D_0__Bacteria|D_1__Proteobacteria|D_2__Gammaproteobacteria|D_3__Xanthomonadales|D_4__Xanthomonadaceae|D_5__Stenotrophomonas"
## [5] "D_0__Bacteria|D_1__Proteobacteria|D_2__Alphaproteobacteria|D_3__Caulobacterales|D_4__Caulobacteraceae|D_5__Brevundimonas"
## [6] "D_0__Bacteria|D_1__Proteobacteria|D_2__Alphaproteobacteria|D_3__Rhizobiales|D_4__Devosiaceae|D_5__Pelagibacterium"
## [7] "D_0__Bacteria|D_1__Firmicutes|D_2__Clostridia|D_3__Clostridiales|D_4__Christensenellaceae|D_5__Christensenellaceae R-7 group"
## [8] "D_0__Bacteria|D_1__Firmicutes|D_2__Clostridia|D_3__Clostridiales|D_4__Lachnospiraceae|D_5__Roseburia"
## [9] "D_0__Bacteria|D_1__Firmicutes|D_2__Clostridia|D_3__Clostridiales|D_4__Lachnospiraceae|D_5__Agathobacter"
## [10] "D_0__Bacteria|D_1__Firmicutes|D_2__Clostridia|D_3__Clostridiales|D_4__Ruminococcaceae|D_5__Butyricicoccus"
## [11] "D_0__Bacteria|D_1__Firmicutes|D_2__Clostridia|D_3__Clostridiales|D_4__Ruminococcaceae|D_5__Faecalibacterium"
## [12] "D_0__Bacteria|D_1__Firmicutes|D_2__Clostridia|D_3__Clostridiales|D_4__Lachnospiraceae|D_5__Blautia"
## [13] "D_0__Bacteria|D_1__Firmicutes|D_2__Clostridia|D_3__Clostridiales|D_4__Lachnospiraceae|D_5__uncultured"
## [14] "D_0__Bacteria|D_1__Bacteroidetes|D_2__Bacteroidia|D_3__Bacteroidales|D_4__Prevotellaceae|D_5__Prevotella 9"

#plot most targeted taxa - Madagascar
#collect IgA Index and taxa data into a data frame
targeted_index <- IgA_simplewilcox_filt[,which(names(IgA_simplewilcox_filt) %in% most_targeted_Mada$Taxa)]
targeted_taxa <- IgTAX75_genus[which(IgTAX75_genus$combined %in% most_targeted_Mada$Taxa),]
targeted_taxa$Simple <- paste(targeted_taxa$Rank6)
targeted_taxa$Simple <- gsub("D_5__", "", targeted_taxa$Simple)
targeted_taxa$Simple <- gsub(" coprostanoligenes group", "", targeted_taxa$Simple)
targeted_taxa$Phylum <- gsub("D_1__", "", targeted_taxa$Rank2)

pdata1 <- data.frame(IgAIndex=targeted_index[,1], Taxa=targeted_taxa$Simple[1], Phylum=targeted_taxa$Phylum[1])
pdata2 <- data.frame(IgAIndex=targeted_index[,2], Taxa=targeted_taxa$Simple[2], Phylum=targeted_taxa$Phylum[2])
pdata3 <- data.frame(IgAIndex=targeted_index[,3], Taxa=targeted_taxa$Simple[3], Phylum=targeted_taxa$Phylum[3])
pdata4 <- data.frame(IgAIndex=targeted_index[,4], Taxa=targeted_taxa$Simple[4], Phylum=targeted_taxa$Phylum[4])
pdata5 <- data.frame(IgAIndex=targeted_index[,5], Taxa=targeted_taxa$Simple[5], Phylum=targeted_taxa$Phylum[5])
pdata6 <- data.frame(IgAIndex=targeted_index[,6], Taxa=targeted_taxa$Simple[6], Phylum=targeted_taxa$Phylum[6])
pdata7 <- data.frame(IgAIndex=targeted_index[,7], Taxa=targeted_taxa$Simple[7], Phylum=targeted_taxa$Phylum[7])
pdata8 <- data.frame(IgAIndex=targeted_index[,8], Taxa=targeted_taxa$Simple[8], Phylum=targeted_taxa$Phylum[8])

pdata <- Reduce(full_join, list(pdata1, pdata2, pdata3, pdata4, pdata5, pdata6, pdata7, pdata8))

## Joining, by = c("IgAIndex", "Taxa", "Phylum")
## Joining, by = c("IgAIndex", "Taxa", "Phylum")
## Joining, by = c("IgAIndex", "Taxa", "Phylum")
## Joining, by = c("IgAIndex", "Taxa", "Phylum")
## Joining, by = c("IgAIndex", "Taxa", "Phylum")
## Joining, by = c("IgAIndex", "Taxa", "Phylum")
## Joining, by = c("IgAIndex", "Taxa", "Phylum")

#plot - most targeted Mada
p <- ggplot(pdata) + geom_jitter(aes(x=Taxa, y=IgAIndex, color=Phylum),
 width=0.2, height=0, shape=1, size=3)
p <- p + geom_boxplot(aes(x=Taxa, y=IgAIndex), color="black", fill=NA, outlier.shape=NA)
p <- p + ggtitle("Most Targeted Mada")
p <- p + theme_bw(base_size=16)
p <- p + theme(axis.text.x = element_text(angle=45, hjust=1), axis.title.x = element_blank())
p <- p + ylim(c(-1, 1))
p <- p + scale_color_manual(values=c("darkgreen", "deepskyblue3", "indianred3"))
p


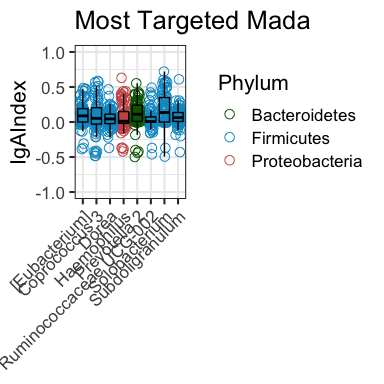


## Main IgA-targeted taxa, CAR (genus level) - Fig 2D

##filter by Country - RCA - genus##
meta_wilcox_filt = filter(meta_wilcox, Country=="RCA")
samples_kept <- meta_wilcox_filt$ChildID
IgA_simplewilcox_filt <- filter(IgA_simplewilcox, rownames(IgA_simplewilcox) %in% samples_kept)

#make table from filtered df
wilcox.dataGenus3 <- sapply(1:ncol(IgA_simplewilcox_filt), function(i){wilcox.test(IgA_simplewilcox_filt[,i], mu=0)$p.value})
wilcox.dataGenus3 <- as.data.frame(wilcox.dataGenus3)
rownames(wilcox.dataGenus3) <- colnames(IgA_simplewilcox_filt)
#Perform multiple comparison correction using FDR
wilcox.dataGenus3$rel.fdr <- p.adjust(wilcox.dataGenus3$wilcox.dataGenus3, method="fdr")
#incorporate other metrics of data centre and dispersion
wilcox.dataGenus3$mean <- t(summarise_each(IgA_simplewilcox_filt,funs(mean)))
wilcox.dataGenus3$median <- t(summarise_each(IgA_simplewilcox_filt,funs(median)))
wilcox.dataGenus3$magnitude <- abs(wilcox.dataGenus3$mean)
wilcox.dataGenus3$variance <- t(summarise_each(IgA_simplewilcox_filt,funs(var)))
wilcox.dataGenus3$IQR <- t(summarise_each(IgA_simplewilcox_filt,funs(IQR))) #interquartile range
wilcox.dataGenus3$Taxa <- row.names(wilcox.dataGenus3)
wilcox.dataGenus3$SampleType <- c("CAR")

#Selecting the most targeted taxa; FDR<0.05, median index >0
most_targeted_RCA <- dplyr::filter(wilcox.dataGenus3, rel.fdr<0.05&median>0)
most_targeted_RCA$Taxa

## [1] "D_0__Bacteria|D_1__Epsilonbacteraeota|D_2__Campylobacteria|D_3__Campylobacterales|D_4__Campylobacteraceae|D_5__Campylobacter"
## [2] "D_0__Bacteria|D_1__Proteobacteria|D_2__Gammaproteobacteria|D_3__Pasteurellales|D_4__Pasteurellaceae|D_5__Haemophilus"
## [3] "D_0__Bacteria|D_1__Firmicutes|D_2__Clostridia|D_3__Clostridiales|D_4__Ruminococcaceae|D_5__[Eubacterium] coprostanoligenes group"
## [4] "D_0__Bacteria|D_1__Firmicutes|D_2__Clostridia|D_3__Clostridiales|D_4__Ruminococcaceae|D_5__Subdoligranulum"
## [5] "D_0__Bacteria|D_1__Firmicutes|D_2__Clostridia|D_3__Clostridiales|D_4__Lachnospiraceae|D_5__Lachnoclostridium"
## [6] "D_0__Bacteria|D_1__Firmicutes|D_2__Clostridia|D_3__Clostridiales|D_4__Lachnospiraceae|D_5__Coprococcus 3"
## [7] "D_0__Bacteria|D_1__Bacteroidetes|D_2__Bacteroidia|D_3__Bacteroidales|D_4__Prevotellaceae|D_5__Prevotella 2"

#Selecting the least targeted taxa; FDR<0.05, median index <0
least_targeted_RCA <- dplyr::filter(wilcox.dataGenus3, rel.fdr<0.05&median<0)
least_targeted_RCA$Taxa #11

## [1] "D_0__Bacteria|D_1__Firmicutes|D_2__Bacilli|D_3__Lactobacillales|D_4__Streptococcaceae|D_5__Streptococcus"
## [2] "D_0__Bacteria|D_1__Bacteroidetes|D_2__Bacteroidia|D_3__Bacteroidales|D_4__Tannerellaceae|D_5__Parabacteroides"
## [3] "D_0__Bacteria|D_1__Bacteroidetes|D_2__Bacteroidia|D_3__Bacteroidales|D_4__Bacteroidaceae|D_5__Bacteroides"
## [4] "D_0__Bacteria|D_1__Proteobacteria|D_2__Gammaproteobacteria|D_3__Pseudomonadales|D_4__Pseudomonadaceae|D_5__Pseudomonas"
## [5] "D_0__Bacteria|D_1__Proteobacteria|D_2__Alphaproteobacteria|D_3__Caulobacterales|D_4__Caulobacteraceae|D_5__Brevundimonas"
## [6] "D_0__Bacteria|D_1__Firmicutes|D_2__Clostridia|D_3__Clostridiales|D_4__Ruminococcaceae|D_5__Butyricicoccus"
## [7] "D_0__Bacteria|D_1__Firmicutes|D_2__Clostridia|D_3__Clostridiales|D_4__Ruminococcaceae|D_5__Ruminococcus 1"
## [8] "D_0__Bacteria|D_1__Firmicutes|D_2__Clostridia|D_3__Clostridiales|D_4__Ruminococcaceae|D_5__Faecalibacterium"
## [9] "D_0__Bacteria|D_1__Firmicutes|D_2__Clostridia|D_3__Clostridiales|D_4__Lachnospiraceae|D_5__uncultured"

#this one is changed significantly by batch filtering - no Bogoriella, Pseudonocardia, Hydrotalea (likely contaminants)

#plot RCA by Genus
targeted_index <- IgA_simplewilcox_filt[,which(names(IgA_simplewilcox_filt) %in% most_targeted_RCA$Taxa)]
targeted_taxa <- IgTAX75_genus[which(IgTAX75_genus$combined %in% most_targeted_RCA$Taxa),]
targeted_taxa$Simple <- paste(targeted_taxa$Rank6)
targeted_taxa$Simple <- gsub(" coprostanoligenes group", "", targeted_taxa$Simple)
targeted_taxa$Simple <- gsub("D_5__", "", targeted_taxa$Simple)
targeted_taxa$Phylum <- gsub("D_1__", "", targeted_taxa$Rank2)

pdata1 <- data.frame(IgAIndex=targeted_index[,1], Taxa=targeted_taxa$Simple[1], Phylum=targeted_taxa$Phylum[1])
pdata2 <- data.frame(IgAIndex=targeted_index[,2], Taxa=targeted_taxa$Simple[2], Phylum=targeted_taxa$Phylum[2])
pdata3 <- data.frame(IgAIndex=targeted_index[,3], Taxa=targeted_taxa$Simple[3], Phylum=targeted_taxa$Phylum[3])
pdata4 <- data.frame(IgAIndex=targeted_index[,4], Taxa=targeted_taxa$Simple[4], Phylum=targeted_taxa$Phylum[4])
pdata5 <- data.frame(IgAIndex=targeted_index[,5], Taxa=targeted_taxa$Simple[5], Phylum=targeted_taxa$Phylum[5])
pdata6 <- data.frame(IgAIndex=targeted_index[,6], Taxa=targeted_taxa$Simple[6], Phylum=targeted_taxa$Phylum[6])
pdata7 <- data.frame(IgAIndex=targeted_index[,7], Taxa=targeted_taxa$Simple[7], Phylum=targeted_taxa$Phylum[7])

pdata <- Reduce(full_join, list(pdata1, pdata2, pdata3, pdata4, pdata5, pdata6, pdata7))

## Joining, by = c("IgAIndex", "Taxa", "Phylum")
## Joining, by = c("IgAIndex", "Taxa", "Phylum")
## Joining, by = c("IgAIndex", "Taxa", "Phylum")
## Joining, by = c("IgAIndex", "Taxa", "Phylum")
## Joining, by = c("IgAIndex", "Taxa", "Phylum")
## Joining, by = c("IgAIndex", "Taxa", "Phylum")

#plot - most targeted RCA - genus
p <- ggplot(pdata) + geom_jitter(aes(x=Taxa, y=IgAIndex, color=Phylum),
 width=0.2, height=0, shape=1, size=3)
p <- p + geom_boxplot(aes(x=Taxa, y=IgAIndex), colour="black", fill=NA, outlier.shape=NA)
p <- p + ggtitle("Most Targeted CAR")
p <- p + theme_bw(base_size=16)
p <- p + theme(axis.text.x = element_text(angle=45, hjust=1), axis.title.x = element_blank())
p <- p + ylim(c(-1, 1))
p <- p + scale_color_manual(values=c("darkgreen", "goldenrod3", "deepskyblue3", "indianred3"))
p


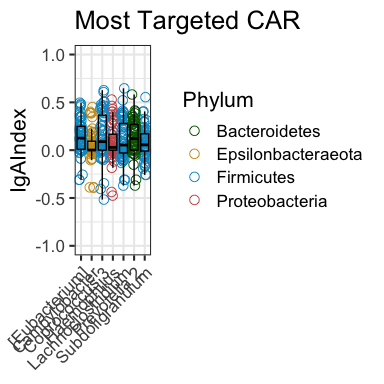


## Bubble plot of the main IgA-targeted taxa in full dataset, Mada, and CAR (genus level) - Fig 2A

###make a bubble plot of all targeted taxa with FDR<0.05
names(wilcox.dataGenus)[names(wilcox.dataGenus) == 'wilcox.dataGenus'] <- 'wilcox.data'
names(wilcox.dataGenus2)[names(wilcox.dataGenus2) == 'wilcox.dataGenus2'] <- 'wilcox.data'
names(wilcox.dataGenus3)[names(wilcox.dataGenus3) == 'wilcox.dataGenus3'] <- 'wilcox.data'
bubble.all <- Reduce(full_join, list(wilcox.dataGenus,wilcox.dataGenus2, wilcox.dataGenus3))

## Joining, by = c("wilcox.data", "rel.fdr", "mean", "median", "magnitude", "variance", "IQR", "Taxa", "SampleType")
## Joining, by = c("wilcox.data", "rel.fdr", "mean", "median", "magnitude", "variance", "IQR", "Taxa", "SampleType")

write.csv(bubble.all, "/Users/kelseyhuus/Dropbox/PhD/Afribiota/20-04-Revisions-Plots/TableS#_most_targeted_genera.csv")

#add a "p value category"
bubble.all$p.category <- bubble.all$wilcox.data
bubble.all$p.category[bubble.all$rel.fdr<0.05] <- "p<0.05"
bubble.all$p.category[bubble.all$rel.fdr<0.01] <- "p<0.01"
bubble.all$p.category[bubble.all$rel.fdr<0.001] <- "p<0.001"
bubble.all$p.category[bubble.all$rel.fdr<0.0001] <- "p<0.0001"
bubble.all$p.category[bubble.all$rel.fdr>=0.05] <- "NS"

#add a pos/neg category ####need to retain an actual average column as well as magnitude
bubble.all$posneg <- bubble.all$mean
bubble.all$posneg[bubble.all$mean>0] <- "pos"
bubble.all$posneg[bubble.all$mean<0] <- "neg"

#combine into 1 p value category
bubble.all$p.value.category <- paste(bubble.all$p.category, bubble.all$posneg, sep="")

#simplify by keeping only Taxa with at least one significance value.
bubble.simple <- filter(bubble.all, rel.fdr<0.05)
TaxaToKeep <- bubble.simple$Taxa
bubble.filter <- filter(bubble.all, Taxa %in% TaxaToKeep)
TaxaNames <- filter(IgTAX, IgTAX$combined %in% bubble.filter$Taxa)
TaxaNames$Simple <- gsub("D_5__", "", TaxaNames$Rank6)
TaxaNames$Simple <- ifelse(TaxaNames$Simple=="uncultured"|TaxaNames$Simple=="uncultured bacterium",
 yes = as.character(TaxaNames$Rank5), no=TaxaNames$Simple)
TaxaNames$Simple <- gsub("D_4__", "", TaxaNames$Simple)
TaxaNames$Simple <- ifelse(TaxaNames$Simple=="uncultured"|TaxaNames$Simple=="uncultured bacterium",
 yes = as.character(TaxaNames$Rank4), no=TaxaNames$Simple)
TaxaNames$Simple <- gsub("D_3__", "", TaxaNames$Simple)
TaxaNames$Simple <- gsub("Allorhizobium-Neorhizobium-Pararhizobium-Rhizobium", "Rhizobium", TaxaNames$Simple,
 fixed=TRUE)
TaxaNames$Taxa <- TaxaNames$combined
bubble.filter <- full_join(bubble.filter, TaxaNames, by="Taxa")

###bubble plot
bubble.filter$SampleType <- factor(bubble.filter$SampleType, levels = c("All", "Mada", "CAR"))
bubble.filter$p.value.category <- factor(bubble.filter$p.value.category, levels = c("p<0.0001neg", "p<0.001neg", "p<0.01neg", "p<0.05neg", "NSneg",
 "NS", "NSpos", "p<0.05pos", "p<0.01pos", "p<0.001pos", "p<0.0001pos"))

p <- ggplot(bubble.filter, aes(x=SampleType, y=Simple, size=magnitude)) + geom_point(shape=21, aes(fill=p.value.category), colour="grey30")
p <- p + scale_fill_manual(values=c("p<0.0001pos"="indianred4",
 "p<0.001pos"="indianred2",
 "p<0.01pos"="lightpink2",
 "p<0.05pos"="lightpink2",
 "NSpos"="ivory",
 "p<0.0001neg"="deepskyblue4",
 "p<0.001neg"="deepskyblue3",
 "p<0.01neg"="skyblue2",
 "p<0.05neg"="skyblue2",
 "NSneg"="aliceblue",
 "NS"="grey80"))
p <- p + theme(axis.text.x = element_text(angle=90, vjust=0.5, size=12), legend.key.size=unit(0.75, "cm"))
p <- p + scale_y_discrete(limits = rev(levels(bubble.filter$Taxa)))
p <- p + theme(strip.background = element_rect(fill="gray85"),
 panel.background = element_rect(fill="white"),
 panel.border = element_rect(colour="black", linetype="solid", fill="transparent")
)
p <- p + facet_grid(.~SampleType, scales = "free", space = "free")
p <- p + guides(colour=FALSE, size=FALSE, shape=FALSE, fill=FALSE)
p


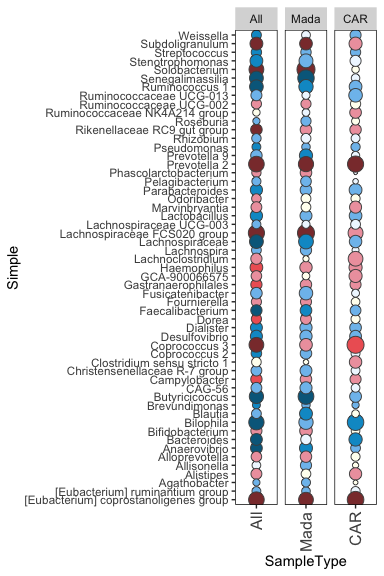


## Relative abundance data & correlations with IgA Index

# Read in relative abundance data and filter it using the same parameters as for the IgA sorting data

#read in new metadata for this 16S sequencing
meta16S <- read.table("/Users/kelseyhuus/Dropbox/PhD/Afribiota/Total16Sdata/sample_metadata_16S.csv",
 sep=";", header=TRUE)
#fecal samples
meta16S_F <- dplyr::filter(meta16S, SampleType=="feces")
#Reformat "ChildID" to match samples with
meta16S_F$ChildID <- meta16S_F$ID_afri
meta16S_F$ChildID <- gsub("SE_|AG_|AD_|SE-|AG-|AD-", "1429", meta16S_F$ChildID)
meta16S_F$ChildID <- gsub("CPB", "ACPB", meta16S_F$ChildID)

##read the 16S data
otu <- read.csv("/Users/kelseyhuus/Dropbox/PhD/Afribiota/Total16Sdata/OTU_table.csv",
 header=TRUE, row.names=1)
tax <- read.table("/Users/kelseyhuus/Dropbox/PhD/Afribiota/Total16Sdata/Taxonomy_table.txt",
 sep="\t", header=TRUE)

dim(meta16S_F)

## [1] 748 9

dim(otu)

## [1] 1423 6178

dim(tax)

## [1] 6178 8

#filter 16S tables to look only at the same children that have IgA Index
meta_16S_feces_filt <- meta16S_F[meta16S_F$ChildID%in%(sample_data(IgPos_forindex)$ChildID),]
dim(meta_16S_feces_filt)

## [1] 118 9

otu_mine <- otu[which(row.names(otu) %in% meta_16S_feces_filt$ID_metag),]
meta_16S_feces_filt <- meta_16S_feces_filt[which(meta_16S_feces_filt$ID_metag %in% row.names(otu_mine)), ]
row.names(meta_16S_feces_filt) <- meta_16S_feces_filt$ID_metag

#add IgPos metadata
metaIgA <- as.data.frame(sample_data(IgPos_forindex))
to_add <- metaIgA[metaIgA$ChildID %in% meta_16S_feces_filt$ChildID,]
to_add <- to_add[order(to_add$ChildID),]
meta_16S_feces_filt <- meta_16S_feces_filt[order(meta_16S_feces_filt$ChildID),]
to_add$ChildID==meta_16S_feces_filt$ChildID

## [1] TRUE TRUE TRUE TRUE TRUE TRUE TRUE TRUE TRUE TRUE TRUE TRUE TRUE TRUE TRUE
## [16] TRUE TRUE TRUE TRUE TRUE TRUE TRUE TRUE TRUE TRUE TRUE TRUE TRUE TRUE TRUE
## [31] TRUE TRUE TRUE TRUE TRUE TRUE TRUE TRUE TRUE TRUE TRUE TRUE TRUE TRUE TRUE
## [46] TRUE TRUE TRUE TRUE TRUE TRUE TRUE TRUE TRUE TRUE TRUE TRUE TRUE TRUE TRUE
## [61] TRUE TRUE TRUE TRUE TRUE TRUE TRUE TRUE TRUE TRUE TRUE TRUE TRUE TRUE TRUE
## [76] TRUE TRUE TRUE TRUE TRUE TRUE TRUE TRUE TRUE TRUE TRUE TRUE TRUE TRUE TRUE
## [91] TRUE TRUE TRUE TRUE TRUE TRUE TRUE TRUE TRUE TRUE TRUE TRUE TRUE TRUE TRUE
## [106] TRUE TRUE TRUE TRUE TRUE TRUE TRUE TRUE TRUE TRUE TRUE

meta_16S_feces_filt2 <- data.frame(meta_16S_feces_filt, to_add)

#phyloseq
otu_phyloseq <- as.data.frame(t(otu_mine))
otu_phyloseq <- otu_table(otu_phyloseq, taxa_are_rows = TRUE)
tax <- as.matrix(tax)
tax_phyloseq <- tax_table(tax)
meta_16S_feces_phyloseq <- sample_data(meta_16S_feces_filt2)

Fecal16S <- merge_phyloseq(otu_phyloseq,tax_phyloseq,meta_16S_feces_phyloseq)
Fecal16S

## phyloseq-class experiment-level object
## otu_table() OTU Table: [ 6177 taxa and 116 samples ]
## sample_data() Sample Data: [ 116 samples by 405 sample variables ]
## tax_table() Taxonomy Table: [ 6177 taxa by 8 taxonomic ranks ]

###filter using the same steps as applied to IgA sorted 16S data
#Prune out unwanted taxa / known contaminants
taxotu <- data.frame(OTU = row.names(otu_table(Fecal16S)), tax_table(Fecal16S))
halotus <- subset(taxotu, Rank1 == "Archaea" | Rank1 == "Eukaryota" |Rank6 == "Halomonas" | Rank6 == "Shewanella" | Rank5 == "Mitochondria" | Rank5 == "Chloroplast")
halotus <- as.character(halotus[["OTU"]])
Fecal16S_pruned1 <- prune_taxa(setdiff(row.names(otu_table(Fecal16S)), halotus), Fecal16S)
Fecal16S_pruned1 <- prune_taxa(taxa_sums(Fecal16S_pruned1) > 0, Fecal16S_pruned1)
Fecal16S_pruned1

## phyloseq-class experiment-level object
## otu_table() OTU Table: [ 1236 taxa and 116 samples ]
## sample_data() Sample Data: [ 116 samples by 405 sample variables ]
## tax_table() Taxonomy Table: [ 1236 taxa by 8 taxonomic ranks ]

##Prune out any samples with very low read counts
Fecal16S_pruned2 <- prune_samples(sample_sums(Fecal16S_pruned1)>=1000, Fecal16S_pruned1)
Fecal16S_pruned2

## phyloseq-class experiment-level object
## otu_table() OTU Table: [ 1236 taxa and 116 samples ]
## sample_data() Sample Data: [ 116 samples by 405 sample variables ]
## tax_table() Taxonomy Table: [ 1236 taxa by 8 taxonomic ranks ]

#remove taxa that are very low rel abund (less than 0.01%)
number <- taxa_sums(Fecal16S_pruned2)/sum(taxa_sums(Fecal16S_pruned2))
Fecal16S_pruned3 <- prune_taxa(number > 0.0001, Fecal16S_pruned2)
Fecal16S_pruned3

## phyloseq-class experiment-level object
## otu_table() OTU Table: [ 452 taxa and 116 samples ]
## sample_data() Sample Data: [ 116 samples by 405 sample variables ]
## tax_table() Taxonomy Table: [ 452 taxa by 8 taxonomic ranks ]

#save sampling depth information before rarefying
sample_data(Fecal16S_pruned3)$sample_sums <- sample_sums(Fecal16S_pruned3)

#rarefy to 5000 reads
set.seed(3)
Fecal16S_pruned4 <- rarefy_even_depth(Fecal16S_pruned3, sample.size = 5000)

## You set `rngseed` to FALSE. Make sure you've set & recorded
## the random seed of your session for reproducibility.
## See `?set.seed`

## ...

## 3 samples removedbecause they contained fewer reads than `sample.size`.

## Up to first five removed samples are:

## S003K-0492_S176S003K-0553_S237S003K-0583_S270

## ...

Fecal16S_pruned4

## phyloseq-class experiment-level object
## otu_table() OTU Table: [ 452 taxa and 113 samples ]
## sample_data() Sample Data: [ 113 samples by 405 sample variables ]
## tax_table() Taxonomy Table: [ 452 taxa by 8 taxonomic ranks ]

#how similar are the remaining samples between datasets
length(sample_data(Fecal16S_pruned4)$ChildID) #113

## [1] 113

length(sample_data(IgPos_forindex)$ChildID) #138

## [1] 138

#relative abundance
Fecal16S_relabund <- transform_sample_counts(Fecal16S_pruned4, function(OTU) OTU/sum(OTU))

#genus level
Fecal16S_genus <- tax_glom(Fecal16S_pruned4, "Rank6")
Fecal16S_genus <- transform_sample_counts(Fecal16S_genus, function(OTU) OTU/sum(OTU))

# Extract & clean relative abundance tables for genus and for ASV

##feces 16S at genus level
taxaf <- as.data.frame(Fecal16S_genus@tax_table@.Data)
taxaf$ASV <- row.names(taxaf)
#taxaf$Simple <- paste(taxaf$Rank6, taxaf$Rank7, substr(taxaf$ASV, start=4, stop=7), taxaf$Accession, sep="|")
taxaf$Simple <- paste(taxaf$Rank6, substr(taxaf$ASV, start=4, stop=7), sep="|")

otuf <- as.data.frame(otu_table(Fecal16S_genus))
otuf_t <- as.data.frame(t(otuf))

#confirm - these are the same order?
names(otuf_t) == taxaf$ASV #TRUE

## [1] TRUE TRUE TRUE TRUE TRUE TRUE TRUE TRUE TRUE TRUE TRUE TRUE TRUE TRUE TRUE
## [16] TRUE TRUE TRUE TRUE TRUE TRUE TRUE TRUE TRUE TRUE TRUE TRUE TRUE TRUE TRUE
## [31] TRUE TRUE TRUE TRUE TRUE TRUE TRUE TRUE TRUE TRUE TRUE TRUE TRUE TRUE TRUE
## [46] TRUE TRUE TRUE TRUE TRUE TRUE TRUE TRUE TRUE TRUE TRUE TRUE TRUE TRUE TRUE
## [61] TRUE TRUE TRUE TRUE TRUE TRUE TRUE TRUE TRUE TRUE TRUE TRUE TRUE TRUE TRUE
## [76] TRUE TRUE TRUE TRUE TRUE TRUE TRUE TRUE TRUE TRUE TRUE TRUE TRUE TRUE TRUE
## [91] TRUE TRUE TRUE TRUE TRUE TRUE TRUE TRUE TRUE TRUE TRUE TRUE TRUE TRUE TRUE
## [106] TRUE TRUE TRUE TRUE TRUE TRUE TRUE TRUE TRUE TRUE TRUE TRUE TRUE TRUE TRUE
## [121] TRUE TRUE TRUE TRUE TRUE TRUE TRUE TRUE TRUE TRUE TRUE TRUE TRUE TRUE TRUE
## [136] TRUE TRUE TRUE TRUE TRUE TRUE TRUE TRUE TRUE TRUE TRUE TRUE TRUE TRUE TRUE
## [151] TRUE

names(otuf_t) <- taxaf$Simple

metaf <- as.data.frame(sample_data(Fecal16S_genus))

dim(otuf_t) #163 x 476 (ASV) or x161 (genus)

## [1] 113 151

dim(metaf) #163 x401

## [1] 113 405

##feces 16S at ASV level
taxaf_asv <- as.data.frame(Fecal16S_relabund@tax_table@.Data)
taxaf_asv$ASV <- row.names(taxaf_asv)
taxaf_asv$Simple <- paste(taxaf_asv$Rank6, taxaf_asv$Rank7, taxaf_asv$Accession, sep="|")

otuf_asv <- as.data.frame(otu_table(Fecal16S_relabund))
otuf_asv_t <- as.data.frame(t(otuf_asv))

#confirm - these are the same order?
names(otuf_asv_t) == taxaf_asv$ASV #TRUE

## [1] TRUE TRUE TRUE TRUE TRUE TRUE TRUE TRUE TRUE TRUE TRUE TRUE TRUE TRUE TRUE
## [16] TRUE TRUE TRUE TRUE TRUE TRUE TRUE TRUE TRUE TRUE TRUE TRUE TRUE TRUE TRUE
## [31] TRUE TRUE TRUE TRUE TRUE TRUE TRUE TRUE TRUE TRUE TRUE TRUE TRUE TRUE TRUE
## [46] TRUE TRUE TRUE TRUE TRUE TRUE TRUE TRUE TRUE TRUE TRUE TRUE TRUE TRUE TRUE
## [61] TRUE TRUE TRUE TRUE TRUE TRUE TRUE TRUE TRUE TRUE TRUE TRUE TRUE TRUE TRUE
## [76] TRUE TRUE TRUE TRUE TRUE TRUE TRUE TRUE TRUE TRUE TRUE TRUE TRUE TRUE TRUE
## [91] TRUE TRUE TRUE TRUE TRUE TRUE TRUE TRUE TRUE TRUE TRUE TRUE TRUE TRUE TRUE
## [106] TRUE TRUE TRUE TRUE TRUE TRUE TRUE TRUE TRUE TRUE TRUE TRUE TRUE TRUE TRUE
## [121] TRUE TRUE TRUE TRUE TRUE TRUE TRUE TRUE TRUE TRUE TRUE TRUE TRUE TRUE TRUE
## [136] TRUE TRUE TRUE TRUE TRUE TRUE TRUE TRUE TRUE TRUE TRUE TRUE TRUE TRUE TRUE
## [151] TRUE TRUE TRUE TRUE TRUE TRUE TRUE TRUE TRUE TRUE TRUE TRUE TRUE TRUE TRUE
## [166] TRUE TRUE TRUE TRUE TRUE TRUE TRUE TRUE TRUE TRUE TRUE TRUE TRUE TRUE TRUE
## [181] TRUE TRUE TRUE TRUE TRUE TRUE TRUE TRUE TRUE TRUE TRUE TRUE TRUE TRUE TRUE
## [196] TRUE TRUE TRUE TRUE TRUE TRUE TRUE TRUE TRUE TRUE TRUE TRUE TRUE TRUE TRUE
## [211] TRUE TRUE TRUE TRUE TRUE TRUE TRUE TRUE TRUE TRUE TRUE TRUE TRUE TRUE TRUE
## [226] TRUE TRUE TRUE TRUE TRUE TRUE TRUE TRUE TRUE TRUE TRUE TRUE TRUE TRUE TRUE
## [241] TRUE TRUE TRUE TRUE TRUE TRUE TRUE TRUE TRUE TRUE TRUE TRUE TRUE TRUE TRUE
## [256] TRUE TRUE TRUE TRUE TRUE TRUE TRUE TRUE TRUE TRUE TRUE TRUE TRUE TRUE TRUE
## [271] TRUE TRUE TRUE TRUE TRUE TRUE TRUE TRUE TRUE TRUE TRUE TRUE TRUE TRUE TRUE
## [286] TRUE TRUE TRUE TRUE TRUE TRUE TRUE TRUE TRUE TRUE TRUE TRUE TRUE TRUE TRUE
## [301] TRUE TRUE TRUE TRUE TRUE TRUE TRUE TRUE TRUE TRUE TRUE TRUE TRUE TRUE TRUE
## [316] TRUE TRUE TRUE TRUE TRUE TRUE TRUE TRUE TRUE TRUE TRUE TRUE TRUE TRUE TRUE
## [331] TRUE TRUE TRUE TRUE TRUE TRUE TRUE TRUE TRUE TRUE TRUE TRUE TRUE TRUE TRUE
## [346] TRUE TRUE TRUE TRUE TRUE TRUE TRUE TRUE TRUE TRUE TRUE TRUE TRUE TRUE TRUE
## [361] TRUE TRUE TRUE TRUE TRUE TRUE TRUE TRUE TRUE TRUE TRUE TRUE TRUE TRUE TRUE
## [376] TRUE TRUE TRUE TRUE TRUE TRUE TRUE TRUE TRUE TRUE TRUE TRUE TRUE TRUE TRUE
## [391] TRUE TRUE TRUE TRUE TRUE TRUE TRUE TRUE TRUE TRUE TRUE TRUE TRUE TRUE TRUE
## [406] TRUE TRUE TRUE TRUE TRUE TRUE TRUE TRUE TRUE TRUE TRUE TRUE TRUE TRUE TRUE
## [421] TRUE TRUE TRUE TRUE TRUE TRUE TRUE TRUE TRUE TRUE TRUE TRUE TRUE TRUE TRUE
## [436] TRUE TRUE TRUE TRUE TRUE TRUE TRUE TRUE TRUE TRUE TRUE TRUE TRUE TRUE TRUE
## [451] TRUE TRUE

#replace names
names(otuf_asv_t) <- taxaf_asv$Simple

metaf_asv <- as.data.frame(sample_data(Fecal16S_relabund))

# Correlations between relative abundance and IgA Index for most- and least-targeted genera

#note: the following were tested one by one to allow the genera to be manually matched between these 2 datasets
#p values were saved and FDR corrected at the end
df <- data.frame(pvals=numeric(), coeffs=numeric(), taxa=factor())

#Haemophilus
taxon_IgA <- IgA_batchtrim75_genus_t$`D_0__Bacteria|D_1__Proteobacteria|D_2__Gammaproteobacteria|D_3__Pasteurellales|D_4__Pasteurellaceae|D_5__Haemophilus`
taxon_IgA <- data.frame(taxon_IgA=taxon_IgA, ChildID=row.names(IgA_batchtrim75_genus_t))
taxon_16S_fecal <- otuf_t$`Haemophilus|GGAG`
taxon_16S_fecal <- data.frame(taxon_16S_fecal=taxon_16S_fecal, ChildID=metaf$ChildID)
taxon_compare <- full_join(taxon_IgA, taxon_16S_fecal)
dim(taxon_compare) #188 x 3

## [1] 138 3

t <- cor.test(taxon_compare$taxon_IgA, taxon_compare$taxon_16S_fecal, method="spearman")
df[1,1] <- t$p.value
df[1,2] <- t$estimate
taxa <- c("Haemophilus")

#Campylobacter
taxon_IgA <- IgA_batchtrim75_genus_t$`D_0__Bacteria|D_1__Epsilonbacteraeota|D_2__Campylobacteria|D_3__Campylobacterales|D_4__Campylobacteraceae|D_5__Campylobacter`
taxon_IgA <- data.frame(taxon_IgA=taxon_IgA, ChildID=row.names(IgA_batchtrim75_genus_t))
taxon_16S_fecal <- otuf_t$`Campylobacter|GGAG`
taxon_16S_fecal <- data.frame(taxon_16S_fecal=taxon_16S_fecal, ChildID=metaf$ChildID)
taxon_compare <- full_join(taxon_IgA, taxon_16S_fecal)
t <- cor.test(taxon_compare$taxon_IgA, taxon_compare$taxon_16S_fecal, method="spearman")
df[2,1] <- t$p.value
df[2,2] <- t$estimate
taxa <- c(taxa, "Campylobacter")

#Eubacterium copro
taxon_IgA <- IgA_batchtrim75_genus_t$`D_0__Bacteria|D_1__Firmicutes|D_2__Clostridia|D_3__Clostridiales|D_4__Ruminococcaceae|D_5__[Eubacterium] coprostanoligenes group`
taxon_IgA <- data.frame(taxon_IgA=taxon_IgA, ChildID=row.names(IgA_batchtrim75_genus_t))
taxon_16S_fecal <- otuf_t$`[Eubacterium]_coprostanoligenes_group|GTAG`
taxon_16S_fecal <- data.frame(taxon_16S_fecal=taxon_16S_fecal, ChildID=metaf$ChildID)
taxon_compare <- full_join(taxon_IgA, taxon_16S_fecal)
t <- cor.test(taxon_compare$taxon_IgA, taxon_compare$taxon_16S_fecal, method="spearman")
df[3,1] <- t$p.value
df[3,2] <- t$estimate
taxa <- c(taxa, "Eubacterium")

#Coprococcus 3
taxon_IgA <- IgA_batchtrim75_genus_t$`D_0__Bacteria|D_1__Firmicutes|D_2__Clostridia|D_3__Clostridiales|D_4__Lachnospiraceae|D_5__Coprococcus 3`
taxon_IgA <- data.frame(taxon_IgA=taxon_IgA, ChildID=row.names(IgA_batchtrim75_genus_t))
taxon_16S_fecal <- otuf_t$`Coprococcus_3|GTAT`
taxon_16S_fecal <- data.frame(taxon_16S_fecal=taxon_16S_fecal, ChildID=metaf$ChildID)
taxon_compare <- full_join(taxon_IgA, taxon_16S_fecal)
t <- cor.test(taxon_compare$taxon_IgA, taxon_compare$taxon_16S_fecal, method="spearman")
df[4,1] <- t$p.value
df[4,2] <- t$estimate
taxa <- c(taxa, "Coprococcus 3")

#Dorea
taxon_IgA <- IgA_batchtrim75_genus_t$`D_0__Bacteria|D_1__Firmicutes|D_2__Clostridia|D_3__Clostridiales|D_4__Lachnospiraceae|D_5__Dorea`
taxon_IgA <- data.frame(taxon_IgA=taxon_IgA, ChildID=row.names(IgA_batchtrim75_genus_t))
taxon_16S_fecal <- otuf_t$`Dorea|GTAG`
taxon_16S_fecal <- data.frame(taxon_16S_fecal=taxon_16S_fecal, ChildID=metaf$ChildID)
taxon_compare <- full_join(taxon_IgA, taxon_16S_fecal)
t <- cor.test(taxon_compare$taxon_IgA, taxon_compare$taxon_16S_fecal, method="spearman")
df[5,1] <- t$p.value
df[5,2] <- t$estimate
taxa <- c(taxa, "Dorea")

#Lachnoclostridium
taxon_IgA <- IgA_batchtrim75_genus_t$`D_0__Bacteria|D_1__Firmicutes|D_2__Clostridia|D_3__Clostridiales|D_4__Lachnospiraceae|D_5__Lachnoclostridium`
taxon_IgA <- data.frame(taxon_IgA=taxon_IgA, ChildID=row.names(IgA_batchtrim75_genus_t))
taxon_16S_fecal <- otuf_t$`Lachnoclostridium|GTAG`
taxon_16S_fecal <- data.frame(taxon_16S_fecal=taxon_16S_fecal, ChildID=metaf$ChildID)
taxon_compare <- full_join(taxon_IgA, taxon_16S_fecal)
t <- cor.test(taxon_compare$taxon_IgA, taxon_compare$taxon_16S_fecal, method="spearman")
df[6,1] <- t$p.value
df[6,2] <- t$estimate
taxa <- c(taxa, "Lachnoclostridium")

#Prevotella 2
taxon_IgA <- IgA_batchtrim75_genus_t$`D_0__Bacteria|D_1__Bacteroidetes|D_2__Bacteroidia|D_3__Bacteroidales|D_4__Prevotellaceae|D_5__Prevotella 2`
taxon_IgA <- data.frame(taxon_IgA=taxon_IgA, ChildID=row.names(IgA_batchtrim75_genus_t))
taxon_16S_fecal <- otuf_t$`Prevotella_2|GGAA`
taxon_16S_fecal <- data.frame(taxon_16S_fecal=taxon_16S_fecal, ChildID=metaf$ChildID)
taxon_compare <- full_join(taxon_IgA, taxon_16S_fecal)
t <- cor.test(taxon_compare$taxon_IgA, taxon_compare$taxon_16S_fecal, method="spearman")
df[7,1] <- t$p.value
df[7,2] <- t$estimate
taxa <- c(taxa, "Prevotella 2")

#Ruminococcacea UCG-002
taxon_IgA <- IgA_batchtrim75_genus_t$`D_0__Bacteria|D_1__Firmicutes|D_2__Clostridia|D_3__Clostridiales|D_4__Ruminococcaceae|D_5__Ruminococcaceae UCG-002`
taxon_IgA <- data.frame(taxon_IgA=taxon_IgA, ChildID=row.names(IgA_batchtrim75_genus_t))
taxon_16S_fecal <- otuf_t$`Ruminococcaceae_UCG-002|GTAG`
taxon_16S_fecal <- data.frame(taxon_16S_fecal=taxon_16S_fecal, ChildID=metaf$ChildID)
taxon_compare <- full_join(taxon_IgA, taxon_16S_fecal)
t <- cor.test(taxon_compare$taxon_IgA, taxon_compare$taxon_16S_fecal, method="spearman")
df[8,1] <- t$p.value
df[8,2] <- t$estimate
taxa <- c(taxa, "Ruminococcaceae UCG-002")

#Subdoligranulum
taxon_IgA <- IgA_batchtrim75_genus_t$`D_0__Bacteria|D_1__Firmicutes|D_2__Clostridia|D_3__Clostridiales|D_4__Ruminococcaceae|D_5__Subdoligranulum`
taxon_IgA <- data.frame(taxon_IgA=taxon_IgA, ChildID=row.names(IgA_batchtrim75_genus_t))
taxon_16S_fecal <- otuf_t$`Subdoligranulum|GTAG`
taxon_16S_fecal <- data.frame(taxon_16S_fecal=taxon_16S_fecal, ChildID=metaf$ChildID)
taxon_compare <- full_join(taxon_IgA, taxon_16S_fecal)
t <- cor.test(taxon_compare$taxon_IgA, taxon_compare$taxon_16S_fecal, method="spearman")
df[9,1] <- t$p.value
df[9,2] <- t$estimate
taxa <- c(taxa, "Subdoligranulum")

#Solobacterium
taxon_IgA <- IgA_batchtrim75_genus_t$`D_0__Bacteria|D_1__Firmicutes|D_2__Erysipelotrichia|D_3__Erysipelotrichales|D_4__Erysipelotrichaceae|D_5__Solobacterium`
taxon_IgA <- data.frame(taxon_IgA=taxon_IgA, ChildID=row.names(IgA_batchtrim75_genus_t))
taxon_16S_fecal <- otuf_t$`Solobacterium|GTAG`
taxon_16S_fecal <- data.frame(taxon_16S_fecal=taxon_16S_fecal, ChildID=metaf$ChildID)
taxon_compare <- full_join(taxon_IgA, taxon_16S_fecal)
t <- cor.test(taxon_compare$taxon_IgA, taxon_compare$taxon_16S_fecal, method="spearman")
df[10,1] <- t$p.value
df[10,2] <- t$estimate
taxa <- c(taxa, "Solobacterium")

###un-targeted taxa associations
#Streptococcus
taxon_IgA <- IgA_batchtrim75_genus_t$`D_0__Bacteria|D_1__Firmicutes|D_2__Bacilli|D_3__Lactobacillales|D_4__Streptococcaceae|D_5__Streptococcus`
taxon_IgA <- data.frame(taxon_IgA=taxon_IgA, ChildID=row.names(IgA_batchtrim75_genus_t))
taxon_16S_fecal <- otuf_t$`Streptococcus|GTAG`
taxon_16S_fecal <- data.frame(taxon_16S_fecal=taxon_16S_fecal, ChildID=metaf$ChildID)
taxon_compare <- full_join(taxon_IgA, taxon_16S_fecal)
t <- cor.test(taxon_compare$taxon_IgA, taxon_compare$taxon_16S_fecal, method="spearman")
df[11,1] <- t$p.value
df[11,2] <- t$estimate
taxa <- c(taxa, "Streptococcus")

#Dialister
taxon_IgA <- IgA_batchtrim75_genus_t$`D_0__Bacteria|D_1__Firmicutes|D_2__Negativicutes|D_3__Selenomonadales|D_4__Veillonellaceae|D_5__Dialister`
taxon_IgA <- data.frame(taxon_IgA=taxon_IgA, ChildID=row.names(IgA_batchtrim75_genus_t))
taxon_16S_fecal <- otuf_t$`Dialister|GTAG`
taxon_16S_fecal <- data.frame(taxon_16S_fecal=taxon_16S_fecal, ChildID=metaf$ChildID)
taxon_compare <- full_join(taxon_IgA, taxon_16S_fecal)
t <- cor.test(taxon_compare$taxon_IgA, taxon_compare$taxon_16S_fecal, method="spearman")
df[12,1] <- t$p.value
df[12,2] <- t$estimate
taxa <- c(taxa, "Dialister")

#Parabacteroides
taxon_IgA <- IgA_batchtrim75_genus_t$`D_0__Bacteria|D_1__Bacteroidetes|D_2__Bacteroidia|D_3__Bacteroidales|D_4__Tannerellaceae|D_5__Parabacteroides`
taxon_IgA <- data.frame(taxon_IgA=taxon_IgA, ChildID=row.names(IgA_batchtrim75_genus_t))
taxon_16S_fecal <- otuf_t$`Parabacteroides|GGAG`
taxon_16S_fecal <- data.frame(taxon_16S_fecal=taxon_16S_fecal, ChildID=metaf$ChildID)
taxon_compare <- full_join(taxon_IgA, taxon_16S_fecal)
t <- cor.test(taxon_compare$taxon_IgA, taxon_compare$taxon_16S_fecal, method="spearman")
df[13,1] <- t$p.value
df[13,2] <- t$estimate
taxa <- c(taxa, "Parabacteroides")

#Bacteroides
taxon_IgA <- IgA_batchtrim75_genus_t$`D_0__Bacteria|D_1__Bacteroidetes|D_2__Bacteroidia|D_3__Bacteroidales|D_4__Bacteroidaceae|D_5__Bacteroides`
taxon_IgA <- data.frame(taxon_IgA=taxon_IgA, ChildID=row.names(IgA_batchtrim75_genus_t))
taxon_16S_fecal <- otuf_t$`Bacteroides|GGAG`
taxon_16S_fecal <- data.frame(taxon_16S_fecal=taxon_16S_fecal, ChildID=metaf$ChildID)
taxon_compare <- full_join(taxon_IgA, taxon_16S_fecal)
t <- cor.test(taxon_compare$taxon_IgA, taxon_compare$taxon_16S_fecal, method="spearman")
df[14,1] <- t$p.value
df[14,2] <- t$estimate
taxa <- c(taxa, "Bacteroides")

#Pseudomonas
taxon_IgA <- IgA_batchtrim75_genus_t$`D_0__Bacteria|D_1__Proteobacteria|D_2__Gammaproteobacteria|D_3__Pseudomonadales|D_4__Pseudomonadaceae|D_5__Pseudomonas`
taxon_IgA <- data.frame(taxon_IgA=taxon_IgA, ChildID=row.names(IgA_batchtrim75_genus_t))
taxon_16S_fecal <- otuf_t$`Pseudomonas|GAAG`
taxon_16S_fecal <- data.frame(taxon_16S_fecal=taxon_16S_fecal, ChildID=metaf$ChildID)
taxon_compare <- full_join(taxon_IgA, taxon_16S_fecal)
t <- cor.test(taxon_compare$taxon_IgA, taxon_compare$taxon_16S_fecal, method="spearman")
df[15,1] <- t$p.value
df[15,2] <- t$estimate
taxa <- c(taxa, "Pseudomonas")

#Stenotrophomonas
taxon_IgA <- IgA_batchtrim75_genus_t$`D_0__Bacteria|D_1__Proteobacteria|D_2__Gammaproteobacteria|D_3__Xanthomonadales|D_4__Xanthomonadaceae|D_5__Stenotrophomonas`
taxon_IgA <- data.frame(taxon_IgA=taxon_IgA, ChildID=row.names(IgA_batchtrim75_genus_t))
taxon_16S_fecal <- otuf_t$`Stenotrophomonas|GAAG`
taxon_16S_fecal <- data.frame(taxon_16S_fecal=taxon_16S_fecal, ChildID=metaf$ChildID)
taxon_compare <- full_join(taxon_IgA, taxon_16S_fecal)
t <- cor.test(taxon_compare$taxon_IgA, taxon_compare$taxon_16S_fecal, method="spearman")
df[16,1] <- t$p.value
df[16,2] <- t$estimate
taxa <- c(taxa, "Stenotrophomonas")

#Brevundimonas / Pelagibacterium / Allorhizobium
#not present in total 16S - likely contaminants

#Roseburia
taxon_IgA <- IgA_batchtrim75_genus_t$`D_0__Bacteria|D_1__Firmicutes|D_2__Clostridia|D_3__Clostridiales|D_4__Lachnospiraceae|D_5__Roseburia`
taxon_IgA <- data.frame(taxon_IgA=taxon_IgA, ChildID=row.names(IgA_batchtrim75_genus_t))
taxon_16S_fecal <- otuf_t$`Roseburia|GTAT`
taxon_16S_fecal <- data.frame(taxon_16S_fecal=taxon_16S_fecal, ChildID=metaf$ChildID)
taxon_compare <- full_join(taxon_IgA, taxon_16S_fecal)
t <- cor.test(taxon_compare$taxon_IgA, taxon_compare$taxon_16S_fecal, method="spearman")
df[17,1] <- t$p.value
df[17,2] <- t$estimate
taxa <- c(taxa, "Roseburia")

#Agathabacter
#not present in total 16S

#Butyricoccus
taxon_IgA <- IgA_batchtrim75_genus_t$`D_0__Bacteria|D_1__Firmicutes|D_2__Clostridia|D_3__Clostridiales|D_4__Ruminococcaceae|D_5__Butyricicoccus`
taxon_IgA <- data.frame(taxon_IgA=taxon_IgA, ChildID=row.names(IgA_batchtrim75_genus_t))
taxon_16S_fecal <- otuf_t$`Butyricicoccus|GTAG`
taxon_16S_fecal <- data.frame(taxon_16S_fecal=taxon_16S_fecal, ChildID=metaf$ChildID)
taxon_compare <- full_join(taxon_IgA, taxon_16S_fecal)
t <- cor.test(taxon_compare$taxon_IgA, taxon_compare$taxon_16S_fecal, method="spearman")
df[18,1] <- t$p.value
df[18,2] <- t$estimate
taxa <- c(taxa, "Butyricoccus")

#Faecalibacterium
taxon_IgA <- IgA_batchtrim75_genus_t$`D_0__Bacteria|D_1__Firmicutes|D_2__Clostridia|D_3__Clostridiales|D_4__Ruminococcaceae|D_5__Faecalibacterium`
taxon_IgA <- data.frame(taxon_IgA=taxon_IgA, ChildID=row.names(IgA_batchtrim75_genus_t))
taxon_16S_fecal <- otuf_t$`Faecalibacterium|GTAG`
taxon_16S_fecal <- data.frame(taxon_16S_fecal=taxon_16S_fecal, ChildID=metaf$ChildID)
taxon_compare <- full_join(taxon_IgA, taxon_16S_fecal)
t <- cor.test(taxon_compare$taxon_IgA, taxon_compare$taxon_16S_fecal, method="spearman")
df[19,1] <- t$p.value
df[19,2] <- t$estimate
taxa <- c(taxa, "Faecalibacterium")

#Blautia
taxon_IgA <- IgA_batchtrim75_genus_t$`D_0__Bacteria|D_1__Firmicutes|D_2__Clostridia|D_3__Clostridiales|D_4__Lachnospiraceae|D_5__Blautia`
taxon_IgA <- data.frame(taxon_IgA=taxon_IgA, ChildID=row.names(IgA_batchtrim75_genus_t))
taxon_16S_fecal <- otuf_t$`Blautia|GTAG`
taxon_16S_fecal <- data.frame(taxon_16S_fecal=taxon_16S_fecal, ChildID=metaf$ChildID)
taxon_compare <- full_join(taxon_IgA, taxon_16S_fecal)
t <- cor.test(taxon_compare$taxon_IgA, taxon_compare$taxon_16S_fecal, method="spearman")
df[20,1] <- t$p.value
df[20,2] <- t$estimate
taxa <- c(taxa, "Blautia")

#Lachnospiraceae uncultured
#impossible to match at genus level

#Prevotella 9
taxon_IgA <- IgA_batchtrim75_genus_t$`D_0__Bacteria|D_1__Bacteroidetes|D_2__Bacteroidia|D_3__Bacteroidales|D_4__Prevotellaceae|D_5__Prevotella 9`
taxon_IgA <- data.frame(taxon_IgA=taxon_IgA, ChildID=row.names(IgA_batchtrim75_genus_t))
taxon_16S_fecal <- otuf_t$`Prevotella_9|GGAA`
taxon_16S_fecal <- data.frame(taxon_16S_fecal=taxon_16S_fecal, ChildID=metaf$ChildID)
taxon_compare <- full_join(taxon_IgA, taxon_16S_fecal)
t <- cor.test(taxon_compare$taxon_IgA, taxon_compare$taxon_16S_fecal, method="spearman")
df[21,1] <- t$p.value
df[21,2] <- t$estimate
taxa <- c(taxa, "Prevotella 9")

#Christensenellaceae R-7
taxon_IgA <- IgA_batchtrim75_genus_t$`D_0__Bacteria|D_1__Firmicutes|D_2__Clostridia|D_3__Clostridiales|D_4__Christensenellaceae|D_5__Christensenellaceae R-7 group`
taxon_IgA <- data.frame(taxon_IgA=taxon_IgA, ChildID=row.names(IgA_batchtrim75_genus_t))
taxon_16S_fecal <- otuf_t$`Christensenellaceae_R-7_group|GTAG`
taxon_16S_fecal <- data.frame(taxon_16S_fecal=taxon_16S_fecal, ChildID=metaf$ChildID)
taxon_compare <- full_join(taxon_IgA, taxon_16S_fecal)
t <- cor.test(taxon_compare$taxon_IgA, taxon_compare$taxon_16S_fecal, method="spearman")
df[22,1] <- t$p.value
df[22,2] <- t$estimate
taxa <- c(taxa, "Christensenellaceae R-7")

#Ruminococcus 1
taxon_IgA <- IgA_batchtrim75_genus_t$`D_0__Bacteria|D_1__Firmicutes|D_2__Clostridia|D_3__Clostridiales|D_4__Ruminococcaceae|D_5__Ruminococcus 1`
taxon_IgA <- data.frame(taxon_IgA=taxon_IgA, ChildID=row.names(IgA_batchtrim75_genus_t))
taxon_16S_fecal <- otuf_t$`Ruminococcus_1|GTAG`
taxon_16S_fecal <- data.frame(taxon_16S_fecal=taxon_16S_fecal, ChildID=metaf$ChildID)
taxon_compare <- full_join(taxon_IgA, taxon_16S_fecal)
t <- cor.test(taxon_compare$taxon_IgA, taxon_compare$taxon_16S_fecal, method="spearman")
df[23,1] <- t$p.value
df[23,2] <- t$estimate
taxa <- c(taxa, "Ruminococcus 1")

#include taxa
df$taxa <- taxa

#fdr correct
df$fdr <- p.adjust(df$pvals, method="fdr")
df[which(df$fdr<0.05),]

## pvals coeffs taxa fdr
## 19 0.0001387144 -0.3508914 Faecalibacterium 0.003190431

#faecalibacteirum does pass multiple corrections

# Plot the spearman coefficients (genus level) in a heatmap - Fig S6A

p <- ggplot(df) + geom_tile(aes(x=1, y=taxa, fill=coeffs))
p <- p + scale_fill_gradient2(low="lightskyblue3", mid="white", high="indianred", midpoint=0)
p <- p + xlab(NULL) + ylab(NULL)
p


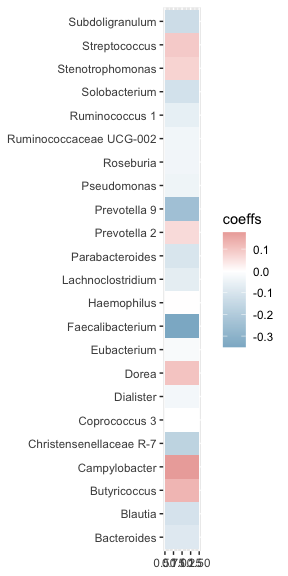


## Putative pathobionts: Fig S6-S7

# Campylobacter and Haemophilus IgA index by stunting, batch - Fig S6B-E

###IgA Index
metaIgA_genus <- as.data.frame(sample_data(IgPos_forindexGen))
metaIgA <- as.data.frame(sample_data(IgPos_forindex))

#Campylobacter
pdata <- data.frame(taxa=IgA_batchtrim75_genus_t$`D_0__Bacteria|D_1__Epsilonbacteraeota|D_2__Campylobacteria|D_3__Campylobacterales|D_4__Campylobacteraceae|D_5__Campylobacter`,
 Stunting=metaIgA_genus$stunt_categ2,
 Country=metaIgA_genus$Country2,
 Batch=metaIgA_genus$Batch)
wilcox.test(pdata$taxa~pdata$Stunting)

##
## Wilcoxon rank sum test with continuity correction
##
## data: pdata$taxa by pdata$Stunting
## W = 2193.5, p-value = 0.4075
## alternative hypothesis: true location shift is not equal to 0

pdata_CAR <- pdata[pdata$Country=="CAR",]
pdata_Mada <- pdata[pdata$Country=="Mada",]
wilcox.test(pdata_CAR$taxa~pdata_CAR$Stunting)

##
## Wilcoxon rank sum test with continuity correction
##
## data: pdata_CAR$taxa by pdata_CAR$Stunting
## W = 381, p-value = 0.3178
## alternative hypothesis: true location shift is not equal to 0

wilcox.test(pdata_Mada$taxa~pdata_Mada$Stunting)

##
## Wilcoxon rank sum test with continuity correction
##
## data: pdata_Mada$taxa by pdata_Mada$Stunting
## W = 725.5, p-value = 0.7652
## alternative hypothesis: true location shift is not equal to 0

p <- ggplot(pdata) + geom_boxplot(aes(x=Country, y=taxa, fill=Stunting), outlier.shape=1, outlier.size=3)
p <- p + ggtitle("Campylobacter")
p <- p + theme_bw(base_size=16)
p <- p + theme(plot.title=element_text(size=15))
p <- p + ylab("IgA Index") + xlab(NULL)
p <- p + scale_fill_manual(values=c("white", "indianred3"))
p <- p + ylim(-1, 1)
p <- p + annotate("text", x = 1, y=0.8, label = "p=0.32", size=4)
p <- p + annotate("segment", x=0.75, xend=1.25, y=0.7, yend=0.7)
p <- p + annotate("text", x = 2, y=0.8, label = "p=0.76", size=4)
p <- p + annotate("segment", x=1.75, xend=2.25, y=0.7, yend=0.7)
p


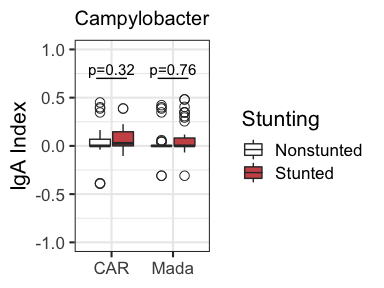


#Campylobacter IgA Index by batch
wilcox.test(pdata$taxa~pdata$Batch) #p=0.172

##
## Wilcoxon rank sum test with continuity correction
##
## data: pdata$taxa by pdata$Batch
## W = 1956, p-value = 0.172
## alternative hypothesis: true location shift is not equal to 0

pdata_Batch1 <- pdata[pdata$Batch=="Batch1",]
pdata_Batch2 <- pdata[pdata$Batch=="Batch2",]
wilcox.test(pdata_Batch1$taxa~pdata_Batch1$Stunting) #p=0.9539

##
## Wilcoxon rank sum test with continuity correction
##
## data: pdata_Batch1$taxa by pdata_Batch1$Stunting
## W = 347.5, p-value = 0.9539
## alternative hypothesis: true location shift is not equal to 0

median(pdata_Batch1$taxa) #0

## [1] 0

mean(pdata_Batch1$taxa) #0.02

## [1] 0.02451444

wilcox.test(pdata_Batch2$taxa~pdata_Batch2$Stunting) #p=0.339

##
## Wilcoxon rank sum test with continuity correction
##
## data: pdata_Batch2$taxa by pdata_Batch2$Stunting
## W = 797, p-value = 0.339
## alternative hypothesis: true location shift is not equal to 0

median(pdata_Batch2$taxa) #0

## [1] 0

mean(pdata_Batch2$taxa) #0.06

## [1] 0.06904736

p <- ggplot(pdata) + geom_boxplot(aes(x=Batch, y=taxa), outlier.shape=1, outlier.size=3, fill="gray70")
p <- p + ggtitle("Campylobacter")
p <- p + theme_bw(base_size=16)
p <- p + theme(plot.title = element_text(size=15))
p <- p + ylab("IgA Index") + xlab(NULL)
p <- p + ylim(-1, 1)
p <- p + annotate("text", x = 1.5, y=0.8, label = "p=0.17", size=4)
p <- p + annotate("segment", x=1, xend=2, y=0.7, yend=0.7)
p


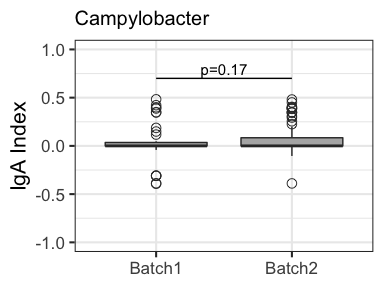


#Haemophilus (ASV)
pdata <- data.frame(taxa=IgA_batchtrim75_t$`D_0__Bacteria|D_1__Proteobacteria|D_2__Gammaproteobacteria|D_3__Pasteurellales|D_4__Pasteurellaceae|D_5__Haemophilus|NA|e27680d4009f98f30248d823bc17fb8e`,
 Stunting=metaIgA$stunt_categ2,
 Country = metaIgA$Country2,
 Batch=metaIgA$Batch)
wilcox.test(pdata$taxa~pdata$Stunting)

##
## Wilcoxon rank sum test with continuity correction
##
## data: pdata$taxa by pdata$Stunting
## W = 2300, p-value = 0.739
## alternative hypothesis: true location shift is not equal to 0

pdata_CAR <- pdata[pdata$Country=="CAR",]
pdata_Mada <- pdata[pdata$Country=="Mada",]
wilcox.test(pdata_CAR$taxa~pdata_CAR$Stunting)

##
## Wilcoxon rank sum test with continuity correction
##
## data: pdata_CAR$taxa by pdata_CAR$Stunting
## W = 453.5, p-value = 0.9408
## alternative hypothesis: true location shift is not equal to 0

wilcox.test(pdata_Mada$taxa~pdata_Mada$Stunting)

##
## Wilcoxon rank sum test with continuity correction
##
## data: pdata_Mada$taxa by pdata_Mada$Stunting
## W = 691.5, p-value = 0.5421
## alternative hypothesis: true location shift is not equal to 0

p <- ggplot(pdata) + geom_boxplot(aes(x=Country, y=taxa, fill=Stunting), outlier.shape=1, outlier.size=3)
p <- p + ggtitle("Haemophilus")
p <- p + theme_bw(base_size=16)
p <- p + theme(plot.title = element_text(size=14))
p <- p + ylab("IgA Index") + xlab(NULL)
p <- p + scale_fill_manual(values=c("white", "indianred3"))
p <- p + ylim(-1, 1)
p <- p + annotate("text", x = 1, y=0.8, label = "p=0.94", size=4)
p <- p + annotate("segment", x=0.7, xend=1.25, y=0.7, yend=0.7)
p <- p + annotate("text", x = 2, y=0.8, label = "p=0.54", size=4)
p <- p + annotate("segment", x=1.75, xend=2.25, y=0.7, yend=0.7)
p


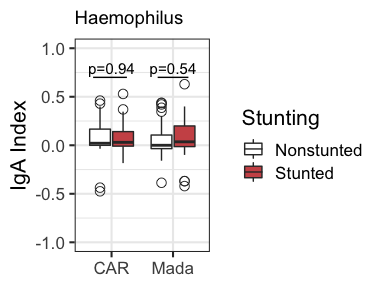


#Haemophilus Iga Index by batch
wilcox.test(pdata$taxa~pdata$Batch)

##
## Wilcoxon rank sum test with continuity correction
##
## data: pdata$taxa by pdata$Batch
## W = 1674, p-value = 0.01118
## alternative hypothesis: true location shift is not equal to 0

pdata_Batch1 <- pdata[pdata$Batch=="Batch1",]
pdata_Batch2 <- pdata[pdata$Batch=="Batch2",]
wilcox.test(pdata_Batch1$taxa~pdata_Batch1$Stunting)

##
## Wilcoxon rank sum test with continuity correction
##
## data: pdata_Batch1$taxa by pdata_Batch1$Stunting
## W = 311, p-value = 0.4781
## alternative hypothesis: true location shift is not equal to 0

median(pdata_Batch1$taxa)

## [1] 0

mean(pdata_Batch1$taxa)

## [1] 0.02300699

wilcox.test(pdata_Batch2$taxa~pdata_Batch2$Stunting)

##
## Wilcoxon rank sum test with continuity correction
##
## data: pdata_Batch2$taxa by pdata_Batch2$Stunting
## W = 894, p-value = 0.9474
## alternative hypothesis: true location shift is not equal to 0

median(pdata_Batch2$taxa)

## [1] 0.03883826

mean(pdata_Batch2$taxa)

## [1] 0.09912935

p <- ggplot(pdata) + geom_boxplot(aes(x=Batch, y=taxa), outlier.shape=1, outlier.size=3, fill="gray70")
p <- p + ggtitle("Haemophilus")
p <- p + theme_bw(base_size=16)
p <- p + theme(plot.title = element_text(size=15))
p <- p + ylab("IgA Index") + xlab(NULL)
p <- p + ylim(-1, 1)
p <- p + annotate("text", x = 1.5, y=0.8, label = "p=0.01", size=4)
p <- p + annotate("segment", x=1, xend=2, y=0.7, yend=0.7)
p


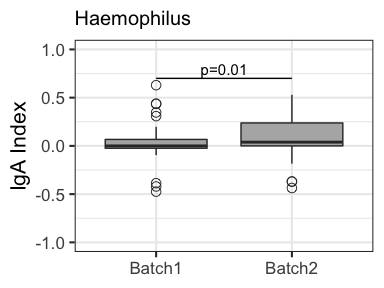


# Campylobacter and Haemophilus IgA index by inflammatory markers - Fig S7

#Campylobacter and inflammatory markers
metaIgA_genus <- as.data.frame(sample_data(IgPos_forindexGen))
pdata = data.frame(taxa=IgA_batchtrim75_genus_t$`D_0__Bacteria|D_1__Epsilonbacteraeota|D_2__Campylobacteria|D_3__Campylobacterales|D_4__Campylobacteraceae|D_5__Campylobacter`,
 AAT=metaIgA_genus$AATmggdePS,
 Calprotectin=metaIgA_genus$CALPROTECTINEggdePS,
 CRP=metaIgA_genus$crp_seuil,
 Country=metaIgA_genus$Country2)

cor.test(pdata$taxa, pdata$AAT, method='spearman')

##
## Spearman's rank correlation rho
##
## data: pdata$taxa and pdata$AAT
## S = 291701, p-value = 0.6762
## alternative hypothesis: true rho is not equal to 0
## sample estimates:
## rho
## -0.038675

cor.test(pdata$taxa, pdata$Calprotectin, method='spearman')

##
## Spearman's rank correlation rho
##
## data: pdata$taxa and pdata$Calprotectin
## S = 391806, p-value = 0.4282
## alternative hypothesis: true rho is not equal to 0
## sample estimates:
## rho
## -0.07008517

wilcox.test(pdata$taxa~pdata$CRP)

##
## Wilcoxon rank sum test with continuity correction
##
## data: pdata$taxa by pdata$CRP
## W = 1146, p-value = 0.1161
## alternative hypothesis: true location shift is not equal to 0

#Campy and AAT
p <- ggplot(pdata,aes(x=AAT, y=taxa))+ geom_point(shape=1, size=4) + geom_smooth(method=lm)
p <- p + ggtitle("Campylobacter")
p <- p + theme_bw(base_size=16)
p <- p + xlab("AAT (mg/g)") + ylab("IgA Index")
p <- p + ylim(-1,1)
p <- p + annotate("text", x = 125, y=-0.6, label = "p=0.68", size=4)
p <- p + annotate("text", x = 125, y=-0.75, label = "rho=-0.04", size=4)
p


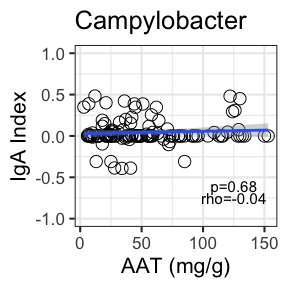


#Campy and calpro
p <- ggplot(pdata, aes(x=Calprotectin, y=taxa))+ geom_point(shape=1, size=4) + geom_smooth(method=lm)
p <- p + ggtitle("Campylobacter")
p <- p + theme_bw(base_size=16)
p <- p + xlab("Calprotectin (mg/g)") + ylab("IgA Index")
p <- p + ylim(-1,1)
p <- p + annotate("text", x = 3000, y=-0.6, label = "p=0.43", size=4)
p <- p + annotate("text", x = 3000, y=-0.75, label = "rho=-0.07", size=4)
p


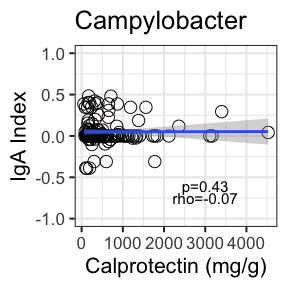


#Campy and CRP
pdata <- pdata[which(pdata$CRP!="NA"),]
pdata$CRP <- ifelse(pdata$CRP=="CRP normale (<=10 mg/l)", yes="Normal", no="High")
pdata$CRP <- factor(pdata$CRP, levels=c("Normal", "High"))

p <- ggplot(pdata) + geom_boxplot(aes(x=CRP, y=taxa), outlier.shape=1, outlier.size=3, fill="grey70")
p <- p + ggtitle("Campylobacter")
p <- p + theme_bw(base_size=16)
p <- p + ylab("IgA Index") + xlab("CRP")
p <- p + ylim(-1, 1)
p <- p + annotate("text", x = 1.5, y=0.8, label = "p=0.12", size=4)
p <- p + annotate("segment", x = 1, xend=2, y=0.7, yend=0.7)
p


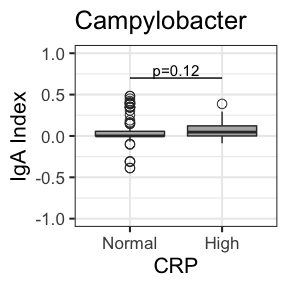


#Haemophilus and inflammatory markers
pdata = data.frame(taxa=IgA_batchtrim75_genus_t$`D_0__Bacteria|D_1__Proteobacteria|D_2__Gammaproteobacteria|D_3__Pasteurellales|D_4__Pasteurellaceae|D_5__Haemophilus`,
 AAT=metaIgA$AATmggdePS,
 Calprotectin=metaIgA$CALPROTECTINEggdePS,
 CRP=metaIgA$crp_seuil,
 Country=metaIgA$Country2)

cor.test(pdata$taxa, pdata$AAT, method='spearman')

##
## Spearman's rank correlation rho
##
## data: pdata$taxa and pdata$AAT
## S = 283837, p-value = 0.9083
## alternative hypothesis: true rho is not equal to 0
## sample estimates:
## rho
## -0.01067285

cor.test(pdata$taxa, pdata$Calprotectin, method='spearman')

##
## Spearman's rank correlation rho
##
## data: pdata$taxa and pdata$Calprotectin
## S = 330460, p-value = 0.27
## alternative hypothesis: true rho is not equal to 0
## sample estimates:
## rho
## 0.09746161

wilcox.test(pdata$taxa~pdata$CRP)

##
## Wilcoxon rank sum test with continuity correction
##
## data: pdata$taxa by pdata$CRP
## W = 953, p-value = 0.9013
## alternative hypothesis: true location shift is not equal to 0

#Haemo and AAT
p <- ggplot(pdata,aes(x=AAT, y=taxa))+ geom_point(shape=1, size=4) + geom_smooth(method=lm)
p <- p + ggtitle("Haemophilus")
p <- p + theme_bw(base_size=16)
p <- p + xlab("AAT (mg/g)") + ylab("IgA Index")
p <- p + ylim(-1,1)
p <- p + annotate("text", x = 125, y=-0.6, label = "p=0.91", size=4)
p <- p + annotate("text", x = 125, y=-0.75, label = "rho=-0.01", size=4)
p


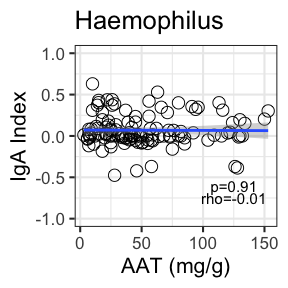


#Haemo and calpro
p <- ggplot(pdata, aes(x=Calprotectin, y=taxa))+ geom_point(shape=1, size=4) + geom_smooth(method=lm)
p <- p + ggtitle("Haemophilus")
p <- p + theme_bw(base_size=16)
p <- p + xlab("Calprotectin (mg/g)") + ylab("IgA Index")
p <- p + ylim(-1,1)
p <- p + annotate("text", x = 3000, y=-0.6, label = "p=0.27", size=4)
p <- p + annotate("text", x = 3000, y=-0.75, label = "rho=0.10", size=4)
p


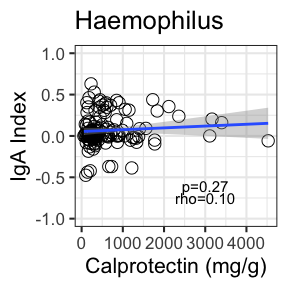


#Haemo and CRP
pdata <- pdata[which(pdata$CRP!="NA"),]
pdata$CRP <- ifelse(pdata$CRP=="CRP normale (<=10 mg/l)", yes="Normal", no="High")
pdata$CRP <- factor(pdata$CRP, levels=c("Normal", "High"))

p <- ggplot(pdata) + geom_boxplot(aes(x=CRP, y=taxa), outlier.shape=1, outlier.size=3, fill="gray70")
p <- p + ggtitle("Haemophilus")
p <- p + theme_bw(base_size=16)
p <- p + ylab("IgA Index") + xlab("CRP")
p <- p + ylim(-1, 1)
p <- p + annotate("text", x = 1.5, y=0.8, label = "p=0.90", size=4)
p <- p + annotate("segment", x = 1, xend=2, y=0.7, yend=0.7)
p


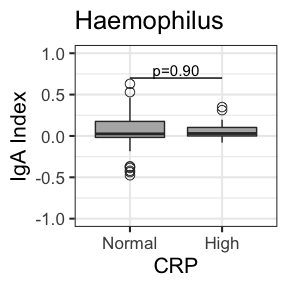


# PCA plot and PERMANOVA analysis: Fig 3 and Fig S8

### PCA plot Fig 3A

#PCA plot coloured by Country
metaIgA <- as.data.frame(sample_data(IgPos_forindex))
iga.pca <- prcomp(IgA_batchtrim75_t)

p <- ggbiplot(iga.pca,ellipse=FALSE,var.axes=FALSE,
 groups=metaIgA$Country2)
p <- p + geom_point(aes(fill=metaIgA$Country2), shape=21, size=5, colour="black")
p <- p + scale_fill_manual(values=c("lightskyblue1", "deepskyblue4"))
p <- p + ggtitle("PCA of IgA Index")
p <- p + theme_bw(base_size=16)
p <- p + guides(groups=FALSE) + labs("Country")
p


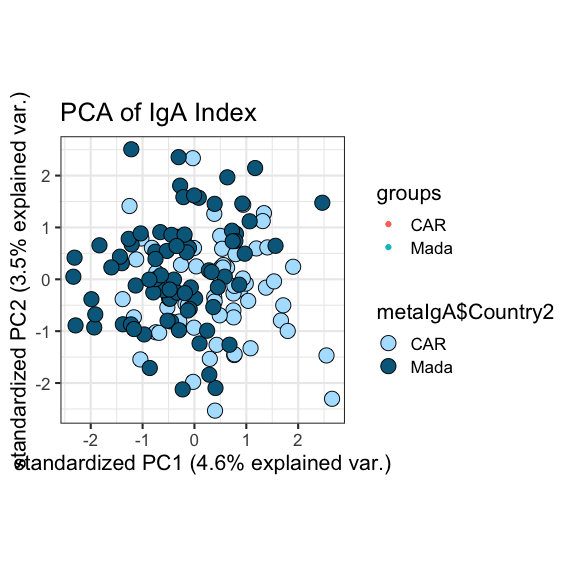


## PERMANOVA analysis of IgA Index - ASV level

### Permanova Analysis on Full Dataset (both countries)

set.seed(123)
#PERMANOVA analysis - gather data frame
metaIgA <- as.data.frame(sample_data(IgPos_forindex))
data_adonis <- data.frame(IgA_batchtrim75_t, metaIgA)
#adding sample sums / depth
sample_sums_pos <- sample_data(IgPos_forindex)$sample_sums
sample_sums_neg <- sample_data(IgNeg_forindex)$sample_sums
data_adonis$depth <- as.numeric(sample_sums_pos)/as.numeric(sample_sums_neg)

#simple PERMANOVA model with main factors
adonis.index <- adonis(IgA_batchtrim75_t ~ depth + Batch + Country + age + sexe + haz_cont,
 data = data_adonis, method='eu', sqrt.dist = FALSE)
adonis.index #depth, batch, country, age are significant; sex and haz are not

##
## Call:
## adonis(formula = IgA_batchtrim75_t ~ depth + Batch + Country + age + sexe + haz_cont, data = data_adonis, method = "eu", sqrt.dist = FALSE)
##
## Permutation: free
## Number of permutations: 999
##
## Terms added sequentially (first to last)
##
## Df SumsOfSqs MeanSqs F.Model R2 Pr(>F)
## depth 1 8.37 8.3653 1.7980 0.01282 0.001 ***
## Batch 1 6.98 6.9838 1.5011 0.01070 0.002 **
## Country 1 11.57 11.5732 2.4875 0.01773 0.001 ***
## age 1 6.12 6.1152 1.3144 0.00937 0.020 *
## sexe 1 4.83 4.8252 1.0371 0.00739 0.378
## haz_cont 1 5.28 5.2796 1.1348 0.00809 0.162
## Residuals 131 609.48 4.6525 0.93389
## Total 137 652.63 1.00000
## ---
## Signif. codes: 0 '***' 0.001 '**' 0.01 '*' 0.05 '.' 0.1 ' ' 1

#FDR correction of PERMANOVA, testing one variable at a time

#generate an empty data frame to store results
permanova.results <- data.frame(Variable=character(),
 p.val=numeric(),
 coef=numeric(),
 stringsAsFactors=FALSE)

#full dataset - select variables of interest
metaIgA_reduced <- data_adonis %>%
 select("depth", "Batch", "Country", "age", "sexe",
 "Percent_IgA_clean", "IgA_Obs_Conc",
 "age_allaite", "helminth",
 "anemie2", "haz_cont", "whz_cont",
 "CALPROTECTINEggdePS", "AATmggdePS", "crp_seuil"
 )
dim(metaIgA_reduced)

## [1] 138 15

dim(IgA_batchtrim75_t)

## [1] 138 140

#loop through each variable and record significance
for(i in 1:15) {
 meta_filt <- metaIgA_reduced[,i]
 df <- data.frame(meta_filt, IgA_batchtrim75_t)
 df_filt <- na.omit(df)
 y <- adonis(df_filt[,2:141] ~ df_filt[,1], data = df_filt, method='eu', sqrt.dist = FALSE)
 permanova.results[i,1] <- names(metaIgA_reduced)[i]
 permanova.results[i,2] <- y$aov.tab[1,6]
 permanova.results[i,3] <- y$aov.tab[1,5]
}

#after looping through all variables
permanova.results$fdr <- p.adjust(permanova.results$p.val, method="fdr")
permanova.results$subset <- c("All (N=138)")
permanova.results[which(permanova.results$fdr<0.05),]

## Variable p.val coef fdr subset
## 1 depth 0.001 0.01281797 0.005 All (N=138)
## 2 Batch 0.002 0.01114678 0.006 All (N=138)
## 3 Country 0.001 0.01744515 0.005 All (N=138)
## 8 age_allaite 0.002 0.01173407 0.006 All (N=138)
## 9 helminth 0.001 0.01791009 0.005 All (N=138)
## 14 AATmggdePS 0.008 0.01184296 0.020 All (N=138)

### Permanova analysis on each country separately

#Madagascar
data_adonis_filt <- dplyr::filter(data_adonis, Country=="Mada")

metaIgA_reduced_filt <- data_adonis_filt %>%
 select("depth", "Batch", "age", "sexe",
 "Percent_IgA_clean", "IgA_Obs_Conc",
 "age_allaite", "helminth",
 "anemie2", "haz_cont", "whz_cont",
 "CALPROTECTINEggdePS", "AATmggdePS", "crp_seuil"
 )

dim(metaIgA_reduced_filt)

## [1] 78 14

dim(data_adonis_filt)

## [1] 78 537

IgA_trim75_Mada <- data_adonis_filt[,1:140] #the numeric IgA indexes (excluding metadata columns)

permanova.results.Mada <- data.frame(Variable=character(),
 p.val=numeric(),
 coef=numeric(),
 stringsAsFactors=FALSE)

for(i in 1:14) {
 meta_filt <- metaIgA_reduced_filt[,i]
 df <- data.frame(meta_filt, IgA_trim75_Mada)
 df_filt <- na.omit(df)
 y <- adonis(df_filt[,2:141] ~ df_filt[,1], data = df_filt, method='eu', sqrt.dist = FALSE)
 permanova.results.Mada[i,1] <- names(metaIgA_reduced_filt)[i]
 permanova.results.Mada[i,2] <- y$aov.tab[1,6]
 permanova.results.Mada[i,3] <- y$aov.tab[1,5]
}

#after looping through all variables
permanova.results.Mada$fdr <- p.adjust(permanova.results.Mada$p.val, method="fdr")
permanova.results.Mada$subset <- c("Mada (N=78)")
permanova.results.Mada[which(permanova.results.Mada$fdr<0.05),]

## Variable p.val coef fdr subset
## 2 Batch 0.002 0.01880544 0.028 Mada (N=78)

#RCA
data_adonis_filt <- dplyr::filter(data_adonis, Country=="RCA")

metaIgA_reduced_filt <- data_adonis_filt %>%
 select("depth", "Batch", "age", "sexe",
 "Percent_IgA_clean", "IgA_Obs_Conc",
 "age_allaite", "helminth",
 "anemie2", "haz_cont", "whz_cont",
 "CALPROTECTINEggdePS", "AATmggdePS", "crp_seuil"
 )

dim(metaIgA_reduced_filt)

## [1] 60 14

dim(data_adonis_filt)

## [1] 60 537

IgA_trim75_RCA <- data_adonis_filt[,1:140] #the numeric IgA indexes (excluding metadata columns)

permanova.results.RCA <- data.frame(Variable=character(),
 p.val=numeric(),
 coef=numeric(),
 stringsAsFactors=FALSE)


for(i in 1:14) {
 meta_filt <- metaIgA_reduced_filt[,i]
 df <- data.frame(meta_filt, IgA_trim75_RCA)
 df_filt <- na.omit(df)
 y <- adonis(df_filt[,2:141] ~ df_filt[,1], data = df_filt, method='eu', sqrt.dist = FALSE)
 permanova.results.RCA[i,1] <- names(metaIgA_reduced_filt)[i]
 permanova.results.RCA[i,2] <- y$aov.tab[1,6]
 permanova.results.RCA[i,3] <- y$aov.tab[1,5]
}

#after looping through all variables
permanova.results.RCA$fdr <- p.adjust(permanova.results.RCA$p.val, method="fdr")
permanova.results.RCA$subset <- c("CAR (N=60)")
permanova.results.RCA[which(permanova.results.RCA$fdr<0.05),]

## [1] Variable p.val coef fdr subset
## <0 rows> (or 0-length row.names)

### Combine all results into a plot indicating variable and PERMANOVA signifiance - Fig 3B

#all together
permanova.results.combined <- Reduce(full_join, list(permanova.results,
 permanova.results.Mada,
 permanova.results.RCA))

## Joining, by = c("Variable", "p.val", "coef", "fdr", "subset")
## Joining, by = c("Variable", "p.val", "coef", "fdr", "subset")

#clean and translate variable names for figure
permanova.results.combined$Variable <- gsub("sexe", "sex", permanova.results.combined$Variable)
permanova.results.combined$Variable <- gsub("Percent_IgA_clean", "percent IgA+", permanova.results.combined$Variable)
permanova.results.combined$Variable <- gsub("IgA_Obs_Conc", "total IgA", permanova.results.combined$Variable)
permanova.results.combined$Variable <- gsub("anemie2", "anemia", permanova.results.combined$Variable)
permanova.results.combined$Variable <- gsub("crp_seuil", "crp", permanova.results.combined$Variable)
permanova.results.combined$Variable <- gsub("Country", "country", permanova.results.combined$Variable)
permanova.results.combined$Variable <- gsub("Batch", "batch", permanova.results.combined$Variable)
permanova.results.combined$Variable <- gsub("CALPROTECTINEggdePS", "calprotectin", permanova.results.combined$Variable)
permanova.results.combined$Variable <- gsub("AATmggdePS", "aat", permanova.results.combined$Variable)
permanova.results.combined$Variable <- gsub("age_allaite", "breastfeeding", permanova.results.combined$Variable)

p <- ggplot(permanova.results.combined, aes(x=Variable,y=coef, fill=p.val)) + geom_bar(stat="identity")
p <- p + ggtitle("PERMANOVA summary")
p <- p + theme_bw(base_size = 14)
p <- p + scale_fill_gradientn(colours=c("darkseagreen1", "darkseagreen3", "darkseagreen4", "black"), values=c(0, 0.05, 0.1, 0.5, 1))
p <- p + theme(axis.text.x = element_text(angle=90, hjust=1, size=10), plot.title = element_text(size = 12))
p <- p + labs(fill="p value")
p <- p + facet_grid(.~subset, labeller=label_wrap_gen(width=2))
p <- p + coord_flip()
p


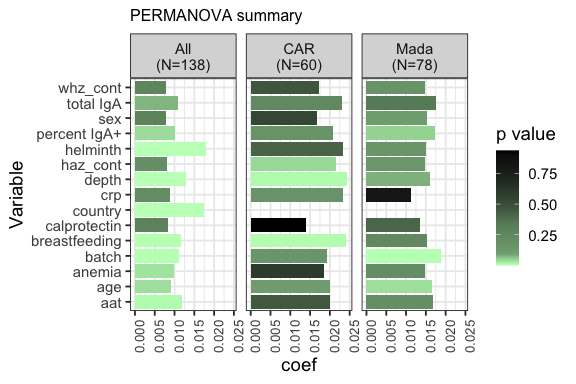


#all results
permanova.results.combined

## Variable p.val coef fdr subset
## 1 depth 0.001 0.012817971 0.00500000 All (N=138)
## 2 batch 0.002 0.011146779 0.00600000 All (N=138)
## 3 country 0.001 0.017445149 0.00500000 All (N=138)
## 4 age 0.037 0.009207778 0.06666667 All (N=138)
## 5 sex 0.280 0.007901477 0.28800000 All (N=138)
## 6 percent IgA+ 0.040 0.009994277 0.06666667 All (N=138)
## 7 total IgA 0.075 0.010898791 0.11250000 All (N=138)
## 8 breastfeeding 0.002 0.011734070 0.00600000 All (N=138)
## 9 helminth 0.001 0.017910086 0.00500000 All (N=138)
## 10 anemia 0.032 0.009793954 0.06666667 All (N=138)
## 11 haz_cont 0.200 0.008110840 0.26625000 All (N=138)
## 12 whz_cont 0.268 0.007954914 0.28800000 All (N=138)
## 13 calprotectin 0.288 0.008349338 0.28800000 All (N=138)
## 14 aat 0.008 0.011842960 0.02000000 All (N=138)
## 15 crp 0.213 0.008818149 0.26625000 All (N=138)
## 16 depth 0.079 0.016088160 0.27650000 Mada (N=78)
## 17 batch 0.002 0.018805435 0.02800000 Mada (N=78)
## 18 age 0.037 0.016664985 0.23333333 Mada (N=78)
## 19 sex 0.107 0.015187466 0.28560000 Mada (N=78)
## 20 percent IgA+ 0.050 0.017198233 0.23333333 Mada (N=78)
## 21 total IgA 0.341 0.017665159 0.39783333 Mada (N=78)
## 22 breastfeeding 0.241 0.015174361 0.30672727 Mada (N=78)
## 23 helminth 0.154 0.014910829 0.28560000 Mada (N=78)
## 24 anemia 0.204 0.014685546 0.28560000 Mada (N=78)
## 25 haz_cont 0.145 0.014774792 0.28560000 Mada (N=78)
## 26 whz_cont 0.172 0.014665779 0.28560000 Mada (N=78)
## 27 calprotectin 0.409 0.013492877 0.44046154 Mada (N=78)
## 28 aat 0.200 0.016681793 0.28560000 Mada (N=78)
## 29 crp 0.817 0.011241066 0.81700000 Mada (N=78)
## 30 depth 0.009 0.024449891 0.06300000 CAR (N=60)
## 31 batch 0.155 0.019308499 0.34800000 CAR (N=60)
## 32 age 0.102 0.020154412 0.34800000 CAR (N=60)
## 33 sex 0.537 0.016624195 0.62650000 CAR (N=60)
## 34 percent IgA+ 0.166 0.020815664 0.34800000 CAR (N=60)
## 35 total IgA 0.242 0.023077378 0.42350000 CAR (N=60)
## 36 breastfeeding 0.007 0.023965471 0.06300000 CAR (N=60)
## 37 helminth 0.419 0.023422419 0.59563636 CAR (N=60)
## 38 anemia 0.591 0.018547410 0.63646154 CAR (N=60)
## 39 haz_cont 0.043 0.021613966 0.20066667 CAR (N=60)
## 40 whz_cont 0.468 0.017133790 0.59563636 CAR (N=60)
## 41 calprotectin 0.936 0.014092893 0.93600000 CAR (N=60)
## 42 aat 0.456 0.020090164 0.59563636 CAR (N=60)
## 43 crp 0.174 0.023284509 0.34800000 CAR (N=60)

#significance
permanova.results.combined[which(permanova.results.combined$fdr<0.05),]

## Variable p.val coef fdr subset
## 1 depth 0.001 0.01281797 0.005 All (N=138)
## 2 batch 0.002 0.01114678 0.006 All (N=138)
## 3 country 0.001 0.01744515 0.005 All (N=138)
## 8 breastfeeding 0.002 0.01173407 0.006 All (N=138)
## 9 helminth 0.001 0.01791009 0.005 All (N=138)
## 14 aat 0.008 0.01184296 0.020 All (N=138)
## 17 batch 0.002 0.01880544 0.028 Mada (N=78)

### Permanova Analysis, Full Dataset, strata correction by country

#simple PERMANOVA wtih country considered as a group (strata) to constrain permutations
adonis.index.strata <- adonis(IgA_batchtrim75_t ~ depth + Batch + age + sexe + haz_cont,
 strata=data_adonis$Country,
 data = data_adonis, method='eu', sqrt.dist = FALSE)
adonis.index.strata #similarly, depth, batch and age are significant, sex and haz are not

##
## Call:
## adonis(formula = IgA_batchtrim75_t ~ depth + Batch + age + sexe + haz_cont, data = data_adonis, method = "eu", strata = data_adonis$Country, sqrt.dist = FALSE)
##
## Blocks: strata
## Permutation: free
## Number of permutations: 999
##
## Terms added sequentially (first to last)
##
## Df SumsOfSqs MeanSqs F.Model R2 Pr(>F)
## depth 1 8.37 8.3653 1.7777 0.01282 0.002 **
## Batch 1 6.98 6.9838 1.4841 0.01070 0.016 *
## age 1 6.02 6.0220 1.2797 0.00923 0.032 *
## sexe 1 4.86 4.8642 1.0336 0.00745 0.366
## haz_cont 1 5.23 5.2263 1.1106 0.00801 0.233
## Residuals 132 621.16 4.7058 0.95179
## Total 137 652.63 1.00000
## ---
## Signif. codes: 0 '***' 0.001 '**' 0.01 '*' 0.05 '.' 0.1 ' ' 1

#FDR correction of PERMANOVA, testing one variable at a time

#generate an empty data frame to store results
permanova.results.strata1 <- data.frame(Variable=character(),
 p.val=numeric(),
 coef=numeric(),
 stringsAsFactors=FALSE)

#full dataset - select variables of interest
metaIgA_reduced <- data_adonis %>%
 select("depth", "Batch", "Country", "age", "sexe",
 "Percent_IgA_clean", "IgA_Obs_Conc",
 "age_allaite", "helminth",
 "anemie2", "haz_cont", "whz_cont",
 "CALPROTECTINEggdePS", "AATmggdePS", "crp_seuil"
 )
dim(metaIgA_reduced)

## [1] 138 15

dim(IgA_batchtrim75_t)

## [1] 138 140

#loop through each variable and record significance
for(i in 1:15) {
 meta_filt <- metaIgA_reduced[,i]
 df <- data.frame(meta_filt, metaIgA_reduced$Country, IgA_batchtrim75_t)
 df_filt <- na.omit(df)
 y <- adonis(df_filt[,3:141] ~ df_filt[,1], strata=df_filt[,2], data = df_filt, method='eu', sqrt.dist = FALSE)
 permanova.results.strata1[i,1] <- names(metaIgA_reduced)[i]
 permanova.results.strata1[i,2] <- y$aov.tab[1,6]
 permanova.results.strata1[i,3] <- y$aov.tab[1,5]
}

#after looping through all variables
permanova.results.strata1$fdr <- p.adjust(permanova.results.strata1$p.val, method="fdr")
permanova.results.strata1$subset <- c("All (~Country)")
permanova.results.strata1[which(permanova.results.strata1$fdr<0.05),]

## Variable p.val coef fdr subset
## 1 depth 0.002 0.01285417 0.0300 All (~Country)
## 14 AATmggdePS 0.005 0.01192633 0.0375 All (~Country)

### Permanova Analysis, Full Dataset, strata correction by batch

#PERMANOVA wtih batch considered as a group (strata) to constrain permutations
#FDR correction of PERMANOVA, testing one variable at a time

#generate an empty data frame to store results
permanova.results.strata2 <- data.frame(Variable=character(),
 p.val=numeric(),
 coef=numeric(),
 stringsAsFactors=FALSE)

#full dataset - select variables of interest
metaIgA_reduced <- data_adonis %>%
 select("depth", "Batch", "Country", "age", "sexe",
 "Percent_IgA_clean", "IgA_Obs_Conc",
 "age_allaite", "helminth",
 "anemie2", "haz_cont", "whz_cont",
 "CALPROTECTINEggdePS", "AATmggdePS", "crp_seuil"
 )
dim(metaIgA_reduced)

## [1] 138 15

dim(IgA_batchtrim75_t)

## [1] 138 140

#loop through each variable and record significance
for(i in 1:15) {
 meta_filt <- metaIgA_reduced[,i]
 df <- data.frame(meta_filt, metaIgA_reduced$Batch, IgA_batchtrim75_t)
 df_filt <- na.omit(df)
 y <- adonis(df_filt[,3:141] ~ df_filt[,1], strata=df_filt[,2], data = df_filt, method='eu', sqrt.dist = FALSE)
 permanova.results.strata2[i,1] <- names(metaIgA_reduced)[i]
 permanova.results.strata2[i,2] <- y$aov.tab[1,6]
 permanova.results.strata2[i,3] <- y$aov.tab[1,5]
}


#after looping through all variables
permanova.results.strata2$fdr <- p.adjust(permanova.results.strata2$p.val, method="fdr")
permanova.results.strata2$subset <- c("All (~Batch)")
permanova.results.strata2[which(permanova.results.strata2$fdr<0.05),]

## Variable p.val coef fdr subset
## 1 depth 0.001 0.01285417 0.0050 All (~Batch)
## 3 Country 0.001 0.01753294 0.0050 All (~Batch)
## 8 age_allaite 0.002 0.01162996 0.0075 All (~Batch)
## 9 helminth 0.001 0.01792936 0.0050 All (~Batch)
## 14 AATmggdePS 0.012 0.01192633 0.0360 All (~Batch)

### PERMANOVA analysis on each batch separately

#Batch 1
data_adonis_filt <- dplyr::filter(data_adonis, Batch=="Batch1")

metaIgA_reduced_filt <- data_adonis_filt %>%
 select("depth", "Country", "age", "sexe",
 "Percent_IgA_clean", "IgA_Obs_Conc",
 "age_allaite", "helminth",
 "anemie2", "haz_cont", "whz_cont",
 "CALPROTECTINEggdePS", "AATmggdePS", "crp_seuil"
 )

dim(metaIgA_reduced_filt) #53x14

## [1] 53 14

IgA_trim75_Batch1 <- data_adonis_filt[,1:140]

permanova.results.Batch1 <- data.frame(Variable=character(),
 p.val=numeric(),
 coef=numeric(),
 stringsAsFactors=FALSE)


for(i in 1:14) {
 meta_filt <- metaIgA_reduced_filt[,i]
 df <- data.frame(meta_filt, IgA_trim75_Batch1)
 df_filt <- na.omit(df)
 y <- adonis(df_filt[,2:141] ~ df_filt[,1], data = df_filt, method='eu', sqrt.dist = FALSE)
 permanova.results.Batch1[i,1] <- names(metaIgA_reduced_filt)[i]
 permanova.results.Batch1[i,2] <- y$aov.tab[1,6]
 permanova.results.Batch1[i,3] <- y$aov.tab[1,5]
}

#after looping through all variables
permanova.results.Batch1$fdr <- p.adjust(permanova.results.Batch1$p.val, method="fdr")
permanova.results.Batch1$subset <- c("Batch1 (N=53)")
permanova.results.Batch1[which(permanova.results.Batch1$fdr<0.05),]

## Variable p.val coef fdr subset
## 2 Country 0.001 0.02824291 0.007000000 Batch1 (N=53)
## 9 anemie2 0.002 0.02755702 0.009333333 Batch1 (N=53)
## 10 haz_cont 0.001 0.03123576 0.007000000 Batch1 (N=53)

#Batch 2
data_adonis_filt <- dplyr::filter(data_adonis, Batch=="Batch2")

metaIgA_reduced_filt <- data_adonis_filt %>%
 select("depth", "Country", "age", "sexe",
 "Percent_IgA_clean", "IgA_Obs_Conc",
 "age_allaite", "helminth",
 "anemie2", "haz_cont", "whz_cont",
 "CALPROTECTINEggdePS", "AATmggdePS", "crp_seuil"
 )

dim(metaIgA_reduced_filt) #85x14

## [1] 85 14

IgA_trim75_Batch2 <- data_adonis_filt[,1:140]

permanova.results.Batch2 <- data.frame(Variable=character(),
 p.val=numeric(),
 coef=numeric(),
 stringsAsFactors=FALSE)


for(i in 1:14) {
 meta_filt <- metaIgA_reduced_filt[,i]
 df <- data.frame(meta_filt, IgA_trim75_Batch2)
 df_filt <- na.omit(df)
 y <- adonis(df_filt[,2:141] ~ df_filt[,1], data = df_filt, method='eu', sqrt.dist = FALSE)
 permanova.results.Batch2[i,1] <- names(metaIgA_reduced_filt)[i]
 permanova.results.Batch2[i,2] <- y$aov.tab[1,6]
 permanova.results.Batch2[i,3] <- y$aov.tab[1,5]
}

#after looping through all variables
permanova.results.Batch2$fdr <- p.adjust(permanova.results.Batch2$p.val, method="fdr")
permanova.results.Batch2$subset <- c("Batch2 (N=85)")
permanova.results.Batch2[which(permanova.results.Batch2$fdr<0.05),]

## Variable p.val coef fdr subset
## 1 depth 0.007 0.01762245 0.02450000 Batch2 (N=85)
## 2 Country 0.001 0.02350363 0.00700000 Batch2 (N=85)
## 3 age 0.020 0.01606033 0.04666667 Batch2 (N=85)
## 7 age_allaite 0.003 0.02016014 0.01400000 Batch2 (N=85)
## 8 helminth 0.001 0.02420542 0.00700000 Batch2 (N=85)
## 13 AATmggdePS 0.020 0.01905446 0.04666667 Batch2 (N=85)

### Combine and plot results from strata & batch analysis - Fig S8A

#all together
permanova.results.combined <- Reduce(full_join, list(permanova.results.strata1,
 permanova.results.strata2,
 permanova.results.Batch1,
 permanova.results.Batch2
 ))

## Joining, by = c("Variable", "p.val", "coef", "fdr", "subset")
## Joining, by = c("Variable", "p.val", "coef", "fdr", "subset")
## Joining, by = c("Variable", "p.val", "coef", "fdr", "subset")

#clean and translate variable names for figure
permanova.results.combined$Variable <- gsub("sexe", "sex", permanova.results.combined$Variable)
permanova.results.combined$Variable <- gsub("Percent_IgA_clean", "percent IgA+", permanova.results.combined$Variable)
permanova.results.combined$Variable <- gsub("IgA_Obs_Conc", "total IgA", permanova.results.combined$Variable)
permanova.results.combined$Variable <- gsub("anemie2", "anemia", permanova.results.combined$Variable)
permanova.results.combined$Variable <- gsub("crp_seuil", "crp", permanova.results.combined$Variable)
permanova.results.combined$Variable <- gsub("Country", "country", permanova.results.combined$Variable)
permanova.results.combined$Variable <- gsub("Batch", "batch", permanova.results.combined$Variable)
permanova.results.combined$Variable <- gsub("CALPROTECTINEggdePS", "calprotectin", permanova.results.combined$Variable)
permanova.results.combined$Variable <- gsub("AATmggdePS", "aat", permanova.results.combined$Variable)
permanova.results.combined$Variable <- gsub("age_allaite", "breastfeeding", permanova.results.combined$Variable)

p <- ggplot(permanova.results.combined, aes(x=Variable,y=coef, fill=p.val)) + geom_bar(stat="identity")
p <- p + ggtitle("PERMANOVA summary (ASV)")
p <- p + theme_bw(base_size = 14)
p <- p + scale_fill_gradientn(colours=c("darkseagreen1", "darkseagreen3", "darkseagreen4", "black"), values=c(0, 0.05, 0.1, 0.5, 1))
p <- p + theme(axis.text.x = element_text(angle=90, hjust=1, size=10), plot.title = element_text(size = 12))
p <- p + labs(fill="p value")
p <- p + facet_grid(.~subset,labeller=label_wrap_gen(width=2))
p <- p + coord_flip()
p


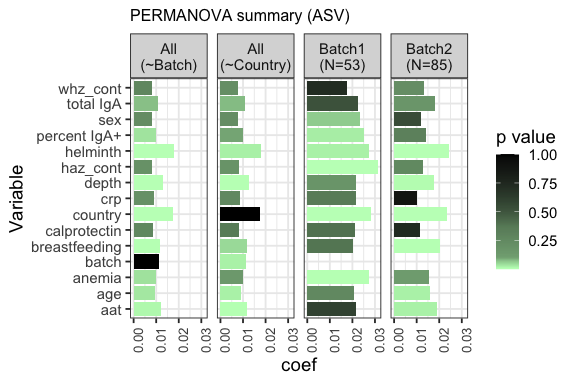


#significance
permanova.results.combined[which(permanova.results.combined$fdr<0.05),]

## Variable p.val coef fdr subset
## 1 depth 0.002 0.01285417 0.030000000 All (~Country)
## 14 aat 0.005 0.01192633 0.037500000 All (~Country)
## 16 depth 0.001 0.01285417 0.005000000 All (~Batch)
## 18 country 0.001 0.01753294 0.005000000 All (~Batch)
## 23 breastfeeding 0.002 0.01162996 0.007500000 All (~Batch)
## 24 helminth 0.001 0.01792936 0.005000000 All (~Batch)
## 29 aat 0.012 0.01192633 0.036000000 All (~Batch)
## 32 country 0.001 0.02824291 0.007000000 Batch1 (N=53)
## 39 anemia 0.002 0.02755702 0.009333333 Batch1 (N=53)
## 40 haz_cont 0.001 0.03123576 0.007000000 Batch1 (N=53)
## 45 depth 0.007 0.01762245 0.024500000 Batch2 (N=85)
## 46 country 0.001 0.02350363 0.007000000 Batch2 (N=85)
## 47 age 0.020 0.01606033 0.046666667 Batch2 (N=85)
## 51 breastfeeding 0.003 0.02016014 0.014000000 Batch2 (N=85)
## 52 helminth 0.001 0.02420542 0.007000000 Batch2 (N=85)
## 57 aat 0.020 0.01905446 0.046666667 Batch2 (N=85)

## PERMANOVA analysis - genus level - Fig S8B

### Main PERMANOVA analysis in full dataset and in each country, with genus level taxa - Fig S8B

#PERMANOVA of main factors at once
#gather dataframe for adonis adonis
data_adonis_genus <- data.frame(IgA_genus_trim75_t, as.data.frame(sample_data(IgPos_forindexGen)))
#adding sample sums / depth
sample_sums_posGen <- sample_data(IgPos_forindexGen)$sample_sums
sample_sums_negGen <- sample_data(IgNeg_forindexGen)$sample_sums
data_adonis_genus$depth <- as.numeric(sample_sums_posGen)/as.numeric(sample_sums_negGen)

#simple model
adonis.index <- adonis(IgA_genus_trim75_t ~ depth + Batch + Country + age + sexe + haz_cont,
 data = data_adonis_genus, method='eu', sqrt.dist = FALSE)
adonis.index

##
## Call:
## adonis(formula = IgA_genus_trim75_t ~ depth + Batch + Country + age + sexe + haz_cont, data = data_adonis_genus, method = "eu", sqrt.dist = FALSE)
##
## Permutation: free
## Number of permutations: 999
##
## Terms added sequentially (first to last)
##
## Df SumsOfSqs MeanSqs F.Model R2 Pr(>F)
## depth 1 6.55 6.5502 1.7036 0.01218 0.001 ***
## Batch 1 6.37 6.3746 1.6580 0.01185 0.001 ***
## Country 1 6.82 6.8204 1.7739 0.01268 0.002 **
## age 1 5.26 5.2586 1.3677 0.00978 0.008 **
## sexe 1 4.09 4.0929 1.0645 0.00761 0.304
## haz_cont 1 5.02 5.0172 1.3049 0.00933 0.027 *
## Residuals 131 503.67 3.8448 0.93657
## Total 137 537.79 1.00000
## ---
## Signif. codes: 0 '***' 0.001 '**' 0.01 '*' 0.05 '.' 0.1 ' ' 1

metaIgA_genus_reduced <- data_adonis_genus %>%
 select("depth", "Batch", "Country", "age", "sexe",
 "Percent_IgA_clean", "IgA_Obs_Conc",
 "age_allaite", "helminth",
 "anemie2", "haz_cont", "whz_cont",
 "CALPROTECTINEggdePS", "AATmggdePS", "crp_seuil"
 )

dim(metaIgA_genus_reduced) #135x15

## [1] 138 15

#loop through a permanova of each factor
permanova.results.genus <- data.frame(Variable=character(),
 p.val=numeric(),
 coef=numeric(),
 stringsAsFactors=FALSE)

for(i in 1:15) {
 meta_filt <- metaIgA_genus_reduced[,i]
 df <- data.frame(meta_filt, IgA_genus_trim75_t)
 df_filt <- na.omit(df)
 y <- adonis(df_filt[,2:127] ~ df_filt[,1], data = df_filt, method='eu', sqrt.dist = FALSE)
 permanova.results.genus[i,1] <- names(metaIgA_genus_reduced)[i]
 permanova.results.genus[i,2] <- y$aov.tab[1,6]
 permanova.results.genus[i,3] <- y$aov.tab[1,5]
}

#after looping through all variables
permanova.results.genus$fdr <- p.adjust(permanova.results.genus$p.val, method="fdr")
permanova.results.genus$subset <- c("All (N=138)")
permanova.results.genus[which(permanova.results.genus$fdr<0.05),]

## Variable p.val coef fdr subset
## 1 depth 0.001 0.012179839 0.00375 All (N=138)
## 2 Batch 0.001 0.011741901 0.00375 All (N=138)
## 3 Country 0.001 0.012299119 0.00375 All (N=138)
## 4 age 0.009 0.009915858 0.02700 All (N=138)
## 9 helminth 0.001 0.014054662 0.00375 All (N=138)

#Madagascar by genus
data_adonis_genus_filt <- dplyr::filter(data_adonis_genus, Country=="Mada")

metaIgA_genus_reduced_filt <- data_adonis_genus_filt %>%
 select("depth", "Batch", "age", "sexe",
 "Percent_IgA_clean", "IgA_Obs_Conc",
 "age_allaite", "helminth",
 "anemie2", "haz_cont", "whz_cont",
 "CALPROTECTINEggdePS", "AATmggdePS", "crp_seuil"
 )

dim(metaIgA_genus_reduced_filt) #78x14

## [1] 78 14

IgA_genus_trim75_Mada <- data_adonis_genus_filt[,1:126]

permanova.results.genus.Mada <- data.frame(Variable=character(),
 p.val=numeric(),
 coef=numeric(),
 stringsAsFactors=FALSE)


for(i in 1:14) {
 meta_filt <- metaIgA_genus_reduced_filt[,i]
 df <- data.frame(meta_filt, IgA_genus_trim75_Mada)
 df_filt <- na.omit(df)
 y <- adonis(df_filt[,2:127] ~ df_filt[,1], data = df_filt, method='eu', sqrt.dist = FALSE)
 permanova.results.genus.Mada[i,1] <- names(metaIgA_genus_reduced_filt)[i]
 permanova.results.genus.Mada[i,2] <- y$aov.tab[1,6]
 permanova.results.genus.Mada[i,3] <- y$aov.tab[1,5]
}

#after looping through all variables
permanova.results.genus.Mada$fdr <- p.adjust(permanova.results.genus.Mada$p.val, method="fdr")
permanova.results.genus.Mada$subset <- c("Mada (N=78)")
permanova.results.genus.Mada[which(permanova.results.genus.Mada$fdr<0.05),]

## [1] Variable p.val coef fdr subset
## <0 rows> (or 0-length row.names)

#RCA by genus
data_adonis_genus_filt <- dplyr::filter(data_adonis_genus, Country=="RCA")

metaIgA_genus_reduced_filt <- data_adonis_genus_filt %>%
 select("depth", "Batch", "age", "sexe",
 "Percent_IgA_clean", "IgA_Obs_Conc",
 "age_allaite", "helminth",
 "anemie2", "haz_cont", "whz_cont",
 "CALPROTECTINEggdePS", "AATmggdePS", "crp_seuil"
 )

dim(metaIgA_genus_reduced_filt) #78x14

## [1] 60 14

IgA_genus_trim75_RCA <- data_adonis_genus_filt[,1:126]

permanova.results.genus.RCA <- data.frame(Variable=character(),
 p.val=numeric(),
 coef=numeric(),
 stringsAsFactors=FALSE)

for(i in 1:14) {
 meta_filt <- metaIgA_genus_reduced_filt[,i]
 df <- data.frame(meta_filt, IgA_genus_trim75_RCA)
 df_filt <- na.omit(df)
 y <- adonis(df_filt[,2:127] ~ df_filt[,1], data = df_filt, method='eu', sqrt.dist = FALSE)
 permanova.results.genus.RCA[i,1] <- names(metaIgA_genus_reduced_filt)[i]
 permanova.results.genus.RCA[i,2] <- y$aov.tab[1,6]
 permanova.results.genus.RCA[i,3] <- y$aov.tab[1,5]
}

#after looping through all variables
permanova.results.genus.RCA$fdr <- p.adjust(permanova.results.genus.RCA$p.val, method="fdr")
permanova.results.genus.RCA$subset <- c("CAR (N=60)")
permanova.results.genus.RCA[which(permanova.results.genus.RCA$fdr<0.05),]

## [1] Variable p.val coef fdr subset
## <0 rows> (or 0-length row.names)

#combine and plot all subsets by genus
permanova.results.combined.genus <- Reduce(full_join, list(permanova.results.genus,
 permanova.results.genus.Mada,
 permanova.results.genus.RCA
 ))

## Joining, by = c("Variable", "p.val", "coef", "fdr", "subset")
## Joining, by = c("Variable", "p.val", "coef", "fdr", "subset")

#plot
permanova.results.combined.genus$Variable <- gsub("sexe", "sex", permanova.results.combined.genus$Variable)
permanova.results.combined.genus$Variable <- gsub("Percent_IgA_clean", "percent IgA+", permanova.results.combined.genus$Variable)
permanova.results.combined.genus$Variable <- gsub("IgA_Obs_Conc", "total IgA", permanova.results.combined.genus$Variable)
permanova.results.combined.genus$Variable <- gsub("anemie2", "anemia", permanova.results.combined.genus$Variable)
permanova.results.combined.genus$Variable <- gsub("crp_seuil", "crp", permanova.results.combined.genus$Variable)
permanova.results.combined.genus$Variable <- gsub("Country", "country", permanova.results.combined.genus$Variable)
permanova.results.combined.genus$Variable <- gsub("Batch", "batch", permanova.results.combined.genus$Variable)
permanova.results.combined.genus$Variable <- gsub("CALPROTECTINEggdePS", "calprotectin", permanova.results.combined.genus$Variable)
permanova.results.combined.genus$Variable <- gsub("AATmggdePS", "aat", permanova.results.combined.genus$Variable)
permanova.results.combined.genus$Variable <- gsub("age_allaite", "breastfeeding", permanova.results.combined.genus$Variable)


p <- ggplot(permanova.results.combined.genus, aes(x=Variable,y=coef, fill=p.val)) + geom_bar(stat="identity")
p <- p + ggtitle("PERMANOVA summary (genus)")
p <- p + theme_bw(base_size = 14)
p <- p + scale_fill_gradientn(colours=c("darkseagreen1", "darkseagreen3", "darkseagreen4", "black"), values=c(0, 0.05, 0.1, 0.5, 1))
p <- p + theme(axis.text.x = element_text(angle=90, hjust=1, size=10), plot.title = element_text(size = 12))
p <- p + labs(fill="p value")
p <- p + facet_grid(.~subset, labeller=label_wrap_gen(width=2))
p <- p + coord_flip()
p


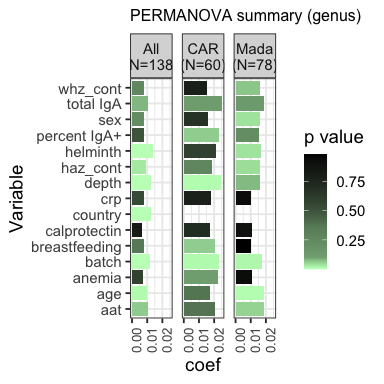


#all results
permanova.results.combined.genus

## Variable p.val coef fdr subset
## 1 depth 0.001 0.012179839 0.0037500 All (N=138)
## 2 batch 0.001 0.011741901 0.0037500 All (N=138)
## 3 country 0.001 0.012299119 0.0037500 All (N=138)
## 4 age 0.009 0.009915858 0.0270000 All (N=138)
## 5 sex 0.225 0.008004380 0.3750000 All (N=138)
## 6 percent IgA+ 0.498 0.007758357 0.6225000 All (N=138)
## 7 total IgA 0.078 0.010763020 0.1462500 All (N=138)
## 8 breastfeeding 0.363 0.007815995 0.4950000 All (N=138)
## 9 helminth 0.001 0.014054662 0.0037500 All (N=138)
## 10 anemia 0.581 0.007435410 0.6225000 All (N=138)
## 11 haz_cont 0.028 0.009400628 0.0700000 All (N=138)
## 12 whz_cont 0.263 0.007938404 0.3945000 All (N=138)
## 13 calprotectin 0.826 0.006619949 0.8260000 All (N=138)
## 14 aat 0.060 0.010517453 0.1285714 All (N=138)
## 15 crp 0.547 0.007721288 0.6225000 All (N=138)
## 16 depth 0.075 0.015833329 0.1312500 Mada (N=78)
## 17 batch 0.013 0.017252013 0.0910000 Mada (N=78)
## 18 age 0.006 0.018584631 0.0840000 Mada (N=78)
## 19 sex 0.035 0.016364460 0.1120000 Mada (N=78)
## 20 percent IgA+ 0.216 0.015237369 0.3024000 Mada (N=78)
## 21 total IgA 0.123 0.019018022 0.1913333 Mada (N=78)
## 22 breastfeeding 0.981 0.010056105 0.9810000 Mada (N=78)
## 23 helminth 0.033 0.016450548 0.1120000 Mada (N=78)
## 24 anemia 0.935 0.010583333 0.9810000 Mada (N=78)
## 25 haz_cont 0.040 0.016354599 0.1120000 Mada (N=78)
## 26 whz_cont 0.066 0.015908179 0.1312500 Mada (N=78)
## 27 calprotectin 0.892 0.010686498 0.9810000 Mada (N=78)
## 28 aat 0.051 0.018575306 0.1190000 Mada (N=78)
## 29 crp 0.931 0.010442896 0.9810000 Mada (N=78)
## 30 depth 0.008 0.024732070 0.0700000 CAR (N=60)
## 31 batch 0.010 0.023391693 0.0700000 CAR (N=60)
## 32 age 0.379 0.017391495 0.5926667 CAR (N=60)
## 33 sex 0.677 0.015764912 0.7960000 CAR (N=60)
## 34 percent IgA+ 0.059 0.023016412 0.2065000 CAR (N=60)
## 35 total IgA 0.111 0.025454528 0.2590000 CAR (N=60)
## 36 breastfeeding 0.058 0.020657013 0.2065000 CAR (N=60)
## 37 helminth 0.592 0.020964373 0.7960000 CAR (N=60)
## 38 anemia 0.105 0.022784558 0.2590000 CAR (N=60)
## 39 haz_cont 0.269 0.018563915 0.5380000 CAR (N=60)
## 40 whz_cont 0.796 0.014972111 0.7960000 CAR (N=60)
## 41 calprotectin 0.708 0.017064551 0.7960000 CAR (N=60)
## 42 aat 0.381 0.020440054 0.5926667 CAR (N=60)
## 43 crp 0.784 0.018093106 0.7960000 CAR (N=60)

#significance
permanova.results.combined.genus[which(permanova.results.combined.genus$fdr<0.05),]

## Variable p.val coef fdr subset
## 1 depth 0.001 0.012179839 0.00375 All (N=138)
## 2 batch 0.001 0.011741901 0.00375 All (N=138)
## 3 country 0.001 0.012299119 0.00375 All (N=138)
## 4 age 0.009 0.009915858 0.02700 All (N=138)
## 9 helminth 0.001 0.014054662 0.00375 All (N=138)

## Table S4 - taxa that correlate with Batch effect

#Multiple testing for taxa that correlate with batch effect (in filtered dataset)#

#select matching IgA and metadata files
IgA_wilcox <- IgA_batchtrim75 #the variables measured
meta_wilcox <- as.data.frame(sample_data(IgPos_forindex)) #the study metadata
names(IgA_wilcox) <- meta_wilcox$ChildID

#IgA and meta file should have the same number of samples in opposite orientation
dim(IgA_wilcox)

## [1] 140 138

dim(meta_wilcox)

## [1] 138 396

#look for main sorting batch differences 2017 vs 2018 (ASV)
meta_1718 <- metaIgA[metaIgA$Sorting_batch_major%in%c(2017, 2018),]
IgA_1718 <- IgA_batchtrim75[,names(IgA_batchtrim75)%in%meta_1718$SampleID]

#multiple wilcox by sorting batch
MW.p = apply(IgA_1718,1,
 function(x) wilcox.test(c(x)~meta_1718$Sorting_batch_major)$p.value)
p.res = data.frame(taxa=row.names(IgA_batchtrim75),MW.p)
# Perform multiple comparison correction using a given method of choice
p.res$rel.fdr <- p.adjust(p.res$MW.p, method="fdr")

#hits
sort_batch_hits <- p.res[which(p.res$rel.fdr<0.05),]$taxa
sort_batch_hits

## [1] "D_0__Bacteria|D_1__Proteobacteria|D_2__Gammaproteobacteria|D_3__Pseudomonadales|D_4__Pseudomonadaceae|D_5__Pseudomonas|NA|402e5913597695a16d7cad415ffff02f"
## [2] "D_0__Bacteria|D_1__Proteobacteria|D_2__Gammaproteobacteria|D_3__Pseudomonadales|D_4__Pseudomonadaceae|D_5__Pseudomonas|NA|1f878f615fcfc8d7bd381a7841ac1e41"
## [3] "D_0__Bacteria|D_1__Proteobacteria|D_2__Alphaproteobacteria|D_3__Caulobacterales|D_4__Caulobacteraceae|D_5__Brevundimonas|NA|7b054f04ff8194f7a97cbbd55455ceef"
## [4] "D_0__Bacteria|D_1__Proteobacteria|D_2__Alphaproteobacteria|D_3__Rhizobiales|D_4__Rhizobiaceae|D_5__Allorhizobium-Neorhizobium-Pararhizobium-Rhizobium|NA|438dc2a2ea2405a782df5830ae62100e"
## [5] "D_0__Bacteria|D_1__Firmicutes|D_2__Clostridia|D_3__Clostridiales|D_4__Ruminococcaceae|D_5__Ruminococcaceae UCG-005|NA|e8120a2c1c4a6888ab33d17a033a6a56"

#multiple wilcox by sequencing batch
MW.p = apply(IgA_batchtrim75,1,
 function(x) wilcox.test(c(x)~metaIgA$Batch)$p.value)
p.res = data.frame(taxa=row.names(IgA_batchtrim75),MW.p)
# Perform multiple comparison correction using a given method of choice
p.res$rel.fdr <- p.adjust(p.res$MW.p, method="fdr")

#hits
seq_batch_hits <- p.res[which(p.res$rel.fdr<0.05),]$taxa
seq_batch_hits

## [1] "D_0__Bacteria|D_1__Proteobacteria|D_2__Gammaproteobacteria|D_3__Pseudomonadales|D_4__Pseudomonadaceae|D_5__Pseudomonas|NA|402e5913597695a16d7cad415ffff02f"
## [2] "D_0__Bacteria|D_1__Proteobacteria|D_2__Gammaproteobacteria|D_3__Pseudomonadales|D_4__Pseudomonadaceae|D_5__Pseudomonas|NA|1f878f615fcfc8d7bd381a7841ac1e41"
## [3] "D_0__Bacteria|D_1__Proteobacteria|D_2__Alphaproteobacteria|D_3__Caulobacterales|D_4__Caulobacteraceae|D_5__Brevundimonas|NA|7b054f04ff8194f7a97cbbd55455ceef"
## [4] "D_0__Bacteria|D_1__Proteobacteria|D_2__Alphaproteobacteria|D_3__Rhizobiales|D_4__Rhizobiaceae|D_5__Allorhizobium-Neorhizobium-Pararhizobium-Rhizobium|NA|438dc2a2ea2405a782df5830ae62100e"
## [5] "D_0__Bacteria|D_1__Bacteroidetes|D_2__Bacteroidia|D_3__Bacteroidales|D_4__Prevotellaceae|D_5__Prevotella 9|NA|4bf3198c78397be5af0b7325d20558de"

## Fig S4 and Supplemental Data: multiple testing of IgA Index - looped corrected models (ASV level)

### Pre-process data frames and functions for the linear models

#define a function to extract the p value from the linear model summary
lmp <- function (modelobject) {
 if (class(modelobject) != "lm") stop("Not an object of class 'lm' ")
 f <- summary(modelobject)$fstatistic
 p <- pf(f[1],f[2],f[3],lower.tail=F)
 attributes(p) <- NULL
 return(p)
}

#select matching IgA and metadata files for testing #filter and adjust as needed here
IgA_wilcox <- IgA_batchtrim75_t #the variables measured
meta_wilcox <- as.data.frame(sample_data(IgPos_forindex)) #the study metadata
row.names(IgA_wilcox) <- meta_wilcox$ChildID

#add depth to test
IgPos <- subset_samples(Table_pruned6, Sort=="IgApos")
IgNeg <- subset_samples(Table_pruned6, Sort=="IgAneg")
sample_sums_pos <- sample_data(IgPos)$sample_sums
sample_sums_neg <- sample_data(IgNeg)$sample_sums
meta_wilcox$sample_sums_pos <- sample_sums_pos
meta_wilcox$sample_sums_neg <- sample_sums_neg
meta_wilcox$depth <- meta_wilcox$sample_sums_pos/meta_wilcox$sample_sums_neg

#clean up the messy taxa names
all_taxa_long <- names(IgA_wilcox)
code <- paste("taxa", 1:length(all_taxa_long), sep="")
colnames(IgA_wilcox) <- code
taxa.names <- data.frame(code, all_taxa_long)

#merge IgA and metadata for input into the model
df <- data.frame(IgA_wilcox, meta_wilcox)

#save a list of the variables to test
all_taxa <- names(IgA_wilcox)

# Full dataset, looped linear models, ASV level

### Country

#Country
model <- lapply(all_taxa, function(x) {
 lm(substitute(i ~ depth + Batch + age + sexe + Country, list(i = as.name(x))), data = df)})

#loop the summary output and save it (as a summary, not as a dataframe)
summary_loop <- lapply(model, summary)

#loop this function to the model to extract all p values (for total)
pval_model <- ldply(model, lmp) #preferable to lapply because it outputs a df
pval_variables <- ldply(model, function(x) (summary(x)$coefficient)[,4]) #provides individual p values for variables
df.lm <- data.frame(pval_model=pval_model$V1, pval_variables, taxa=all_taxa_long, code=code)

#FDR adjust the p-values for the whole model
df.lm$rel.fdr <- p.adjust(df.lm$pval_model, method="fdr")
#FDR adjust the p-values for the outcome variable of interest
df.lm$variable_FDR <- p.adjust(df.lm$Country, method="fdr")

#significant results
hits <- dplyr::filter(df.lm, variable_FDR<0.05)
hits

## pval_model X.Intercept. depth BatchBatch2 age sexeMasculin
## 1 4.209514e-08 0.65329133 0.5406755 0.008205236 0.04785018 0.2839423
## 2 6.265823e-03 0.07679008 0.2516234 0.031313733 0.26218765 0.8772793
## 3 8.084812e-03 0.93446773 0.8648369 0.851446574 0.48767302 0.2962590
## 4 4.450737e-03 0.04304278 0.7118425 0.254684711 0.23477905 0.1659481
## CountryRCA
## 1 4.307273e-09
## 2 8.143228e-04
## 3 4.889781e-04
## 4 2.767621e-04
## taxa
## 1 D_0__Bacteria|D_1__Firmicutes|D_2__Erysipelotrichia|D_3__Erysipelotrichales|D_4__Erysipelotrichaceae|D_5__Solobacterium|D_6__uncultured bacterium|4615dffd5616f456ec13b4b8b2e2c556
## 2 D_0__Bacteria|D_1__Firmicutes|D_2__Clostridia|D_3__Clostridiales|D_4__Ruminococcaceae|D_5__Subdoligranulum|NA|e553b9a0bb32467c71c89a4e97e55792
## 3 D_0__Bacteria|D_1__Firmicutes|D_2__Clostridia|D_3__Clostridiales|D_4__Ruminococcaceae|D_5__Subdoligranulum|NA|d2c0ca30e3b7c2d0deb4041a22dfaa65
## 4 D_0__Bacteria|D_1__Firmicutes|D_2__Clostridia|D_3__Clostridiales|D_4__Lachnospiraceae|D_5__Blautia|NA|c1dc9ad5116d96b8ed863458fc0d0aec
## code rel.fdr variable_FDR
## 1 taxa1 5.893319e-06 6.030182e-07
## 2 taxa81 1.114859e-01 2.850130e-02
## 3 taxa84 1.114859e-01 2.281898e-02
## 4 taxa96 1.038505e-01 1.937334e-02

#semi-significant results
hits_relaxed <- dplyr::filter(df.lm, variable_FDR<0.1)
hits_relaxed

## pval_model X.Intercept. depth BatchBatch2 age sexeMasculin
## 1 4.209514e-08 0.65329133 0.5406755 0.008205236 0.04785018 0.2839423
## 2 3.522448e-02 0.82559081 0.5901440 0.118390217 0.53840682 0.2840133
## 3 6.265823e-03 0.07679008 0.2516234 0.031313733 0.26218765 0.8772793
## 4 8.084812e-03 0.93446773 0.8648369 0.851446574 0.48767302 0.2962590
## 5 4.450737e-03 0.04304278 0.7118425 0.254684711 0.23477905 0.1659481
## CountryRCA
## 1 4.307273e-09
## 2 2.294081e-03
## 3 8.143228e-04
## 4 4.889781e-04
## 5 2.767621e-04
## taxa
## 1 D_0__Bacteria|D_1__Firmicutes|D_2__Erysipelotrichia|D_3__Erysipelotrichales|D_4__Erysipelotrichaceae|D_5__Solobacterium|D_6__uncultured bacterium|4615dffd5616f456ec13b4b8b2e2c556
## 2 D_0__Bacteria|D_1__Bacteroidetes|D_2__Bacteroidia|D_3__Bacteroidales|D_4__Tannerellaceae|D_5__Parabacteroides|D_6__Parabacteroides distasonis|f4f297232da0f8d7dae7f9c432501e22
## 3 D_0__Bacteria|D_1__Firmicutes|D_2__Clostridia|D_3__Clostridiales|D_4__Ruminococcaceae|D_5__Subdoligranulum|NA|e553b9a0bb32467c71c89a4e97e55792
## 4 D_0__Bacteria|D_1__Firmicutes|D_2__Clostridia|D_3__Clostridiales|D_4__Ruminococcaceae|D_5__Subdoligranulum|NA|d2c0ca30e3b7c2d0deb4041a22dfaa65
## 5 D_0__Bacteria|D_1__Firmicutes|D_2__Clostridia|D_3__Clostridiales|D_4__Lachnospiraceae|D_5__Blautia|NA|c1dc9ad5116d96b8ed863458fc0d0aec
## code rel.fdr variable_FDR
## 1 taxa1 5.893319e-06 6.030182e-07
## 2 taxa20 2.054761e-01 6.423426e-02
## 3 taxa81 1.114859e-01 2.850130e-02
## 4 taxa84 1.114859e-01 2.281898e-02
## 5 taxa96 1.038505e-01 1.937334e-02

### Plot country hits

#plot country hits
#Solobacterium 4615
pdata <- data.frame(IgA_batchtrim75_t$`D_0__Bacteria|D_1__Firmicutes|D_2__Erysipelotrichia|D_3__Erysipelotrichales|D_4__Erysipelotrichaceae|D_5__Solobacterium|D_6__uncultured bacterium|4615dffd5616f456ec13b4b8b2e2c556`
 ,meta_wilcox$Batch, meta_wilcox$Country2)
colnames(pdata) <- c("IgAIndex", "Batch", "Country")
p <- ggplot(pdata) + geom_boxplot(aes(x=Country, y=IgAIndex, fill=Country),
 outlier.shape=NA, outlier.size=NA)
p <- p + ggtitle("Solobacterium 461")
p <- p + theme_bw(base_size=16) + xlab(NULL)
p <- p + theme(plot.title = element_text(size=15))
p <- p + scale_fill_manual(values=c("lightskyblue1", "deepskyblue4"))
p <- p + ylim(c(-1, 1))
p <- p + annotate("text", x = 1.5, y=1, label = "p=6.0e-07", size=4)
p <- p + annotate("segment", x=1, xend=2, y=0.9, yend=0.9)
p <- p + guides(colour=FALSE, size=FALSE, shape=FALSE, fill=FALSE)
p


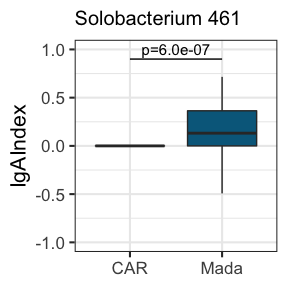


#Subdoligranulum e55
pdata <- data.frame(IgA_batchtrim75_t$`D_0__Bacteria|D_1__Firmicutes|D_2__Clostridia|D_3__Clostridiales|D_4__Ruminococcaceae|D_5__Subdoligranulum|NA|e553b9a0bb32467c71c89a4e97e55792`
 ,meta_wilcox$Batch, meta_wilcox$Country2)
colnames(pdata) <- c("IgAIndex", "Batch", "Country")
p <- ggplot(pdata) + geom_boxplot(aes(x=Country, y=IgAIndex, fill=Country),
 outlier.shape=NA, outlier.size=NA)
p <- p + ggtitle("Subdoligranulum e55")
p <- p + theme_bw(base_size=16) + xlab(NULL)
p <- p + theme(plot.title = element_text(size=15))
p <- p + scale_fill_manual(values=c("lightskyblue1", "deepskyblue4"))
p <- p + ylim(c(-1, 1))
p <- p + annotate("text", x = 1.5, y=1, label = "p=0.0285", size=4)
p <- p + annotate("segment", x=1, xend=2, y=0.9, yend=0.9)
p <- p + guides(colour=FALSE, size=FALSE, shape=FALSE, fill=FALSE)
p


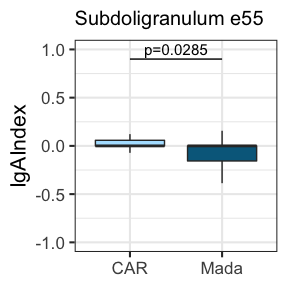


#Subdoligranulum d2c0
pdata <- data.frame(IgA_batchtrim75_t$`D_0__Bacteria|D_1__Firmicutes|D_2__Clostridia|D_3__Clostridiales|D_4__Ruminococcaceae|D_5__Subdoligranulum|NA|d2c0ca30e3b7c2d0deb4041a22dfaa65`
 ,meta_wilcox$Batch, meta_wilcox$Country2)
colnames(pdata) <- c("IgAIndex", "Batch", "Country")
p <- ggplot(pdata) + geom_boxplot(aes(x=Country, y=IgAIndex, fill=Country),
 outlier.shape=NA, outlier.size=NA)
p <- p + ggtitle("Subdoligranulum d2c")
p <- p + theme_bw(base_size=16) + xlab(NULL)
p <- p + theme(plot.title = element_text(size=15))
p <- p + scale_fill_manual(values=c("lightskyblue1", "deepskyblue4"))
p <- p + ylim(c(-1, 1))
p <- p + annotate("text", x = 1.5, y=1, label = "p=0.0281", size=4)
p <- p + annotate("segment", x=1, xend=2, y=0.9, yend=0.9)
p <- p + guides(colour=FALSE, size=FALSE, shape=FALSE, fill=FALSE)
p


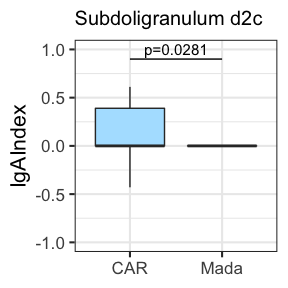


#Blautia c1d
pdata <- data.frame(IgA_batchtrim75_t$`D_0__Bacteria|D_1__Firmicutes|D_2__Clostridia|D_3__Clostridiales|D_4__Lachnospiraceae|D_5__Blautia|NA|c1dc9ad5116d96b8ed863458fc0d0aec`
 ,meta_wilcox$Batch, meta_wilcox$Country2)
colnames(pdata) <- c("IgAIndex", "Batch", "Country")
p <- ggplot(pdata) + geom_boxplot(aes(x=Country, y=IgAIndex, fill=Country),
 outlier.shape=NA, outlier.size=NA)
p <- p + ggtitle("Blautia c1d")
p <- p + theme_bw(base_size=16) + xlab(NULL)
p <- p + theme(plot.title = element_text(size=15))
p <- p + scale_fill_manual(values=c("lightskyblue1", "deepskyblue4"))
p <- p + ylim(c(-1, 1))
p <- p + annotate("text", x = 1.5, y=1, label = "p=0.0194", size=4)
p <- p + annotate("segment", x=1, xend=2, y=0.9, yend=0.9)
p <- p + guides(colour=FALSE, size=FALSE, shape=FALSE, fill=FALSE)
p


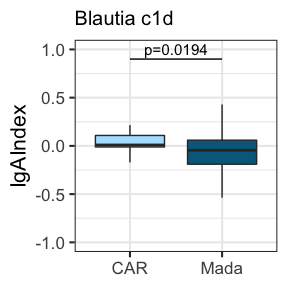


### HAZ, Age, & Breastfeeding

##Age
model <- lapply(all_taxa, function(x) {
 lm(substitute(i ~ depth + Batch + Country + sexe + age, list(i = as.name(x))), data = df)})

#loop the summary output and save it (as a summary, not as a dataframe)
summary_loop <- lapply(model, summary)

#loop this function to the model to extract all p values (for total)
pval_model <- ldply(model, lmp) #preferable to lapply because it outputs a df
pval_variables <- ldply(model, function(x) (summary(x)$coefficient)[,4]) #provides individual p values for variables
df.lm <- data.frame(pval_model=pval_model$V1, pval_variables, taxa=all_taxa_long, code=code)

#FDR adjust the p-values for the whole model
df.lm$rel.fdr <- p.adjust(df.lm$pval_model, method="fdr")
names(df.lm)

## [1] "pval_model" "X.Intercept." "depth" "BatchBatch2" "CountryRCA"
## [6] "sexeMasculin" "age" "taxa" "code" "rel.fdr"

#FDR adjust the p-values for the outcome variable of interest
df.lm$variable_FDR <- p.adjust(df.lm$age, method="fdr")

#significant results
hits <- dplyr::filter(df.lm, variable_FDR<0.05)
hits

## [1] pval_model X.Intercept. depth BatchBatch2 CountryRCA
## [6] sexeMasculin age taxa code rel.fdr
## [11] variable_FDR
## <0 rows> (or 0-length row.names)

#semi-significant results?
hits_relaxed <- dplyr::filter(df.lm, variable_FDR<0.1)
hits_relaxed

## pval_model X.Intercept. depth BatchBatch2 CountryRCA sexeMasculin
## 1 0.002452925 0.03974604 0.376893 0.02223155 0.3515142 0.2763848
## age
## 1 0.0006805284
## taxa
## 1 D_0__Bacteria|D_1__Firmicutes|D_2__Clostridia|D_3__Clostridiales|D_4__Ruminococcaceae|D_5__Intestinimonas|D_6__uncultured bacterium|ff016aa2413b286069636eff66376cbd
## code rel.fdr variable_FDR
## 1 taxa64 0.08585238 0.09527398

##HAZ
model <- lapply(all_taxa, function(x) {
 lm(substitute(i ~ depth + Batch + Country + age + sexe + haz_cont, list(i = as.name(x))), data = df)})

#loop the summary output and save it (as a summary, not as a dataframe)
summary_loop <- lapply(model, summary)

#loop this function to the model to extract all p values (for total)
pval_model <- ldply(model, lmp) #preferable to lapply because it outputs a df
pval_variables <- ldply(model, function(x) (summary(x)$coefficient)[,4]) #provides individual p values for variables
df.lm <- data.frame(pval_model=pval_model$V1, pval_variables, taxa=all_taxa_long)

#FDR adjust the p-values for the whole model
df.lm$rel.fdr <- p.adjust(df.lm$pval_model, method="fdr")
#FDR adjust the p-values for the outcome variable of interest
df.lm$variable_FDR <- p.adjust(df.lm$haz_cont, method="fdr")

#significant results
hits <- dplyr::filter(df.lm, variable_FDR<0.05)
hits

## [1] pval_model X.Intercept. depth BatchBatch2 CountryRCA
## [6] age sexeMasculin haz_cont taxa rel.fdr
## [11] variable_FDR
## <0 rows> (or 0-length row.names)

#semi-significant results?
hits_relaxed <- dplyr::filter(df.lm, variable_FDR<0.1)
hits_relaxed

## [1] pval_model X.Intercept. depth BatchBatch2 CountryRCA
## [6] age sexeMasculin haz_cont taxa rel.fdr
## [11] variable_FDR
## <0 rows> (or 0-length row.names)

##Stunting
model <- lapply(all_taxa, function(x) {
 lm(substitute(i ~ depth + Batch + Country + age + sexe + stunted, list(i = as.name(x))), data = df)})

#loop the summary output and save it (as a summary, not as a dataframe)
summary_loop <- lapply(model, summary)

#loop this function to the model to extract all p values (for total)
pval_model <- ldply(model, lmp) #preferable to lapply because it outputs a df
pval_variables <- ldply(model, function(x) (summary(x)$coefficient)[,4]) #provides individual p values for variables
df.lm <- data.frame(pval_model=pval_model$V1, pval_variables, taxa=all_taxa_long)

#FDR adjust the p-values for the whole model
df.lm$rel.fdr <- p.adjust(df.lm$pval_model, method="fdr")
#FDR adjust the p-values for the outcome variable of interest
df.lm$variable_FDR <- p.adjust(df.lm$stunted, method="fdr")

#significant results
hits <- dplyr::filter(df.lm, variable_FDR<0.05)
hits

## [1] pval_model X.Intercept. depth BatchBatch2 CountryRCA
## [6] age sexeMasculin stunted taxa rel.fdr
## [11] variable_FDR
## <0 rows> (or 0-length row.names)

#semi-significant results?
hits_relaxed <- dplyr::filter(df.lm, variable_FDR<0.1)
hits_relaxed

## [1] pval_model X.Intercept. depth BatchBatch2 CountryRCA
## [6] age sexeMasculin stunted taxa rel.fdr
## [11] variable_FDR
## <0 rows> (or 0-length row.names)

##Breastfeeding
model <- lapply(all_taxa, function(x) {
 lm(substitute(i ~ depth + Batch + Country + age + sexe + age_allaite, list(i = as.name(x))), data = df)})

#loop the summary output and save it (as a summary, not as a dataframe)
summary_loop <- lapply(model, summary)

#loop this function to the model to extract all p values (for total)
pval_model <- ldply(model, lmp) #preferable to lapply because it outputs a df
pval_variables <- ldply(model, function(x) (summary(x)$coefficient)[,4]) #provides individual p values for variables
df.lm <- data.frame(pval_model=pval_model$V1, pval_variables, taxa=all_taxa_long, code=code)

#FDR adjust the p-values for the whole model
df.lm$rel.fdr <- p.adjust(df.lm$pval_model, method="fdr")
#FDR adjust the p-values for the outcome variable of interest
df.lm$variable_FDR <- p.adjust(df.lm$age_allaite, method="fdr")

#significant results
hits <- dplyr::filter(df.lm, variable_FDR<0.05)
hits

## [1] pval_model X.Intercept. depth BatchBatch2 CountryRCA
## [6] age sexeMasculin age_allaite taxa code
## [11] rel.fdr variable_FDR
## <0 rows> (or 0-length row.names)

#semi-significant results?
hits_relaxed <- dplyr::filter(df.lm, variable_FDR<0.1)
hits_relaxed

## pval_model X.Intercept. depth BatchBatch2 CountryRCA age
## 1 0.02354185 0.2411863 0.5324124 0.6661052 0.5113298 0.03690794
## sexeMasculin age_allaite
## 1 0.5614665 0.0006549103
## taxa
## 1 D_0__Bacteria|D_1__Firmicutes|D_2__Clostridia|D_3__Clostridiales|D_4__Christensenellaceae|D_5__Christensenellaceae R-7 group|D_6__uncultured prokaryote|df15aa00cffdc5235078831e54deb6db
## code rel.fdr variable_FDR
## 1 taxa38 0.1432982 0.09168744

### Test significant hits (full dataset) by non-parametric methods

#bootstrap on regression coefficient
set.seed(1)

#taxa 1 (solobacterium) - bootstrap
N <- 1000
N2 <- 10000
boot_country1 <- boot(data=df, statistic=bs,
 R=N2, formula= taxa1 ~ depth + Batch + age + sexe + Country)
boot_country1

##
## ORDINARY NONPARAMETRIC BOOTSTRAP
##
##
## Call:
## boot(data = df, statistic = bs, R = N2, formula = taxa1 ~ depth +
## Batch + age + sexe + Country)
##
##
## Bootstrap Statistics :
## original bias std. error
## t1* 0.030381410 1.065404e-03 0.063270580
## t2* -0.017653970 -1.267369e-03 0.019403536
## t3* 0.088742952 8.901208e-04 0.037903442
## t4* 0.002577116 4.054170e-06 0.001247557
## t5* 0.032536084 -7.306810e-04 0.030336211
## t6* -0.202504387 -3.571679e-04 0.032769372

hist(boot_country1$t[,6])


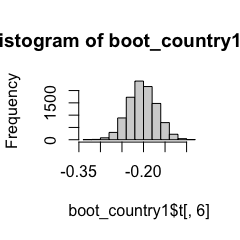


estimate <- boot_country1$t[,6]
p1 = (1 - sum((mean(estimate) + estimate) < mean(estimate)) / N2) *2
p1

## [1] 0

#taxa 1 (solobacterium) - simple wilcox
wilcox.test(df$taxa1~df$Country)

##
## Wilcoxon rank sum test with continuity correction
##
## data: df$taxa1 by df$Country
## W = 3668.5, p-value = 4.442e-11
## alternative hypothesis: true location shift is not equal to 0

#taxa 81 (Subdoligranulum e55)
boot_country2 <- boot(data=df, statistic=bs,
 R=N2, formula= taxa81 ~ depth + Batch + age + sexe + Country)
boot_country2

##
## ORDINARY NONPARAMETRIC BOOTSTRAP
##
##
## Call:
## boot(data = df, statistic = bs, R = N2, formula = taxa81 ~ depth +
## Batch + age + sexe + Country)
##
##
## Bootstrap Statistics :
## original bias std. error
## t1* -0.141755492 -4.024380e-03 0.092758535
## t2* 0.039031962 2.607915e-03 0.037329066
## t3* -0.084745830 -2.796951e-04 0.037174190
## t4* 0.001711254 3.451359e-05 0.001633328
## t5* 0.005510700 9.474727e-04 0.034862801
## t6* 0.129936314 3.777612e-04 0.036062378

hist(boot_country2$t[,6])


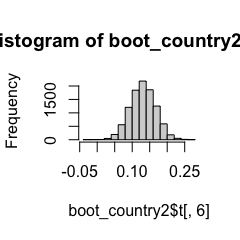


estimate <- boot_country2$t[,6]
p2 = (1 - sum((mean(estimate) + estimate) > mean(estimate)) / N2) *2
p2

## [1] 8e-04

#taxa 81 (subdologranulum e55) - simple wilcox
wilcox.test(df$taxa81~df$Country)

##
## Wilcoxon rank sum test with continuity correction
##
## data: df$taxa81 by df$Country
## W = 1664.5, p-value = 0.002064
## alternative hypothesis: true location shift is not equal to 0

#taxa84 (Subdoligranulum d2c)
boot_country3 <- boot(data=df, statistic=bs,
 R=N2, formula= taxa84 ~ depth + Batch + age + sexe + Country)
boot_country3

##
## ORDINARY NONPARAMETRIC BOOTSTRAP
##
##
## Call:
## boot(data = df, statistic = bs, R = N2, formula = taxa84 ~ depth +
## Batch + age + sexe + Country)
##
##
## Bootstrap Statistics :
## original bias std. error
## t1* -0.006688291 0.0019263879 0.076603214
## t2* 0.005905591 -0.0031748746 0.034744309
## t3* -0.007463879 0.0017887016 0.040618859
## t4* 0.001080391 0.0000174632 0.001507592
## t5* -0.038152772 -0.0001873339 0.037216258
## t6* 0.138486564 0.0002486448 0.039494662

hist(boot_country3$t[,6])


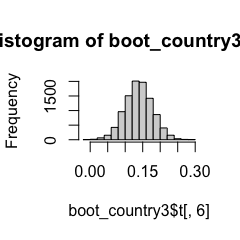


estimate <- boot_country3$t[,6]
p3 = (1 - sum((mean(estimate) + estimate) > mean(estimate)) / N2) *2
p3

## [1] 6e-04

#taxa84 (Subdoligranulum d2c) - wilcox
wilcox.test(df$taxa84~df$Country)

##
## Wilcoxon rank sum test with continuity correction
##
## data: df$taxa84 by df$Country
## W = 1611, p-value = 0.0001915
## alternative hypothesis: true location shift is not equal to 0

#taxa96 (Blautia c1d)
boot_country4 <- boot(data=df, statistic=bs,
 R=N2, formula= taxa96 ~ depth + Batch + age + sexe + Country)
boot_country4

##
## ORDINARY NONPARAMETRIC BOOTSTRAP
##
##
## Call:
## boot(data = df, statistic = bs, R = N2, formula = taxa96 ~ depth +
## Batch + age + sexe + Country)
##
##
## Bootstrap Statistics :
## original bias std. error
## t1* -0.169945416 7.598778e-03 0.083382981
## t2* 0.013133356 -9.627685e-03 0.045317666
## t3* -0.046626292 2.411206e-03 0.041184698
## t4* 0.001898479 2.745306e-05 0.001660676
## t5* 0.051930441 -4.238974e-04 0.037230601
## t6* 0.148279320 -7.840897e-04 0.038684687

hist(boot_country4$t[,6])


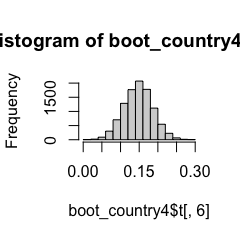


estimate <- boot_country4$t[,6]
p4 = (1 - sum((mean(estimate) + estimate) > mean(estimate)) / N2) *2
p4

## [1] 0

wilcox.test(df$taxa96~df$Country)

##
## Wilcoxon rank sum test with continuity correction
##
## data: df$taxa96 by df$Country
## W = 1489.5, p-value = 0.0002567
## alternative hypothesis: true location shift is not equal to 0

#taxa 64 (Intestinimonas) vs age
boot_age1 <- boot(data=df, statistic=bs,
 R=N2, formula= taxa64 ~ depth + Batch + Country + sexe + age)
boot_age1

##
## ORDINARY NONPARAMETRIC BOOTSTRAP
##
##
## Call:
## boot(data = df, statistic = bs, R = N2, formula = taxa64 ~ depth +
## Batch + Country + sexe + age)
##
##
## Bootstrap Statistics :
## original bias std. error
## t1* 0.143033585 -8.270670e-03 0.065623798
## t2* -0.026042163 8.809580e-03 0.047920078
## t3* 0.078067310 -1.895156e-03 0.033996872
## t4* -0.030717470 -7.677016e-04 0.031755126
## t5* 0.033732597 -8.878557e-05 0.030053852
## t6* -0.004582191 2.504720e-05 0.001199265

hist(boot_age1$t[,6])


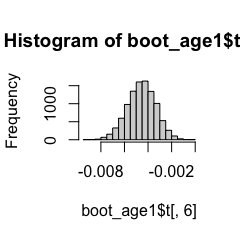


estimate <- boot_age1$t[,6]
p5 = (1 - sum((mean(estimate) + estimate) < mean(estimate)) / N2) *2
p5

## [1] 0

#simple spearman correlation
cor.test(df$taxa64, df$age, method='spearman')

##
## Spearman's rank correlation rho
##
## data: df$taxa64 and df$age
## S = 566920, p-value = 0.0004573
## alternative hypothesis: true rho is not equal to 0
## sample estimates:
## rho
## -0.2943713

#plot correlation - all - age - Intestinimonas
#taxa code
p <- ggplot(df, aes(x=age, y=taxa64)) + geom_point(shape=1, size=4) + geom_smooth(method=lm)
p <- p + ggtitle("Intestinimonas (All)")
p <- p + theme_bw(base_size=16)
p <- p + xlab("Age (months)") + ylab("IgA Index")
p <- p + ylim(-1,1)
p <- p + annotate("text", x = 55, y=0.8, label = "p=0.0004", size=4)
p <- p + annotate("text", x = 55, y=0.6, label = "rho=-0.29", size=4)
p

## `geom_smooth()` using formula 'y ~ x'


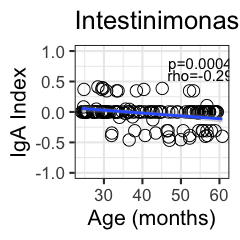


#remove zeros and re-test
df_ZeroesAsNA <- df
df_ZeroesAsNA[df_ZeroesAsNA==0] <- NA
cor.test(df_ZeroesAsNA$taxa64, df_ZeroesAsNA$age, method='spearman')

##
## Spearman's rank correlation rho
##
## data: df_ZeroesAsNA$taxa64 and df_ZeroesAsNA$age
## S = 24403, p-value = 0.000345
## alternative hypothesis: true rho is not equal to 0
## sample estimates:
## rho
## -0.5049632

p <- ggplot(df_ZeroesAsNA, aes(x=age, y=taxa64)) + geom_point(shape=1, size=4) + geom_smooth(method=lm)
p <- p + ggtitle("Intestinimonas (All)")
p <- p + theme_bw(base_size=16)
p <- p + xlab("Age (months)") + ylab("IgA Index")
p <- p + ylim(-1,1)
p <- p + annotate("text", x = 55, y=0.8, label = "p=0.0003", size=4)
p <- p + annotate("text", x = 55, y=0.6, label = "rho=-0.5", size=4)
p

## `geom_smooth()` using formula 'y ~ x'


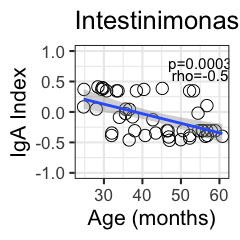


#taxa 38 (Christensenellaceae) vs breastfeeding
boot_allaite1 <- boot(data=df, statistic=bs,
 R=N2, formula= taxa64 ~ depth + Batch + Country + age + sexe + age_allaite)
boot_allaite1

##
## ORDINARY NONPARAMETRIC BOOTSTRAP
##
##
## Call:
## boot(data = df, statistic = bs, R = N2, formula = taxa64 ~ depth +
## Batch + Country + age + sexe + age_allaite)
##
##
## Bootstrap Statistics :
## original bias std. error
## t1* 0.092260437 -7.843570e-03 0.076911953
## t2* -0.025808632 8.960972e-03 0.047318692
## t3* 0.076466433 -2.204839e-03 0.034579630
## t4* -0.013155761 -4.885040e-04 0.033071925
## t5* -0.004800931 2.576643e-05 0.001265823
## t6* 0.042804651 7.932015e-04 0.031081148
## t7* 0.002175387 -5.614952e-05 0.002114242

estimate <- boot_allaite1$t[,7]
p6 = (1 - sum((mean(estimate) + estimate) > mean(estimate)) / N2) *2
p6

## [1] 0.3072

#simple spearman correlation
cor.test(df$taxa38, df$age_allaite, method='spearman')

##
## Spearman's rank correlation rho
##
## data: df$taxa38 and df$age_allaite
## S = 344517, p-value = 0.1045
## alternative hypothesis: true rho is not equal to 0
## sample estimates:
## rho
## 0.1408444

#plot correlation - all - breastfeeding - Christensenellaceae R-7
#taxa code
p <- ggplot(df, aes(x=age_allaite, y=taxa38)) + geom_point(shape=1, size=4) + geom_smooth(method=lm)
p <- p + ggtitle("Christensenellaceae (All)")
p <- p + theme_bw(base_size=16)
p <- p + xlab("Months of breastfeeding") + ylab("IgA Index")
p <- p + ylim(-1,1)
p <- p + annotate("text", x = 45, y=0.8, label = "p=0.104", size=4)
p <- p + annotate("text", x = 45, y=0.6, label = "rho=0.14", size=4)
p

## `geom_smooth()` using formula 'y ~ x'


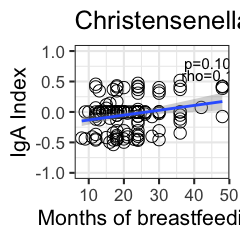


#remove zeros and re-test
cor.test(df_ZeroesAsNA$taxa38, df_ZeroesAsNA$age_allaite, method='spearman')

##
## Spearman's rank correlation rho
##
## data: df_ZeroesAsNA$taxa38 and df_ZeroesAsNA$age_allaite
## S = 71914, p-value = 0.02543
## alternative hypothesis: true rho is not equal to 0
## sample estimates:
## rho
## 0.2452678

p <- ggplot(df_ZeroesAsNA, aes(x=age_allaite, y=taxa38)) + geom_point(shape=1, size=4) + geom_smooth(method=lm)
p <- p + ggtitle("Christensenellaceae (All)")
p <- p + theme_bw(base_size=16)
p <- p + xlab("Months of breastfeeding") + ylab("IgA Index")
p <- p + ylim(-1,1)
p <- p + annotate("text", x = 45, y=0.8, label = "p=0.025", size=4)
p <- p + annotate("text", x = 45, y=0.6, label = "rho=0.24", size=4)
p

## `geom_smooth()` using formula 'y ~ x'


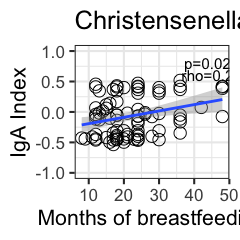


### Plot country differences for IgA hits, in unsorted relative abundance (from original 16S dataset)

##unsorted relative abundance
#data frames from before
dim(otuf_asv_t)

## [1] 113 452

dim(metaf_asv)

## [1] 113 405

#Subdoligranulum d2c0 (ASV) is a perfect match to the 16S data sequence "GQ871714.1.1480"
pdata <- data.frame(taxa=otuf_asv_t$`Subdoligranulum|uncultured_bacterium|GQ871714.1.1480`, Country=metaf_asv$Country2)
p <- ggplot(pdata) + geom_boxplot(aes(x=Country, y=taxa, fill=Country), outlier.shape=1, outlier.size=3)
p <- p + ggtitle("Subdoligranulum d2c")
p <- p + theme_bw(base_size=16)
p <- p + theme(plot.title = element_text(size=15))
p <- p + ylab("Relative Abundance") + xlab(NULL)
p <- p + scale_fill_manual(values=c("lightskyblue1", "deepskyblue4"))
p <- p + guides(colour=FALSE, size=FALSE, shape=FALSE, fill=FALSE)
p


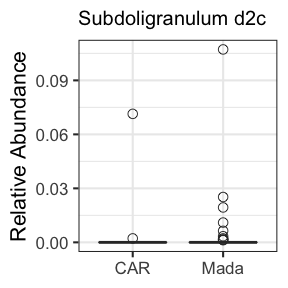


#Subdoligranulum e553b9a0bb32467c71c89a4e97e55792 is a perfect match to "BAAV01000882.904.2385"
pdata <- data.frame(taxa=otuf_asv_t$`Subdoligranulum|human_gut_metagenome|BAAZ01001428.1.1376`, Country=metaf_asv$Country2)
p <- ggplot(pdata) + geom_boxplot(aes(x=Country, y=taxa, fill=Country), outlier.shape=1, outlier.size=3)
p <- p + ggtitle("Subdoligranulum e55")
p <- p + theme_bw(base_size=16)
p <- p + theme(plot.title = element_text(size=15))
p <- p + ylab("Relative Abundance") + xlab(NULL)
p <- p + scale_fill_manual(values=c("lightskyblue1", "deepskyblue4"))
p <- p + guides(colour=FALSE, size=FALSE, shape=FALSE, fill=FALSE)
p


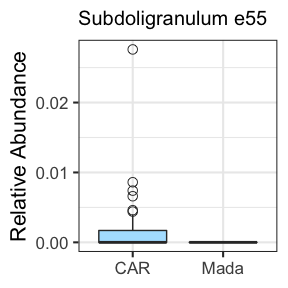


#Blautia (ASV) - c1d is a perfect match to the 16S data sequence "BABE01000454.586.2095"
pdata <- data.frame(taxa=otuf_asv_t$`Blautia|human_gut_metagenome|BABE01000454.586.2095`, Country=metaf_asv$Country2)
p <- ggplot(pdata) + geom_boxplot(aes(x=Country, y=taxa, fill=Country), outlier.shape=1, outlier.size=3)
p <- p + ggtitle("Blautia c1d")
p <- p + theme_bw(base_size=16)
p <- p + theme(plot.title = element_text(size=15))
p <- p + ylab("Relative Abundance") + xlab(NULL)
p <- p + scale_fill_manual(values=c("lightskyblue1", "deepskyblue4"))
p <- p + guides(colour=FALSE, size=FALSE, shape=FALSE, fill=FALSE)
p


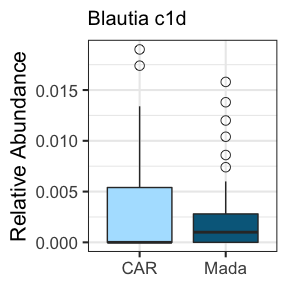


#Solobacterium (ASV) AAQK01001555.694.2198 #does not exist in filtered 16S dataset
#To retreive from un-filtered, un-rarefied dataset.
#there are 2 16S sequences with the same accession for some reason; the first hit, not the second, has 100% identity to the same 16S sequence in the IgA-sorting dataset
Fecal16S_pruned2_relabund <- transform_sample_counts(Fecal16S_pruned2, function(OTU) OTU/sum(OTU))
tax_unfilt <- as.data.frame(Fecal16S_pruned2_relabund@tax_table@.Data)
otu_unfilt <- as.data.frame(otu_table(Fecal16S_pruned2_relabund))
tax_unfilt$ASV <- row.names(tax_unfilt)
tax_unfilt$ASV==row.names(otu_unfilt) #TRUE

## [1] TRUE TRUE TRUE TRUE TRUE TRUE TRUE TRUE TRUE TRUE TRUE TRUE TRUE TRUE
## [15] TRUE TRUE TRUE TRUE TRUE TRUE TRUE TRUE TRUE TRUE TRUE TRUE TRUE TRUE
## [29] TRUE TRUE TRUE TRUE TRUE TRUE TRUE TRUE TRUE TRUE TRUE TRUE TRUE TRUE
## [43] TRUE TRUE TRUE TRUE TRUE TRUE TRUE TRUE TRUE TRUE TRUE TRUE TRUE TRUE
## [57] TRUE TRUE TRUE TRUE TRUE TRUE TRUE TRUE TRUE TRUE TRUE TRUE TRUE TRUE
## [71] TRUE TRUE TRUE TRUE TRUE TRUE TRUE TRUE TRUE TRUE TRUE TRUE TRUE TRUE
## [85] TRUE TRUE TRUE TRUE TRUE TRUE TRUE TRUE TRUE TRUE TRUE TRUE TRUE TRUE
## [99] TRUE TRUE TRUE TRUE TRUE TRUE TRUE TRUE TRUE TRUE TRUE TRUE TRUE TRUE
## [113] TRUE TRUE TRUE TRUE TRUE TRUE TRUE TRUE TRUE TRUE TRUE TRUE TRUE TRUE
## [127] TRUE TRUE TRUE TRUE TRUE TRUE TRUE TRUE TRUE TRUE TRUE TRUE TRUE TRUE
## [141] TRUE TRUE TRUE TRUE TRUE TRUE TRUE TRUE TRUE TRUE TRUE TRUE TRUE TRUE
## [155] TRUE TRUE TRUE TRUE TRUE TRUE TRUE TRUE TRUE TRUE TRUE TRUE TRUE TRUE
## [169] TRUE TRUE TRUE TRUE TRUE TRUE TRUE TRUE TRUE TRUE TRUE TRUE TRUE TRUE
## [183] TRUE TRUE TRUE TRUE TRUE TRUE TRUE TRUE TRUE TRUE TRUE TRUE TRUE TRUE
## [197] TRUE TRUE TRUE TRUE TRUE TRUE TRUE TRUE TRUE TRUE TRUE TRUE TRUE TRUE
## [211] TRUE TRUE TRUE TRUE TRUE TRUE TRUE TRUE TRUE TRUE TRUE TRUE TRUE TRUE
## [225] TRUE TRUE TRUE TRUE TRUE TRUE TRUE TRUE TRUE TRUE TRUE TRUE TRUE TRUE
## [239] TRUE TRUE TRUE TRUE TRUE TRUE TRUE TRUE TRUE TRUE TRUE TRUE TRUE TRUE
## [253] TRUE TRUE TRUE TRUE TRUE TRUE TRUE TRUE TRUE TRUE TRUE TRUE TRUE TRUE
## [267] TRUE TRUE TRUE TRUE TRUE TRUE TRUE TRUE TRUE TRUE TRUE TRUE TRUE TRUE
## [281] TRUE TRUE TRUE TRUE TRUE TRUE TRUE TRUE TRUE TRUE TRUE TRUE TRUE TRUE
## [295] TRUE TRUE TRUE TRUE TRUE TRUE TRUE TRUE TRUE TRUE TRUE TRUE TRUE TRUE
## [309] TRUE TRUE TRUE TRUE TRUE TRUE TRUE TRUE TRUE TRUE TRUE TRUE TRUE TRUE
## [323] TRUE TRUE TRUE TRUE TRUE TRUE TRUE TRUE TRUE TRUE TRUE TRUE TRUE TRUE
## [337] TRUE TRUE TRUE TRUE TRUE TRUE TRUE TRUE TRUE TRUE TRUE TRUE TRUE TRUE
## [351] TRUE TRUE TRUE TRUE TRUE TRUE TRUE TRUE TRUE TRUE TRUE TRUE TRUE TRUE
## [365] TRUE TRUE TRUE TRUE TRUE TRUE TRUE TRUE TRUE TRUE TRUE TRUE TRUE TRUE
## [379] TRUE TRUE TRUE TRUE TRUE TRUE TRUE TRUE TRUE TRUE TRUE TRUE TRUE TRUE
## [393] TRUE TRUE TRUE TRUE TRUE TRUE TRUE TRUE TRUE TRUE TRUE TRUE TRUE TRUE
## [407] TRUE TRUE TRUE TRUE TRUE TRUE TRUE TRUE TRUE TRUE TRUE TRUE TRUE TRUE
## [421] TRUE TRUE TRUE TRUE TRUE TRUE TRUE TRUE TRUE TRUE TRUE TRUE TRUE TRUE
## [435] TRUE TRUE TRUE TRUE TRUE TRUE TRUE TRUE TRUE TRUE TRUE TRUE TRUE TRUE
## [449] TRUE TRUE TRUE TRUE TRUE TRUE TRUE TRUE TRUE TRUE TRUE TRUE TRUE TRUE
## [463] TRUE TRUE TRUE TRUE TRUE TRUE TRUE TRUE TRUE TRUE TRUE TRUE TRUE TRUE
## [477] TRUE TRUE TRUE TRUE TRUE TRUE TRUE TRUE TRUE TRUE TRUE TRUE TRUE TRUE
## [491] TRUE TRUE TRUE TRUE TRUE TRUE TRUE TRUE TRUE TRUE TRUE TRUE TRUE TRUE
## [505] TRUE TRUE TRUE TRUE TRUE TRUE TRUE TRUE TRUE TRUE TRUE TRUE TRUE TRUE
## [519] TRUE TRUE TRUE TRUE TRUE TRUE TRUE TRUE TRUE TRUE TRUE TRUE TRUE TRUE
## [533] TRUE TRUE TRUE TRUE TRUE TRUE TRUE TRUE TRUE TRUE TRUE TRUE TRUE TRUE
## [547] TRUE TRUE TRUE TRUE TRUE TRUE TRUE TRUE TRUE TRUE TRUE TRUE TRUE TRUE
## [561] TRUE TRUE TRUE TRUE TRUE TRUE TRUE TRUE TRUE TRUE TRUE TRUE TRUE TRUE
## [575] TRUE TRUE TRUE TRUE TRUE TRUE TRUE TRUE TRUE TRUE TRUE TRUE TRUE TRUE
## [589] TRUE TRUE TRUE TRUE TRUE TRUE TRUE TRUE TRUE TRUE TRUE TRUE TRUE TRUE
## [603] TRUE TRUE TRUE TRUE TRUE TRUE TRUE TRUE TRUE TRUE TRUE TRUE TRUE TRUE
## [617] TRUE TRUE TRUE TRUE TRUE TRUE TRUE TRUE TRUE TRUE TRUE TRUE TRUE TRUE
## [631] TRUE TRUE TRUE TRUE TRUE TRUE TRUE TRUE TRUE TRUE TRUE TRUE TRUE TRUE
## [645] TRUE TRUE TRUE TRUE TRUE TRUE TRUE TRUE TRUE TRUE TRUE TRUE TRUE TRUE
## [659] TRUE TRUE TRUE TRUE TRUE TRUE TRUE TRUE TRUE TRUE TRUE TRUE TRUE TRUE
## [673] TRUE TRUE TRUE TRUE TRUE TRUE TRUE TRUE TRUE TRUE TRUE TRUE TRUE TRUE
## [687] TRUE TRUE TRUE TRUE TRUE TRUE TRUE TRUE TRUE TRUE TRUE TRUE TRUE TRUE
## [701] TRUE TRUE TRUE TRUE TRUE TRUE TRUE TRUE TRUE TRUE TRUE TRUE TRUE TRUE
## [715] TRUE TRUE TRUE TRUE TRUE TRUE TRUE TRUE TRUE TRUE TRUE TRUE TRUE TRUE
## [729] TRUE TRUE TRUE TRUE TRUE TRUE TRUE TRUE TRUE TRUE TRUE TRUE TRUE TRUE
## [743] TRUE TRUE TRUE TRUE TRUE TRUE TRUE TRUE TRUE TRUE TRUE TRUE TRUE TRUE
## [757] TRUE TRUE TRUE TRUE TRUE TRUE TRUE TRUE TRUE TRUE TRUE TRUE TRUE TRUE
## [771] TRUE TRUE TRUE TRUE TRUE TRUE TRUE TRUE TRUE TRUE TRUE TRUE TRUE TRUE
## [785] TRUE TRUE TRUE TRUE TRUE TRUE TRUE TRUE TRUE TRUE TRUE TRUE TRUE TRUE
## [799] TRUE TRUE TRUE TRUE TRUE TRUE TRUE TRUE TRUE TRUE TRUE TRUE TRUE TRUE
## [813] TRUE TRUE TRUE TRUE TRUE TRUE TRUE TRUE TRUE TRUE TRUE TRUE TRUE TRUE
## [827] TRUE TRUE TRUE TRUE TRUE TRUE TRUE TRUE TRUE TRUE TRUE TRUE TRUE TRUE
## [841] TRUE TRUE TRUE TRUE TRUE TRUE TRUE TRUE TRUE TRUE TRUE TRUE TRUE TRUE
## [855] TRUE TRUE TRUE TRUE TRUE TRUE TRUE TRUE TRUE TRUE TRUE TRUE TRUE TRUE
## [869] TRUE TRUE TRUE TRUE TRUE TRUE TRUE TRUE TRUE TRUE TRUE TRUE TRUE TRUE
## [883] TRUE TRUE TRUE TRUE TRUE TRUE TRUE TRUE TRUE TRUE TRUE TRUE TRUE TRUE
## [897] TRUE TRUE TRUE TRUE TRUE TRUE TRUE TRUE TRUE TRUE TRUE TRUE TRUE TRUE
## [911] TRUE TRUE TRUE TRUE TRUE TRUE TRUE TRUE TRUE TRUE TRUE TRUE TRUE TRUE
## [925] TRUE TRUE TRUE TRUE TRUE TRUE TRUE TRUE TRUE TRUE TRUE TRUE TRUE TRUE
## [939] TRUE TRUE TRUE TRUE TRUE TRUE TRUE TRUE TRUE TRUE TRUE TRUE TRUE TRUE
## [953] TRUE TRUE TRUE TRUE TRUE TRUE TRUE TRUE TRUE TRUE TRUE TRUE TRUE TRUE
## [967] TRUE TRUE TRUE TRUE TRUE TRUE TRUE TRUE TRUE TRUE TRUE TRUE TRUE TRUE
## [981] TRUE TRUE TRUE TRUE TRUE TRUE TRUE TRUE TRUE TRUE TRUE TRUE TRUE TRUE
## [995] TRUE TRUE TRUE TRUE TRUE TRUE TRUE TRUE TRUE TRUE TRUE TRUE TRUE TRUE
## [1009] TRUE TRUE TRUE TRUE TRUE TRUE TRUE TRUE TRUE TRUE TRUE TRUE TRUE TRUE
## [1023] TRUE TRUE TRUE TRUE TRUE TRUE TRUE TRUE TRUE TRUE TRUE TRUE TRUE TRUE
## [1037] TRUE TRUE TRUE TRUE TRUE TRUE TRUE TRUE TRUE TRUE TRUE TRUE TRUE TRUE
## [1051] TRUE TRUE TRUE TRUE TRUE TRUE TRUE TRUE TRUE TRUE TRUE TRUE TRUE TRUE
## [1065] TRUE TRUE TRUE TRUE TRUE TRUE TRUE TRUE TRUE TRUE TRUE TRUE TRUE TRUE
## [1079] TRUE TRUE TRUE TRUE TRUE TRUE TRUE TRUE TRUE TRUE TRUE TRUE TRUE TRUE
## [1093] TRUE TRUE TRUE TRUE TRUE TRUE TRUE TRUE TRUE TRUE TRUE TRUE TRUE TRUE
## [1107] TRUE TRUE TRUE TRUE TRUE TRUE TRUE TRUE TRUE TRUE TRUE TRUE TRUE TRUE
## [1121] TRUE TRUE TRUE TRUE TRUE TRUE TRUE TRUE TRUE TRUE TRUE TRUE TRUE TRUE
## [1135] TRUE TRUE TRUE TRUE TRUE TRUE TRUE TRUE TRUE TRUE TRUE TRUE TRUE TRUE
## [1149] TRUE TRUE TRUE TRUE TRUE TRUE TRUE TRUE TRUE TRUE TRUE TRUE TRUE TRUE
## [1163] TRUE TRUE TRUE TRUE TRUE TRUE TRUE TRUE TRUE TRUE TRUE TRUE TRUE TRUE
## [1177] TRUE TRUE TRUE TRUE TRUE TRUE TRUE TRUE TRUE TRUE TRUE TRUE TRUE TRUE
## [1191] TRUE TRUE TRUE TRUE TRUE TRUE TRUE TRUE TRUE TRUE TRUE TRUE TRUE TRUE
## [1205] TRUE TRUE TRUE TRUE TRUE TRUE TRUE TRUE TRUE TRUE TRUE TRUE TRUE TRUE
## [1219] TRUE TRUE TRUE TRUE TRUE TRUE TRUE TRUE TRUE TRUE TRUE TRUE TRUE TRUE
## [1233] TRUE TRUE TRUE TRUE

tax_unfilt$Simple <- paste(tax_unfilt$Rank6, tax_unfilt$Rank7, tax_unfilt$Accession, tax_unfilt$ASV, sep="|")
row.names(otu_unfilt) <- tax_unfilt$Simple
otu_unfilt_t <- as.data.frame(t(otu_unfilt))
metaf_unfilt <- as.data.frame(sample_data(Fecal16S_pruned2))

pdata <- data.frame(taxa=otu_unfilt_t$`Solobacterium|metagenome|AAQK01001555.694.2198|TACGTAGGTAGCGAGCGTTATCCGGAATTATTGGGCGTAAAGGGTGCGTAGGCGGCCTGTTAAGTTTATGGTGAAAGCGTGGGGCTCAACCCCATAAAGCCATAGATACTGGCAGGCTAGAGTACTGGAGAGGGTAGTGGAATTCCATGTGTAGCGGTAAAATGCGTAGATATATGGAGGAACACCGGTGGCGAAGGCGGCTACCTAGACAGAGACTGACGCTGAGGCACGAAAGCGTGGGGAGCAAATAGG`, Country=metaf_unfilt$Country2)
p <- ggplot(pdata) + geom_boxplot(aes(x=Country, y=taxa, fill=Country), outlier.shape=1, outlier.size=3)
p <- p + ggtitle("Solobacterium 461")
p <- p + theme_bw(base_size=16)
p <- p + theme(plot.title = element_text(size=15))
p <- p + ylab("Relative Abundance") + xlab(NULL)
p <- p + scale_fill_manual(values=c("lightskyblue1", "deepskyblue4"))
p <- p + guides(colour=FALSE, size=FALSE, shape=FALSE, fill=FALSE)
p


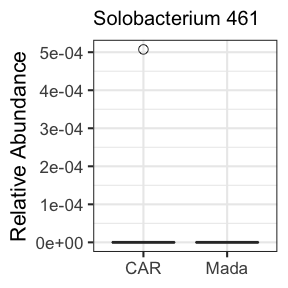


### Madagascar only, looped linear models, ASV level

##By country models##
#filter - Madagascar
meta_wilcox_filt = filter(meta_wilcox, Country=="Mada")
samples_kept <- meta_wilcox_filt$ChildID
IgA_wilcox_filt <- filter(IgA_batchtrim75_t, rownames(IgA_batchtrim75_t) %in% samples_kept)

#clean up the messy taxa names
all_taxa_long <- names(IgA_wilcox_filt)
code <- paste("taxa", 1:length(all_taxa_long), sep="")
colnames(IgA_wilcox_filt) <- code
taxa.names <- data.frame(code, all_taxa_long)

#merge IgA and metadata for input into the model
df <- data.frame(IgA_wilcox_filt, meta_wilcox_filt)

#save a list of the variables to test
all_taxa <- names(IgA_wilcox_filt)

##HAZ - Madagascar
model <- lapply(all_taxa, function(x) {
 lm(substitute(i ~ depth + Batch + age + sexe + haz_cont, list(i = as.name(x))), data = df)})

#loop the summary output and save it (as a summary, not as a dataframe)
summary_loop <- lapply(model, summary)

#loop this function to the model to extract all p values (for total)
pval_model <- ldply(model, lmp) #preferable to lapply because it outputs a df
pval_variables <- ldply(model, function(x) (summary(x)$coefficient)[,4]) #provides individual p values for variables
df.lm <- data.frame(pval_model=pval_model$V1, pval_variables, taxa=all_taxa_long)

#FDR adjust the p-values for the whole model
df.lm$rel.fdr <- p.adjust(df.lm$pval_model, method="fdr")
#FDR adjust the p-values for the outcome variable of interest
df.lm$haz_FDR <- p.adjust(df.lm$haz_cont, method="fdr")

#significant results
hits <- dplyr::filter(df.lm, haz_FDR<0.05)
hits

## pval_model X.Intercept. depth BatchBatch2 age sexeMasculin
## 1 0.007787581 0.08360266 0.6417374 0.4042954 0.7974676 0.5781771
## haz_cont
## 1 0.0002981328
## taxa
## 1 D_0__Bacteria|D_1__Firmicutes|D_2__Clostridia|D_3__Clostridiales|D_4__Lachnospiraceae|D_5__Lachnospiraceae NK4A136 group|D_6__uncultured organism|eb61cae65bc6cdd2440323bbf603ba5c
## rel.fdr haz_FDR
## 1 0.1557516 0.0417386

##Stunting
model <- lapply(all_taxa, function(x) {
 lm(substitute(i ~ depth + Batch + age + sexe + stunted, list(i = as.name(x))), data = df)})

#loop the summary output and save it (as a summary, not as a dataframe)
summary_loop <- lapply(model, summary)

#loop this function to the model to extract all p values (for total)
pval_model <- ldply(model, lmp) #preferable to lapply because it outputs a df
pval_variables <- ldply(model, function(x) (summary(x)$coefficient)[,4]) #provides individual p values for variables
df.lm <- data.frame(pval_model=pval_model$V1, pval_variables, taxa=all_taxa_long)

#FDR adjust the p-values for the whole model
df.lm$rel.fdr <- p.adjust(df.lm$pval_model, method="fdr")
#FDR adjust the p-values for the outcome variable of interest
df.lm$variable_FDR <- p.adjust(df.lm$stunted, method="fdr")

#significant results
hits <- dplyr::filter(df.lm, variable_FDR<0.05)
hits

## pval_model X.Intercept. depth BatchBatch2 age sexeMasculin
## 1 0.002932158 0.1566 0.5553517 0.5030581 0.6843926 0.7140929
## stunted
## 1 8.96459e-05
## taxa
## 1 D_0__Bacteria|D_1__Firmicutes|D_2__Clostridia|D_3__Clostridiales|D_4__Lachnospiraceae|D_5__Lachnospiraceae NK4A136 group|D_6__uncultured organism|eb61cae65bc6cdd2440323bbf603ba5c
## rel.fdr variable_FDR
## 1 0.1026255 0.01255043

#semi-significant results?
hits_relaxed <- dplyr::filter(df.lm, variable_FDR<0.1)
hits_relaxed

## pval_model X.Intercept. depth BatchBatch2 age sexeMasculin
## 1 0.002932158 0.1566 0.5553517 0.5030581 0.6843926 0.7140929
## stunted
## 1 8.96459e-05
## taxa
## 1 D_0__Bacteria|D_1__Firmicutes|D_2__Clostridia|D_3__Clostridiales|D_4__Lachnospiraceae|D_5__Lachnospiraceae NK4A136 group|D_6__uncultured organism|eb61cae65bc6cdd2440323bbf603ba5c
## rel.fdr variable_FDR
## 1 0.1026255 0.01255043

#Age - Madagascar
model <- lapply(all_taxa, function(x) {
 lm(substitute(i ~ depth + Batch + sexe + age, list(i = as.name(x))), data = df)})

#loop the summary output and save it (as a summary, not as a dataframe)
summary_loop <- lapply(model, summary)

#loop this function to the model to extract all p values (for total)
pval_model <- ldply(model, lmp) #preferable to lapply because it outputs a df
pval_variables <- ldply(model, function(x) (summary(x)$coefficient)[,4]) #provides individual p values for variables
df.lm <- data.frame(pval_model=pval_model$V1, pval_variables, taxa=all_taxa_long)

#FDR adjust the p-values for the whole model
df.lm$rel.fdr <- p.adjust(df.lm$pval_model, method="fdr")
#FDR adjust the p-values for the outcome variable of interest
df.lm$variable_FDR <- p.adjust(df.lm$age, method="fdr")

#significant results
hits <- dplyr::filter(df.lm, variable_FDR<0.05)
hits

## [1] pval_model X.Intercept. depth BatchBatch2 sexeMasculin
## [6] age taxa rel.fdr variable_FDR
## <0 rows> (or 0-length row.names)

#semi-significant results?
hits_relaxed <- dplyr::filter(df.lm, variable_FDR<0.1)
hits_relaxed

## [1] pval_model X.Intercept. depth BatchBatch2 sexeMasculin
## [6] age taxa rel.fdr variable_FDR
## <0 rows> (or 0-length row.names)

##Breastfeeding - Madagascar
model <- lapply(all_taxa, function(x) {
 lm(substitute(i ~ depth + Batch + age + sexe + age_allaite, list(i = as.name(x))), data = df)})

#loop the summary output and save it (as a summary, not as a dataframe)
summary_loop <- lapply(model, summary)

#loop this function to the model to extract all p values (for total)
pval_model <- ldply(model, lmp) #preferable to lapply because it outputs a df
pval_variables <- ldply(model, function(x) (summary(x)$coefficient)[,4]) #provides individual p values for variables
df.lm <- data.frame(pval_model=pval_model$V1, pval_variables, taxa=all_taxa_long)

#FDR adjust the p-values for the whole model
df.lm$rel.fdr <- p.adjust(df.lm$pval_model, method="fdr")
#FDR adjust the p-values for the outcome variable of interest
df.lm$variable_FDR <- p.adjust(df.lm$age_allaite, method="fdr")

#significant results
hits <- dplyr::filter(df.lm, variable_FDR<0.05)
hits

## [1] pval_model X.Intercept. depth BatchBatch2 age
## [6] sexeMasculin age_allaite taxa rel.fdr variable_FDR
## <0 rows> (or 0-length row.names)

#semi-significant results?
hits_relaxed <- dplyr::filter(df.lm, variable_FDR<0.1)
hits_relaxed

## [1] pval_model X.Intercept. depth BatchBatch2 age
## [6] sexeMasculin age_allaite taxa rel.fdr variable_FDR
## <0 rows> (or 0-length row.names)

### Test significant hits (Mada) by non-parametric methods

#taxa 108 (Lachno KK4A136) - bootstrap
N1 <- 1000
N2 <- 10000
boot_haz1 <- boot(data=df, statistic=bs,
 R=N2, formula= taxa108 ~ depth + Batch + age + sexe + haz_cont)
boot_haz1

##
## ORDINARY NONPARAMETRIC BOOTSTRAP
##
##
## Call:
## boot(data = df, statistic = bs, R = N2, formula = taxa108 ~ depth +
## Batch + age + sexe + haz_cont)
##
##
## Bootstrap Statistics :
## original bias std. error
## t1* -0.2336440472 -5.460698e-03 0.149971276
## t2* 0.0403210573 2.473142e-03 0.080926299
## t3* 0.0352714587 8.148763e-05 0.039376981
## t4* -0.0004688808 1.572815e-05 0.002020064
## t5* 0.0234747787 1.710030e-03 0.041932341
## t6* -0.0618457399 -7.502290e-04 0.018799133

hist(boot_haz1$t[,6])


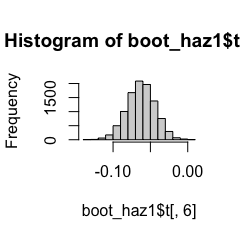


estimate <- boot_haz1$t[,6]
p1 = (1 - sum((mean(estimate) + estimate) < mean(estimate)) / N2) *2
p1

## [1] 4e-04

#taxa 108 (Lachno NK4A136) - simple spearman
cor.test(df$taxa108, df$haz_cont, method='spearman')

##
## Spearman's rank correlation rho
##
## data: df$taxa108 and df$haz_cont
## S = 113529, p-value = 6.708e-05
## alternative hypothesis: true rho is not equal to 0
## sample estimates:
## rho
## -0.4356422

#plot
p <- ggplot(df, aes(x=haz_cont, y=taxa108)) + geom_point(shape=1, size=4) + geom_smooth(method=lm)
p <- p + ggtitle("Lachno. NK4A136 (Mada)")
p <- p + theme_bw(base_size=16)
p <- p + xlab("HAZ") + ylab("IgA Index")
p <- p + ylim(-1, 1)
p <- p + annotate("text", x = 0, y=0.8, label = "p=6.7e-05", size=4)
p <- p + annotate("text", x = 0, y=0.6, label = "rho=-0.43", size=4)
p

## `geom_smooth()` using formula 'y ~ x'


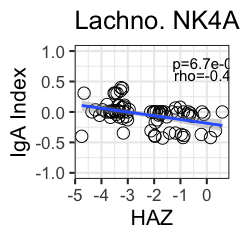


### CAR only, looped linear models, ASV level

#filter - RCA
meta_wilcox_filt = filter(meta_wilcox, Country=="RCA")
samples_kept <- meta_wilcox_filt$ChildID
IgA_wilcox_filt <- filter(IgA_batchtrim75_t, row.names(IgA_batchtrim75_t) %in% samples_kept)

#clean up the messy taxa names
all_taxa_long <- names(IgA_wilcox_filt)
code <- paste("taxa", 1:length(all_taxa_long), sep="")
colnames(IgA_wilcox_filt) <- code
taxa.names <- data.frame(code, all_taxa_long)

#merge IgA and metadata for input into the model
df <- data.frame(IgA_wilcox_filt, meta_wilcox_filt)

#save a list of the variables to test
all_taxa <- names(IgA_wilcox_filt)

##HAZ - RCA
model <- lapply(all_taxa, function(x) {
 lm(substitute(i ~ depth + Batch + Sorting_batch_major + age + sexe + haz_cont, list(i = as.name(x))), data = df)})

#loop the summary output and save it (as a summary, not as a dataframe)
summary_loop <- lapply(model, summary)

#loop this function to the model to extract all p values (for total)
pval_model <- ldply(model, lmp) #preferable to lapply because it outputs a df
pval_variables <- ldply(model, function(x) (summary(x)$coefficient)[,4]) #provides individual p values for variables
df.lm <- data.frame(pval_model=pval_model$V1, pval_variables, taxa=all_taxa_long)

#FDR adjust the p-values for the whole model
df.lm$rel.fdr <- p.adjust(df.lm$pval_model, method="fdr")
#FDR adjust the p-values for the outcome variable of interest
df.lm$haz_FDR <- p.adjust(df.lm$haz_cont, method="fdr")

#significant results
hits <- dplyr::filter(df.lm, haz_FDR<0.05)
hits

## [1] pval_model X.Intercept. depth
## [4] BatchBatch2 Sorting_batch_major age
## [7] sexeMasculin haz_cont taxa
## [10] rel.fdr haz_FDR
## <0 rows> (or 0-length row.names)

##Stunting
model <- lapply(all_taxa, function(x) {
 lm(substitute(i ~ depth + Batch + age + sexe + stunted, list(i = as.name(x))), data = df)})

#loop the summary output and save it (as a summary, not as a dataframe)
summary_loop <- lapply(model, summary)

#loop this function to the model to extract all p values (for total)
pval_model <- ldply(model, lmp) #preferable to lapply because it outputs a df
pval_variables <- ldply(model, function(x) (summary(x)$coefficient)[,4]) #provides individual p values for variables
df.lm <- data.frame(pval_model=pval_model$V1, pval_variables, taxa=all_taxa_long)

#FDR adjust the p-values for the whole model
df.lm$rel.fdr <- p.adjust(df.lm$pval_model, method="fdr")
#FDR adjust the p-values for the outcome variable of interest
df.lm$variable_FDR <- p.adjust(df.lm$stunted, method="fdr")

#significant results
hits <- dplyr::filter(df.lm, variable_FDR<0.05)
hits

## [1] pval_model X.Intercept. depth BatchBatch2 age
## [6] sexeMasculin stunted taxa rel.fdr variable_FDR
## <0 rows> (or 0-length row.names)

#semi-significant results?
hits_relaxed <- dplyr::filter(df.lm, variable_FDR<0.1)
hits_relaxed

## [1] pval_model X.Intercept. depth BatchBatch2 age
## [6] sexeMasculin stunted taxa rel.fdr variable_FDR
## <0 rows> (or 0-length row.names)

#Age - RCA
model <- lapply(all_taxa, function(x) {
 lm(substitute(i ~ depth + Batch + Sorting_batch_major + sexe + age, list(i = as.name(x))), data = df)})

#loop the summary output and save it (as a summary, not as a dataframe)
summary_loop <- lapply(model, summary)

#loop this function to the model to extract all p values (for total)
pval_model <- ldply(model, lmp) #preferable to lapply because it outputs a df
pval_variables <- ldply(model, function(x) (summary(x)$coefficient)[,4]) #provides individual p values for variables
df.lm <- data.frame(pval_model=pval_model$V1, pval_variables, taxa=all_taxa_long)

#FDR adjust the p-values for the whole model
df.lm$rel.fdr <- p.adjust(df.lm$pval_model, method="fdr")
#FDR adjust the p-values for the outcome variable of interest
df.lm$variable_FDR <- p.adjust(df.lm$age, method="fdr")

#significant results
hits <- dplyr::filter(df.lm, variable_FDR<0.05)
hits

## [1] pval_model X.Intercept. depth
## [4] BatchBatch2 Sorting_batch_major sexeMasculin
## [7] age taxa rel.fdr
## [10] variable_FDR
## <0 rows> (or 0-length row.names)

#semi-significant results?
hits_relaxed <- dplyr::filter(df.lm, variable_FDR<0.1)
hits_relaxed

## [1] pval_model X.Intercept. depth
## [4] BatchBatch2 Sorting_batch_major sexeMasculin
## [7] age taxa rel.fdr
## [10] variable_FDR
## <0 rows> (or 0-length row.names)

##Breastfeeding - RCA
model <- lapply(all_taxa, function(x) {
 lm(substitute(i ~ depth + Batch + Sorting_batch_major + age + sexe + age_allaite, list(i = as.name(x))), data = df)})

#loop the summary output and save it (as a summary, not as a dataframe)
summary_loop <- lapply(model, summary)

#loop this function to the model to extract all p values (for total)
pval_model <- ldply(model, lmp) #preferable to lapply because it outputs a df
pval_variables <- ldply(model, function(x) (summary(x)$coefficient)[,4]) #provides individual p values for variables
df.lm <- data.frame(pval_model=pval_model$V1, pval_variables, taxa=all_taxa_long)

#FDR adjust the p-values for the whole model
df.lm$rel.fdr <- p.adjust(df.lm$pval_model, method="fdr")
#FDR adjust the p-values for the outcome variable of interest
df.lm$variable_FDR <- p.adjust(df.lm$age_allaite, method="fdr")

#significant results
hits <- dplyr::filter(df.lm, variable_FDR<0.05)
hits

## [1] pval_model X.Intercept. depth
## [4] BatchBatch2 Sorting_batch_major age
## [7] sexeMasculin age_allaite taxa
## [10] rel.fdr variable_FDR
## <0 rows> (or 0-length row.names)

#semi-significant results?
hits_relaxed <- dplyr::filter(df.lm, variable_FDR<0.1)
hits_relaxed

## [1] pval_model X.Intercept. depth
## [4] BatchBatch2 Sorting_batch_major age
## [7] sexeMasculin age_allaite taxa
## [10] rel.fdr variable_FDR
## <0 rows> (or 0-length row.names)

# Percentage IgG-targeting

### Fig 4 A-F: Overall %IgG+ Bacteria

#to use the full metadata without excluding any samples for sequencing depth
metaIgG <- read.table("FinalAnalysis/metadata_2020-04-16.txt", header=TRUE)
metaIgG <- filter(metaIgG, (ChildID != "1429HMET018")) #no consent
metaIgG <- filter(metaIgG, Percent_IgG_clean!="NA")
metaIgG <- filter(metaIgG, !duplicated(ChildID))
dim(metaIgG) #75 children

## [1] 75 395

#distribution of data and number "IgGpos"
hist(metaIgG$Percent_IgG_clean)


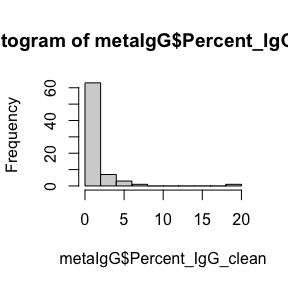


metaIgG$Percent_IgG_categ <- ifelse(metaIgG$Percent_IgG_clean >= 2,
 yes="IgG+", no="IgG-")
dplyr::count(metaIgG, Percent_IgG_categ)

## # A tibble: 2 x 2
## Percent_IgG_categ n
## <chr> <int>
## 1 IgG- 63
## 2 IgG+ 12

#clean factors into numbers
metaIgG$IgG1.Obs.Conc <- as.numeric(as.character(metaIgG$IgG1.Obs.Conc))
metaIgG$IgG2.Obs.Conc <- as.numeric(as.character(metaIgG$IgG2.Obs.Conc))
metaIgG$IgG3.Obs.Conc <- as.numeric(as.character(metaIgG$IgG3.Obs.Conc))
metaIgG$IgG4.Obs.Conc <- as.numeric(as.character(metaIgG$IgG4.Obs.Conc))
metaIgG$totalIgG <- metaIgG$IgG1.Obs.Conc + metaIgG$IgG2.Obs.Conc + metaIgG$IgG3.Obs.Conc + metaIgG$IgG4.Obs.Conc

#%IgG+ bacteria by total fecal IgG levels
cor.test(metaIgG$Percent_IgG_clean, metaIgG$totalIgG, method='spearman')

##
## Spearman's rank correlation rho
##
## data: metaIgG$Percent_IgG_clean and metaIgG$totalIgG
## S = 30191, p-value = 0.5948
## alternative hypothesis: true rho is not equal to 0
## sample estimates:
## rho
## 0.07131178

#plot correlation: %IgG vs total IgG
p <- ggplot(metaIgG, aes(x=Percent_IgG_clean, y=log(totalIgG))) + geom_point(shape=1, size=4) + geom_smooth(method=lm)
p <- p + ggtitle("Total IgG")
p <- p + theme_bw(base_size=16)
p <- p + xlab("%IgG+") + ylab("Log IgG Concentration")
p <- p + xlim(0, 8)
p <- p + annotate("text", x = 6, y=12, label = "p=0.60", size=4)
p <- p + annotate("text", x = 6, y=11, label = "rho=0.07", size=4)
p

## `geom_smooth()` using formula 'y ~ x'


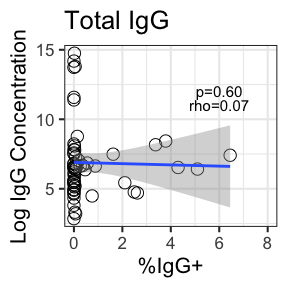


cor.test(metaIgG$Percent_IgG_clean, metaIgG$IgG1.Obs.Conc, method='spearman')

##
## Spearman's rank correlation rho
##
## data: metaIgG$Percent_IgG_clean and metaIgG$IgG1.Obs.Conc
## S = 30429, p-value = 0.1312
## alternative hypothesis: true rho is not equal to 0
## sample estimates:
## rho
## 0.1954146

cor.test(metaIgG$Percent_IgG_clean, metaIgG$IgG2.Obs.Conc, method='spearman')

##
## Spearman's rank correlation rho
##
## data: metaIgG$Percent_IgG_clean and metaIgG$IgG2.Obs.Conc
## S = 33790, p-value = 0.6427
## alternative hypothesis: true rho is not equal to 0
## sample estimates:
## rho
## 0.06112708

cor.test(metaIgG$Percent_IgG_clean, metaIgG$IgG3.Obs.Conc, method='spearman')

##
## Spearman's rank correlation rho
##
## data: metaIgG$Percent_IgG_clean and metaIgG$IgG3.Obs.Conc
## S = 31986, p-value = 0.1298
## alternative hypothesis: true rho is not equal to 0
## sample estimates:
## rho
## 0.194523

cor.test(metaIgG$Percent_IgG_clean, metaIgG$IgG4.Obs.Conc, method='spearman')

##
## Spearman's rank correlation rho
##
## data: metaIgG$Percent_IgG_clean and metaIgG$IgG4.Obs.Conc
## S = 33755, p-value = 0.1362
## alternative hypothesis: true rho is not equal to 0
## sample estimates:
## rho
## 0.1898238

#Country effect
wilcox.test(metaIgG$Percent_IgG_clean~metaIgG$Country)

##
## Wilcoxon rank sum test with continuity correction
##
## data: metaIgG$Percent_IgG_clean by metaIgG$Country
## W = 1002, p-value = 0.0008407
## alternative hypothesis: true location shift is not equal to 0

#plot binary: %IgG vs Country
p <- ggplot(metaIgG) + geom_jitter(aes(x=Country2, y=Percent_IgG_clean, color=IgG_sorted),
 width=0.2, height=0, shape=1, size=3)
p <- p + ggtitle("Country")
p <- p + theme_bw(base_size=16)
p <- p + ylab("%IgG+ Bacteria") + xlab("Country")
p <- p + scale_color_manual(values=c("black", "red"))
p <- p + annotate("text", x = 1.5, y=24, label = "p=0.0008", size=4)
p <- p + annotate("segment", x = 1, xend=2, y=23, yend=23)
p


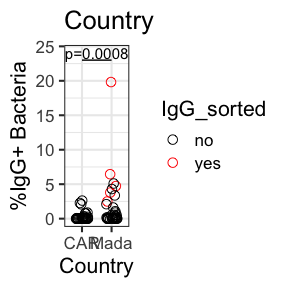


#age, sexe
cor.test(metaIgG$Percent_IgG_clean, metaIgG$age, method='spearman')

##
## Spearman's rank correlation rho
##
## data: metaIgG$Percent_IgG_clean and metaIgG$age
## S = 65244, p-value = 0.5398
## alternative hypothesis: true rho is not equal to 0
## sample estimates:
## rho
## 0.07191903

wilcox.test(metaIgG$Percent_IgG_clean~metaIgG$sexe)

##
## Wilcoxon rank sum test with continuity correction
##
## data: metaIgG$Percent_IgG_clean by metaIgG$sexe
## W = 668.5, p-value = 0.7918
## alternative hypothesis: true location shift is not equal to 0

#chronic undernutrition
cor.test(metaIgG$Percent_IgG_clean, metaIgG$haz_cont, method='spearman')

##
## Spearman's rank correlation rho
##
## data: metaIgG$Percent_IgG_clean and metaIgG$haz_cont
## S = 75265, p-value = 0.5471
## alternative hypothesis: true rho is not equal to 0
## sample estimates:
## rho
## -0.07063178

wilcox.test(metaIgG$Percent_IgG_clean~as.factor(metaIgG$stunted))

##
## Wilcoxon rank sum test with continuity correction
##
## data: metaIgG$Percent_IgG_clean by as.factor(metaIgG$stunted)
## W = 597, p-value = 0.3878
## alternative hypothesis: true location shift is not equal to 0

#plot binary: %IgG vs stunting
metaIgG$stunt_categ2 <- gsub("Nonstunted", "Non-stunted", metaIgG$stunt_categ2)
p <- ggplot(metaIgG) + geom_jitter(aes(x=stunt_categ2, y=Percent_IgG_clean, color=IgG_sorted),
 width=0.2, height=0, shape=1, size=3)
p <- p + ggtitle("Stunting")
p <- p + theme_bw(base_size=16)
p <- p + ylab("%IgG+ Bacteria") + xlab("Stunting")
p <- p + scale_color_manual(values=c("black", "red"))
p <- p + annotate("text", x = 1.5, y=24, label = "p=0.39", size=4)
p <- p + annotate("segment", x = 1, xend=2, y=23, yend=23)
p


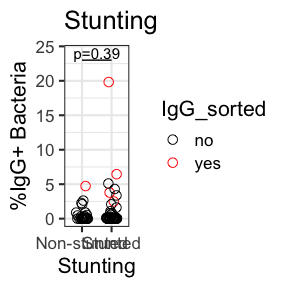


#inflammation
cor.test(metaIgG$Percent_IgG_clean, metaIgG$CALPROTECTINEggdePS, method='spearman')

##
## Spearman's rank correlation rho
##
## data: metaIgG$Percent_IgG_clean and metaIgG$CALPROTECTINEggdePS
## S = 51615, p-value = 0.4248
## alternative hypothesis: true rho is not equal to 0
## sample estimates:
## rho
## 0.0969207

cor.test(metaIgG$Percent_IgG_clean, metaIgG$AATmggdePS, method='spearman')

##
## Spearman's rank correlation rho
##
## data: metaIgG$Percent_IgG_clean and metaIgG$AATmggdePS
## S = 36648, p-value = 0.3473
## alternative hypothesis: true rho is not equal to 0
## sample estimates:
## rho
## 0.1203886

wilcox.test(metaIgG$Percent_IgG_clean~metaIgG$crp_seuil)

##
## Wilcoxon rank sum test with continuity correction
##
## data: metaIgG$Percent_IgG_clean by metaIgG$crp_seuil
## W = 216.5, p-value = 0.1522
## alternative hypothesis: true location shift is not equal to 0

#recent infection
wilcox.test(metaIgG$Percent_IgG_clean~metaIgG$atcd_diarrhee)

##
## Wilcoxon rank sum test with continuity correction
##
## data: metaIgG$Percent_IgG_clean by metaIgG$atcd_diarrhee
## W = 60, p-value = 0.1858
## alternative hypothesis: true location shift is not equal to 0

wilcox.test(metaIgG$Percent_IgG_clean~metaIgG$atcd_resp)

##
## Wilcoxon rank sum test with continuity correction
##
## data: metaIgG$Percent_IgG_clean by metaIgG$atcd_resp
## W = 60, p-value = 0.2842
## alternative hypothesis: true location shift is not equal to 0

#when was the recent diarrheal infection for the sorted samples?
metaIgG[metaIgG$IgG_sorted=='yes',]$atcd_diarrhee

## [1] "Non" "Non" "Oui" "Oui" "Non"

metaIgG[metaIgG$IgG_sorted=='yes',]$age_a_diarrhee #one child had diarrhea at a year old

## [1] NA NA 1 NA NA

metaIgG[metaIgG$IgG_sorted=='yes',]$age_m_diarrhee #one child had diarrhea at 3 months old

## [1] NA NA 0 3 NA

metaIgG[metaIgG$IgG_sorted=='yes',]$age #those two children were ~4.5 yrs and ~2.5 years at time of sampling

## [1] 31.29829 54.04872 32.41608 44.54746 33.92840

### Data processing: Create the IgG index (genus level)

#use un-rarefied data because read counts were lower in IgG-sorted samples
#rel abund
IgG_genus <- tax_glom(Table_pruned1, "Rank6")
IgG_genus <- transform_sample_counts(IgG_genus, function(OTU) OTU/sum(OTU))

#add a pseudocount to transformed samples
otu_table(IgG_genus) <- otu_table(IgG_genus) + 0.0000001
#subset
IgG_genusNeg_forindex = subset_samples(IgG_genus, Sort %in% c("IgGneg"))
IgG_genusPos_forindex = subset_samples(IgG_genus, Sort %in% c("IgGpos"))
#make into dataframes
IgG_genusNegOTU <- data.frame(otu_table(IgG_genusNeg_forindex))
IgG_genusPosOTU <- data.frame(otu_table(IgG_genusPos_forindex))
#now the dataframes should be the same dimensions.
dim(IgG_genusNegOTU)

## [1] 528 5

dim(IgG_genusPosOTU)

## [1] 528 5

#but they don't seem to be in the same order. Transpose, then "order".
IgG_genusNegOTU <- t(IgG_genusNegOTU)
IgG_genusNeg_Order <- IgG_genusNegOTU[ order(row.names(IgG_genusNegOTU)), ]
IgG_genusPosOTU <- t(IgG_genusPosOTU)
IgG_genusPos_Order <- IgG_genusPosOTU[ order(row.names(IgG_genusPosOTU)), ]

#Log transform and calculate IgG Index.
IgG_genusNegLog <- log(IgG_genusNeg_Order)
IgG_genusPosLog <- log(IgG_genusPos_Order)
IgG_genusIndex <- -(IgG_genusPosLog - IgG_genusNegLog)/(IgG_genusPosLog + IgG_genusNegLog)

#trim step to remove never-targeted taxa (length-1) or taxa that are zero-targeted in at least "x"% of samples (length*x)
IgG_genus_t <- as.data.frame(t(IgG_genusIndex))
IgG_genus_trim <- IgG_genus_t[rowSums(IgG_genus_t==0) <=(length(IgG_genus_t)-1), ]
dim(IgG_genus_trim) #120

## [1] 120 5

names(IgG_genus_trim) <- sample_data(IgG_genusPos_forindex)$ChildID

#add full taxonomy ID to the IgA Index
IgG_genusTAX <- as.data.frame(IgG_genusPos_forindex@tax_table@.Data)
IgG_genusTAX$ASV <- row.names(IgG_genusTAX)

TaxaToKeepG <- row.names(IgG_genus_trim)
IgG_genusTAX <- filter(IgG_genusTAX, ASV %in% TaxaToKeepG)
row.names(IgG_genus_trim) <- IgG_genusTAX$ASV

IgG_genusIndex_t <- as.data.frame(t(IgG_genus_trim))

### Fig 4G: IgG index heatmap

IgG_genusTAX$Simple <- gsub("D_5__", "", IgG_genusTAX$Rank6)
IgG_genusTAX$Simple <- ifelse(IgG_genusTAX$Simple=="uncultured"|IgG_genusTAX$Simple=="uncultured bacterium",
 yes = as.character(IgG_genusTAX$Rank5), no=IgG_genusTAX$Simple)
IgG_genusTAX$Simple <- gsub("D_4__", "", IgG_genusTAX$Simple)
IgG_genusTAX$Simple <- ifelse(IgG_genusTAX$Simple=="uncultured"|IgG_genusTAX$Simple=="uncultured bacterium",
 yes = as.character(IgG_genusTAX$Rank4), no=IgG_genusTAX$Simple)
IgG_genusTAX$Simple <- gsub("D_3__", "", IgG_genusTAX$Simple)

IgG_genus_trim$Taxa <- IgG_genusTAX$Simple

#Child 1
IgG.Child1 <- IgG_genus_trim[,c(1,6)]
IgG.Child1$ChildID <- c("AF57") #rename for better anonymity as per the new coded names
names(IgG.Child1) <- c("IgGIndex", "Taxa", "ChildID")
#order
IgG.Child1 <- IgG.Child1[order(IgG.Child1$IgGIndex, decreasing=TRUE),]
#top 10
TopTaxa1 <- head(IgG.Child1$Taxa, n=10)
#bottom 10
BottomTaxa1 <- tail(IgG.Child1$Taxa, n=10)
#filter
#IgG.Child1_filt <- dplyr::filter(IgG.Child1, Taxa %in% TopTaxa1 | Taxa %in% BottomTaxa1)

#Child2
IgG.Child2 <- IgG_genus_trim[,c(2,6)]
IgG.Child2$ChildID <- c("AF60")
names(IgG.Child2) <- c("IgGIndex", "Taxa", "ChildID")
#order
IgG.Child2 <- IgG.Child2[order(IgG.Child2$IgGIndex, decreasing=TRUE),]
#top 10
TopTaxa2 <- head(IgG.Child2$Taxa, n=10)
#bottom 10
BottomTaxa2 <- tail(IgG.Child2$Taxa, n=10)

#Child3
IgG.Child3 <- IgG_genus_trim[,c(3,6)]
IgG.Child3$ChildID <- c("AF61")
names(IgG.Child3) <- c("IgGIndex", "Taxa", "ChildID")
#order
IgG.Child3 <- IgG.Child3[order(IgG.Child3$IgGIndex, decreasing=TRUE),]
#top 10
TopTaxa3 <- head(IgG.Child3$Taxa, n=10)
#bottom 10
BottomTaxa3 <- tail(IgG.Child3$Taxa, n=10)

#Child4
IgG.Child4 <- IgG_genus_trim[,c(4,6)]
IgG.Child4$ChildID <- c("AF63")
names(IgG.Child4) <- c("IgGIndex", "Taxa", "ChildID")
#order
IgG.Child4 <- IgG.Child4[order(IgG.Child4$IgGIndex, decreasing=TRUE),]
#top 10
TopTaxa4 <- head(IgG.Child4$Taxa, n=10)
#bottom 10
BottomTaxa4 <- tail(IgG.Child4$Taxa, n=10)

#Child5
IgG.Child5 <- IgG_genus_trim[,c(5,6)]
IgG.Child5$ChildID <- c("AF91")
names(IgG.Child5) <- c("IgGIndex", "Taxa", "ChildID")
#order
IgG.Child5 <- IgG.Child5[order(IgG.Child5$IgGIndex, decreasing=TRUE),]
#top 10
TopTaxa5 <- head(IgG.Child5$Taxa, n=10)
#bottom 10
BottomTaxa5 <- tail(IgG.Child5$Taxa, n=10)

#join
IgG.Bubble <- Reduce(full_join, list(IgG.Child1, IgG.Child2, IgG.Child3, IgG.Child4, IgG.Child5))

## Joining, by = c("IgGIndex", "Taxa", "ChildID")
## Joining, by = c("IgGIndex", "Taxa", "ChildID")
## Joining, by = c("IgGIndex", "Taxa", "ChildID")
## Joining, by = c("IgGIndex", "Taxa", "ChildID")

dim(IgG.Bubble)

## [1] 600 3

#filter for top 10 top/bottom taxa per child
all_taxa <- c(TopTaxa1, TopTaxa2, TopTaxa3, TopTaxa4, TopTaxa5,
 BottomTaxa1, BottomTaxa2, BottomTaxa3, BottomTaxa4, BottomTaxa5)
IgG.Bubble_maintaxa <- dplyr::filter(IgG.Bubble, Taxa %in% all_taxa)
dim(IgG.Bubble_maintaxa) #320 x 3

## [1] 320 3

###bubble plot
p <- ggplot(IgG.Bubble_maintaxa, aes(x=ChildID, y=Taxa)) + geom_tile(aes(fill=IgGIndex))
p <- p + scale_fill_gradient2(low="deepskyblue4", mid="white", high="indianred4", midpoint=0)
p <- p + scale_y_discrete(limits = rev(levels(IgG.Bubble_maintaxa $Taxa)))
p <- p + theme(strip.background = element_rect(fill="gray85"),
 panel.background = element_rect(fill="white"),
 panel.border = element_rect(colour="black", linetype="solid", fill="transparent")
)
p <- p + coord_flip()
p <- p + theme(axis.text.x = element_text(angle=90, hjust=1, vjust=0.5),
 legend.key.size=unit(0.75, "cm"))
p


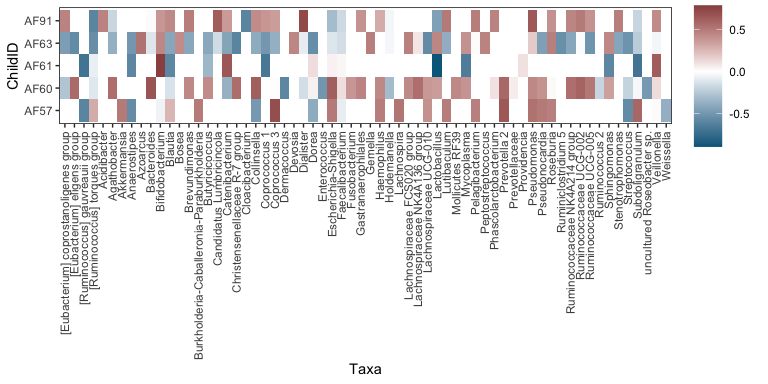

Supplement: Supplementary file 14 — Additional file 14:. Supplementary Files. File S1. R markdown code and statistical analysis used in this study [file 40168_2020_890_MOESM14_ESM.docx]
