# Supplementary material for: Immunoglobulin recognition of fecal bacteria in stunted and non-stunted children: findings from the Afribiota study
Source: Microbiome. 2020 Jul 27;8:113. doi: 10.1186/s40168-020-00890-1 (PMC7385872; doi:10.1186/s40168-020-00890-1)
Supplement: Supplementary file 2 [file 40168_2020_890_MOESM10_ESM.docx]

Table S1.

Description	Madagascar (N=78)	CAR (N=60)	*P*
**Sex**			
Male	37/78 (47.4%)	27/60 (45.0%)	0.8636
Female	41/78 (52.6%)	33/60 (55.0%)	
**Age**			
Median (months)	42.6	38.0	0.6770
2-3 years	28/78 (35.9%)	26/60 (43.3%)	
3-4 years	23/78 (29.5%)	12/60 (20.0%)	
4-5+ years	27/78 (34.6%)	21/60 (35.0%)	
**Nutritional status**			
Median HAZ score	-2.12	-1.90	0.7360
Median WHZ score	-0.40	-0.50	0.8839
Median hemoglobin (g/100 mL serum)	11.5	10.7	**0.0014**
Presence of anemia	22/77 (28.6%)	23/53 (43.4%)	**0.0019**
**Breastfeeding**			
Median duration (months)	24	18	**1.3e-06**
**Inflammation**			
Median AAT (mg/g dry fecal weight)	49.5	37.0	0.0809
Median calprotectin (µg/g dry fecal weight)	502	296	**0.0009**
Elevated CRP (> 10 mg/l serum)	7/77 (9.1%)	10/50 (20.0%)	0.1088
**Parasites**			
Presence of helminths	69/78 (88.5%)	1/47 (2.1%)	**2.2e-16**
Presence of *Giardia*	19/78 (24.4%)	9/47 (19.1%)	0.6584
**Sequencing Batch**			
Batch 1	40/78 (51.2%)	13/60 (21.7%)	**0.0004**
Batch 2	38/78 (48.7%)	47/60 (78.3%)	
