# Supplementary material for: Immunoglobulin recognition of fecal bacteria in stunted and non-stunted children: findings from the Afribiota study
Source: Microbiome. 2020 Jul 27;8:113. doi: 10.1186/s40168-020-00890-1 (PMC7385872; doi:10.1186/s40168-020-00890-1)
Supplement: Supplementary file 3 [file 40168_2020_890_MOESM11_ESM.docx]

Table S2.

Subset	Phylum	Genus
All, Mada, CAR	*Firmicutes*	*Streptococcus*
All, Mada	*Firmicutes*	*Dialister*
All, CAR	*Bacteroidetes*	*Parabacteroides*
All, CAR	*Bacteroidetes*	*Bacteroides*
All, Mada, CAR	*Proteobacteria*	*Pseudomonas*
All, Mada	*Proteobacteria*	*Stenotrophomonas*
All, Mada, CAR	*Proteobacteria*	*Brevundimonas*
All, Mada	*Proteobacteria*	*Pelagibacterium*
All	*Proteobacteria*	*Allorhizobium-Neorhizobium-Pararhizobium-Rhizobium*
All, Mada	*Firmicutes*	*Roseburia*
All, Mada	*Firmicutes*	*Agathobacter*
All, Mada, CAR	*Firmicutes*	*Butyricicoccus*
All, Mada, CAR	*Firmicutes*	*Faecalibacterium*
All, Mada	*Firmicutes*	*Blautia*
All, Mada, CAR	*Firmicutes*	*Lachnospiraceae* uncultured
All, Mada	*Bacteroidetes*	*Prevotella* 9
Mada	*Firmicutes*	*Christensenellaceae* R-7 group
CAR	*Firmicutes*	*Ruminococcus* 1
