# Supplementary material for: Immunoglobulin recognition of fecal bacteria in stunted and non-stunted children: findings from the Afribiota study
Source: Microbiome. 2020 Jul 27;8:113. doi: 10.1186/s40168-020-00890-1 (PMC7385872; doi:10.1186/s40168-020-00890-1)
Supplement: Supplementary file 4 [file 40168_2020_890_MOESM12_ESM.docx]

Table S3.

Batch(es)	Phylum	Genus
**Sort, Seq**	***Proteobacteria***	***Pseudomonas***
**Sort, Seq**	***Proteobacteria***	***Brevundimonas***
**Sort, Seq**	***Proteobacteria***	***Allorhizobium-Neorhizobium-Pararhizobium-Rhizob***[illegible]
**Seq**	***Bacteroidetes***	***Prevotella 9***
Sort	*Firmicutes*	*Ruminococcaceae* UCG-005
