# Supplementary material for: Immunoglobulin recognition of fecal bacteria in stunted and non-stunted children: findings from the Afribiota study
Source: Microbiome. 2020 Jul 27;8:113. doi: 10.1186/s40168-020-00890-1 (PMC7385872; doi:10.1186/s40168-020-00890-1)
Supplement: Supplementary file 5 [file 40168_2020_890_MOESM13_ESM.docx]

Table S4.

ChildID	HAZ	WHZ	History of diarrhea	AAT	Calpro	CRP
AF57	**-4.31**	-0.73	No	**>mean**	<mean	<=10
AF60	**-3.33**	-1.54	No	<mean	<mean	<=10
AF61	-1.78	0.53	**Yes**	<mean	**>mean**	<=10
AF63	**-3.29**	-0.21	**Yes**	**>mean**	<mean	<=10
AF91	**-3.38**	-0.55	No	**>mean**	**>mean**	<=10
