# Supplementary material for: Immunoglobulin recognition of fecal bacteria in stunted and non-stunted children: findings from the Afribiota study
Source: Microbiome. 2020 Jul 27;8:113. doi: 10.1186/s40168-020-00890-1 (PMC7385872; doi:10.1186/s40168-020-00890-1)
Supplement: Supplementary file 6 [file 40168_2020_890_MOESM1_ESM.docx]

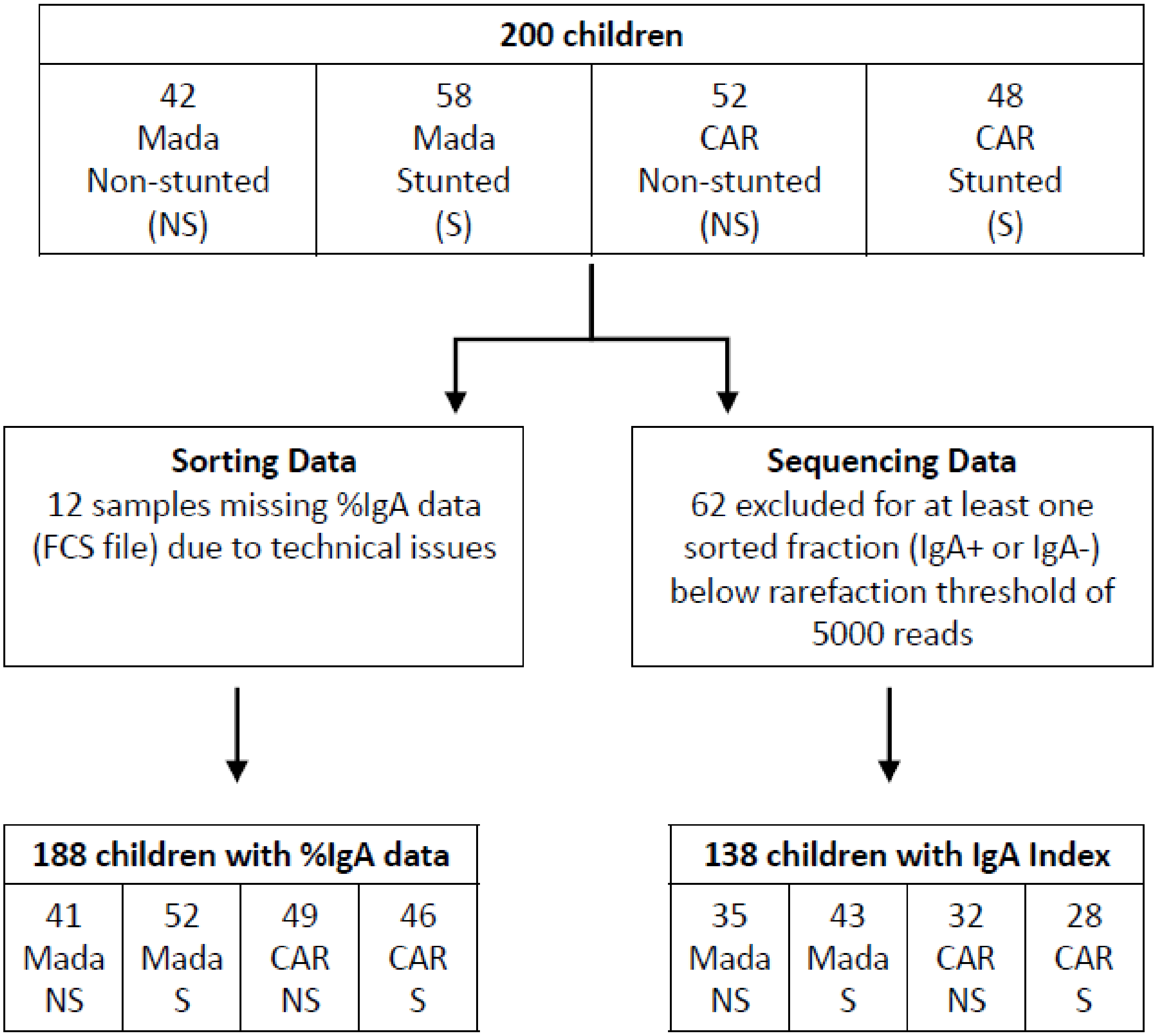
200 children
42 Mada Non-stunted (NS)
58 Mada Stunted (S)
52 CAR Non-stunted (NS)
48 CAR Stunted (S)
Sorting Data
12 samples missing %IgA data (FCS file) due to technical issues
Sequencing Data
62 excluded for at least one sorted fraction (IgA+ or IgA-) below rarefaction threshold of 5000 reads
188 children with %IgA data
41 Mada NS
52 Mada S
49 CAR NS
46 CAR S
138 children with IgA Index
35 Mada NS
43 Mada S
32 CAR NS
28 CAR S

Fig S1.
