# Supplementary material for: Immunoglobulin recognition of fecal bacteria in stunted and non-stunted children: findings from the Afribiota study
Source: Microbiome. 2020 Jul 27;8:113. doi: 10.1186/s40168-020-00890-1 (PMC7385872; doi:10.1186/s40168-020-00890-1)
Supplement: Supplementary file 7 [file 40168_2020_890_MOESM2_ESM.docx]

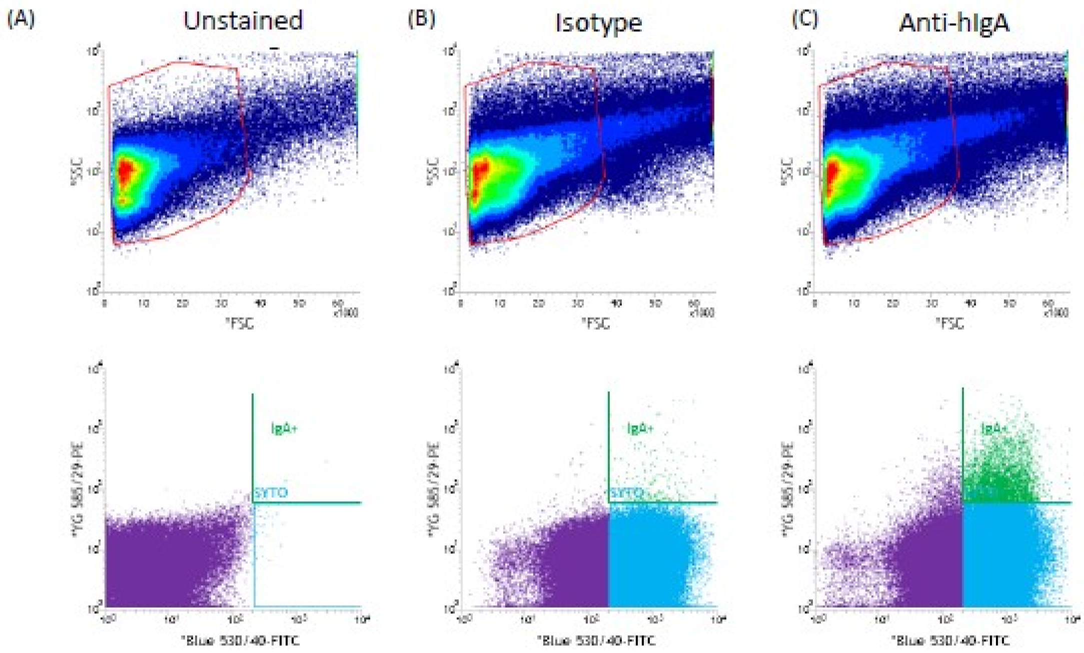
(A)
Unstained
(B)
Isotype
(C)
Anti-hIgA
*SSC
*FSC
*YG 585/29-PE
*Blue 530/40-FITC
IgA+
SYTO

**Fig S2.**
