# Supplementary material for: Immunoglobulin recognition of fecal bacteria in stunted and non-stunted children: findings from the Afribiota study
Source: Microbiome. 2020 Jul 27;8:113. doi: 10.1186/s40168-020-00890-1 (PMC7385872; doi:10.1186/s40168-020-00890-1)
Supplement: Supplementary file 8 [file 40168_2020_890_MOESM3_ESM.docx]

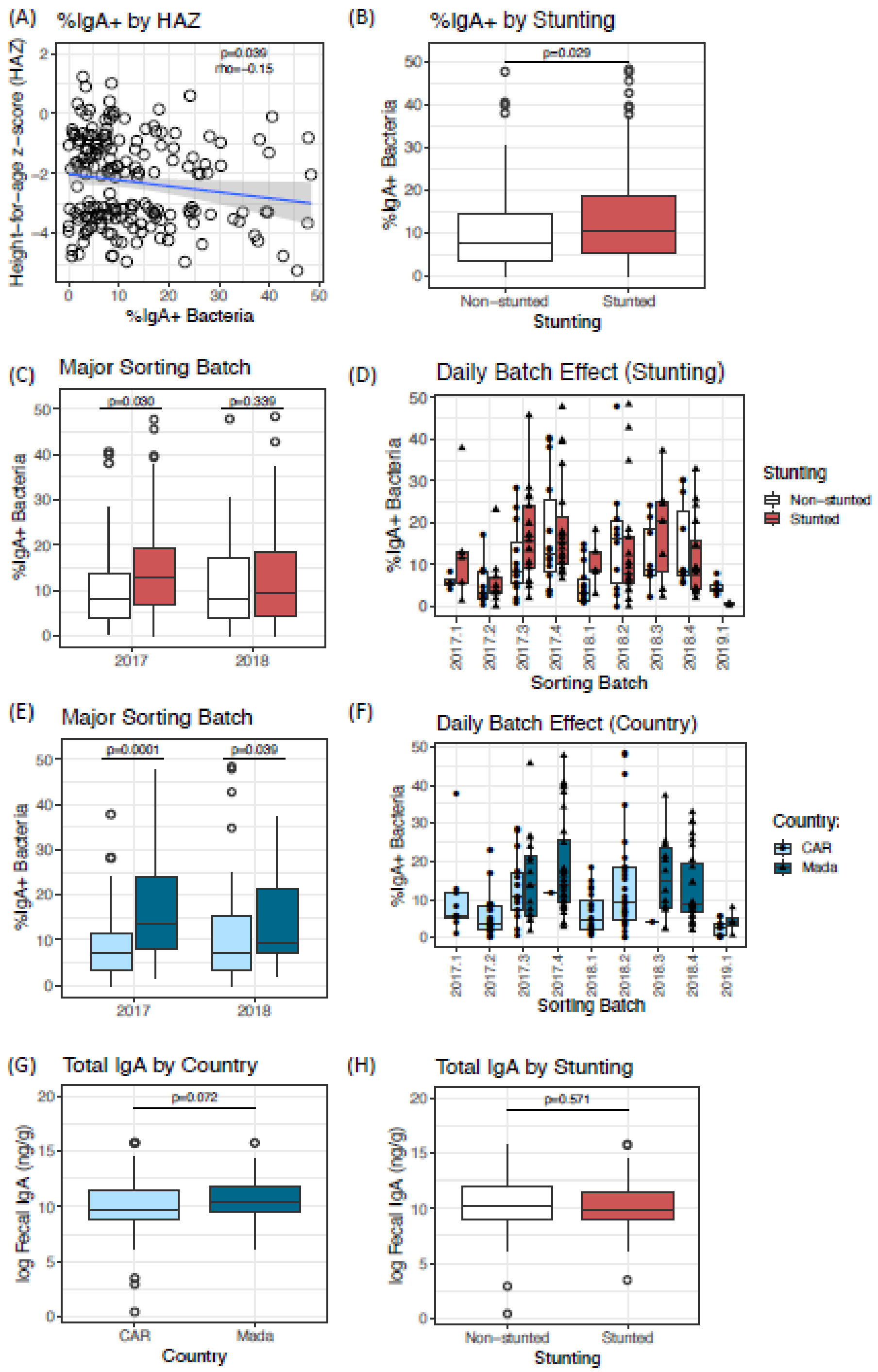

(A)
%IgA+ by HAZ
p=0.039
rho=-0.15
Height-for-age z-score (HAZ)
%IgA+ Bacteria
(B)
%IgA+ by Stunting
p=0.029
%IgA+ Bacteria
Non-stunted
Stunted
Stunting
(C)
Major Sorting Batch
p=0.030
p=0.339
%IgA+ Bacteria
2017
2018
(D)
Daily Batch Effect (Stunting)
%IgA+ Bacteria
Sorting Batch
Stunting
Non-stunted
Stunted
(E)
Major Sorting Batch
p=0.0001
p=0.039
%IgA+ Bacteria
2017
2018
(F)
Daily Batch Effect (Country)
%IgA+ Bacteria
Sorting Batch
Country:
CAR
Mada
(G)
Total IgA by Country
p=0.072
log Fecal IgA (ng/g)
CAR
Mada
Country
(H)
Total IgA by Stunting
p=0.571
log Fecal IgA (ng/g)
Non-stunted
Stunted
Stunting

Fig S3.
