# Supplementary material for: Immunoglobulin recognition of fecal bacteria in stunted and non-stunted children: findings from the Afribiota study
Source: Microbiome. 2020 Jul 27;8:113. doi: 10.1186/s40168-020-00890-1 (PMC7385872; doi:10.1186/s40168-020-00890-1)
Supplement: Supplementary file 9 [file 40168_2020_890_MOESM4_ESM.docx]

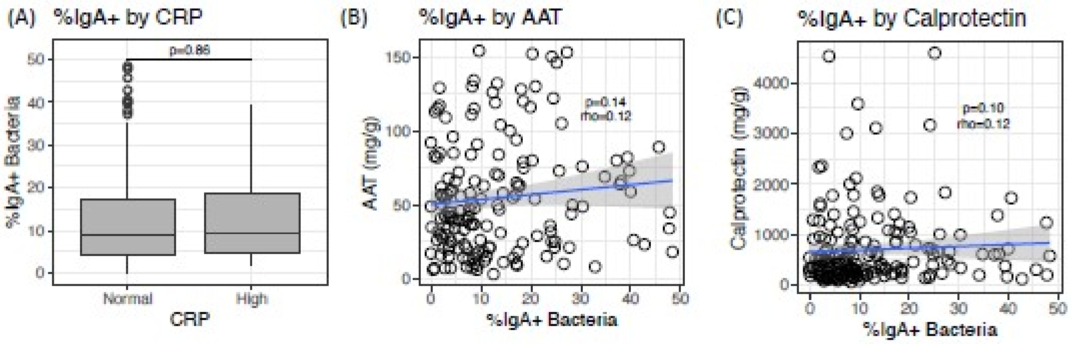
(A) %IgA+ by CRP
p=0.86
%IgA+ Bacteria
Normal
High
CRP
(B) %IgA+ by AAT
p=0.14
rho=0.12
AAT (mg/g)
%IgA+ Bacteria
(C) %IgA+ by Calprotectin
p=0.10
rho=0.12
Calprotectin (mg/g)
%IgA+ Bacteria

Fig S4.
