# Supplementary material for: Immunoglobulin recognition of fecal bacteria in stunted and non-stunted children: findings from the Afribiota study
Source: Microbiome. 2020 Jul 27;8:113. doi: 10.1186/s40168-020-00890-1 (PMC7385872; doi:10.1186/s40168-020-00890-1)
Supplement: Supplementary file 10 [file 40168_2020_890_MOESM5_ESM.docx]

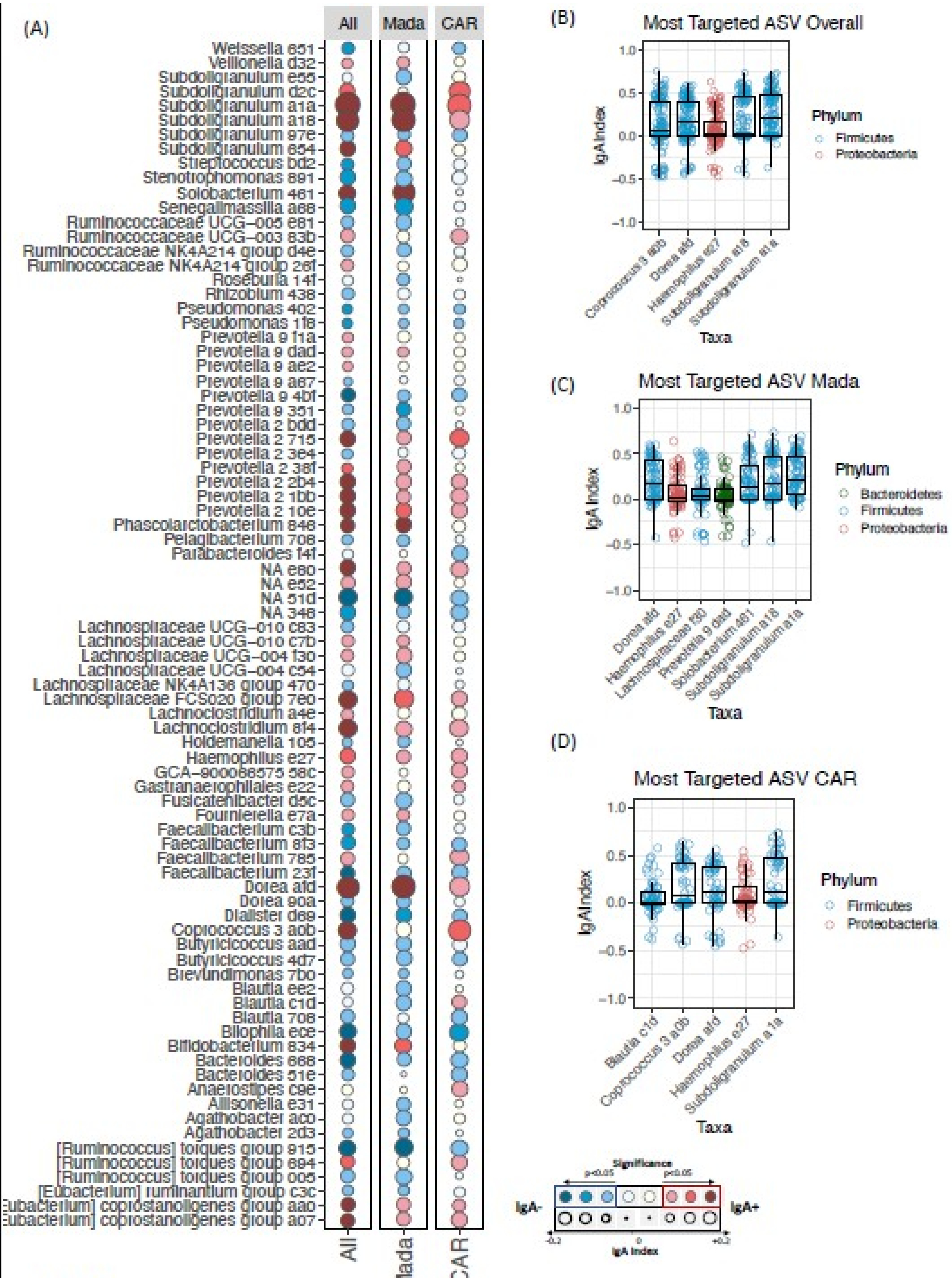

(A)
All
Mada
CAR
Weissella 651
Veillonella d32
Subdoligranulum e55
Subdoligranulum d2c
Subdoligranulum a1a
Subdoligranulum a18
Subdoligranulum 97e
Subdoligranulum 654
Streptococcus bd2
Stenotrophomonas 891
Solobacterium 461
Senegalimassilia a66
Ruminococcaceae UCG−005 e81
Ruminococcaceae UCG−003 83b
Ruminococcaceae NK4A214 group d4e
Ruminococcaceae NK4A214 group 28f
Roseburia 14f
Rhizobium 438
Pseudomonas 402
Pseudomonas 1f8
Prevotella 9 f1a
Prevotella 9 dad
Prevotella 9 ae2
Prevotella 9 a67
Prevotella 9 4bf
Prevotella 9 351
Prevotella 2 bdd
Prevotella 2 715
Prevotella 2 3e4
Prevotella 2 38f
Prevotella 2 2b4
Prevotella 2 1bb
Prevotella 2 10e
Phascolarctobacterium 846
Pelagibacterium 708
Parabacteroides f4f
NA e80
NA e52
NA 51d
NA 348
Lachnospiraceae UCG−010 c83
Lachnospiraceae UCG−010 c7b
Lachnospiraceae UCG−004 f30
Lachnospiraceae UCG−004 c54
Lachnospiraceae NK4A136 group 470
Lachnospiraceae FCS020 group 7e0
Lachnoclostridium a4e
Lachnoclostridium 6f4
Holdemanella 105
Haemophilus e27
GCA−900066575 58c
Gastranaerophilales e22
Fusicatenibacter d5c
Fournierella e7a
Faecalibacterium c3b
Faecalibacterium 8f3
Faecalibacterium 785
Faecalibacterium 23f
Dorea afd
Dorea 90a
Dialister d89
Coprococcus 3 a0b
Butyricicoccus aad
Butyricicoccus 4d7
Brevundimonas 7b0
Blautia ee2
Blautia c1d
Blautia 708
Bilophila ece
Bifidobacterium 834
Bacteroides 668
Bacteroides 51e
Anaerostipes c9e
Allisonella e31
Agathobacter ac0
Agathobacter 2d3
[Ruminococcus] torques group 915
[Ruminococcus] torques group 894
[Ruminococcus] torques group 005
[Eubacterium] ruminantium group c3c
Eubacterium] coprostanoligenes group aa0
Eubacterium] coprostanoligenes group a07
All
Mada
CAR
(B)
Most Targeted ASV Overall
IgA Index
1.0
0.5
0.0
−0.5
−1.0
Coprococcus 3 a0b
Dorea afd
Haemophilus e27
Subdoligranulum a18
Subdoligranulum a1a
Taxa
Phylum
Firmicutes
Proteobacteria
(C)
Most Targeted ASV Mada
IgA Index
1.0
0.5
0.0
−0.5
−1.0
Dorea afd
Haemophilus e27
Lachnospiraceae f30
Prevotella 9 dad
Solobacterium 461
Subdoligranulum a18
Subdoligranulum a1a
Taxa
Phylum
Bacteroidetes
Firmicutes
Proteobacteria
(D)
Most Targeted ASV CAR
IgA Index
1.0
0.5
0.0
−0.5
−1.0
Blautia c1d
Coprococcus 3 a0b
Dorea afd
Haemophilus e27
Subdoligranulum a1a
Taxa
Phylum
Firmicutes
Proteobacteria
Significance
p<0.05
p<0.05
IgA-
IgA+
-0.2
0
+0.2
IgA Index


Fig S5.
