# Supplementary material for: Immunoglobulin recognition of fecal bacteria in stunted and non-stunted children: findings from the Afribiota study
Source: Microbiome. 2020 Jul 27;8:113. doi: 10.1186/s40168-020-00890-1 (PMC7385872; doi:10.1186/s40168-020-00890-1)
Supplement: Supplementary file 11 [file 40168_2020_890_MOESM6_ESM.docx]

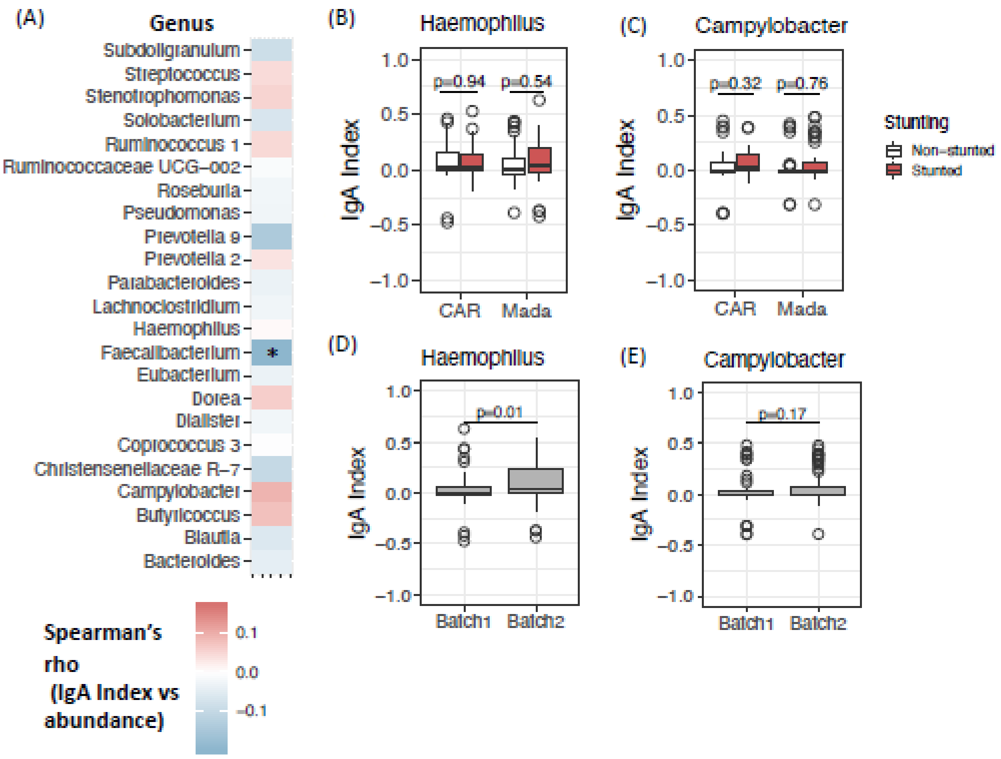

(A)
Genus
Subdoligranulum
Streptococcus
Stenotrophomonas
Solobacterium
Ruminococcus 1
Ruminococcaceae UCG−002
Roseburia
Pseudomonas
Prevotella 9
Prevotella 2
Parabacteroides
Lachnoclostridium
Haemophilus
Faecalibacterium
Eubacterium
Dorea
Dialister
Coprococcus 3
Christensenellaceae R−7
Campylobacter
Butyricoccus
Blautia
Bacteroides
*
Spearman's rho (IgA Index vs abundance)
0.1
0.0
−0.1
(B)
Haemophilus
IgA Index
p=0.94
p=0.54
CAR
Mada
(C)
Campylobacter
IgA Index
p=0.32
p=0.76
CAR
Mada
Stunting
Non−stunted
Stunted
(D)
Haemophilus
IgA Index
p=0.01
Batch1
Batch2
(E)
Campylobacter
IgA Index
p=0.17
Batch1
Batch2
1.0
0.5
0.0
−0.5
−1.0

Fig S6.
