# Supplementary material for: Immunoglobulin recognition of fecal bacteria in stunted and non-stunted children: findings from the Afribiota study
Source: Microbiome. 2020 Jul 27;8:113. doi: 10.1186/s40168-020-00890-1 (PMC7385872; doi:10.1186/s40168-020-00890-1)
Supplement: Supplementary file 12 [file 40168_2020_890_MOESM7_ESM.docx]

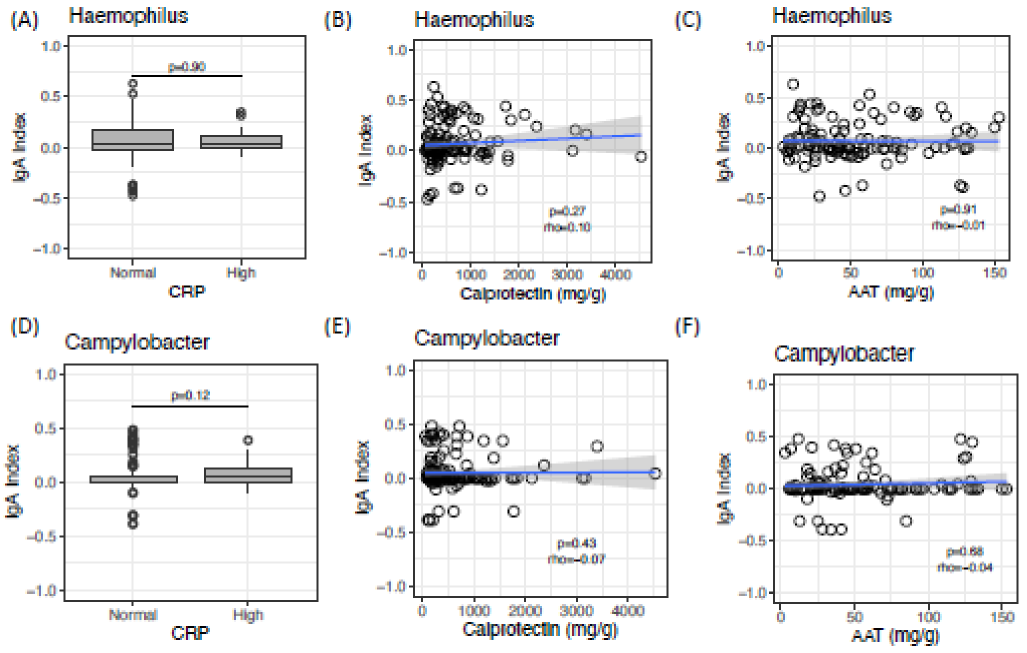
(A)
Haemophilus
p=0.90
IgA Index
Normal
High
CRP
(B)
Haemophilus
IgA Index
p=0.27
rho=0.10
Calprotectin (mg/g)
(C)
Haemophilus
IgA Index
p=0.91
rho=-0.01
AAT (mg/g)
(D)
Campylobacter
p=0.12
IgA Index
Normal
High
CRP
(E)
Campylobacter
IgA Index
p=0.43
rho=-0.07
Calprotectin (mg/g)
(F)
Campylobacter
IgA Index
p=0.68
rho=-0.04
AAT (mg/g)

Fig S7.
