# Supplementary material for: Immunoglobulin recognition of fecal bacteria in stunted and non-stunted children: findings from the Afribiota study
Source: Microbiome. 2020 Jul 27;8:113. doi: 10.1186/s40168-020-00890-1 (PMC7385872; doi:10.1186/s40168-020-00890-1)
Supplement: Supplementary file 13 [file 40168_2020_890_MOESM8_ESM.docx]

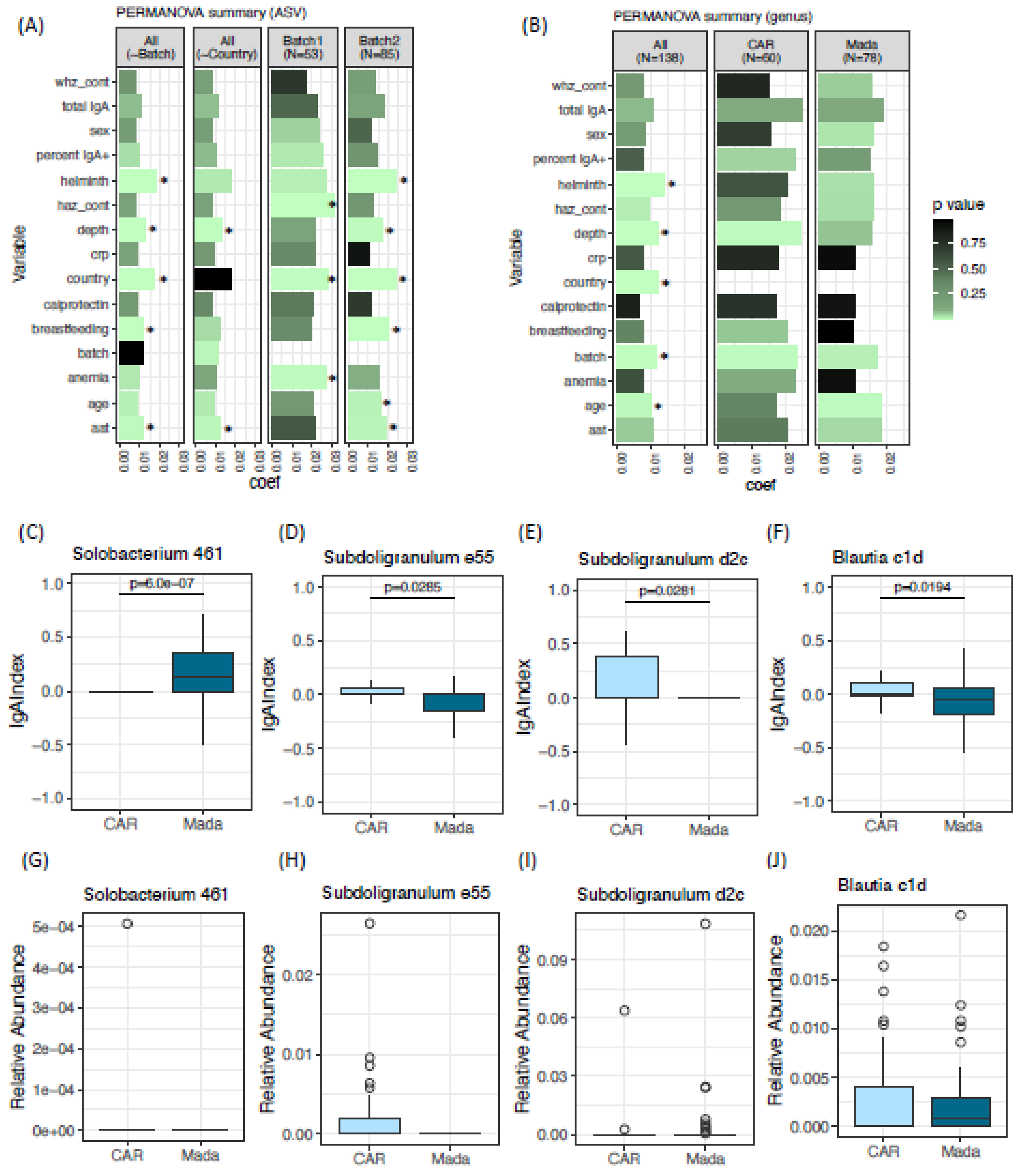

(A)
PERMANOVA summary (ASV)
All (~Batch)
All (~Country)
Batch1 (N=53)
Batch2 (N=85)
whz_cont
total IgA
sex
percent IgA+
helminth
haz_cont
depth
crp
country
calprotectin
breastfeeding
batch
anemia
age
aat
Variable
coef
(B)
PERMANOVA summary (genus)
All (N=138)
CAR (N=60)
Mada (N=78)
p value
0.75
0.50
0.25
(C)
Solobacterium 461
p=6.0e−07
IgAIndex
CAR
Mada
(D)
Subdoligranulum e55
p=0.0285
(E)
Subdoligranulum d2c
p=0.0281
(F)
Blautia c1d
p=0.0194
(G)
Solobacterium 461
Relative Abundance
(H)
Subdoligranulum e55
(I)
Subdoligranulum d2c
(J)
Blautia c1d

Fig S8.
