# Supplementary material for: Immunoglobulin recognition of fecal bacteria in stunted and non-stunted children: findings from the Afribiota study
Source: Microbiome. 2020 Jul 27;8:113. doi: 10.1186/s40168-020-00890-1 (PMC7385872; doi:10.1186/s40168-020-00890-1)
Supplement: Supplementary file 14 [file 40168_2020_890_MOESM9_ESM.docx]

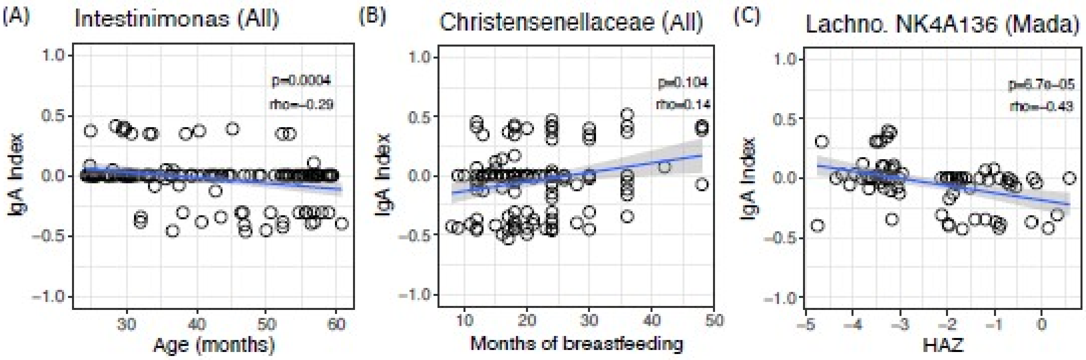
(A)
Intestinimonas (All)
p=0.0004
rho=-0.29
IgA Index
Age (months)
(B)
Christensenellaceae (All)
p=0.104
rho=0.14
IgA Index
Months of breastfeeding
(C)
Lachno. NK4A136 (Mada)
p=6.7e-05
rho=-0.43
IgA Index
HAZ

Fig S9.
